# Supplementary material for: STIM1 and ORAI1 form a novel cold transduction mechanism in sensory and sympathetic neurons
Source: EMBO J. 2022 Dec 16;42(3):e111348. doi: 10.15252/embj.2022111348 (PMC9890232; doi:10.15252/embj.2022111348)
Supplement: Supplementary file 1 — Appendix [file EMBJ-42-e111348-s007.docx]

**Appendix**

Table of contents

Page 2, Appendix Figure S1: Starting temperature of 37°C instead of 32°C does not affect the cold response of SCG neurons

Page 3, Appendix Figure S2: Individual fluorescence traces compared with F_340/380_ ratio traces.

Page 4, Appendix Figure S3: Cold-dependent change in solution pH does not explain calcium changes

Page 5, Appendix Figure S4: ORAI blocker YM58483 inhibits the novel cold-evoked calcium increase in DRG neurons independently of tachyphylaxis.

Page 6, Appendix FigureS5: The ORAI blocker MRS1845 blocks cold responses in the presence of Verapamil.

Page 7, Appendix Figure S6: Cold responses are potentiated by alkaline pH.

Page 8, Appendix Figure S7: Overexpression of STIM1 + ORAI1 increases cold-sensitivity of PC12 cells.

Page 9, Appendix Figure S8: Knockout of ORAI3 has no effect on cold responses of SCG neurons.

Page 10, Appendix Figure S9: Non-targeting siRNA does not affect responses of SCG neurons to cold.

Page 11, Appendix Figure S10: ORAI1-CFP and STIM1-YFP fluorescence is directly affected by cold.


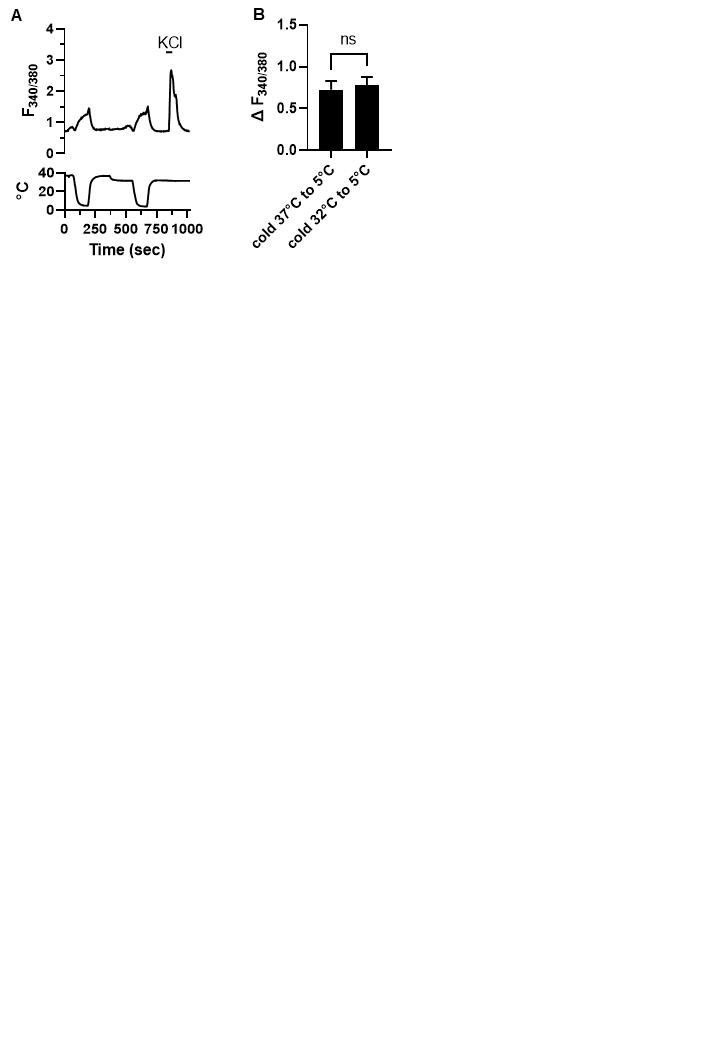


**Appendix Figure S1: Starting temperature of 37°C instead of 32°C does not affect the cold response of SCG neurons**

**(A)** Representative Ca^2+^ imaging trace showing cold responses of an SCG neuron with a starting temperature of 37°C or 32°C. Temperature trace shown below.

**(B)** Collected results of 27 cold-sensitive neurons on 8 coverslips. There is no significant difference in the amplitude of the cold response following the change in starting temperature from 37°C to 32°C (P=0.4909, paired t-test)


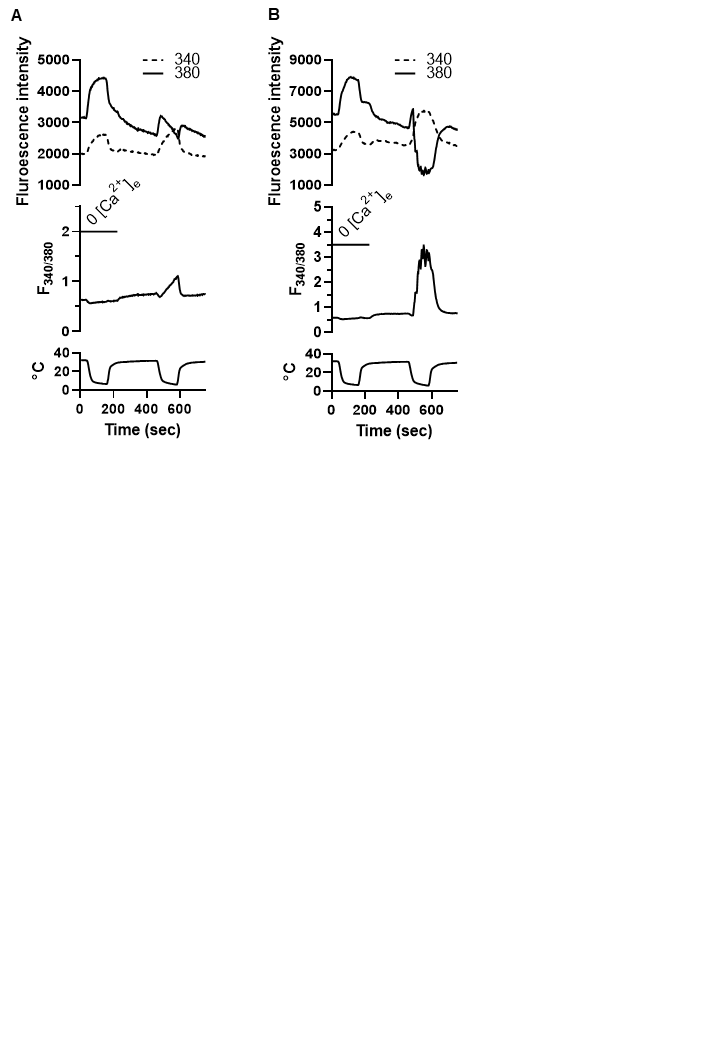


**Appendix Figure S2: Individual fluorescence traces compared with F_340/380_ ratio traces.**

Individual fluorescence traces (top) are significantly affected by cooling (bottom traces) but the ratio is stable (middle traces) apart from a consistent small decrease in F_340/380_ ratio when the temperature is lowered from 32°C to 4°C. Note that the small decrease in F_340/380_ ratio follows the time course of the temperature change in the absence of extracellular calcium, as expected for a physical change in dye properties, and that there is no change in F_340/380_ once the temperature has reached a stable low level. In contrast, in the presence of extracellular calcium significantly delayed changes in F_340/380_ ratio are observed. **(A)** shows a delayed increase in intracellular calcium consistent with activation of ORAI1 ion channels (see **Fig. 3**) while **(B)** shows a larger and more rapid increase consistent with activation of voltage-dependent Ca_V_ channels (see **Fig. 2**).


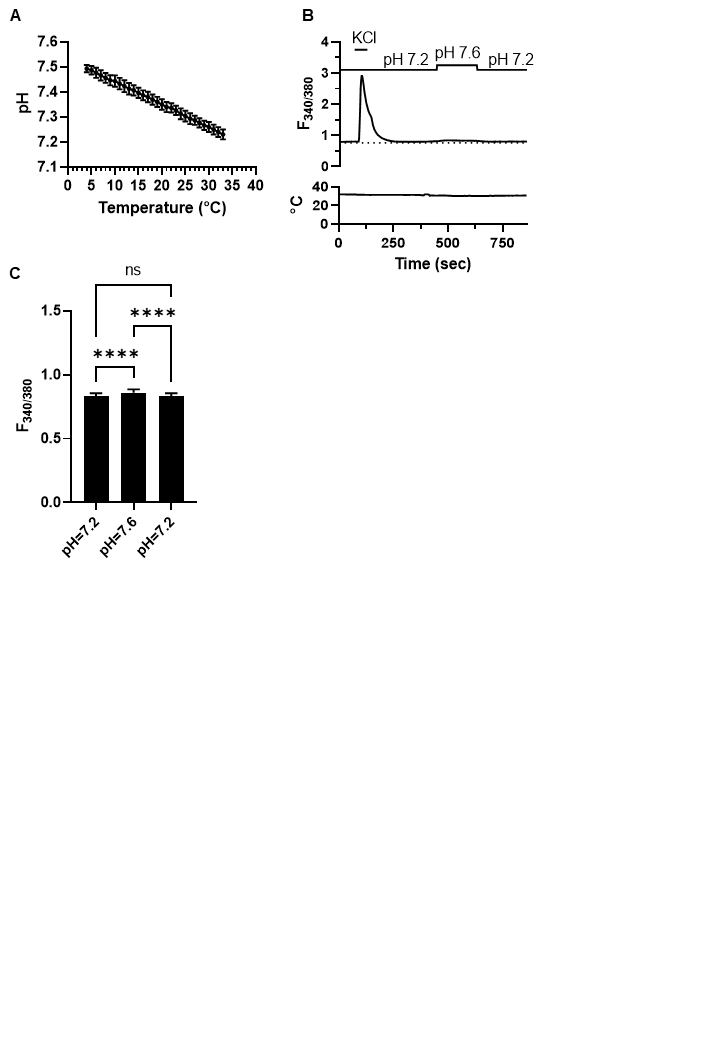


**Appendix Figure S3: Cold-dependent change in solution pH does not explain calcium changes**

**(A)** Reduction in temperature linearly increases the pH of the extracellular solution (n=3 independent measurements). **(B)** Representative trace of an SCG neuron exposed to pH 7.2 and pH 7.6. **(C)** Bar chart summarizing baseline F_340/380_ ratio from 65 SCG neurons using the protocol shown in (**B**). pH 7.6 induced a small but significant increase in the F_340/380_ ratio (p<0.0001, RM one-way ANOVA + Tukey’s test).


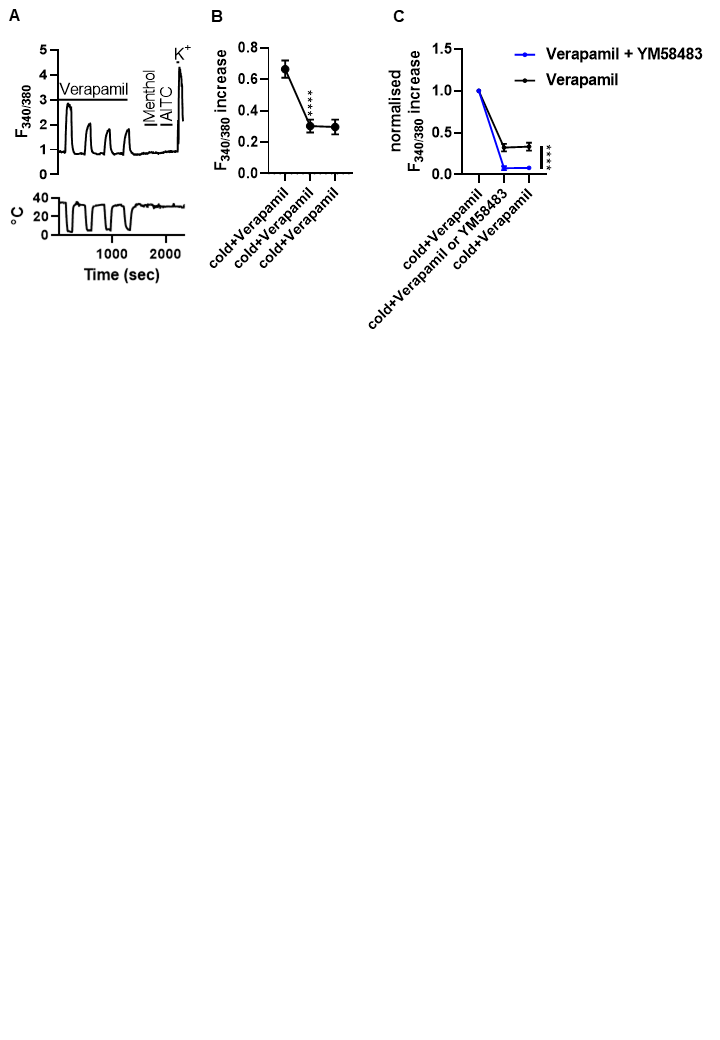


**Appendix Figure S4: ORAI blocker YM58483 inhibits the novel cold-evoked calcium increase in DRG neurons independently of tachyphylaxis.**

(**A**) Responses of novel cold-sensitive DRG neuron to four repeated cold stimuli show tachyphylaxis in the presence of L-type Ca_V_ antagonist verapamil (100µM). Temperature trace below. (**B**) Collected results (n = 76 cold-sensitive neurons). Significant decrease in cold-response amplitude between the first and second cold stimulus (p<0.0001, RM one-way ANOVA + Dunnett’s test). (**C**) Normalised responses of novel cold-sensitive DRG neurons to repeated cold stimuli in the presence of L-type Ca_V_ antagonist verapamil (100µM) alone (n=59), or in combination with Orai blocker YM58483 (3µM, n=28) applied during the second cold stimulus (p<0.0001, Two-Way ANOVA + Sidak’s test). Cold responses diminish because of tachyhylaxis but are only fully blocked when both verapamil and YM58483 are applied.


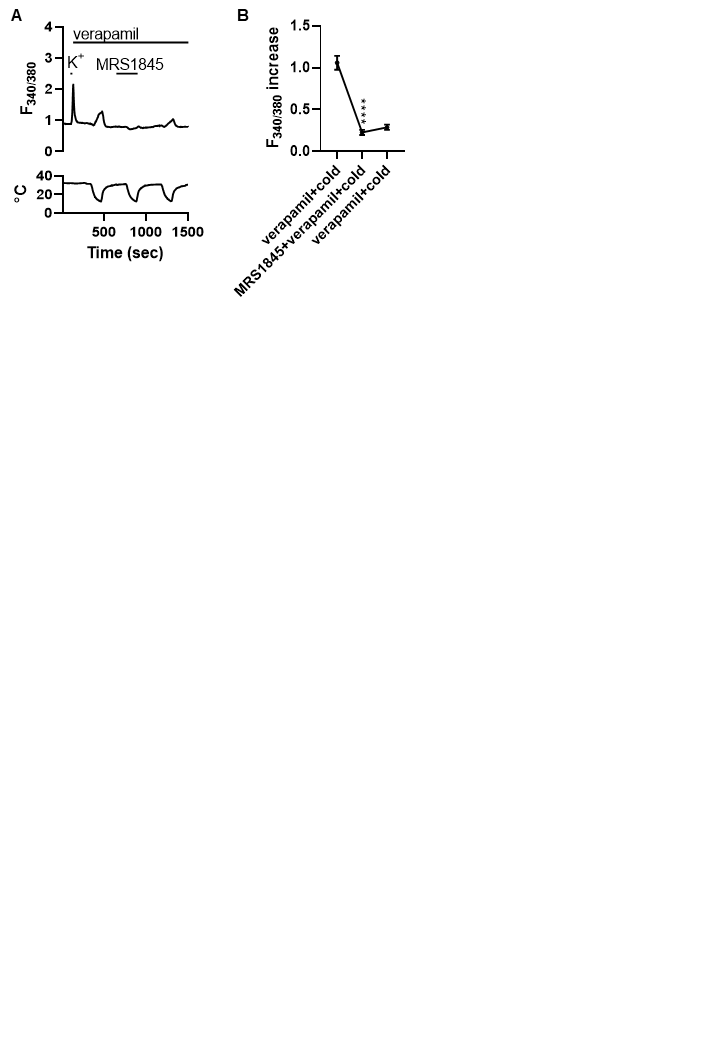


**Appendix FigureS5: The ORAI blocker MRS1845 blocks cold responses in the presence of Verapamil.**

(**A**) Representative Ca^2+^ imaging traces showing the effect of ORAI and CaV channel antagonist MRS1845 (30μM) on cold responses in an SCG neuron in the presence of L-type CaV antagonist Verapamil (100μM). (**B**) Collected results of similar experiments (n=201 SCG neurons). MRS1845 caused a significant decrease in cold-response amplitude (p<0.0001, RM one-way ANOVA + Dunnett’s test).


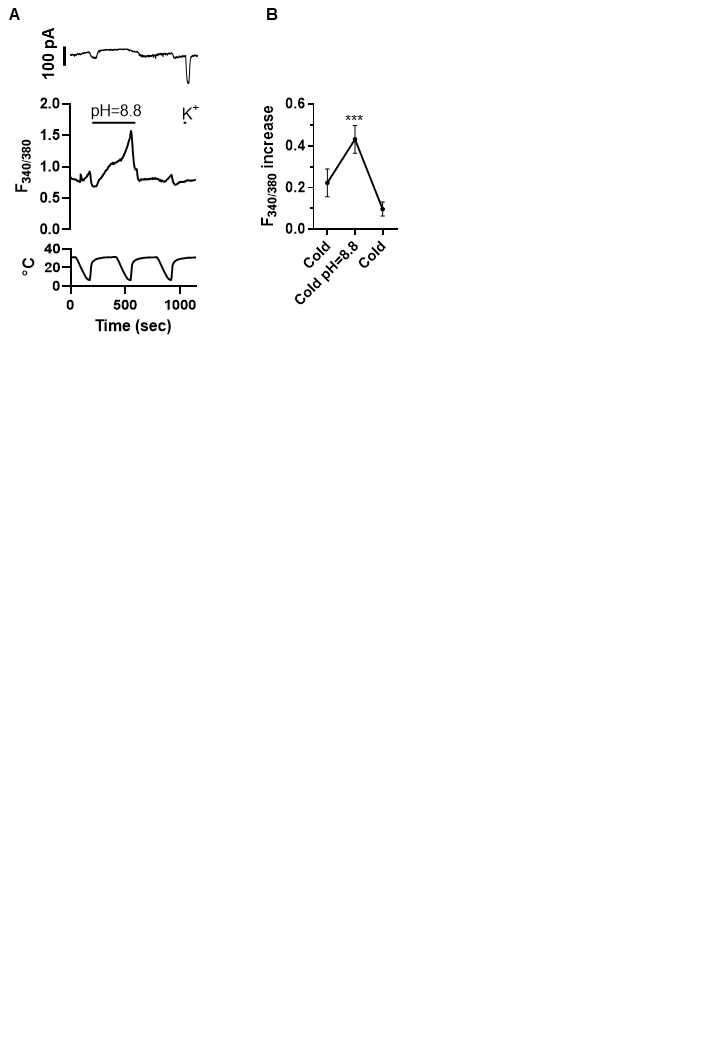


**Appendix Figure S6: Cold responses are potentiated by alkaline pH.**

(**A**) Concurrent voltage clamp and Ca^2+^ imaging of an SCG neuron exposed to cold ramp from 32°C to 6°C. Calcium increase is not associated with a detectable inward current. Top: current trace of SCG neuron voltage-clamped at -60mV. Middle: simultaneous Ca^2+^ imaging trace showing that cold increased Ca^2+^ influx is enhanced by alkaline pH. Bottom: temperature trace. (**B**) Cold response amplitudes of 9 SCG neurons that were voltage clamped at -60mV and exposed to pH 8.8. Alkaline pH caused a significant increase in cold response amplitude (p=0.0007, RM one-way ANOVA + Dunnett’s test).


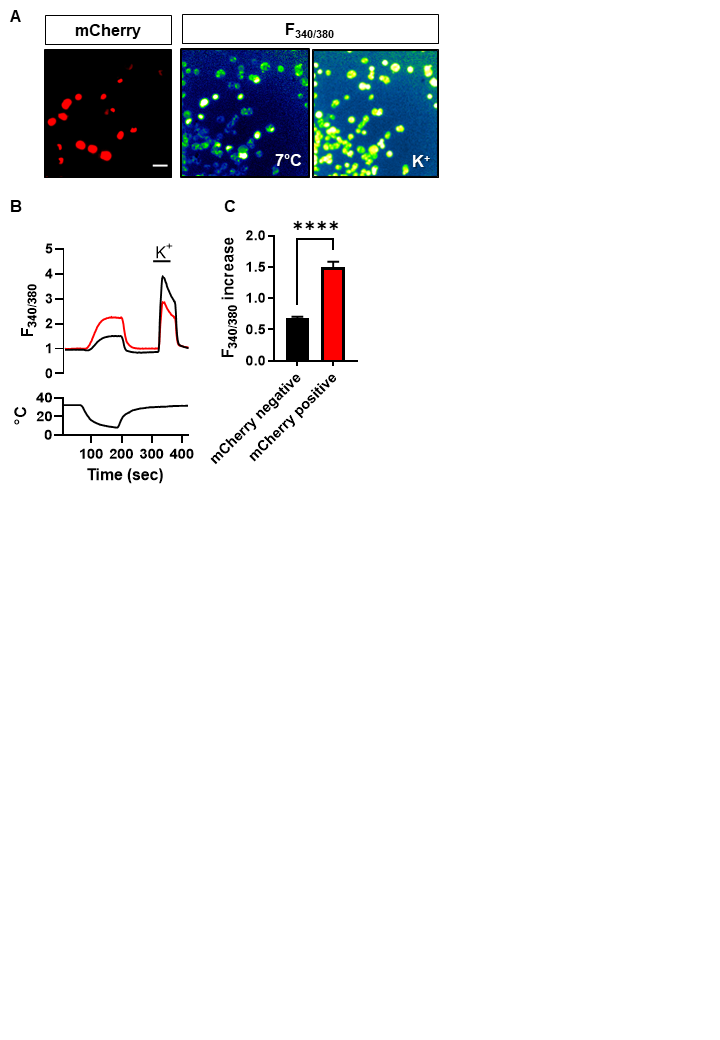


**Appendix Figure S7: Overexpression of STIM1 + ORAI1 increases cold-sensitivity of PC12 cells.**

(**A**) Representative fluorescence images of PC12 cells transfected with mCherry + STIM1 + ORAI1 during a cold stimulus and upon application of high K^+^ solution. Scale bar = 10μm.(**B**) Average traces showing cold-induced increase in F_340/380_ of PC12 cells transfected with mCherry+STIM1+ORAI1. Black trace: cells not expressing mCherry (64% of total). Red trace: cells expressing mCherry (36% of cells). Temperature trace below. (**C**) Bar chart summarising results of 765 PC12 cells on 7 coverslips (mean ± SEM). “mCherry negative” corresponds to black trace in **B** (n=491 cells) and “mCherry positive” to red trace in **B** (n=274 cells). Difference significant (unpaired t-test, P<0.0001).


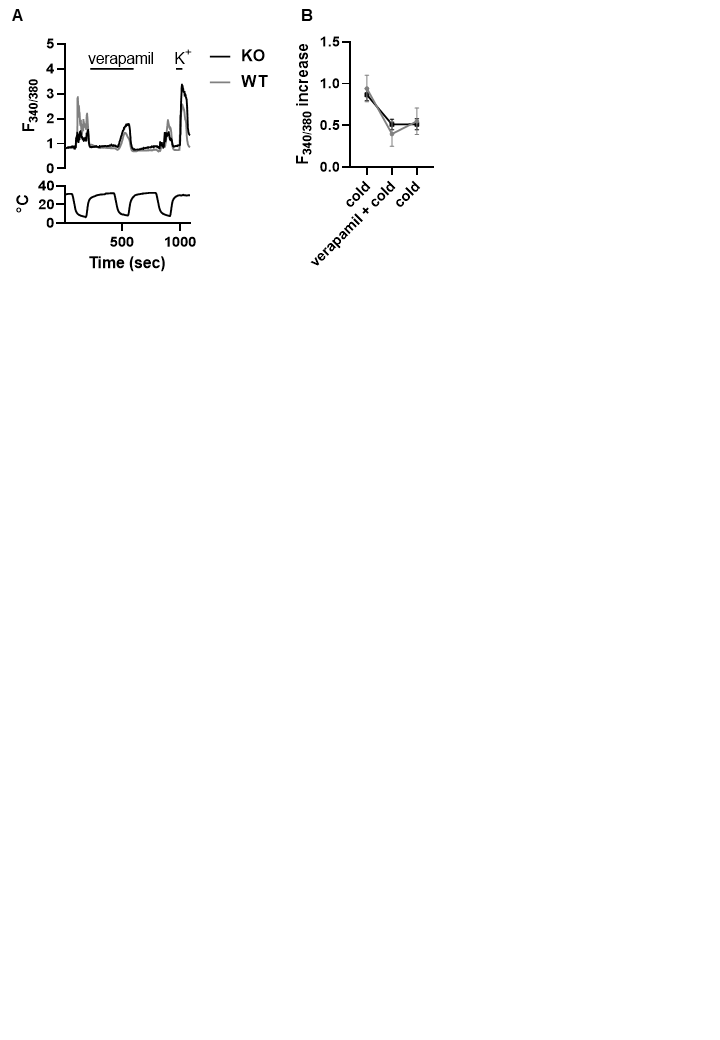


**Appendix Figure S8: Knockout of ORAI3 has no effect on cold responses of SCG neurons.**

(**A**) Representative Ca^2+^ imaging responses to cold of wildtype (grey) and ORAI3 Knockout neurons (black) from littermates. Temperature trace shown below. (**B**) Mean cold response amplitudes (mean ± SEM) of 30 WT and 119 KO neurons imaged on two separate days. No significant difference between groups (p=0.84, RM one-way ANOVA + Dunnett’s test).


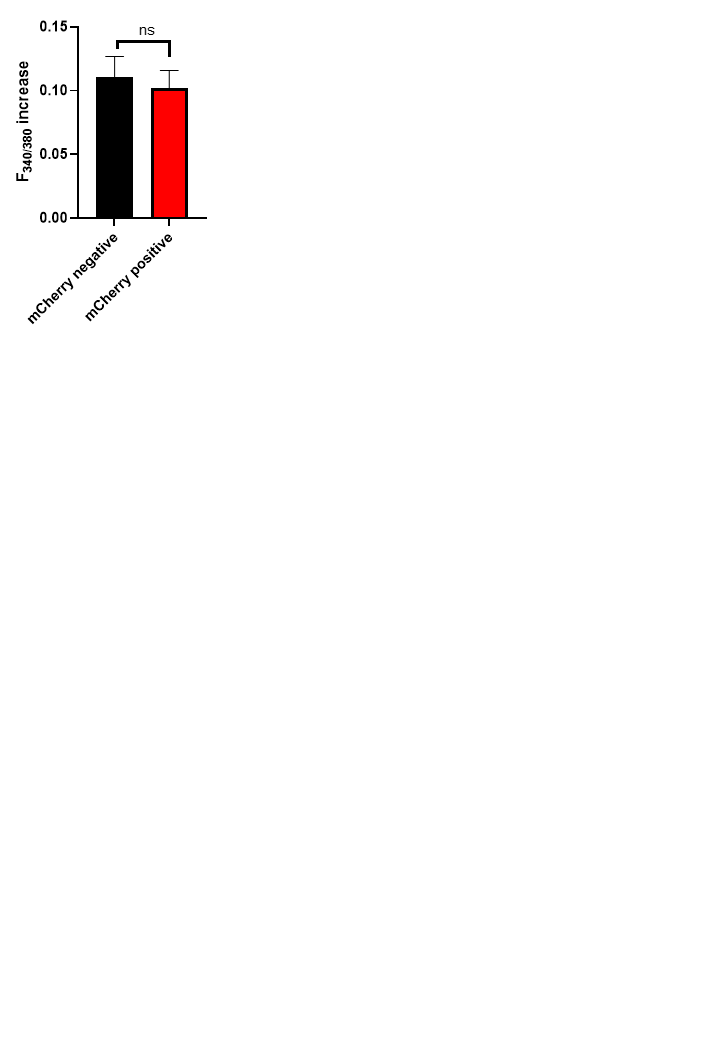


**Appendix Figure S9: Non-targeting siRNA does not affect responses of SCG neurons to cold.**

Values for increase in fura-2 fluorescence ratio (ΔF_340/380_) in response to a drop in temperature from 32°C to 6°C in experiments similar to those shown in **Fig. 4D – G** were not different between mCherry positive SCG neurons, transfected with the non-targeting siRNA (red bar, n=41) and neurons not transfected with siRNA (black bar, p>0.05, n=63). Responses to cold in all neurons were significantly above baseline (p< 0.0001 for both groups, unpaired t-test).


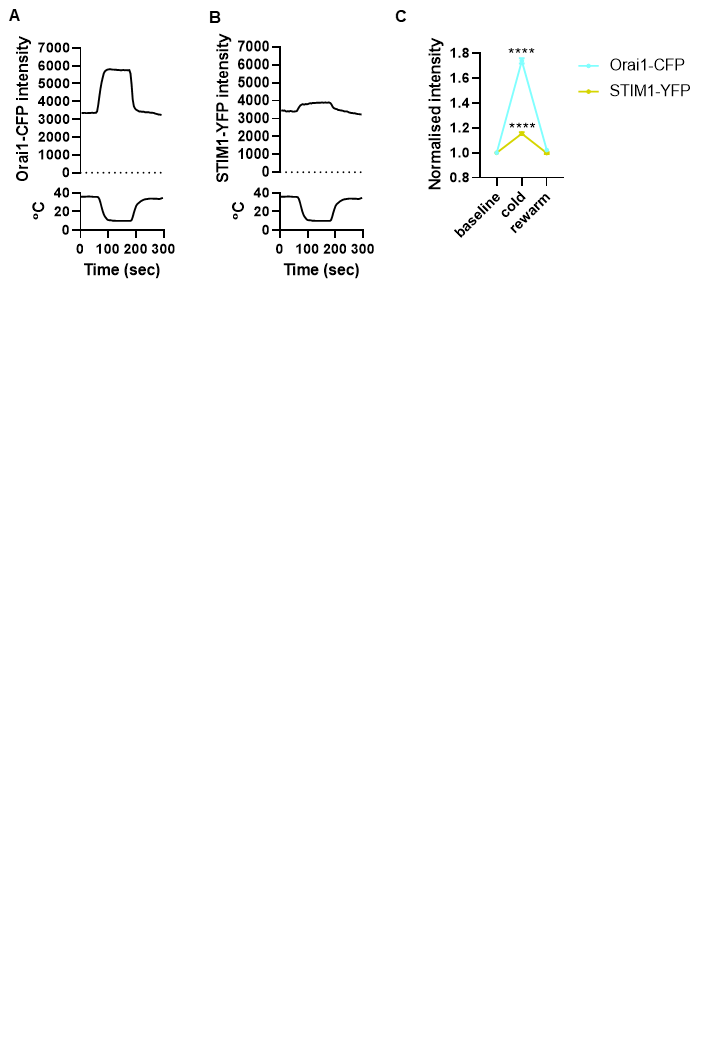


**Appendix Figure S10: ORAI1-CFP and STIM1-YFP fluorescence is directly affected by cold.**

(**A**) Effect of cooling on fluorescence intensity of ORAI1-CFP expressed in HEK293 cell (no transfected STIM1). Temperature trace below. (**B**) Similar experiment on STIM1-YFP (no transfected ORAI1). (**C**) Normalised mean ± SEM of ORAI1-CFP and STIM1-YFP intensity before, during, and after a cold stimulus (total cell fluorescence measured, n=64, 55 cells, respectively, p<0.0001, 2 way ANOVA + Dunnett's test).
