# Supplementary material for: STIM1 and ORAI1 form a novel cold transduction mechanism in sensory and sympathetic neurons
Source: EMBO J. 2022 Dec 16;42(3):e111348. doi: 10.15252/embj.2022111348 (PMC9890232; doi:10.15252/embj.2022111348)
Supplement: Supplementary file 2 — Expanded View Figures PDF [file EMBJ-42-e111348-s008.pdf]

Expanded View Figures

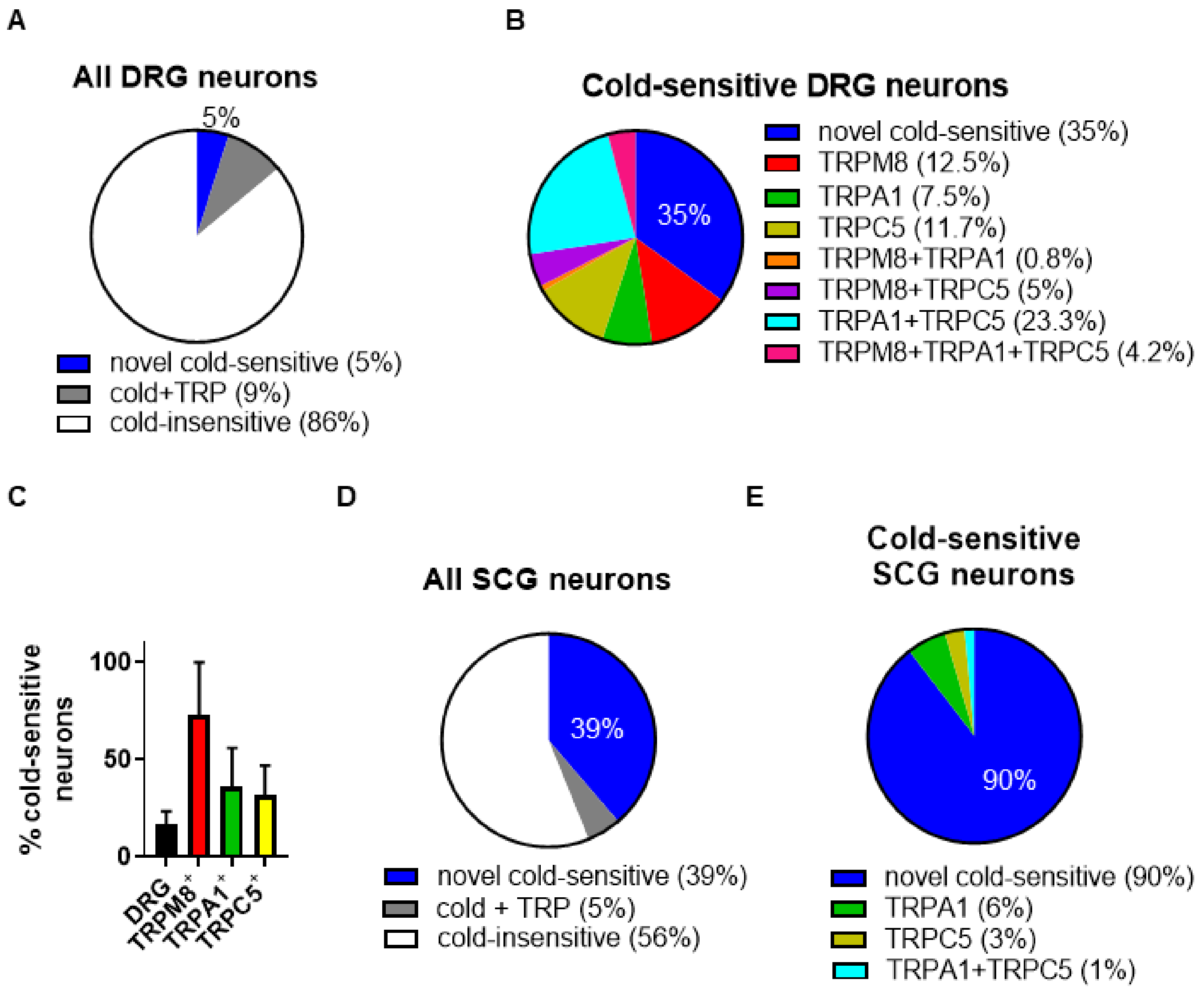

Figure EV1. Expression of TRPM8 is more highly correlated with cold-sensitivity than expression of TRPA1 or TRPC5.

A Percentage of DRG neurons that were both cold-sensitive and expressed one or more of TRPM8, TRPA1, or TRPC5 (grey), and novel cold-sensitive DRG neurons that did not express any of these TRP channels (blue). Protocol as in Fig 1A and E, $n = 859$ neurons imaged on three separate days. 5% of DRG neurons expressed only a novel cold-sensitive mechanism (blue).

B Different populations of cold-sensitive neurons by expression of TRPM8, TRPA1, and TRPC5. $n = 99$ cold-sensitive neurons selected from the experiment in A. 35% of cold-sensitive neurons did not express any of TRPM8, TRPA1 or TRPC5 (blue).

C Average percentage of cold-sensitive DRG neurons as follows: out of all neurons (black); out of the subpopulation of DRG neurons expressing TRPM8 but not TRPA1 or TRPC5 (red); out of the subpopulation of DRG neurons expressing TRPA1, but not TRPM8 or TRPC5 (green); and out of the subpopulation of DRG neurons expressing TRPC5, but not TRPM8 or TRPA1 (yellow, all mean ± SEM).

D Percentage of total SCG neurons that were both cold-sensitive and expressed either TRPA1 or TRPC5 (grey); and novel cold-sensitive SCG neurons that did not express any of the TRP channels (blue). No neuron responded to the TRPM8 agonist menthol ($n = 155$ neurons, 3 days). 39% of SCG neurons expressed a novel cold-sensitive mechanism.

E Different populations of cold-sensitive SCG neurons by expression of TRPA1 and TRPC5. $n = 67$ neurons taken from (D). 90% of cold-sensitive neurons did not express any of TRPM8, TRPA1, or TRPC5 (blue).

Source data are available online for this figure.

 © 2022 The Authors

Figure EV2. Effect of Ca_V channel antagonists on cold-induced Ca^{2+} influx in SCG neurons.

A Cd^{2+} (100 μM) which blocks Ca_V and ORAI channels but activates TRPA1, suppresses cold-induced Ca^{2+} influx in SCG neurons. Temperature trace below.
B Collected results with Cd^{2+} ($P < 0.0001$, RM one-way ANOVA + Dunnett's test, $n = 60$ neurons on 8 coverslips). Cooling SCG neurons from 34°C to 6°C in the presence of Cd^{2+} reversibly decreased cold-response amplitudes by 87%.
C Gd^{3+} (1 μM), which blocks Ca_V and ORAI channels, but activates TRPC5, suppresses cold-induced Ca^{2+} influx in SCG neurons.
D Collected results with Gd^{3+} ($P < 0.0001$, RM one-way ANOVA + Dunnett's test, $n = 78$ neurons, 12 coverslips). Cooling SCG neurons from 35°C to 5°C in the presence of Gd^{3+} reversibly decreased cold-response amplitudes by 82%.
E Effect of L-type Ca_V channel blocker Verapamil (100 μM). Verapamil had opposing effects on different neurons, increasing some responses (blue trace), and decreasing other responses (black trace). There were also many neurons with intermediate levels of either suppression or enhancement.
F Collected results with verapamil ($P < 0.0001$, RM one-way ANOVA + Dunnett's test, $n = 277$ neurons, 4 coverslips). 13% of cold responses were fully blocked by Verapamil, as shown in (E) (black trace).
G Non-selective Ca_V channel blocker Mibefradil (10 μM) enhanced some responses (blue) and suppressed others (black).
H No overall effect on $F_{340/380}$ (114 cold-sensitive neurons).
I Effect of L-type Ca_V channel blocker Nifedipine (10 μM) on cold-induced Ca^{2+} influx in SCG neurons. Nifedipine enhanced cold-induced Ca^{2+} influx in some neurons (blue trace) but suppressed it in others (black).
J Normalized results of 18 cold-sensitive neurons exposed to nifedipine. No overall significant difference in cold response amplitude.
K Effect of non-selective Ca_V channel blocker Bepridil (10 μM).
L Normalized results from 45 cold-sensitive neurons exposed to bepridil. No overall significant difference in cold response amplitude. All error bars mean ± SEM.

Source data are available online for this figure.

© 2022 The Authors

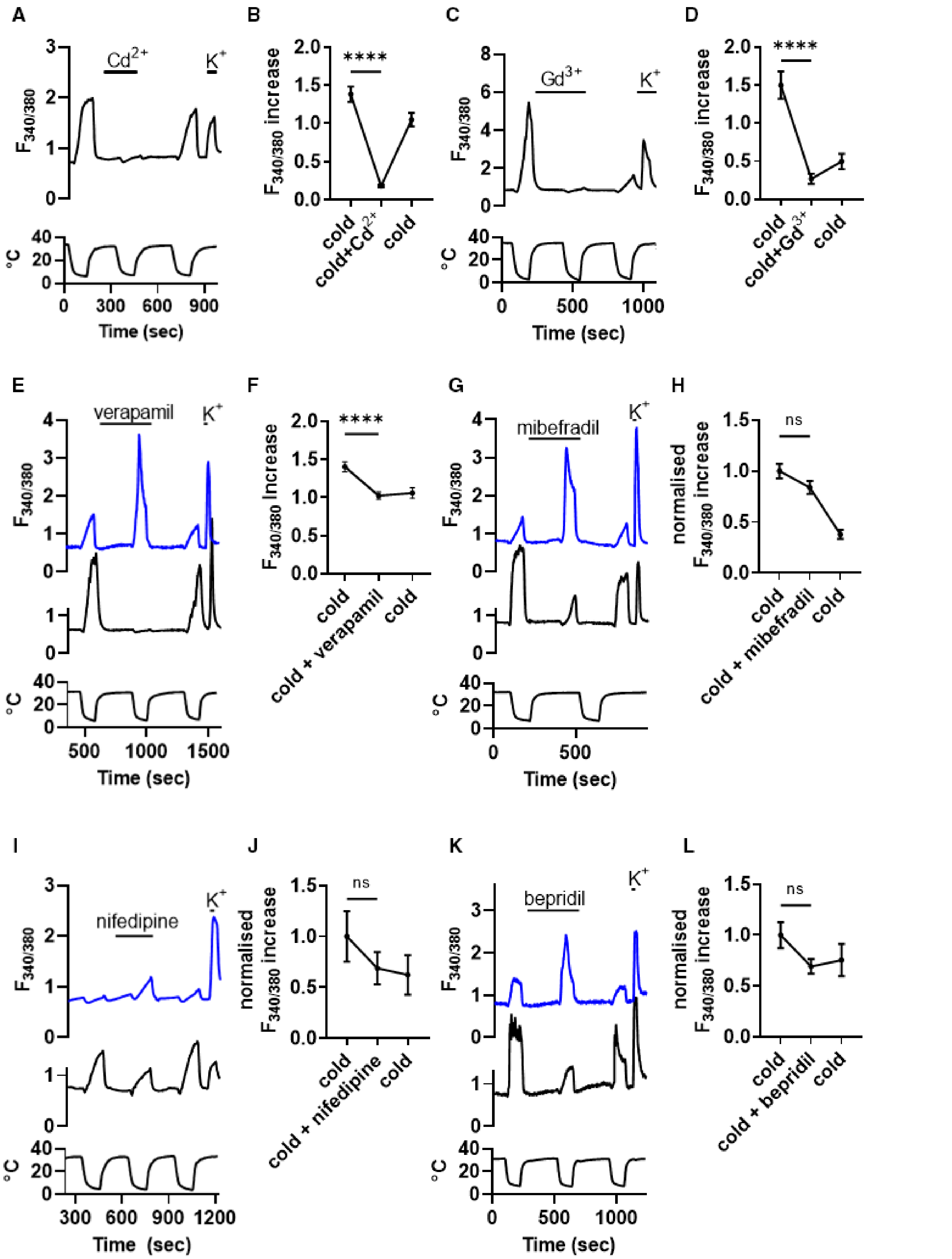

Figure EV2.

 © 2022 The Authors

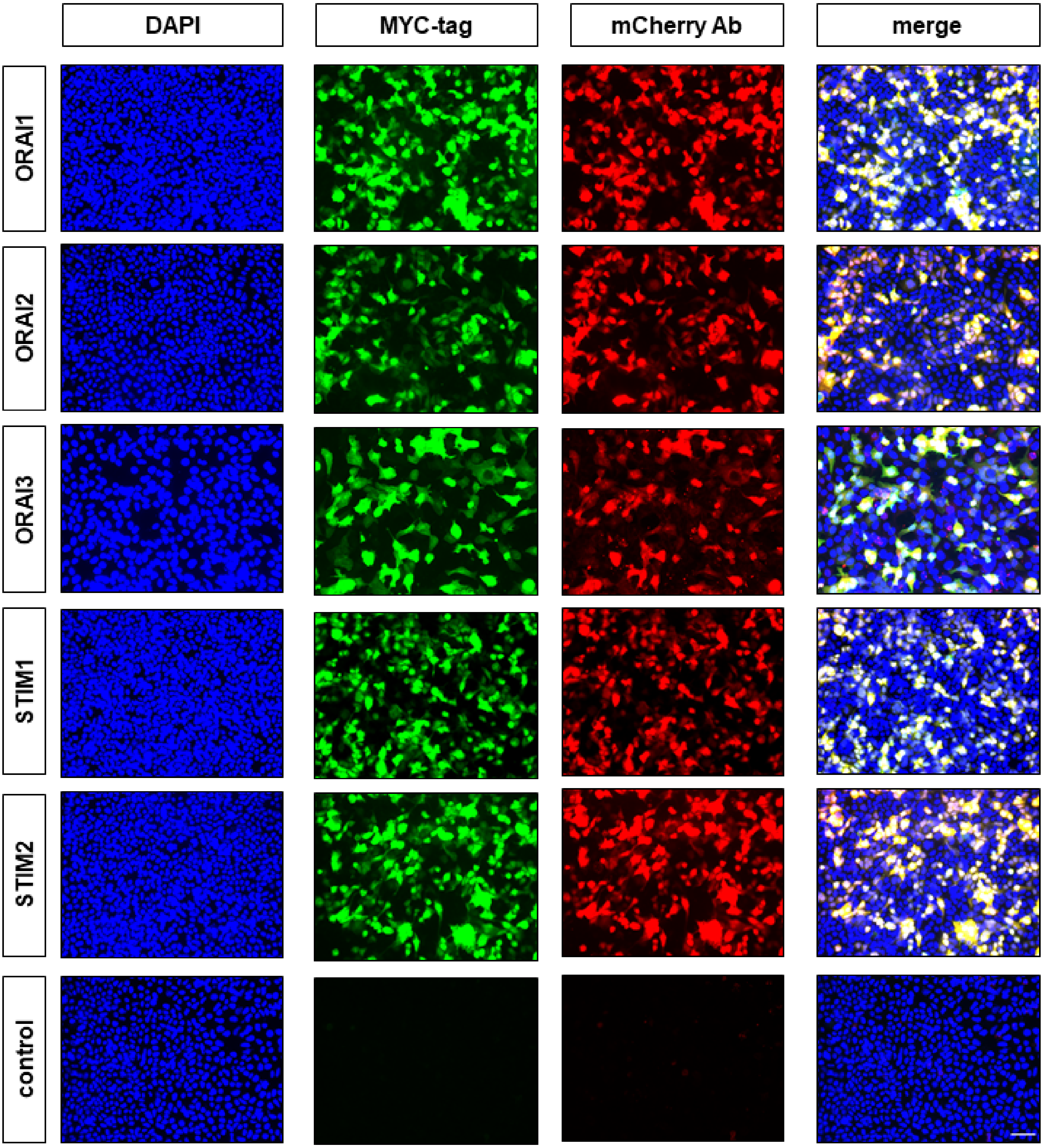

Figure EV3. Presence of mCherry reliably indicates successful overexpression of all STIM and ORAI plasmids.

Immunocytochemistry fluorescence images showing HEK293 cells transfected with a combination of mCherry and one of the MYC-tagged plasmids for STIM or ORAI proteins (indicated at left). Control cells (bottom) underwent same protocol without DNA. Cells were stained with nuclear marker DAPI and antibodies against mCherry and MYC. Not all cells were successfully transfected but all those cells expressing mCherry also express the cotransfected STIM/ORAI proteins, as shown by the merged images (right). $n = 4$ cultures each. Scale bar = 50 μm.

Source data are available online for this figure.

© 2022 The Authors

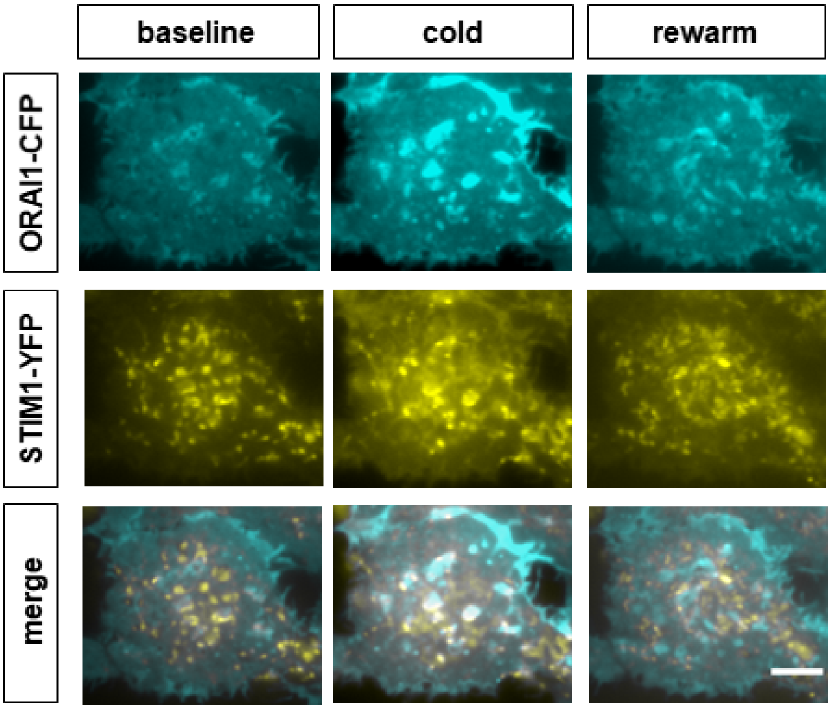

Figure EV4. The effect of cold on ORAI1 puncta formation and colocalization of ORAI1 and STIM1 in a HEK293 cell.

Similar to Fig 5A but with a larger number of visible STIM1 and ORAI1 puncta. Scale bar = 5 μm.

Source data are available online for this figure.

 © 2022 The Authors

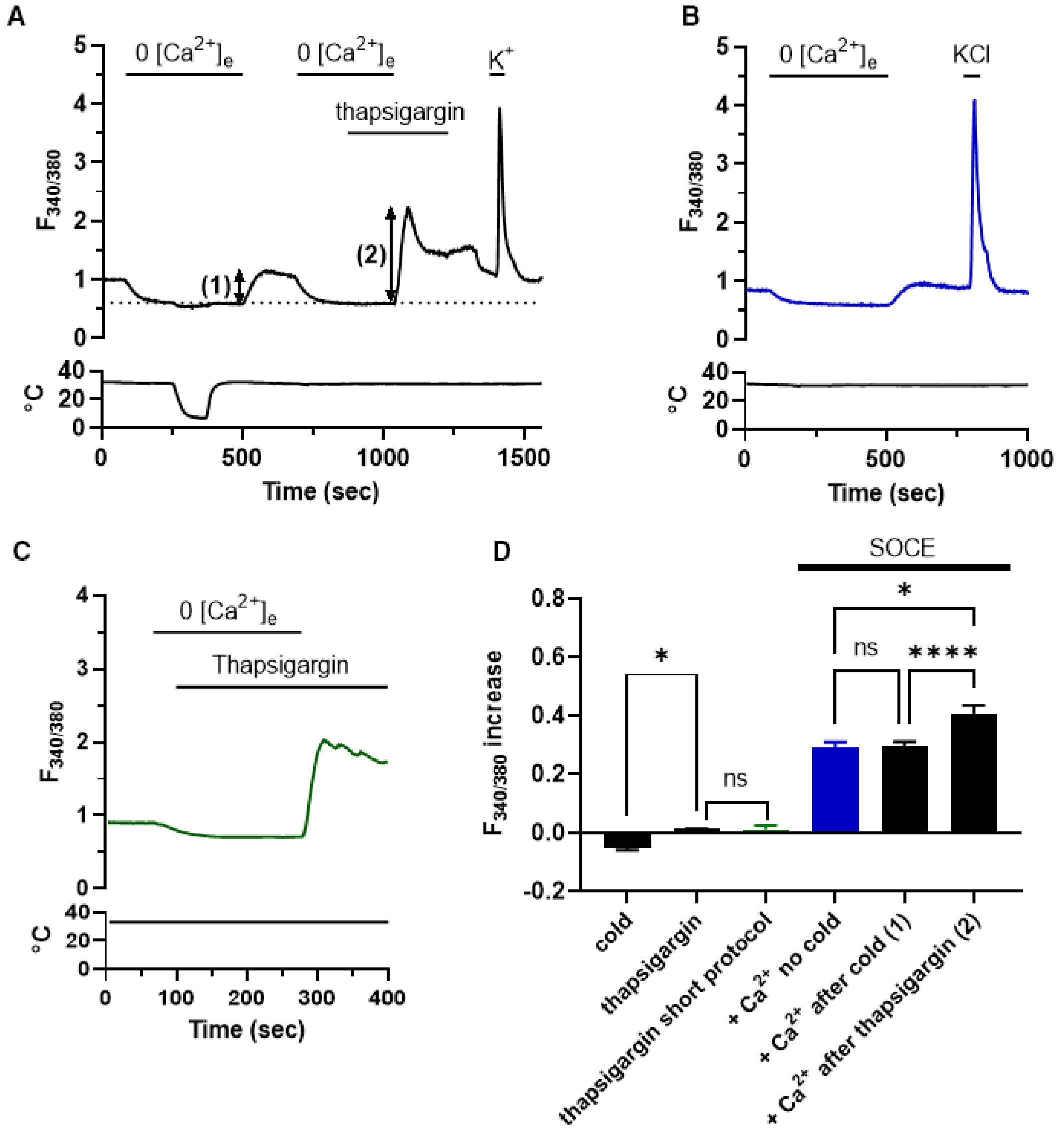

Figure EV5. Cold does not cause discharge of calcium from subcellular stores.

A, B Representative Ca^{2+} imaging trace showing responses of an SCG neuron to cold and to 10 μM thapsigargin, both in the absence of extracellular Ca^{2+}. There was no detectable increase in intracellular calcium caused by discharge of stores in either case (the small negative deflection during application of cold is an artefact caused by the effect of cold on fura-2, see Appendix Fig S2). However on readmission of extracellular calcium the prior discharge of store calcium by thapsigargin is indicated by a significant store-operated calcium entry (SOCE, arrow (2)), while following application of a cold stimulus, SOCE induced by the re-admission of extracellular Ca^{2+} (arrow (1)) is identical to that observed with no cold stimulus - see (B).

C Thapsigargin does not cause a detectable calcium increase even when applied very soon (30 s) after transfer to zero Ca^{2+}.

D Bar chart summarizing response amplitudes (mean ± SEM) with protocol shown in (A) (n = 169) or (B) (n = 26) or (C) (n = 34). No difference in SOCE with or without cold application (ns, $P > 0.05$) while SOCE following thapsigargin is significantly larger than that following cold application ($P < 0.0001$, RM one-way ANOVA + Tukey's test).

Source data are available online for this figure.

© 2022 The Authors
