# Supplementary material for: STIM1 and ORAI1 form a novel cold transduction mechanism in sensory and sympathetic neurons
Source: EMBO J. 2022 Dec 16;42(3):e111348. doi: 10.15252/embj.2022111348 (PMC9890232; doi:10.15252/embj.2022111348)
Supplement: Supplementary file 3 — Movie EV1 [file EMBJ-42-e111348-s005.zip › Movie EV1/Legend Movie EV1.docx]

**Movie EV1: Video of movement of STIM1 puncta.**

Left-hand video: In normal extracellular calcium, STIM1 is visible in endoplasmic reticulum as diffuse fluorescence and as long streaks, but in addition some is visible as preformed puncta. Both streaks and puncta move continuously during the recording.

Right-hand video: In nominal zero calcium STIM1 is largely aggregated into preformed puncta that are to some extent mobile.

Both images of STIM1-YFP obtained with TIRF microscopy, nominal imaging depth 100nm. Real-time duration of both videos 230s.
