# Supplementary figures and images for: STIM1 and ORAI1 form a novel cold transduction mechanism in sensory and sympathetic neurons
Source: EMBO J. 2022 Dec 16;42(3):e111348. doi: 10.15252/embj.2022111348 (PMC9890232; doi:10.15252/embj.2022111348)

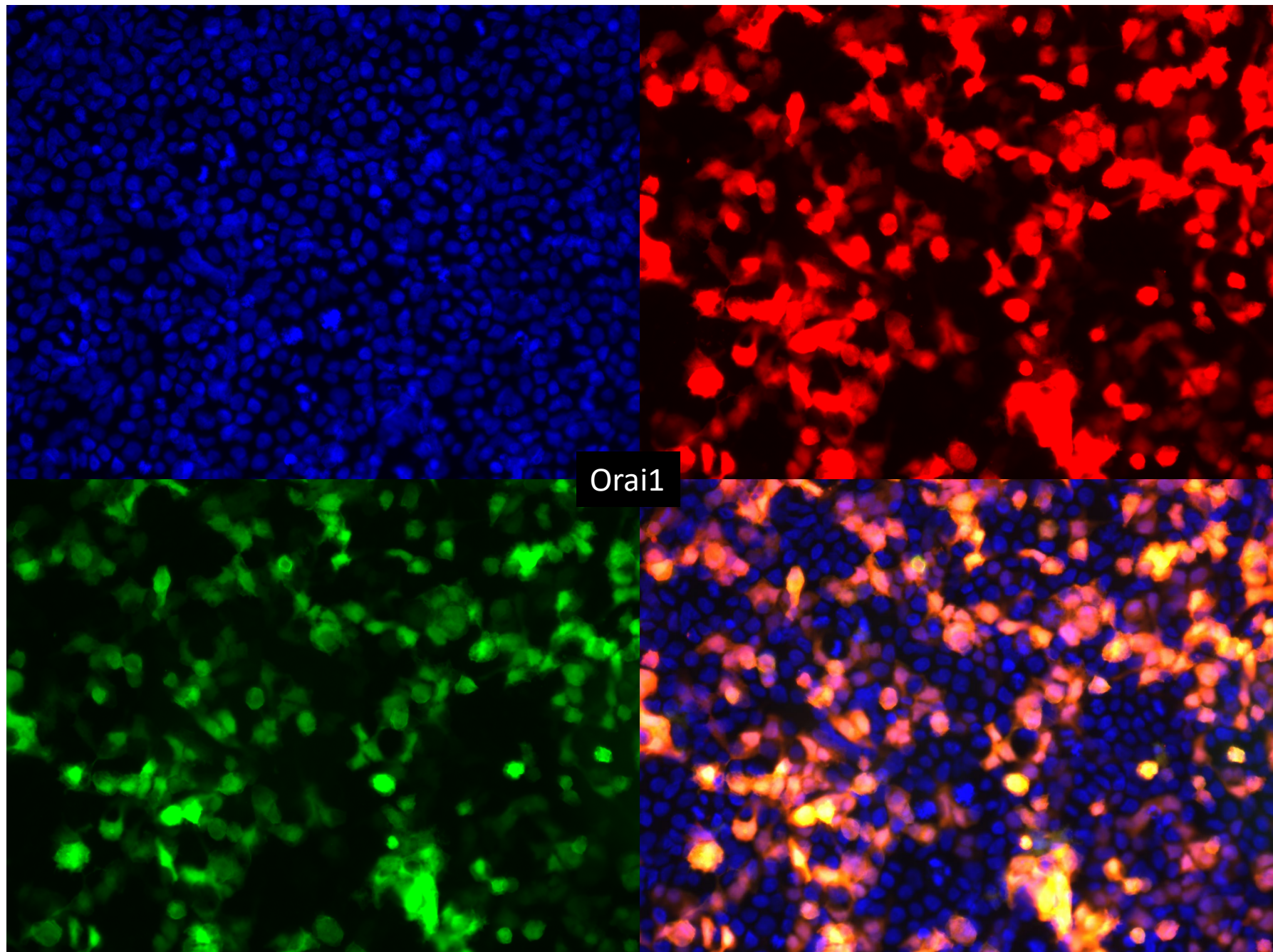

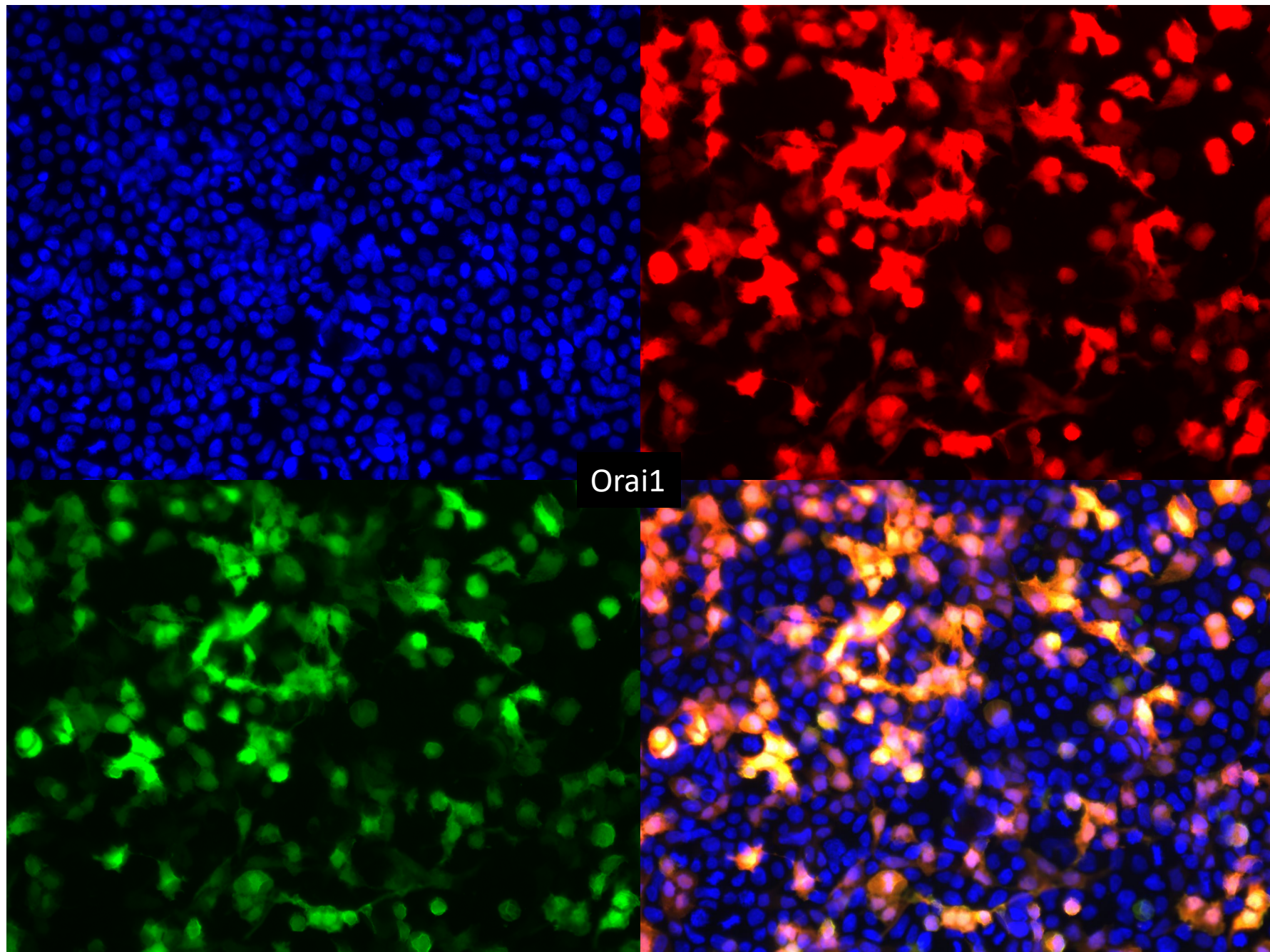

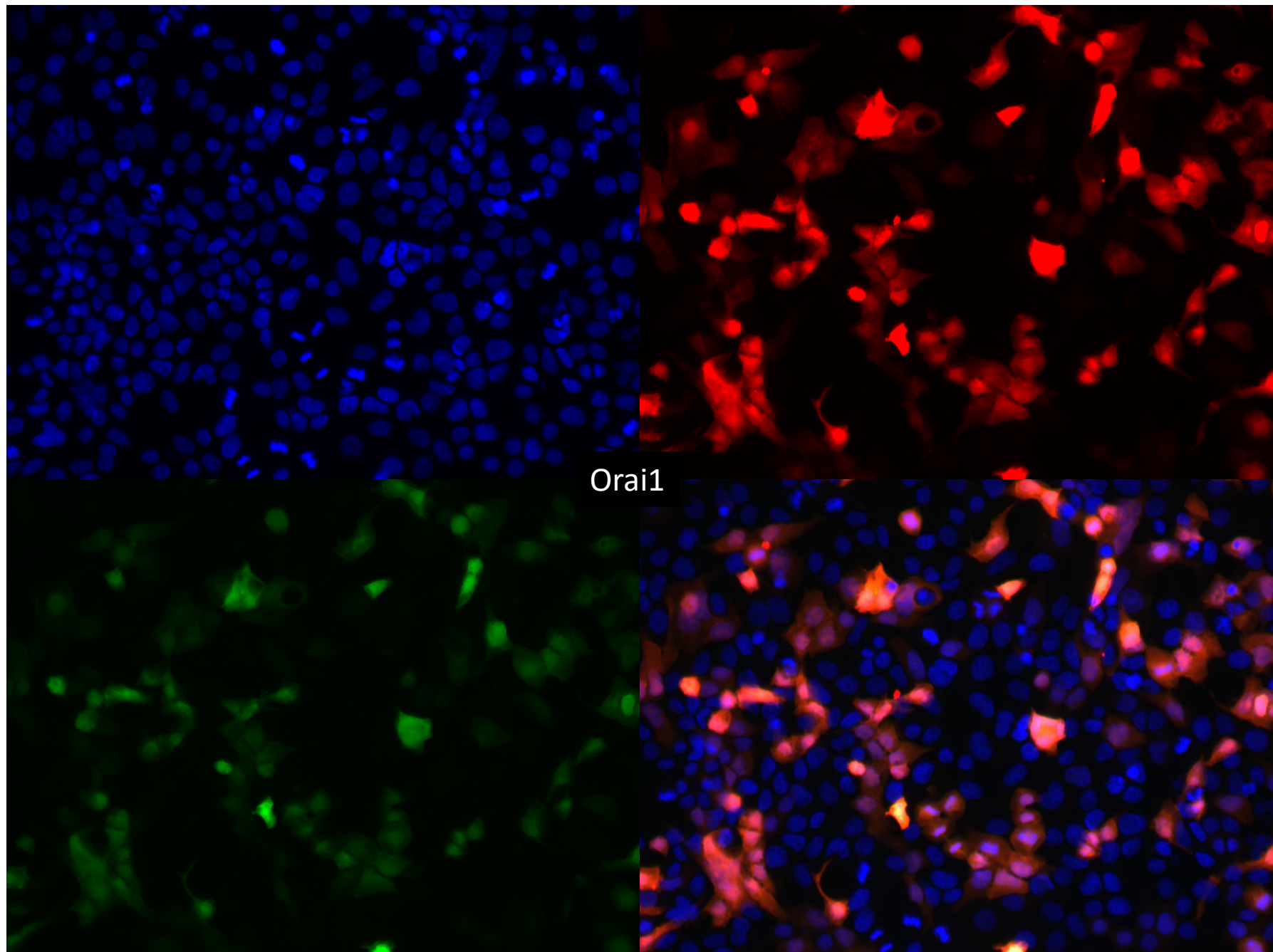

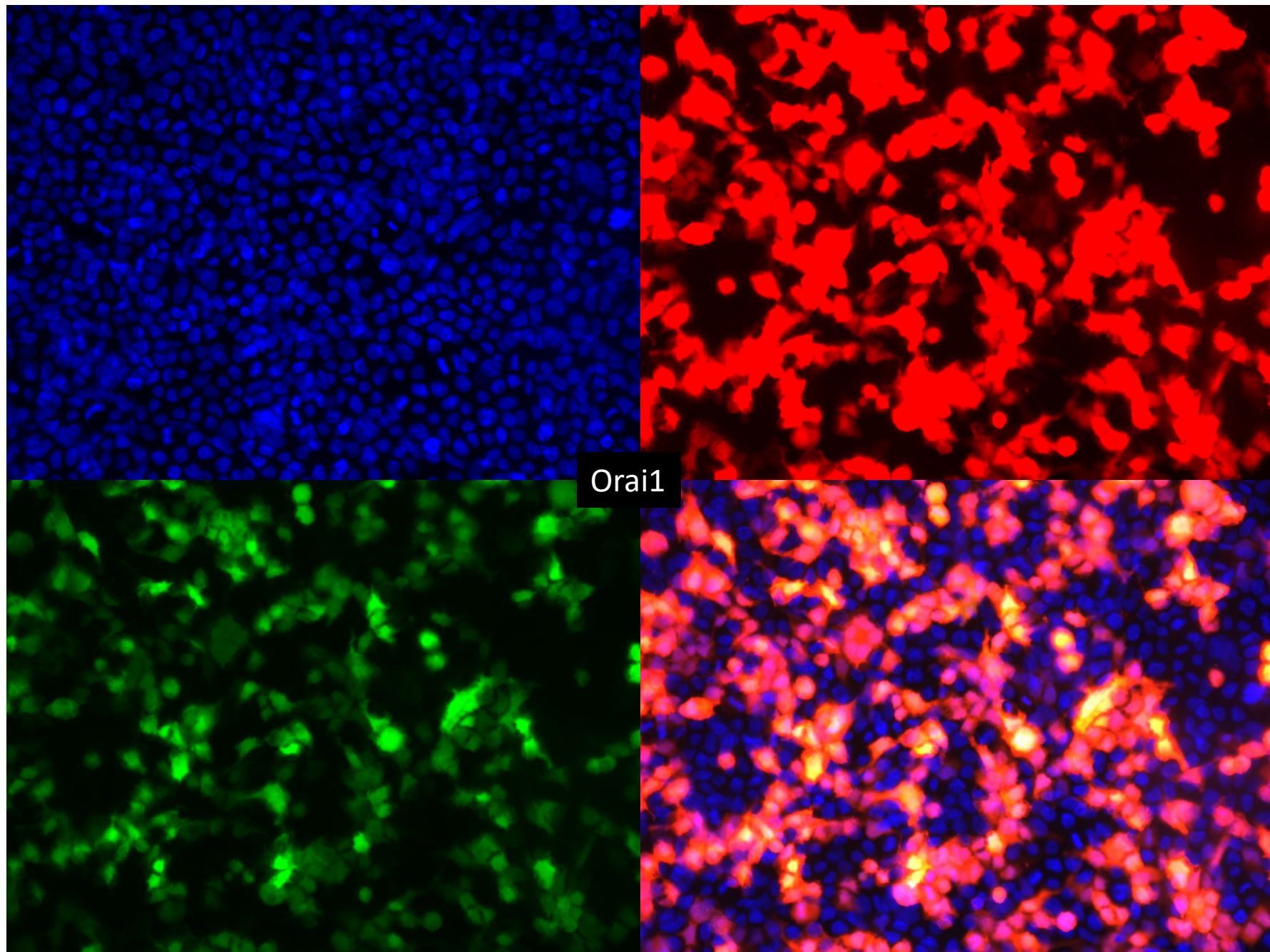

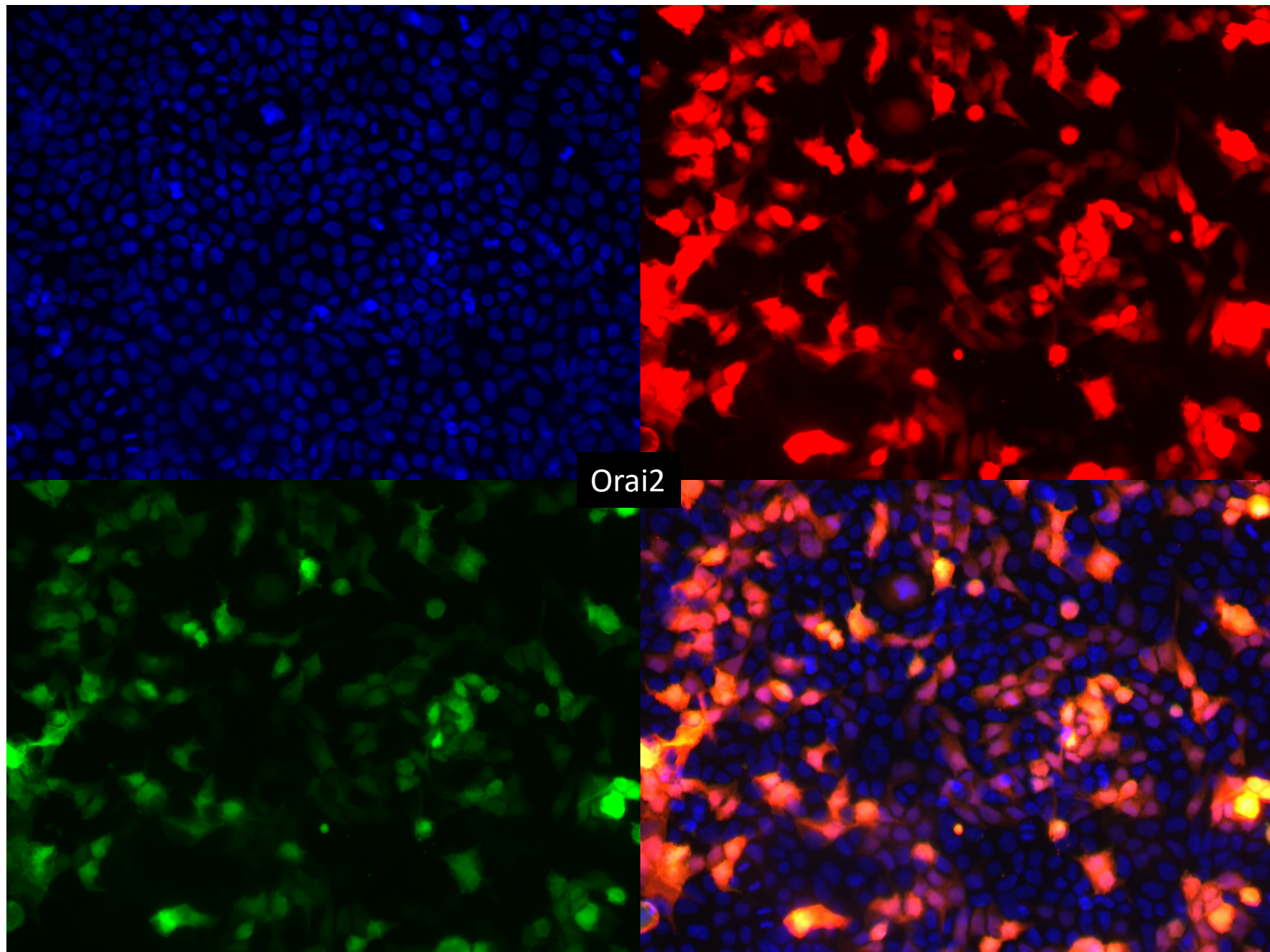

Orai2

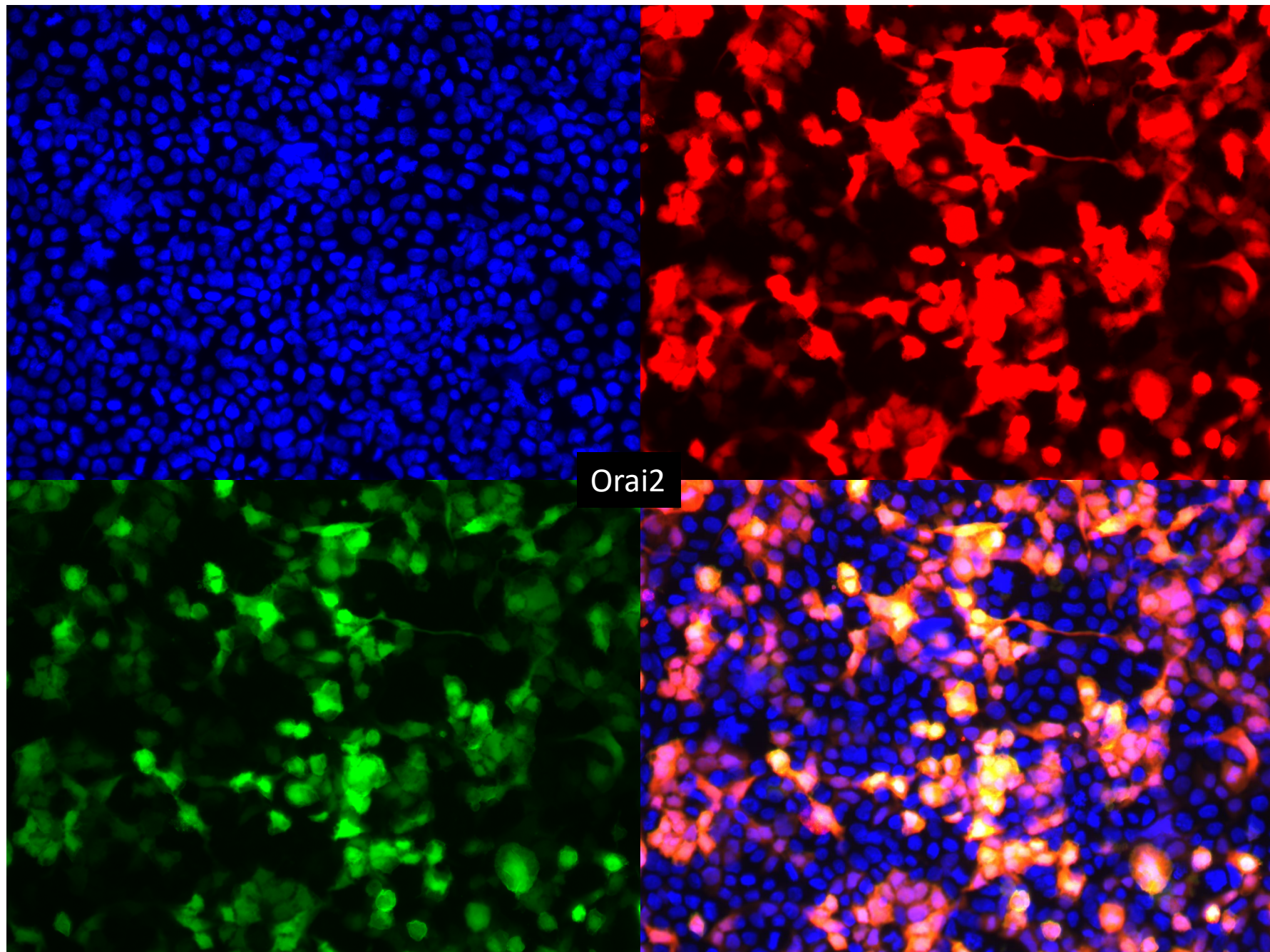

Orai2

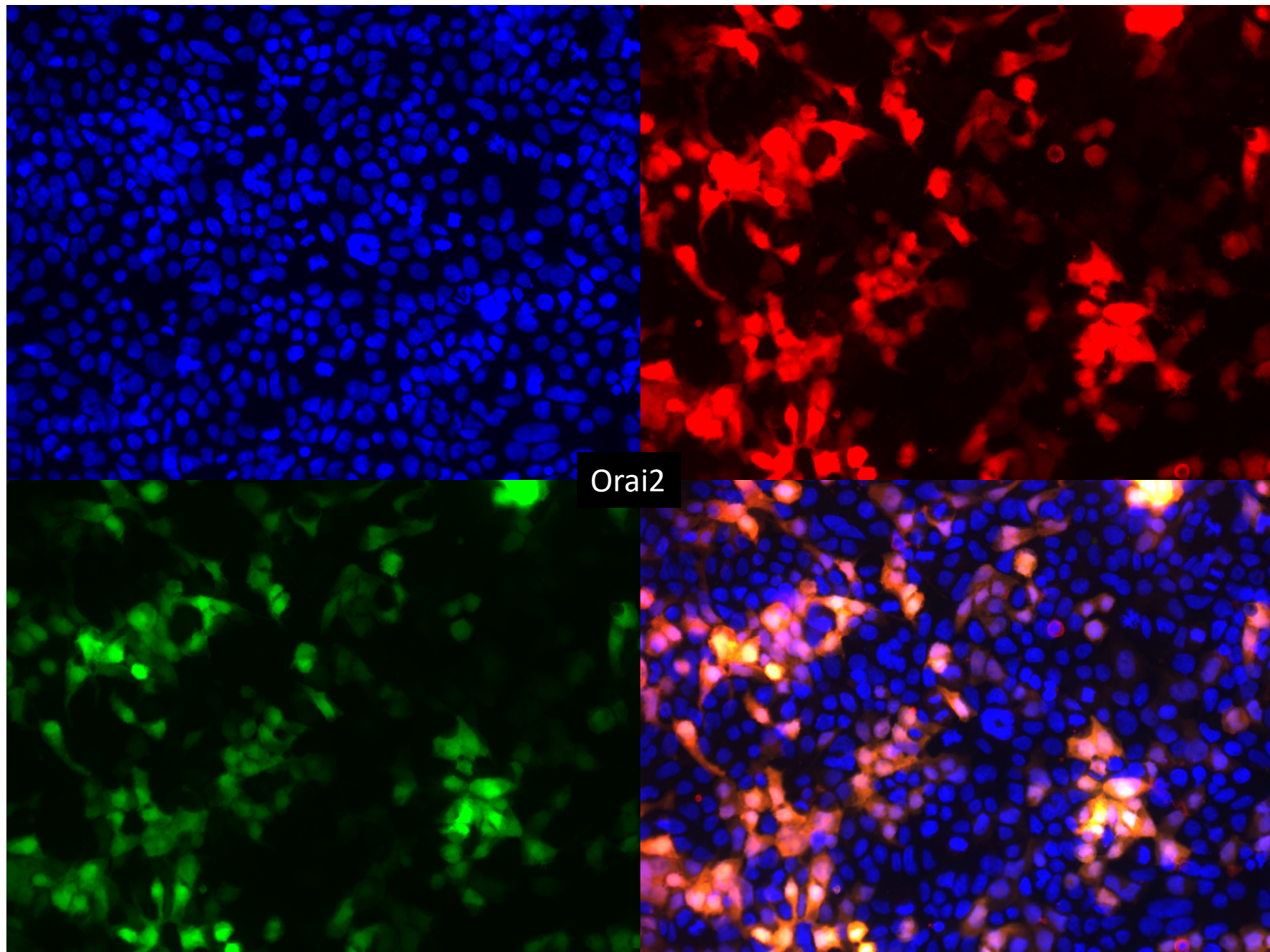

Orai2

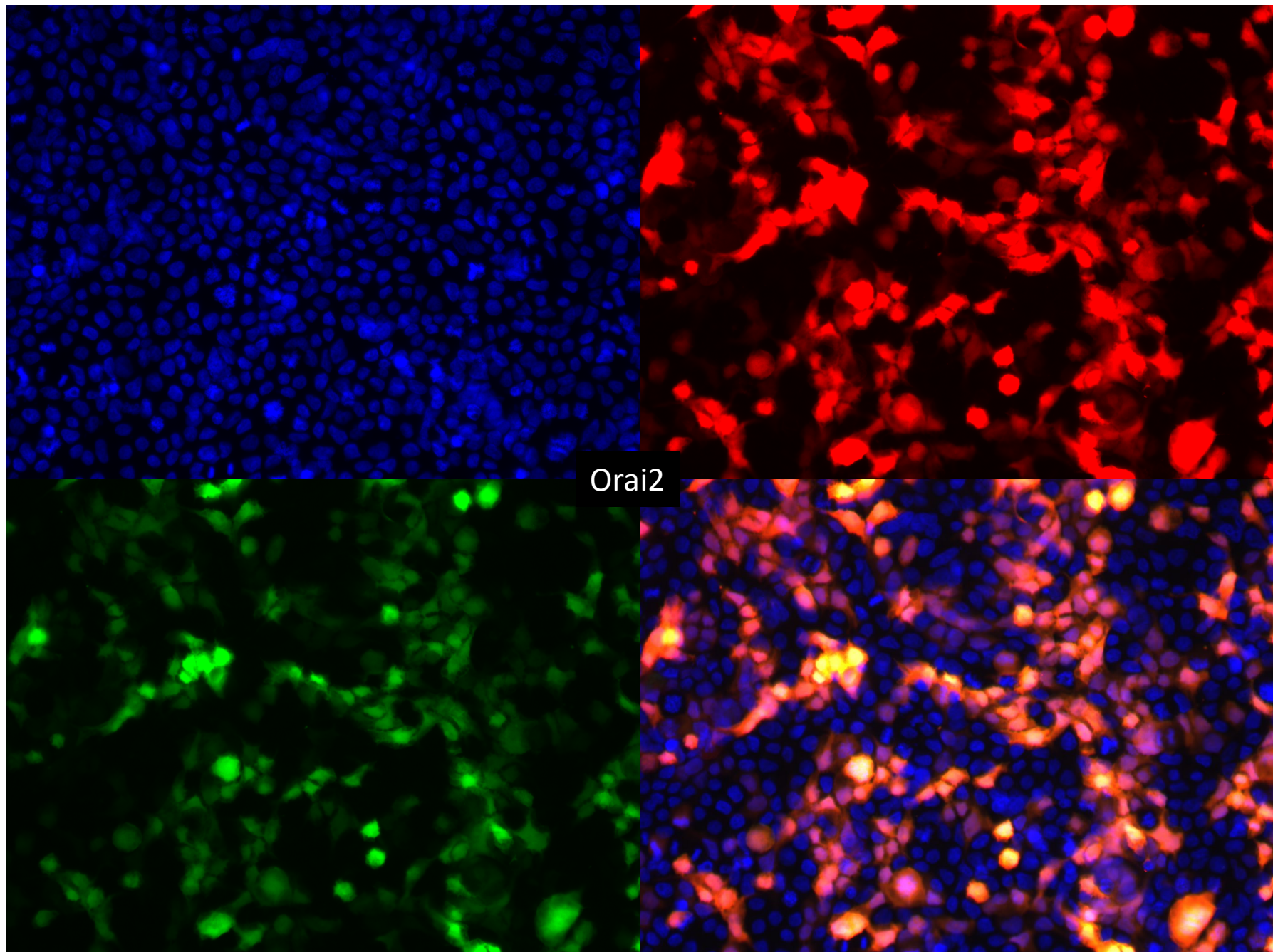

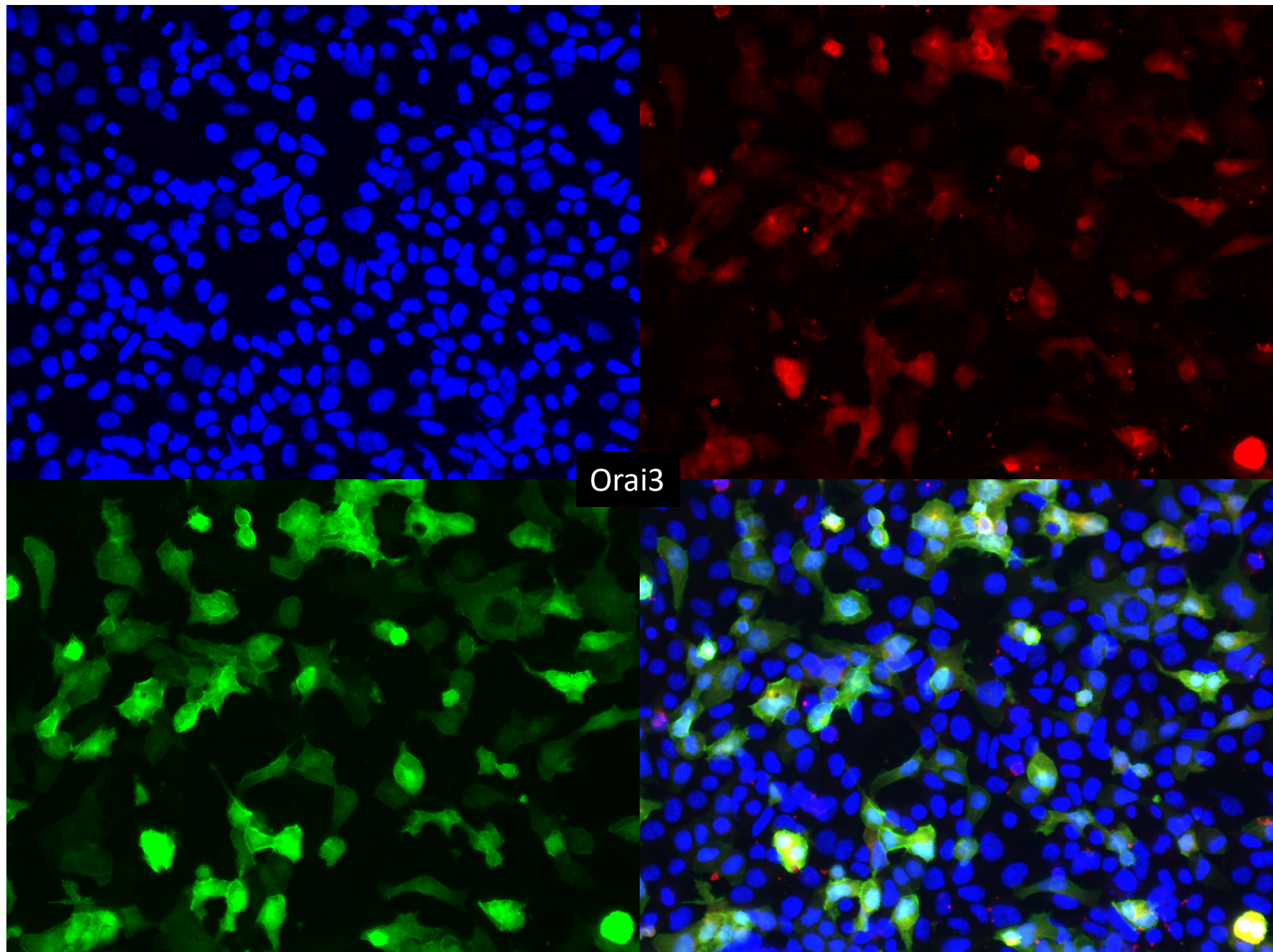

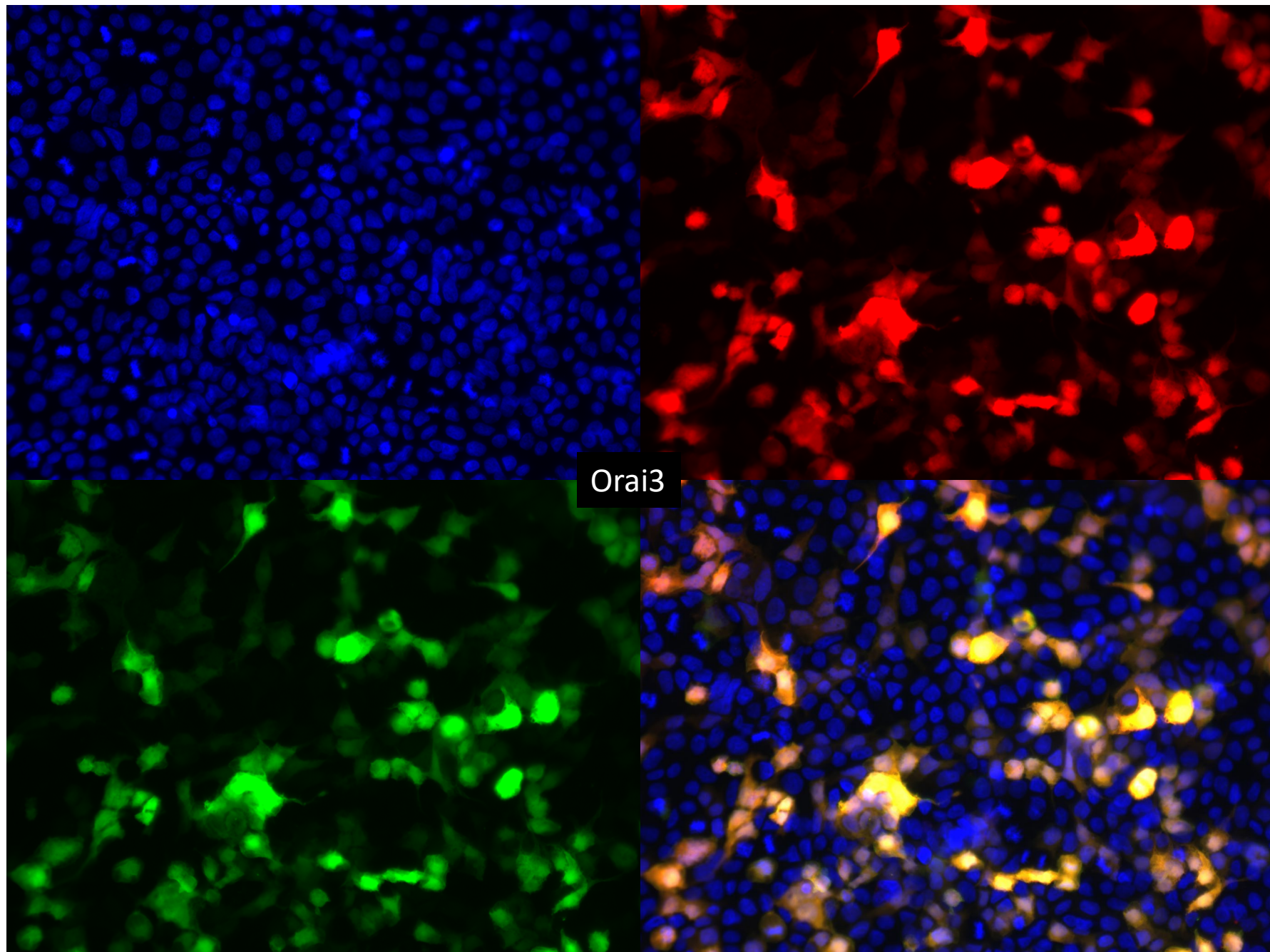

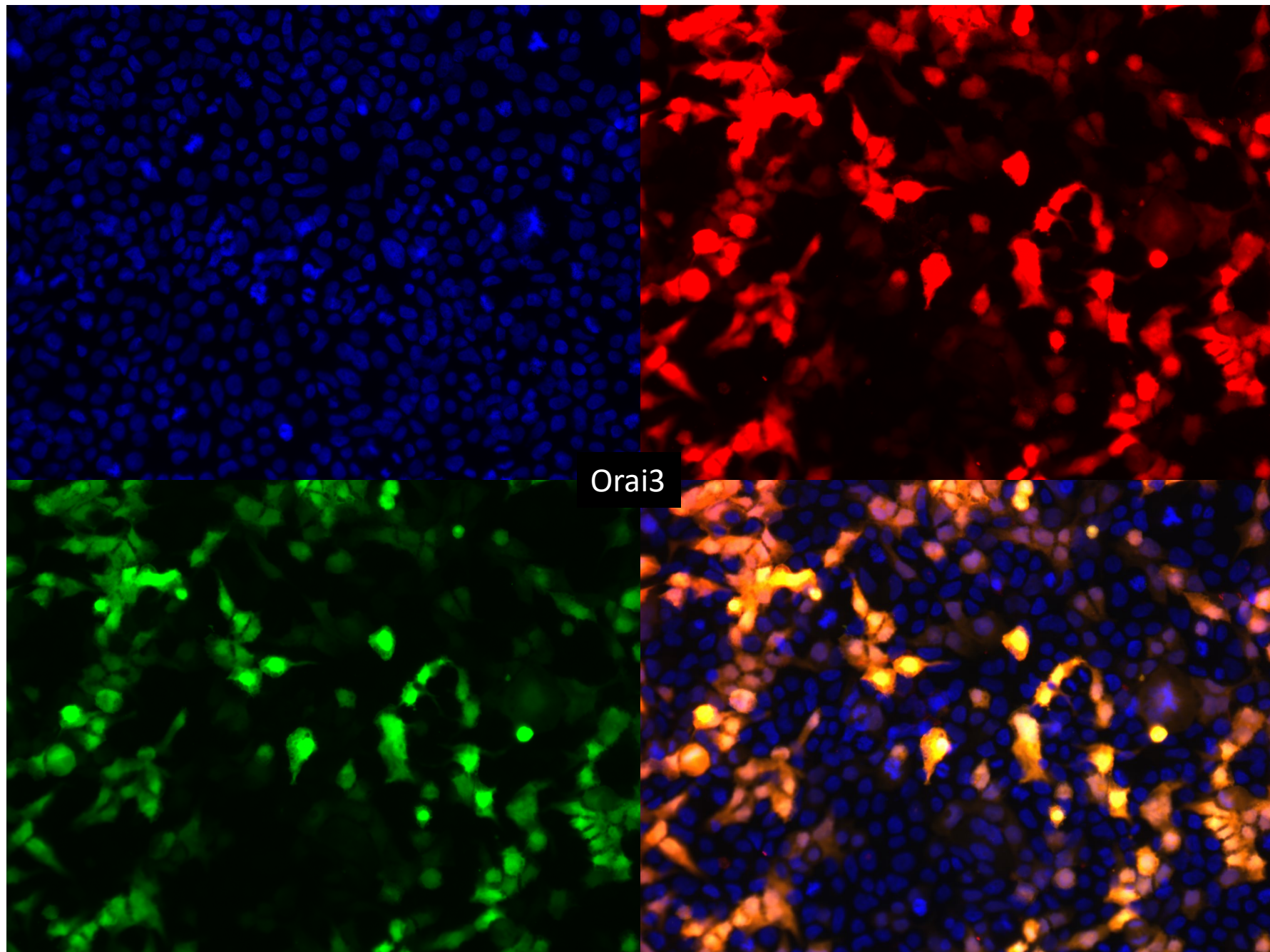

Orai3

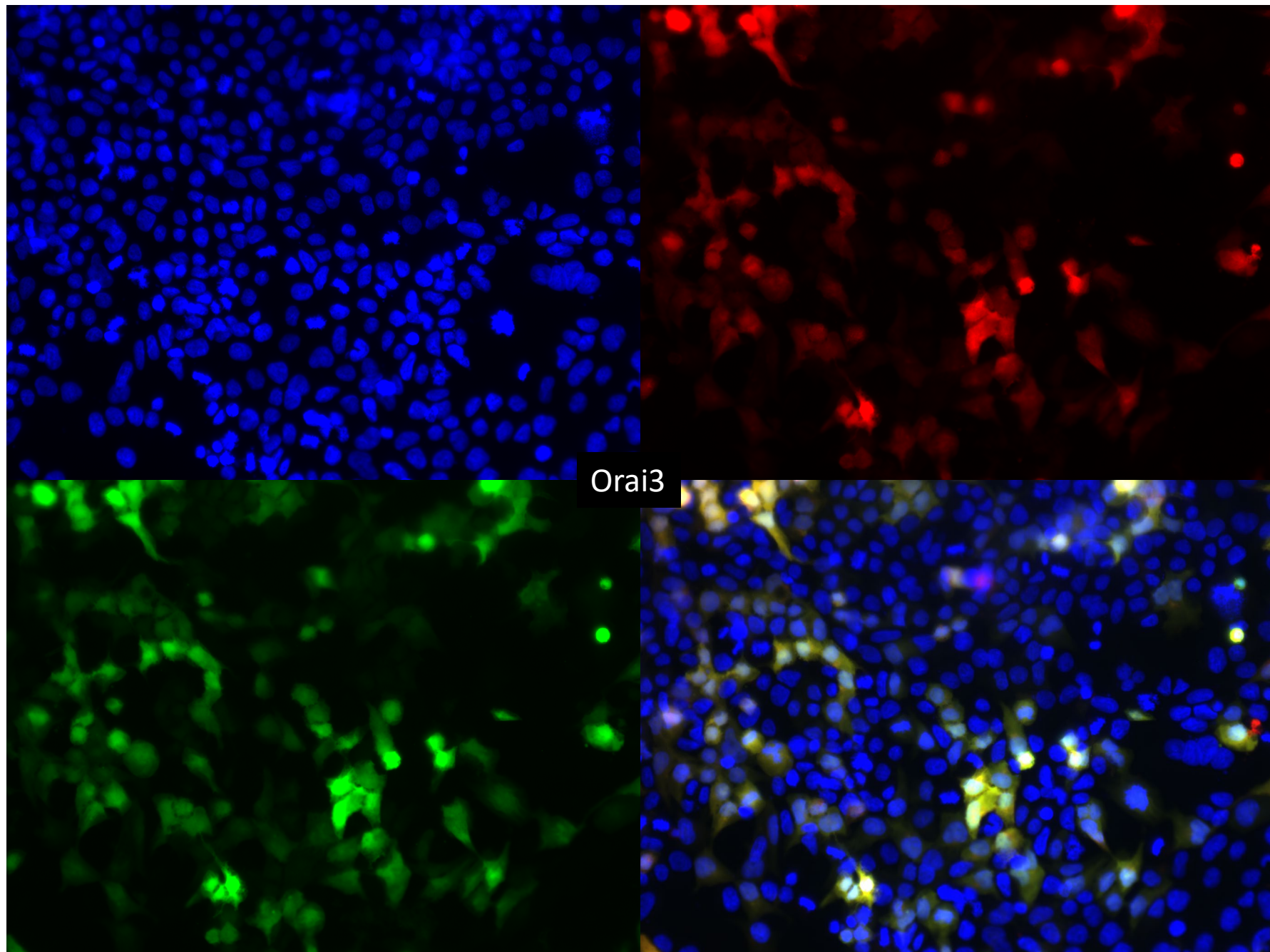

Orai3

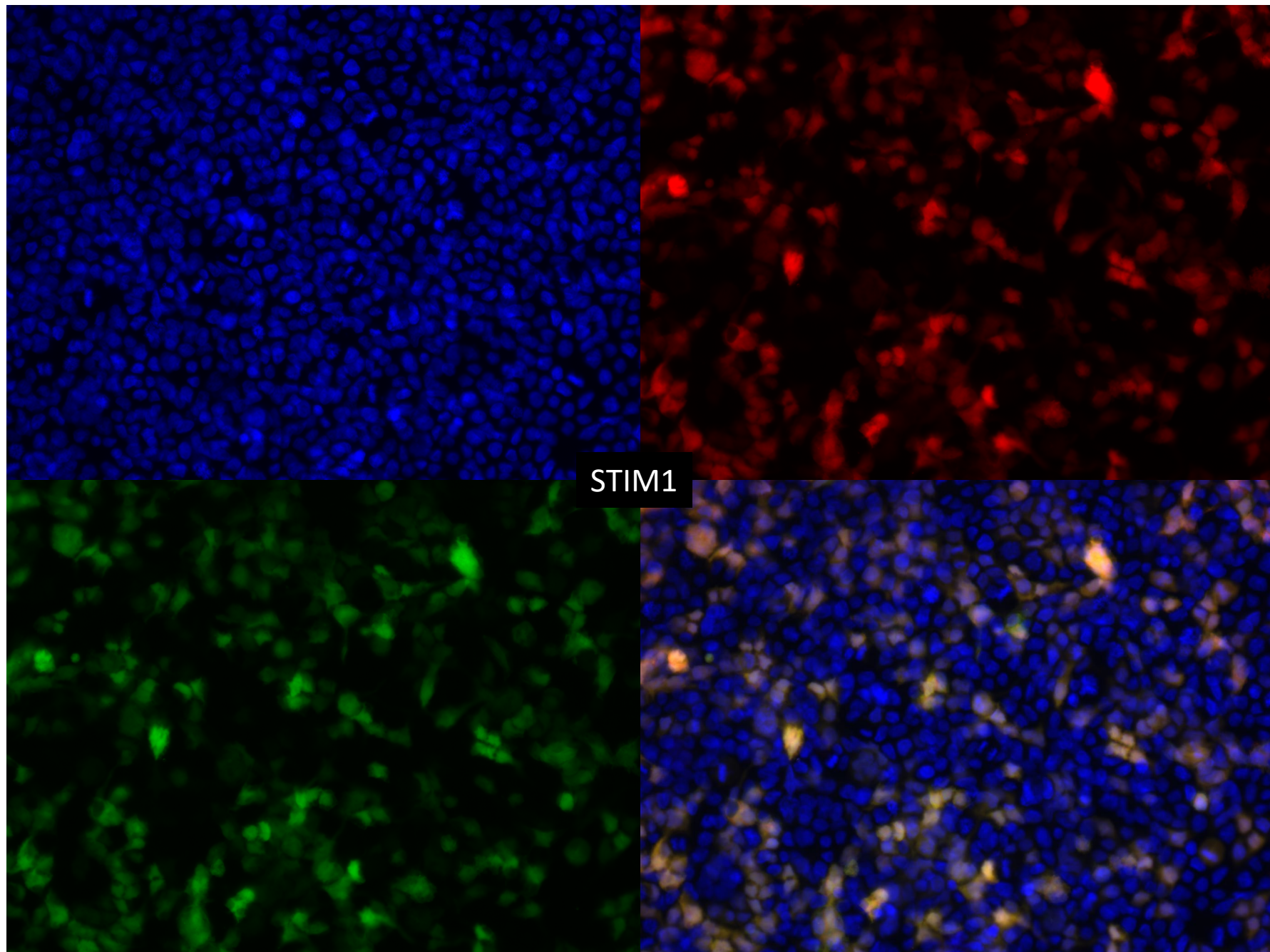

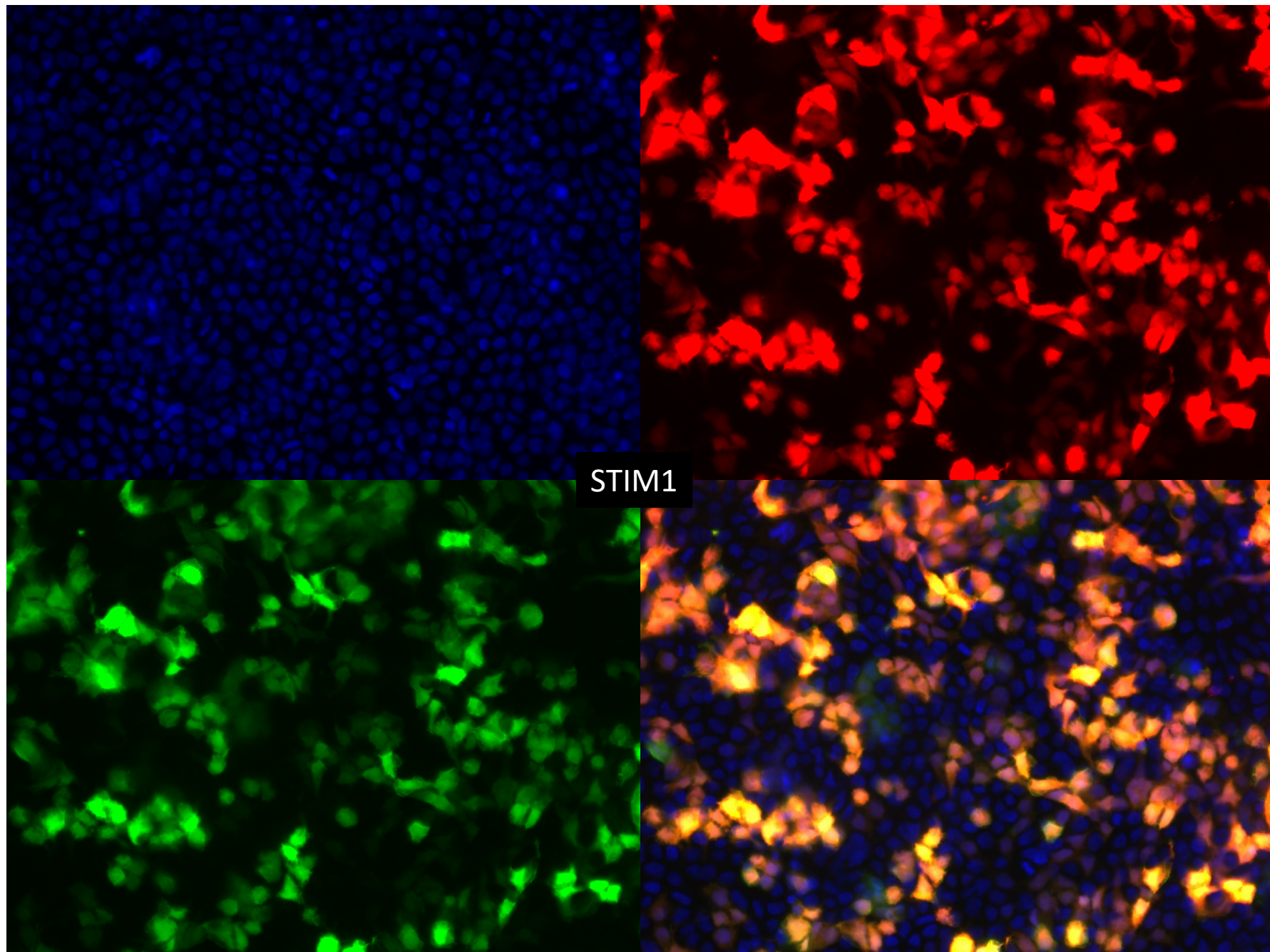

STIM1

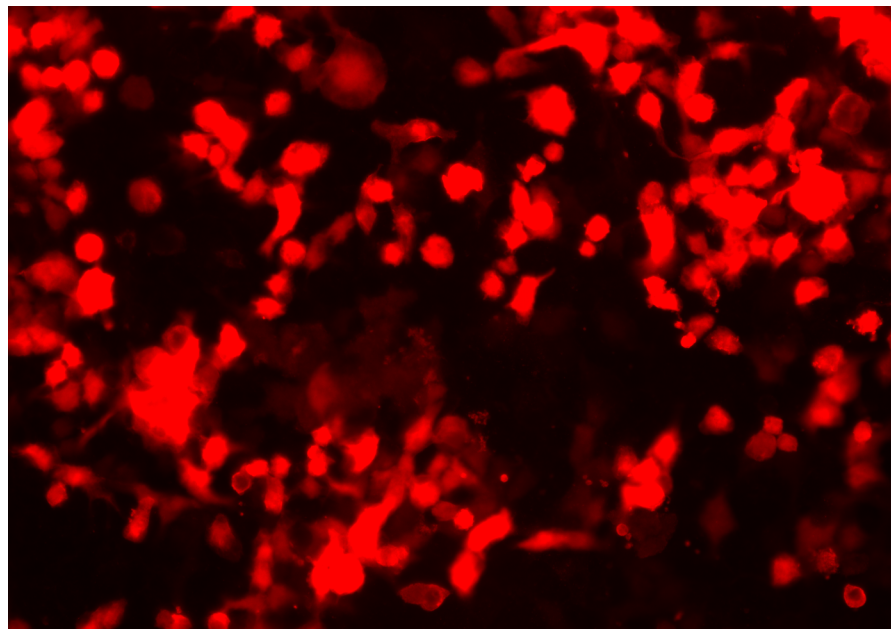

STIM1

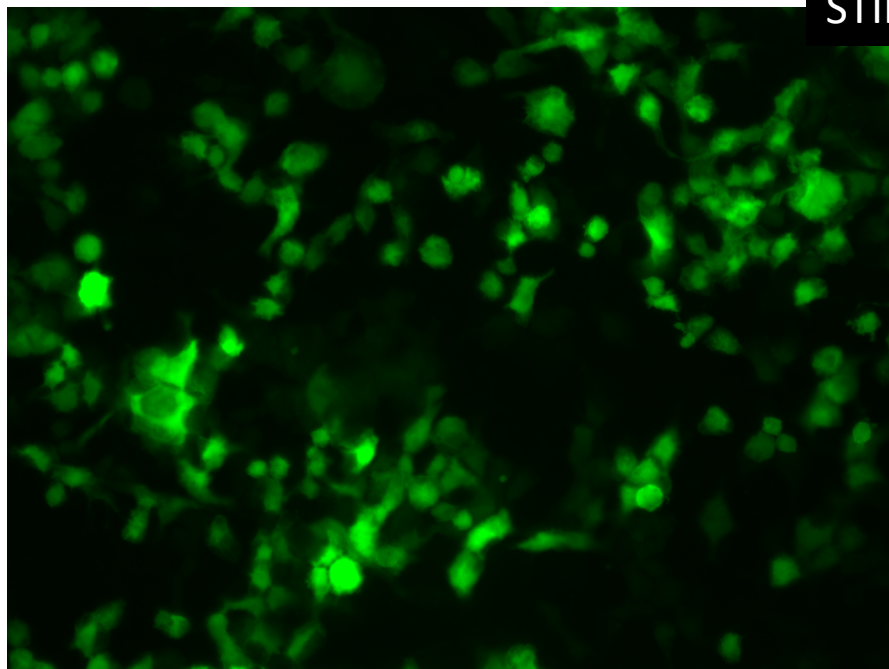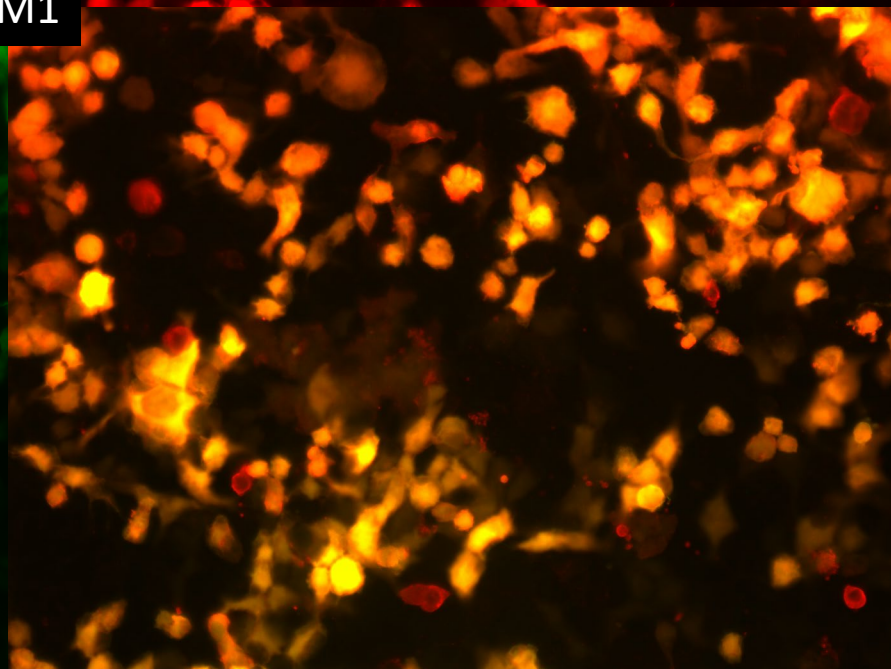

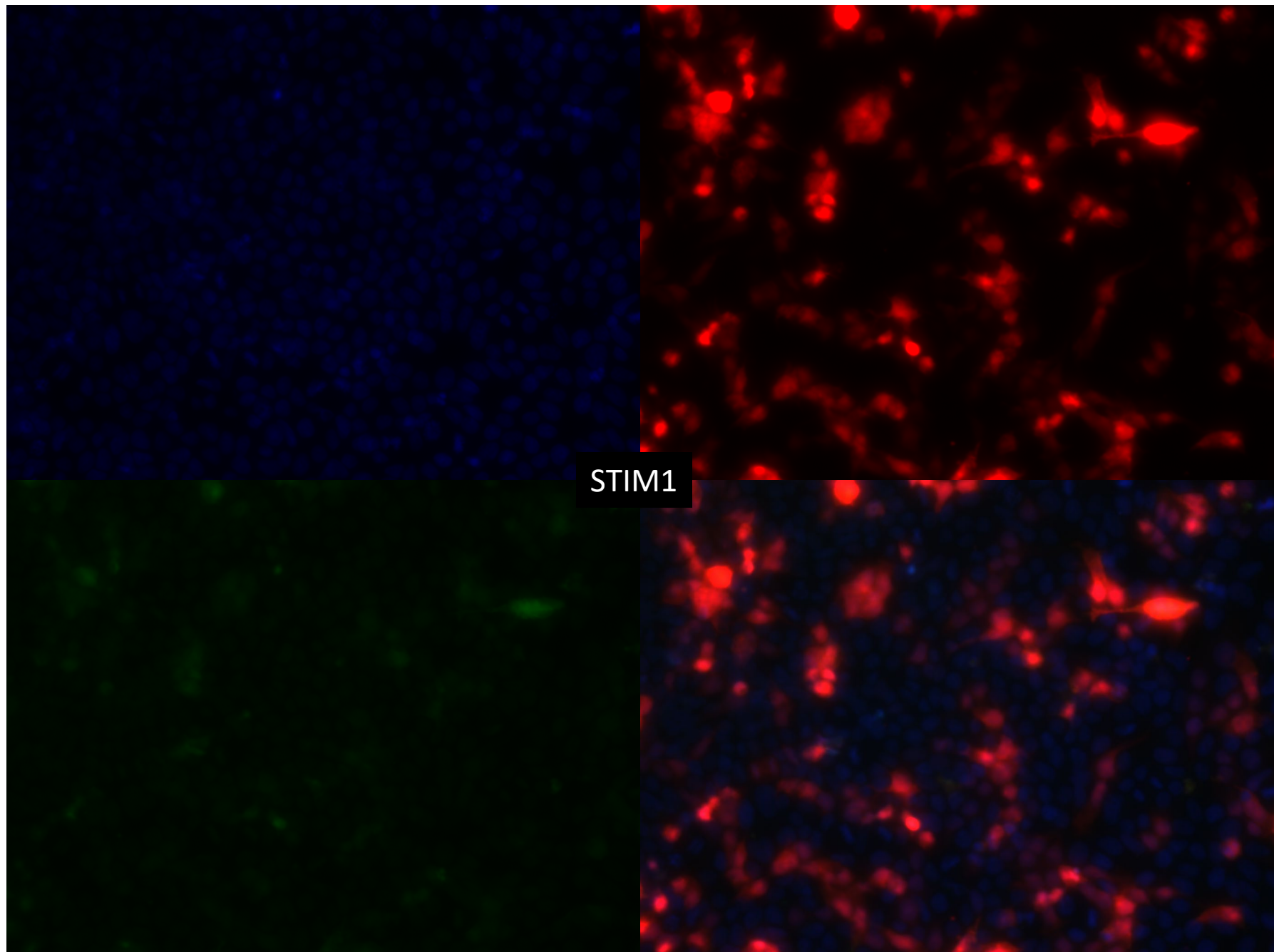

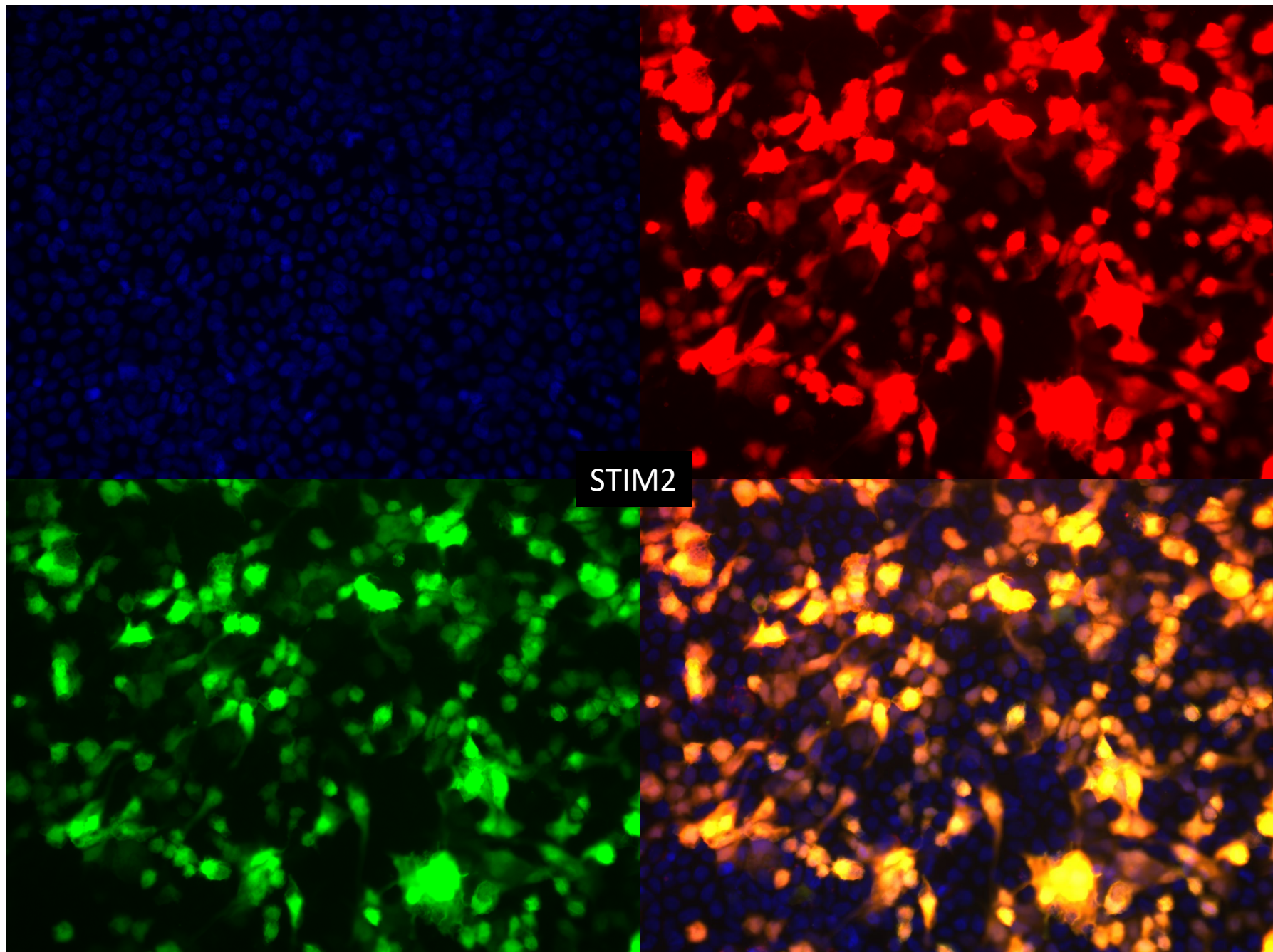

STIM2

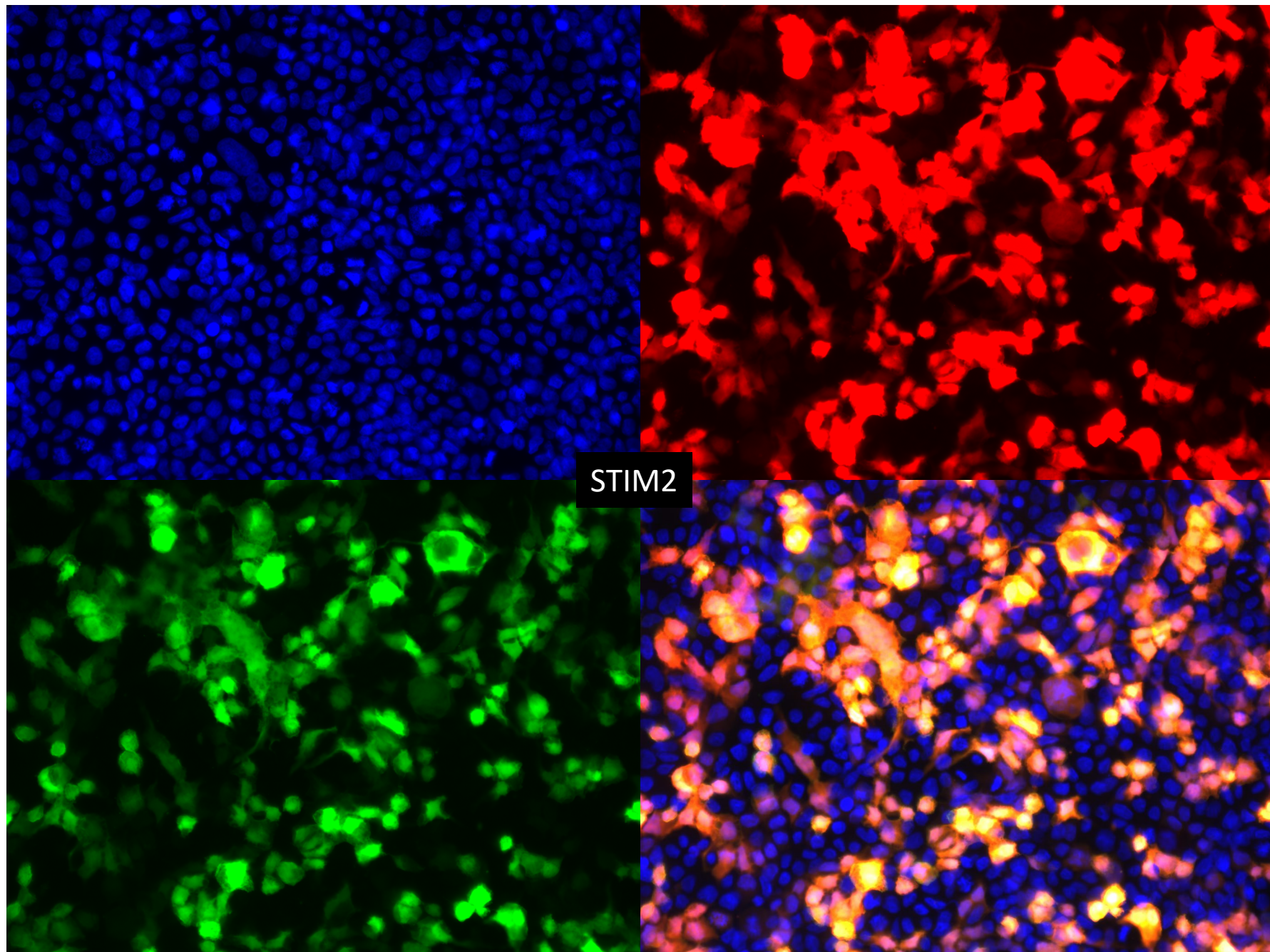

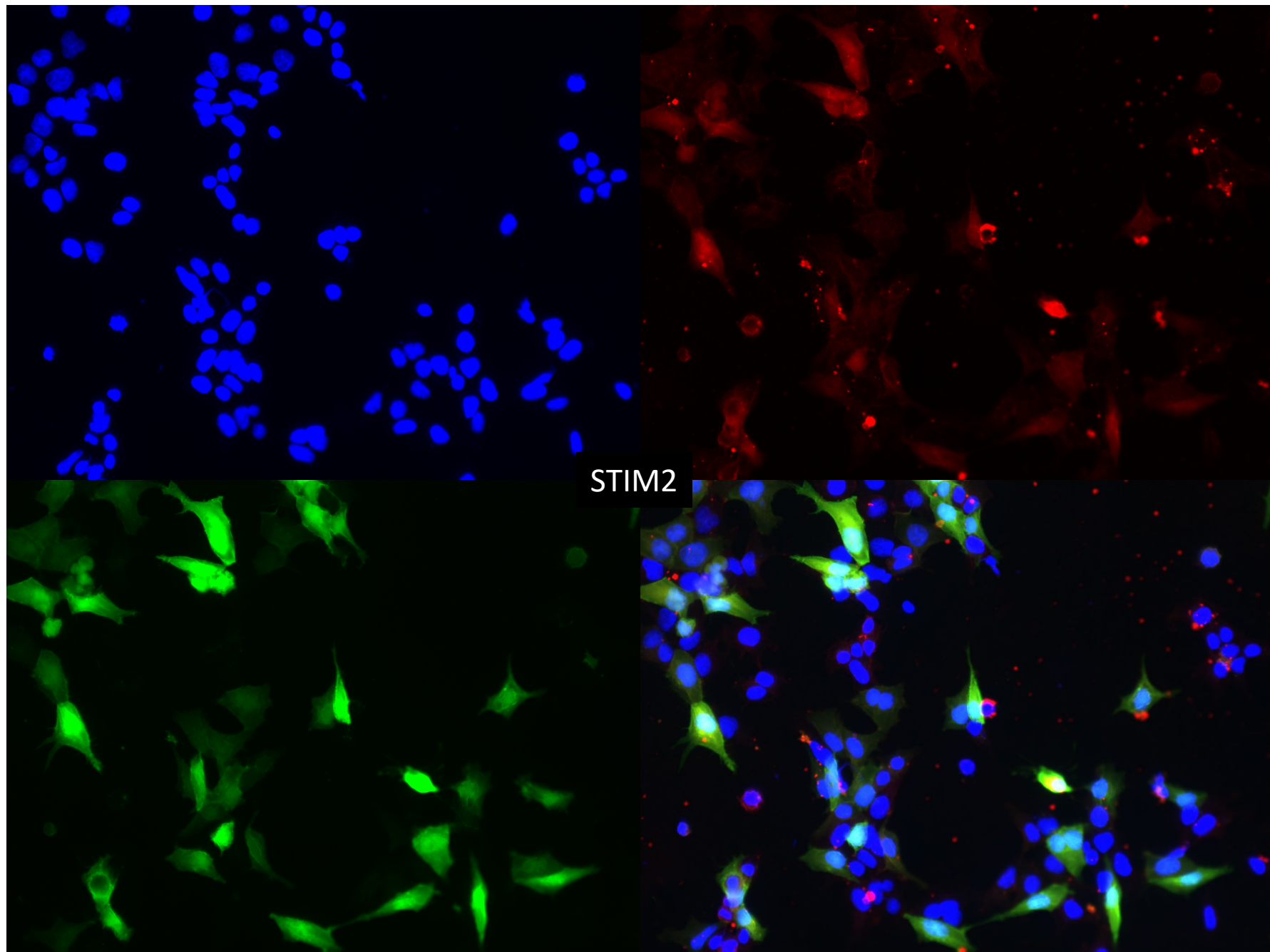

STIM2

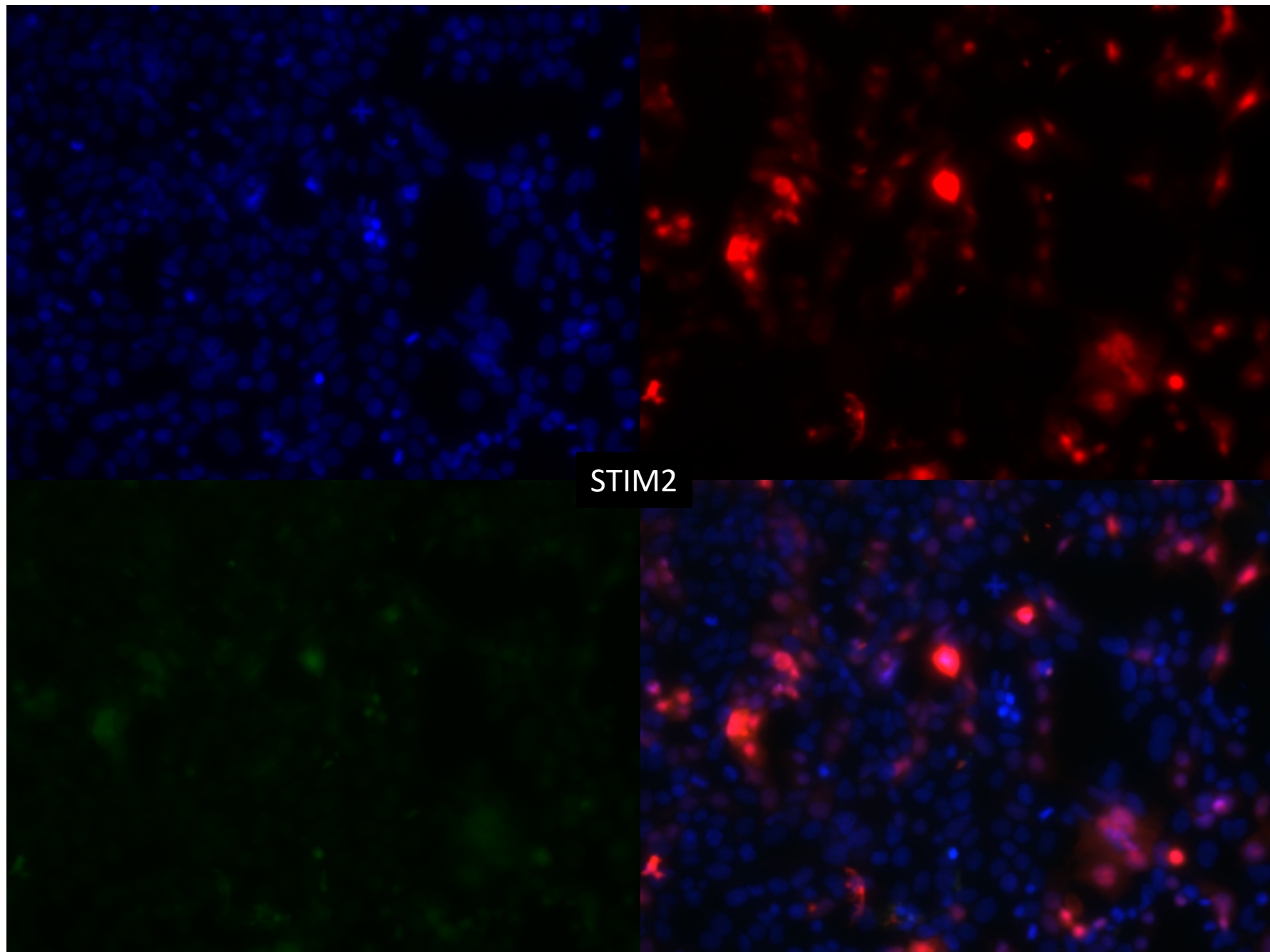

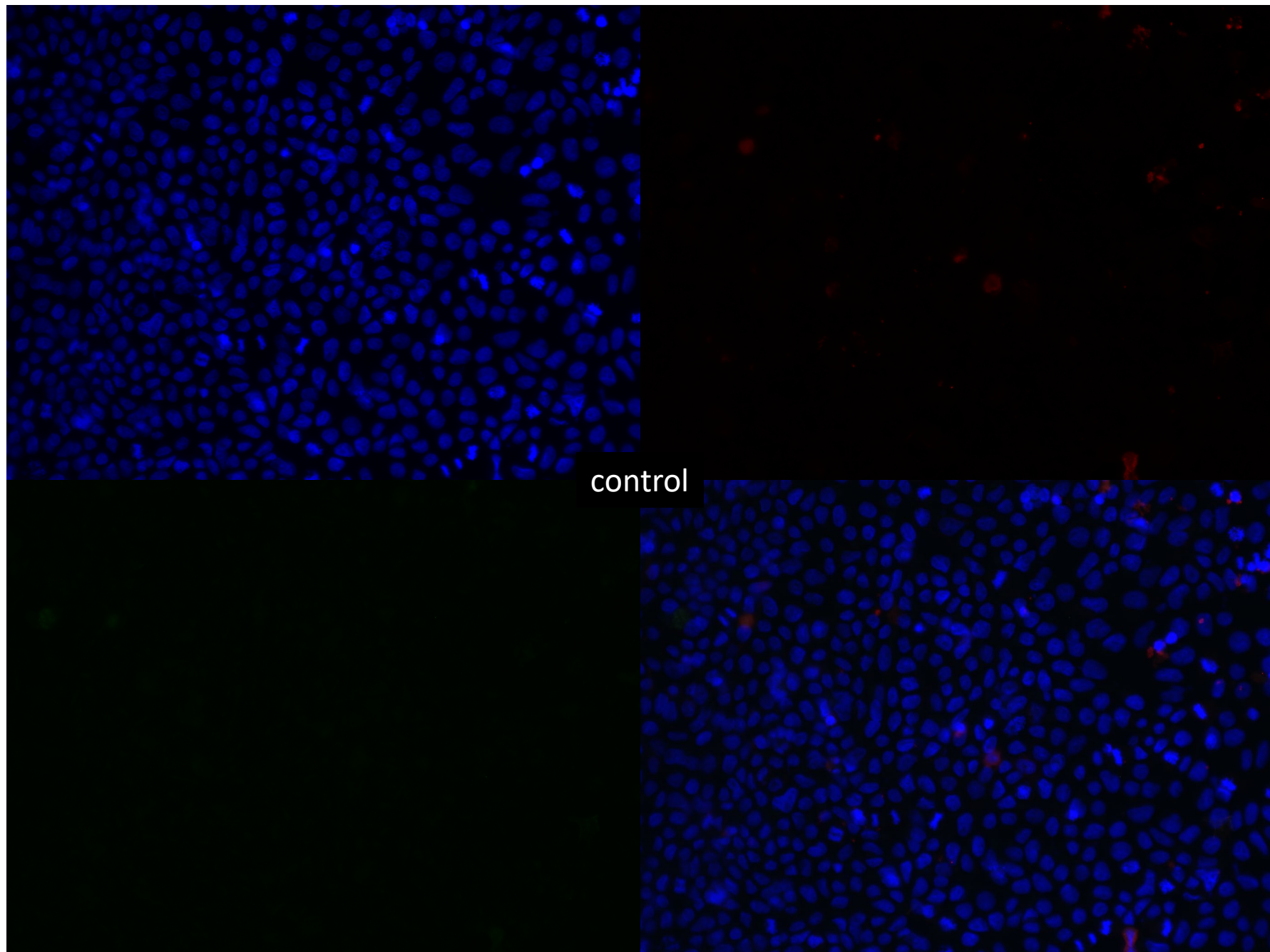

control

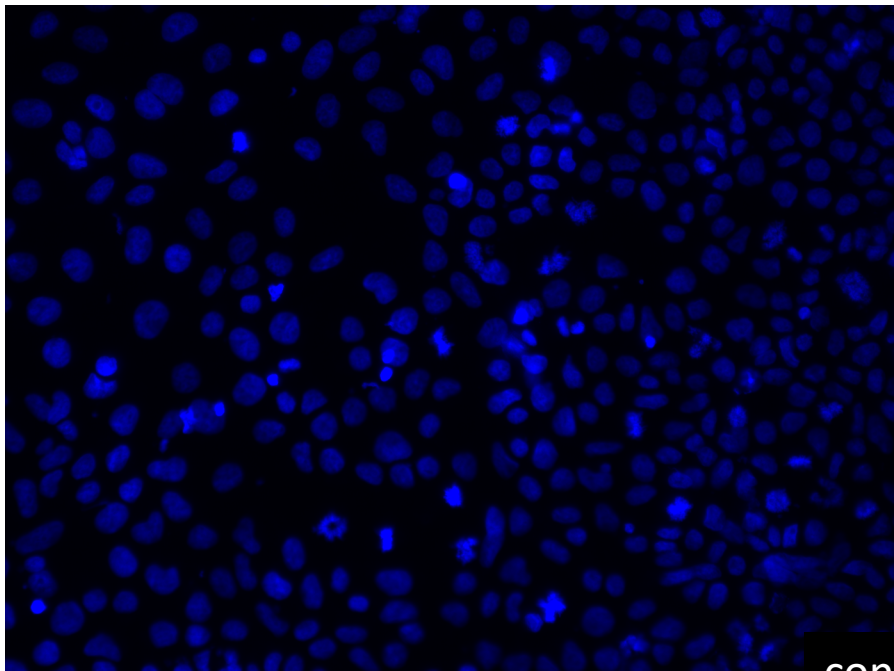

control

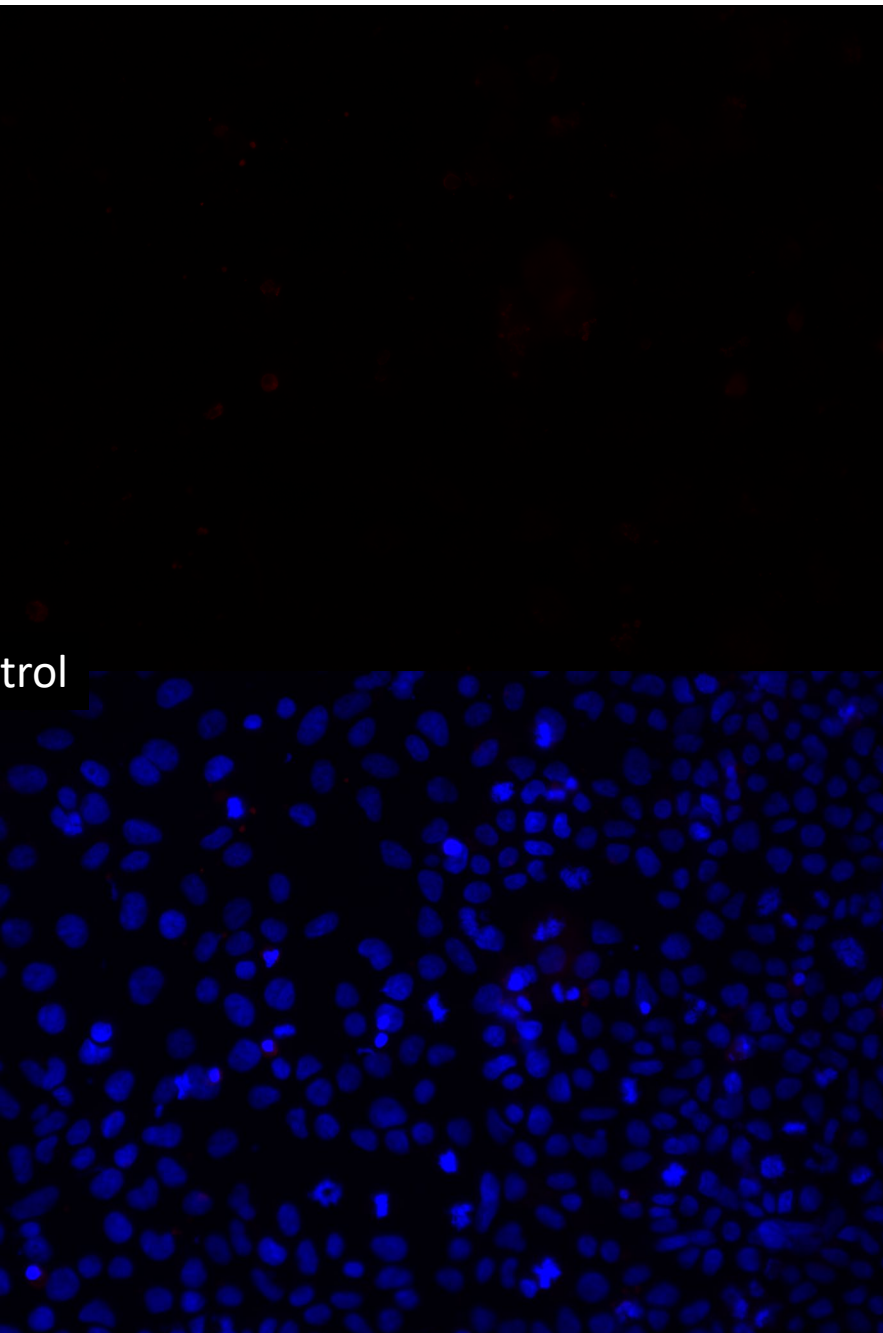

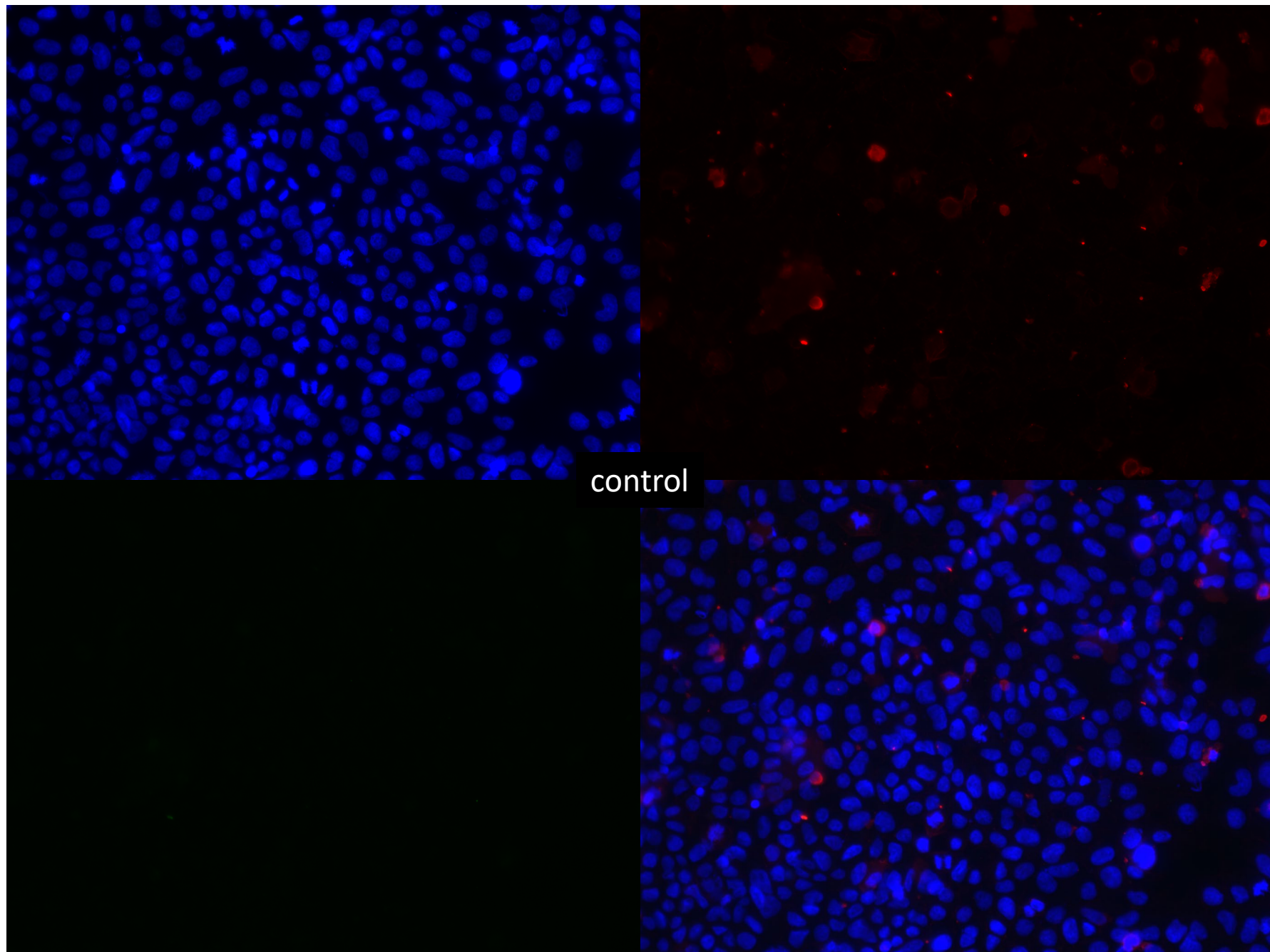

control

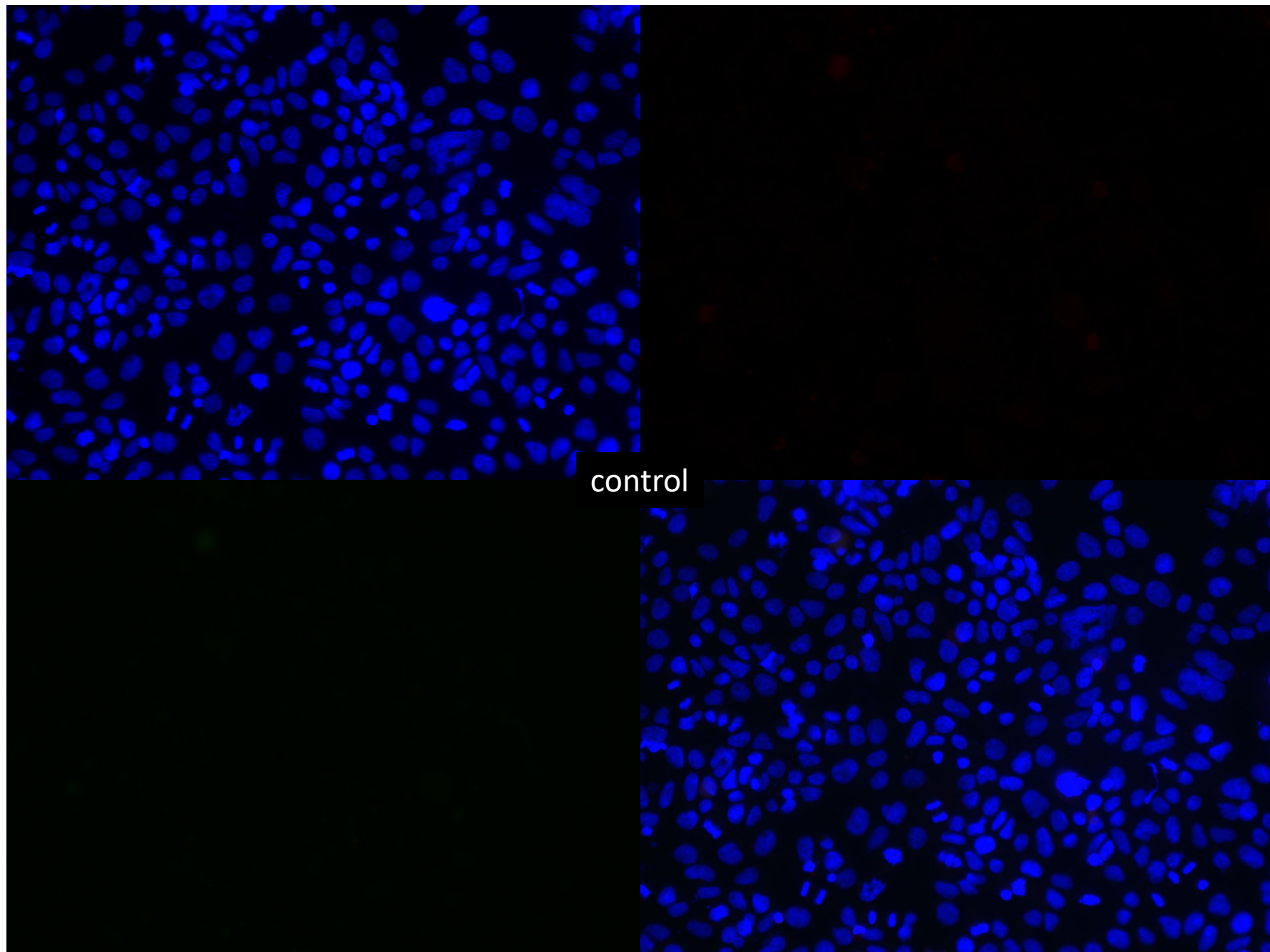

control

Supplement: Supplementary file 4 — Source Data for Expanded View and Appendix [file EMBJ-42-e111348-s004.zip › Source_Data_for_EV_and_Appendix/Figure_EV3/EMBOJ-2022-111348R1_SourceDataForFigEV3.pdf]
