# Supplementary material for: STIM1 and ORAI1 form a novel cold transduction mechanism in sensory and sympathetic neurons
Source: EMBO J. 2022 Dec 16;42(3):e111348. doi: 10.15252/embj.2022111348 (PMC9890232; doi:10.15252/embj.2022111348)
Supplement: Supplementary file 5 — PDF+ [file EMBJ-42-e111348-s009.pdf]

Article

SOURCE DATA TRANSPARENT PROCESS OPEN ACCESS

THE EMBO JOURNAL

STIM1 and ORAI1 form a novel cold transduction mechanism in sensory and sympathetic neurons

Tamara J Buijs[1,†], Bruno Vilar[1], Chun-Hsiang Tan[2,‡,§] & Peter A McNaughton[1,*]

Abstract

Moderate coolness is sensed by TRPM8 ion channels in peripheral sensory nerves, but the mechanism by which noxious cold is detected remains elusive. Here, we show that somatosensory and sympathetic neurons express two distinct mechanisms to detect noxious cold. In the first, inhibition by cold of a background outward current causes membrane depolarization that activates an inward current through voltage-dependent calcium (Ca_V) channels. A second cold-activated mechanism is independent of membrane voltage, is inhibited by blockers of ORAI ion channels and by down-regulation of STIM1, and is recapitulated in HEK293 cells by co-expression of ORAI1 and STIM1. Using total internal reflection fluorescence microscopy we found that cold causes STIM1 to aggregate with and activate ORAI1 ion channels, in a mechanism similar to that underlying store-operated calcium entry (SOCE), but directly activated by cold and not by emptying of calcium stores. This novel mechanism may explain the phenomenon of cold-induced vasodilation (CIVD), in which extreme cold increases blood flow in order to preserve the integrity of peripheral tissues.

Keywords calcium influx; cold sensation; ORAI; sensory neuron; STIM
Subject Category Membranes & Trafficking
DOI 10.15252/embj.2022111348 | Received 4 April 2022 | Revised 25 October 2022 | Accepted 10 November 2022 | Published online 16 December 2022
The EMBO Journal (2023) 42: e111348

Introduction

A conscious sensation of temperature is initiated when thermally-sensitive ion channels expressed in peripheral nerve endings are activated by temperature changes, leading to membrane depolarization and the generation of action potentials that propagate to the spinal cord and from there to higher centres responsible for thermal sensation. To date, all of the ion channels known to generate distinct thermal sensations are thermo-TRP channels, members of the large TRP ion channel family. The sensation of noxious heat is generated by three TRP channels, namely TRPV1 (Cesare & Mcnaughton, 1996; Caterina *et al*, 1997), TRPM3 (Vriens *et al*, 2011), and TRPA1 (Vandewauw *et al*, 2018), whose thermal activation overlaps in the temperature range that humans describe as painfully hot. Non-noxious warmth is signalled by TRPM2 (Tan & McNaughton, 2016; Vilar *et al*, 2020), while non-noxious coolness is signalled by TRPM8 (McKemy *et al*, 2002; Peier *et al*, 2002; Bautista *et al*, 2007; Dhaka *et al*, 2007).

The molecular basis for the detection of painful levels of cold, however, remains unresolved. TRPA1 has been suggested as a noxious cold sensor (Story *et al*, 2003) but *in vivo* studies showed no change in the sensation of strong cold when TRPA1 was genetically deleted (Knowlton *et al*, 2010). In agreement, TRPA1 was found to be activated by extreme cold when expressed in some cells but not in others, possibly because TRPA1 is activated by increases in intracellular calcium, rather than being directly activated by cold, and cold increases intracellular calcium only in some cells (Zurborg *et al*, 2007). A second candidate, TRPC5, was found to contribute to cold responses of DRG neurons *in vitro*, but TRPC5 KO mice displayed no difference in temperature preference compared with WT mice (Zimmermann *et al*, 2011). A third potential mechanism for generating a sensation of extreme cold arises from the observation that cold suppresses the activity of K^+ channels belonging to the 2-pore family (K2P channels), such as TREK and TRAAK channels (Lesage *et al*, 2000; Viana *et al*, 2002; Noël *et al*, 2009; Pereira *et al*, 2014; Viatchenko-Karpinski *et al*, 2018). Suppression of K2P ion channel activity by cold can therefore lead to membrane depolarization by background inward currents and to neuronal excitation. In the present work, we confirm that suppression of a background K^+ current by strong cold causes membrane depolarization and activation of voltage-dependent calcium channels in both sensory and sympathetic neurons.

Finally, a calcium entry was found to be activated by the removal of a heat stimulus in non-neuronal cells transfected with STIM1 and ORAI1 (Xiao *et al*, 2011) and in keratinocytes (Liu *et al*, 2019a). A second study, also on keratinocytes, found a calcium increase in response to a mild cool stimulus (Sadler *et al*, 2020), but the mechanism was not determined in that study. STIM1 is a protein, located in the membranes of subcellular calcium stores, that detects the degree of store filling by calcium (Prakriya & Lewis, 2015; Qiu &

1 Wolfson Centre for Age-Related Diseases, King's College London, London, UK
2 Department of Pharmacology, University of Cambridge, Cambridge, UK
*Corresponding author. Tel: +44 7780 674039; E-mail: peter.mcnaughton@kcl.ac.uk
†Present address: Department of Synapse and Network Development, Netherlands Institute for Neuroscience, Amsterdam, The Netherlands
‡Present address: Department of Neurology, Kaohsiung Medical University Hospital, Kaohsiung, Taiwan
§Present address: Graduate Institute of Clinical Medicine, College of Medicine, Kaohsiung Medical University, Kaohsiung, Taiwan

© 2022 The Authors. Published under the terms of the CC BY 4.0 license.

Lewis, 2019). When stores are depleted of calcium, STIM1 migrates within the ER membrane to areas of close ER-plasma membrane apposition, where it binds to and activates ORAI1, a surface membrane calcium-selective ion channel, and the resulting calcium influx refills stores in a process known as store-operated calcium entry (SOCE). The studies showing an activation of a STIM1-ORAI1 mechanism in the mild temperature range (Liu *et al*, 2019a) were conducted on keratinocytes and it is not clear how they might relate to the sensation of cold in sensory neurons.

Here, we show that extreme cold causes aggregation and activation of calcium-permeable ORAI1 ion channels, located in the surface membrane, around STIM1 puncta present in the endoplasmic reticulum. In contrast to SOCE, however, the calcium increase is triggered by cold alone, without emptying of intracellular calcium stores. Because of the high selectivity of ORAI1 ion channels for calcium, the calcium influx mediated by this mechanism does not generate a detectable inward current and thus does not cause significant membrane depolarization that could trigger nerve activity and signal a sensation of cold to higher centres. The STIM1-ORAI1-dependent calcium influx is present in somatosensory neurons, where it may contribute to local reactions to noxious cold, and it is also prominent in sympathetic neurons, where it may be responsible for triggering cold-induced vasodilation, an important protective mechanism that restores skin blood flow in conditions of extreme cold and thus protects exposed peripheral tissues from damage by frostbite.

Results

DRG and SCG neurons express novel mechanisms that detect noxious cold

The sensitivity to mild cool stimuli of a small subset of somatosensory neurons from the dorsal root ganglion (DRG) is mediated by expression of TRPM8, but other cold-sensitive DRG neurons do not express TRPM8 and instead respond to cold by an unknown mechanism that is also present in sympathetic neurons (Munns *et al*, 2007). To decipher the contribution of TRP channels to cold sensation in peripheral neurons, we exposed neurons from the DRG and from the sympathetic superior cervical ganglion (SCG) to a cold ramp from 32°C to 4°C while measuring the intracellular calcium level, and in the same neurons we tested the responses to agonists for the potentially cold-activated TRP channels TRPM8, TRPA1 and TRPC5 (Figs 1A and B and EV1). The starting temperature of 32°C was chosen because it is similar to the temperature of peripheral skin; a starting temperature of 37°C (core body temperature) made no significant difference to cold responses (Appendix Fig S1). The fluorescence recorded at the individual excitation wavelengths of 340 and 380 nm was significantly affected by temperature (Appendix Fig S2), but the $F_{340/380}$ ratio was only slightly temperature-dependent, with a small and steady decrease observed at low temperatures in the absence of external calcium, in contrast to the large calcium increases observed in the presence of external calcium (Appendix Fig S2; two different types of responses shown, as defined below in Figs 2 and 3). Finally, the possibility that a temperature-dependent change in solution pH might have affected calcium signals was tested in Appendix Fig S3; a temperature change from 34°C to 4°C caused a small change in solution pH (from pH 7.2 to 7.5) but a similar pH change evoked only a very small increase $F_{340/380}$.

Overall, 14% of DRG neurons responded with an increase in intracellular calcium to a cold ramp to 4°C (Figs 1C and EV1A); 23% of these cold-sensitive DRG neurons (around 3% of the total number of neurons) expressed TRPM8, as judged from their response to menthol, a selective agonist for TRPM8. TRPA1 was expressed in 31% of DRG neurons overall; most of these TRPA1-positive neurons co-expressed TRPC5 (Fig EV1B) but only a small fraction of these was cold-sensitive (Fig EV1C). Classification of DRG neurons by their expression of these three TRP channels revealed that TRPM8 expression is more highly correlated with cold-sensitivity than TRPA1 or TRPC5 expression, because most neurons (73%) that responded to the TRPM8 agonist menthol also responded to cold, whereas most neurons that responded to TRPA1 or TRPC5 agonists did not respond to cold (36 and 32% cold-responsive, respectively, see Fig EV1C). A possible reason why the expression of TRPA1 and/or TRPC5 is poorly correlated with sensitivity to cold could be that another feature of neurons that express TRPA1 or TRPC5 may confer cold-sensitivity, such as the presence of TRP-independent cold sensors, whose existence we demonstrate

Figure 1. DRG and SCG neurons express novel mechanisms to detect noxious cold.

A, B Representative images of changes in intracellular calcium, as measured in fluorescence ratio images ($F_{340/380}$) (A) and traces (B). Examples show a TRPM8-expressing DRG neuron (left in A, top in B) responding to both the TRPM8 agonist menthol (300 μM) and to a cold stimulus from 32°C to 4°C (temperature trace bottom in B), and a neuron that responds to cold (right in A, middle in B) but does not express any of TRPM8, TRPA1 or TRPC5, as shown by lack of response to the TRPM8 agonist menthol (300 μM), the TRPA1 agonist AITC (50 μM) or the TRPC5 agonist rosiglitazone (100 μM). Calcium increase is due to influx from extracellular solution (see Fig 2A and B).

C Venn diagram showing the proportion of neurons that responded to at least one of the three TRP agonists menthol, AITC or rosiglitazone (grey, 44%) and those that responded to cold (blue, 14%). Neurons that responded to cold, but not to any TRP agonist (5%) were classified as "novel cold-sensitive neurons" (n = 859 neurons imaged on 3 separate days).

D Frequency histogram showing cold activation thresholds of novel cold-sensitive DRG neurons (left, mean cold threshold 15.1 ± 1.0°C, n = 40 neurons) and $TRPM8^+$ neurons (right, mean cold threshold 23.3 ± 2.1°C, n = 15 neurons).

E, F Similar experiments to (A) and (B) showing an SCG neuron responding to the TRPA1 agonist AITC (left in E and top in F) and a novel cold-sensitive SCG neuron that responds to cold but does not express any of TRPM8, TRPA1 or TRPC5 (right in E and middle in F). Many glial cells are also visibly activated by AITC but not by cold.

G Similar Venn diagram to C for SCG neurons (n = 155 neurons). Few responded to any of the TRP agonists (grey, 6%), while many neurons responded to cold (blue, 44%), of which most responded only to cold (39% "novel cold-sensitive neurons").

H Similar histogram to (D) showing cold activation thresholds of novel cold-sensitive SCG neurons (mean cold threshold 13.1 ± 0.8°C, n = 61 neurons). See Fig EV1 for further data on distribution of cold-sensitivity in neurons of the DRG and SCG.

Source data are available online for this figure.

© 2022 The Authors

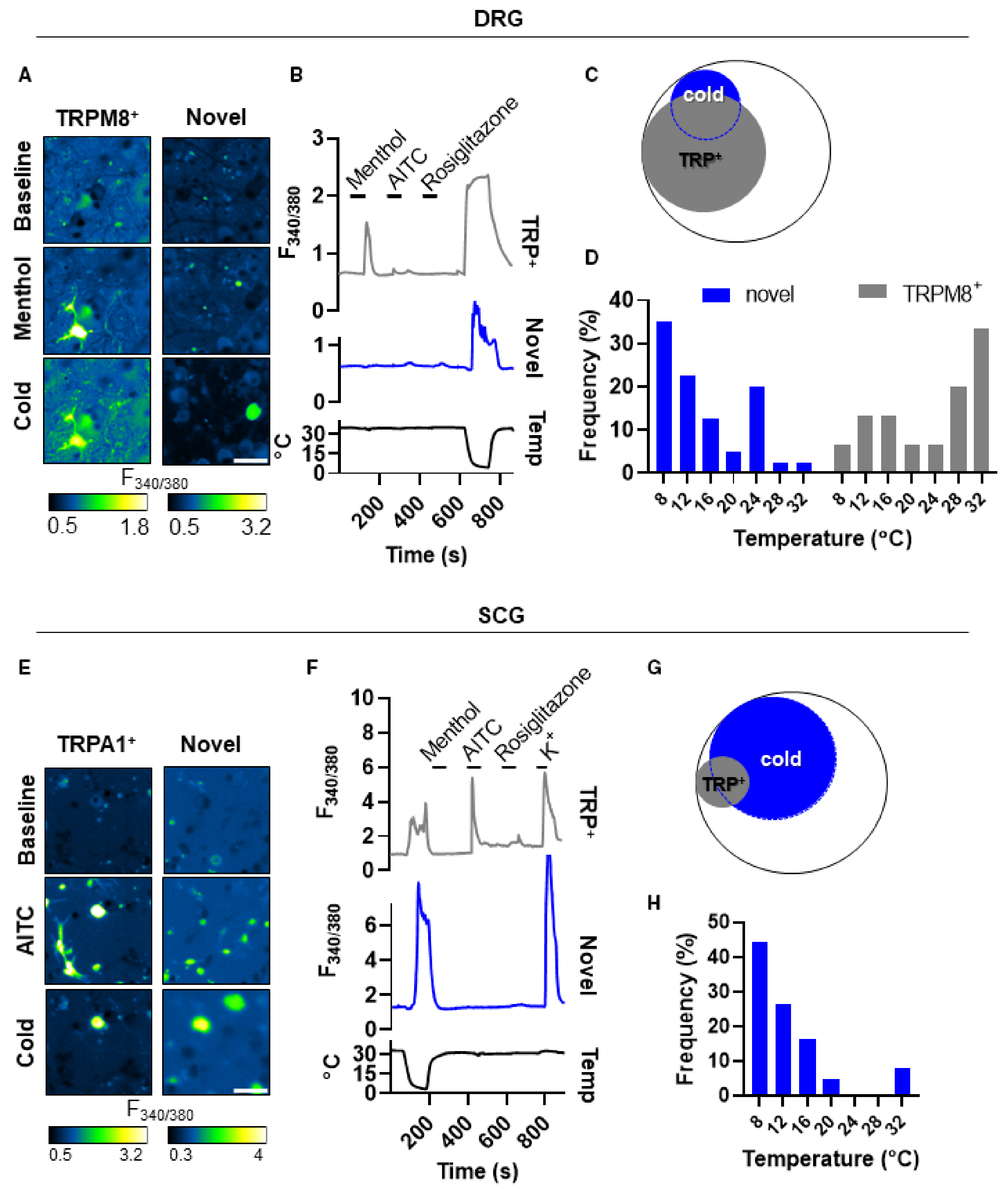
DRG
A
TRPM8⁺
Novel
Baseline
Menthol
Cold
F340/380
0.5 1.8 0.5 3.2
B
Menthol
AITC
Rosiglitazone
F340/380
TRP⁺
Novel
°C
Temp
Time (s)
C
cold
TRP⁺
D
novel
TRPM8⁺
Frequency (%)
Temperature (°C)
SCG
E
TRPA1⁺
Novel
Baseline
AITC
Cold
F340/380
0.5 3.2 0.3 4
F
Menthol
AITC
Rosiglitazone
K⁺
F340/380
TRP⁺
Novel
°C
Temp
Time (s)
G
cold
TRP⁺
H
Frequency (%)
Temperature (°C)

Figure 1.

below. Expression of TRPA1 and TRPC5 may therefore be unrelated to the cold responses of sensory neurons.

A more surprising observation was that in 35% of cold-sensitive DRG neurons, no calcium increase was observed in response to any of the agonists for TRPM8, TRPA1 or TRPC5. We named these "novel cold-sensitive neurons," in which the cold response is not attributable to any of the potential TRP candidates for cold sensitivity (Figs 1C and EV1A and B). The average thermal response threshold of novel cold-sensitive DRG neurons was 15.1 ± 1.0°C (Fig 1D left, mean ± SEM). The mean activation threshold of TRPM8-expressing DRG neurons, on the other hand, was at a much less cold 23.3 ± 2.1°C, well within the range of non-noxious coolness (Fig 1D right panel, $P < 0.001$ compared with novel cold-sensitive DRG neurons). Note that the spread of the thresholds of cold-sensitive neurons around the mean values is large in both cases, however, and that the behavioural sensitivity to a cold stimulus is likely to be determined more by neurons responding towards the upper end of the range than by the mean values.

Novel cold-sensitive neurons were even more prominent in neurons from the SCG, in which 44% of neurons responded to cold, but of these cold-sensitive neurons, only 10% also responded to one or more TRP channel agonists (Figs 1E–G and EV1D, E). No SCG neurons responded to menthol, indicating that TRPM8 is not expressed in the SCG, while 10% of cold-sensitive SCG neurons responded to AITC, a selective TRPA1 agonist, or rosiglitazone, a selective TRPC5 agonist, indicating that they express TRPA1 and/or TRPC5 (Fig EV1E). Thus, 90% of cold-sensitive SCG neurons, or nearly 40% of the total number of SCG neurons, express a novel mechanism (or mechanisms) for generating an internal calcium increase in response to cold. The average cold activation threshold of novel cold-sensitive SCG neurons was 13.1 ± 0.8°C (Fig 1H), similar to the value of 15.1 ± 1.0°C found in DRG neurons (see above). Because of the prevalence of a novel cold-sensitive mechanism or mechanisms in SCG neurons, we mainly used SCG neurons in subsequent experiments to understand the molecular identity of the novel cold sensor(s).

In summary, the present results agree with earlier work showing that TRPM8-expressing neurons are activated at a temperature corresponding to mild non-noxious coolness (McKemy *et al*, 2002; Peier *et al*, 2002; Bautista *et al*, 2007; Dhaka *et al*, 2007), while the novel cold-sensitive mechanism(s) characterized here are activated at temperatures around 10°C colder, in the range of painful cold sensation.

Peripheral neurons express both a voltage-dependent and a voltage-independent cold transduction mechanism

Fig 2A and B show that no cold-activated calcium increase is observed in the absence of extracellular calcium, demonstrating that the origin of the intracellular calcium increase in response to cold is influx through the plasma membrane rather than release from intracellular stores. Concurrent Ca^{2+} imaging and patch clamp electrophysiology in current-clamp mode (Fig 2C) shows that an SCG neuron initially depolarizes gradually in response to cold, with a relatively small increase in $[Ca^{2+}]_i$ (from $F_{340/380}$ of around 1.0 to 2.0), but that a subsequent sharp depolarization causes a larger increase in $[Ca^{2+}]_i$ (marked by vertical dotted line, from $F_{340/380}$ of around 2 to 4). The second depolarization and calcium increase are characteristic of activation of voltage-dependent calcium channels, a proposal that is supported by voltage clamp at −60 mV (Fig 2D), in which the early calcium increase (increase of $F_{340/380}$ from around 1.0 to 2.0) was still seen, but the later depolarization-activated calcium increase was abolished. Note that the calcium influx seen under voltage-clamp at −60 mV is not associated with an inward current, and in fact the current trace shows a small change in the outward direction (<14pA, Fig 2D).

These observations support the existence of two distinct sources of cold-induced calcium influx: a depolarization-activated calcium influx, typical of voltage-dependent calcium channels; and a voltage-independent calcium influx of unknown origin. Work described below investigates the following questions: (i) What causes the depolarization to the threshold for activation of voltage-dependent calcium channels in current clamp mode (Fig 2C)? (ii) What is the origin of the voltage-independent calcium influx (Fig 2D)? The absence of a detectable inward current associated with the intracellular calcium increase in voltage-clamp mode shows that the voltage-independent calcium influx must originate from a near-electroneutral process such as a highly calcium-selective ion channel or a calcium exchange mechanism, and that a cold-activated TRP channel is therefore an unlikely candidate

Figure 2. Electrophysiological dissection of the mechanisms governing cold-induced Ca^{2+} influx.

A Representative Ca^{2+} imaging traces showing cold responses of SCG neurons in absence and presence of extracellular Ca^{2+}. Temperature trace below.
B Collected results of 110 cold-sensitive SCG neurons on 3 coverslips ($P < 0.0001$, RM one-way ANOVA + Dunnett's test). Removal of extracellular Ca^{2+} during the first cold ramp completely prevented cold-induced Ca^{2+} influx. Statistical significance was determined by a repeated measures one-way ANOVA + Dunnett's multiple comparison test.
C Top: membrane potential from current-clamped SCG neuron. Middle: Concurrent Ca^{2+} imaging trace showing cold-induced Ca^{2+} increase. Bottom: temperature trace. Cold causes a slow depolarization and small calcium increase, followed by a sharp depolarization (vertical line) and a second larger rise in $[Ca^{2+}]_i$. Similar results obtained in 11 SCG neurons.
D Top: current trace from SCG neuron voltage-clamped at −60 mV. Middle: Concurrent Ca^{2+} imaging trace showing cold-induced Ca^{2+} increase. Bottom: temperature trace. Cold-induced increase in $[Ca^{2+}]_i$ is not associated with inward current. Similar results obtained in 21 SCG neurons.
E Effect of cold on current–voltage relation of cold-sensitive SCG neuron. Holding voltage −60 mV, voltage ramp from +30 mV to −100 mV in 500 ms applied every 30 s. Top: current trace. Middle: Concurrent Ca^{2+} imaging trace showing cold-induced Ca^{2+} increase. Bottom: temperature trace. Similar results obtained in 10 SCG neurons.
F Voltage ramp used to determine the current voltage relation of SCG neurons before, during and after the cold stimulation in (E).
G Mean current voltage relation of 10 SCG neurons at 32°C (dashed line), 4°C (blue) and 32°C (red dots).
H Difference trace from (G).

Source data are available online for this figure.

© 2022 The Authors

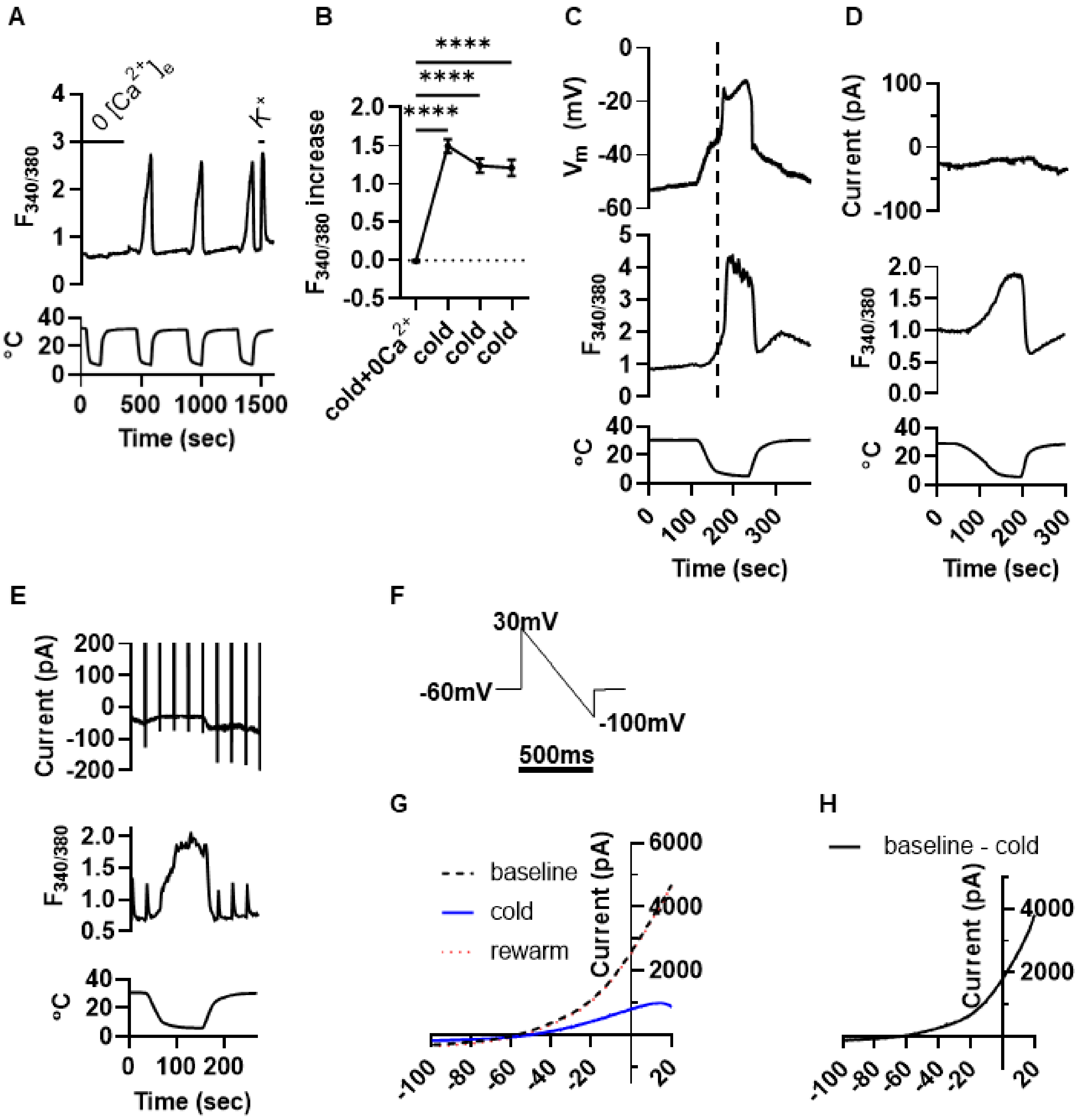

Figure 2.

because most TRP channels are only weakly Ca^{2+}-selective and current is carried mainly by Na^+ ions, producing a large inward current (Cesare & Mcnaughton, 1996; Ramsey *et al*, 2006).

The first question was investigated in the experiments shown in Fig 2E–H, in which a voltage ramp was used to quantify changes in the steady-state background current caused by application of a cold ramp from 31°C to 4°C. Measurement of the neuronal current–voltage relation with a ramped voltage (Fig 2E and F) showed that application of a cold stimulus suppressed an outwardly rectifying current with reversal potential ≈ -60 mV (Fig 2G and H), consistent with a current carried largely by K^+ ions. The reversal potential of this current is negative to the neuronal resting potential, explaining the depolarization observed in response to cold in current-clamped neurons (Fig 2C). The outwardly rectifying IV relation and suppression of the current by cold are both characteristic of a two-pore potassium channel (K2P) such as a member of TREK or TRAAK families

(Maingret *et al*, 2000; Noël *et al*, 2009; Pereira *et al*, 2014). Thus the depolarization-induced calcium entry caused by a cold stimulus is likely to be explained by a suppression of K2P ion channel activity, followed by activation of voltage-dependent calcium channels.

Regarding the second question, the origin of the voltage-independent calcium influx, the experiment of Fig 2D showed that under voltage-clamp the current changed in the *outward* direction on application of the cold stimulus, in the opposite direction to the inward current expected for an influx of calcium ions. An increase in intracellular calcium concentration of the observed size could, however, be generated by a highly calcium-selective inward current of as little as 1pA (see Methods, "Calculation of cold-induced calcium concentration increase"), which would be undetectable against the observed change in background currents caused by cold-dependent suppression of the K2P current.

The experiments shown in Fig 3A–C further investigate the properties of the two distinct cold-activated calcium influx pathways in SCG neurons. Under voltage-clamp at −60 mV (Fig 3A) the calcium influx in response to a cold ramp was not blocked by the calcium channel blocker verapamil, and in fact application of verapamil was even seen to *enhance* the calcium influx. On release of the voltage clamp, a larger cold-induced calcium influx was observed, which can be attributed to activation of voltage-dependent calcium channels in the absence of verapamil, as in Fig 2C. In the inverse experiment under current clamp (Fig 3B), the calcium influx was suppressed by verapamil, identifying the origin of the majority of the calcium influx in this neuron as voltage-dependent calcium channels. In the same neuron, a smaller calcium influx was seen under voltage-clamp, consistent with Figs 2D and 3A. These results show a paradoxical double action of verapamil, which decreases the amplitude of the calcium signal activated by cold during current clamp (Fig 3B), when Ca_V channels are active, but *potentiates* cold responses during voltage clamp at −60 mV (Fig 3A), when Ca_V channel activity is suppressed.

Next, the effects of Ca_V channel antagonists on the cold-induced Ca^{2+} influx were determined (Fig EV2). The non-selective Ca^{2+} channel blockers Cd^{2+} and Gd^{3+} blocked all cold responses (Fig EV2A–D), but the Ca_V-selective antagonists verapamil, mibefradil, bepridil and nifedipine had only a small effect on mean cold-response amplitude (Fig EV2E–L). Closer examination of the results showed that this was due to different effects on individual neurons; in some neurons each of the different Ca_V channel blockers inhibited the response amplitude (black traces), while in others the response was potentiated (blue traces). These traces have been selected to show the extremes of the spectrum of sensitivities to calcium channel antagonists, and in most neurons the effects of calcium channel antagonists fell between these two extremes. These paradoxical results extend the data obtained under voltage and current clamp in Fig 3A–C and support the presence of two distinct calcium influx mechanisms activated by noxious cold: one that is activated when K2P channels are suppressed by cold, that is blocked by calcium channel antagonists; and a second novel Ca_V-independent calcium entry that is *enhanced* by calcium channel antagonists such as verapamil. A clue to the identity of the second calcium-entry mechanism comes from the recent discovery that activation of members of the ORAI calcium-selective ion channel family is enhanced by verapamil (Liu *et al*, 2019b).

The way in which these two mechanisms determine the cold temperature threshold in SCG neurons was investigated in Fig 3D. Cold activated a calcium increase at a mean of 10.8 ± 0.2°C in this experiment, which for unknown reasons is lower than the value of 13.1°C found in Fig 1H. However, following block of Ca_V channels by verapamil, the cold threshold was reduced significantly, to 9.4 ± 0.1°C (Fig 3D; $P < 0.0001$). The calcium entry component that is not blocked by verapamil was found to be blocked by the ORAI channel blocker YM58483/BTP2 (Zitt *et al*, 2004; Fig 3E and F), an effect that was particularly prominent in neurons showing a large increase in the cold-evoked calcium signal when exposed to verapamil (Fig 3G).

Similar experiments on cold-sensitive DRG neurons are shown in Fig 3H–J. The cold temperature threshold in DRG neurons not

Figure 3. Dissection of mechanisms underlying the novel cold-induced Ca^{2+}entry.

A Example of concurrent electrophysiological and Ca^{2+} imaging of a cold-sensitive SCG neuron in which the calcium channel blocker verapamil enhances the cold response under voltage clamp at −60 mV. Larger increase in $[Ca]_i$ seen in current-clamp mode (4th cold ramp). Similar results obtained in 4 SCG neurons.

B Example of cold-sensitive SCG neuron in which the calcium channel blocker verapamil inhibits cold response under current clamp. Smaller cold response seen under voltage-clamp (4th cold ramp). Similar results obtained in 5 SCG neurons.

C Mean cold-sensitive increase in calcium-dependent fluorescence ratio $F_{340/380}$ in SCG neurons is significantly smaller in neurons recorded in voltage-clamp at −60 mV (0.56 ± 0.08, mean ± SEM, $n = 21$ neurons) than in neurons recorded in current-clamp mode (1.16 ± 0.29, $n = 11$ neurons, $P = 0.0165$, unpaired *t*-test).

D Frequency distribution showing the effect of verapamil (100 μM, blue bars) on cold activation thresholds of SCG neurons. Mean cold threshold before (10.8 ± 0.2°C) and after (9.4 ± 0.1°C) addition of verapamil, $n = 301$ neurons, $P < 0.0001$.

E Representative calcium imaging trace showing block of cold responses with ORAI channel antagonist YM58483 (3 μM) in SCG neuron in the presence of Ca_V antagonist verapamil (100 μM). Temperature trace below.

F Summary of results of experiments on SCG neurons (mean ± SEM, $n = 115$ neurons, 4 separate cultures). YM58483 caused a significant decrease in cold-response amplitude ($P < 0.0001$, RM one-way ANOVA + Dunnett's test), with only partial recovery.

G In neurons with a small response to cold ($F_{340/380}$ increase <0.2; $n = 24$ neurons), many responses are potentiated by verapamil (31% of cold-insensitive neurons) and are fully blocked by ORAI channel antagonist YM58483 ($P < 0.0001$, RM one-way ANOVA + Dunnett's test).

H Similar histogram to (D) showing the effect of verapamil (100 μM, blue bars) on cold activation thresholds of novel cold-sensitive DRG neurons. Mean cold threshold without verapamil (15.1 ± 1.0°C, black bars, $n = 40$), and in the presence of verapamil (10.1 ± 1.6°C, blue bars, $n = 26$, $P = 0.0080$).

I Similar experiment to that shown in (E) but in DRG neurons. Black trace shows DRG neuron responding with a calcium increase to menthol and thus expressing TRPM8; cold-dependent calcium increase is partially blocked by verapamil (100 μM) but not by ORAI blocker YM58483. Blue trace shows neuron not responding to either menthol or AITC but activated by cold (novel cold-sensitive neuron) in which response is blocked by ORAI channel antagonist YM58483. Temperature trace below.

J Summary of results of experiments on novel cold-sensitive DRG neurons (mean ± SEM, $n = 28$ neurons, 3 separate cultures). YM58483 caused a significant decrease in cold-response amplitude ($P < 0.0001$, RM one-way ANOVA + Dunnett's test).

Source data are available online for this figure.

© 2022 The Authors

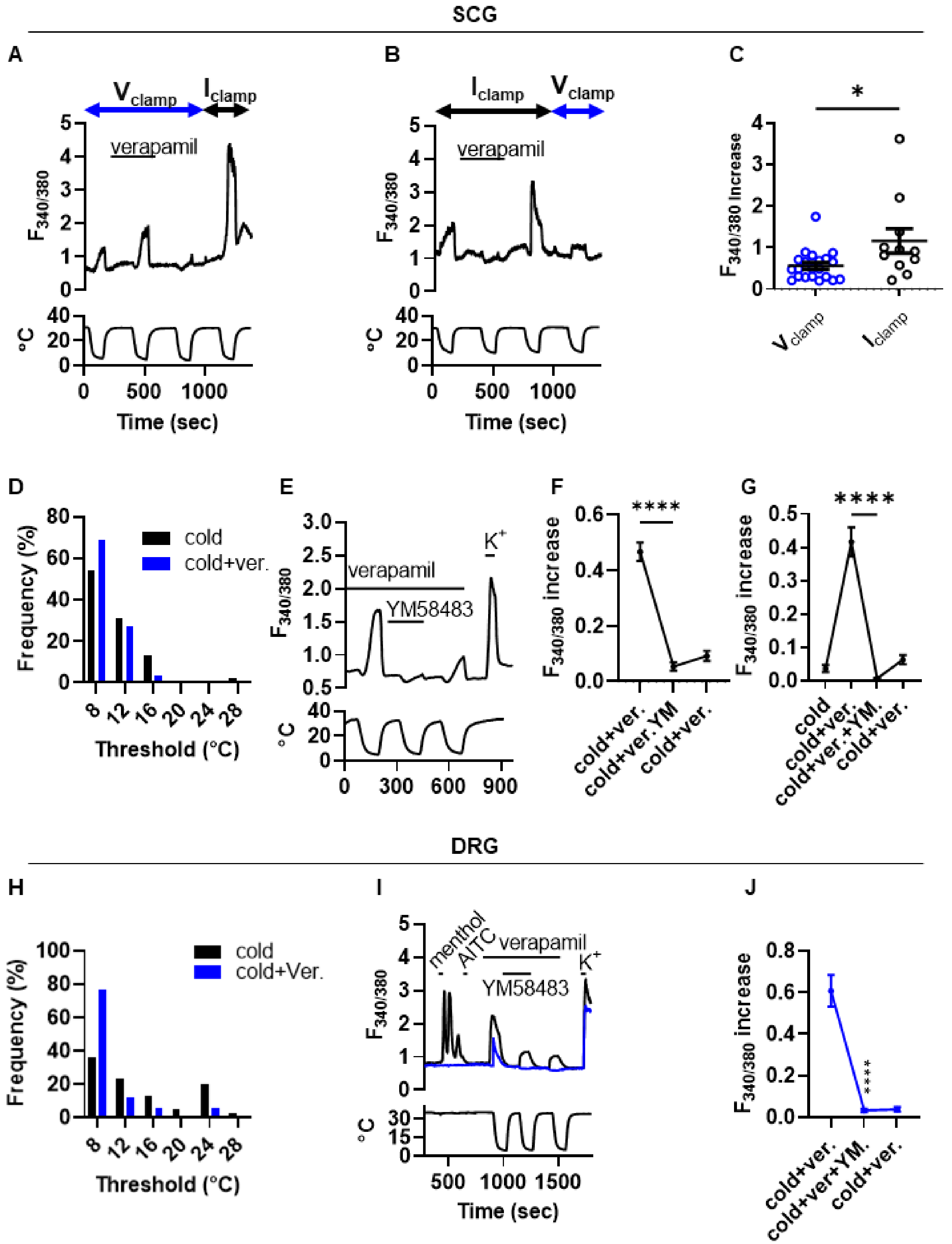
SCG
A
B
C
Vclamp
Iclamp
verapamil
F340/380
°C
Time (sec)
F340/380 Increase
*
D
E
F
G
cold
cold+ver.
Frequency (%)
Threshold (°C)
verapamil
YM58483
K+
****
F340/380 increase
cold+ver.YM
cold+ver.+YM.
DRG
H
I
J
cold+Ver.
menthol
AITC
cold+ver+YM.

Figure 3.

expressing TRPM8 ("novel cold-sensitive neurons") was 15.1 ± 1.0°C, identical to the value obtained in similar experiments in Fig 1D, while block of the Ca_V-dependent mechanism with verapamil reduced the threshold to 10.1 ± 1.6°C (significantly lower, $P = 0.008$, Fig 3H). In neurons whose cold response was attributable to TRPM8, as shown by a large increase in calcium on exposure to the TRPM8 agonist menthol, the ORAI channel blocker YM58483 did not block the cold response (Fig 3I), while in novel (TRPM8-independent) cold-sensitive neurons YM58483 completely suppressed the component of the cold response that is not blocked by verapamil (Fig 3J). The novel cold-induced response in DRG neurons exhibited some tachyphylaxis, but this did not explain the suppression of the cold response by YM58483 (Appendix Fig S4). Another structurally-different ORAI channel blocker, MRS1845, was also found to suppress the novel cold response in a similar way to YM58483 (Appendix Fig S5). In addition, ORAI channels are potentiated by elevated pH (Scrimgeour *et al*, 2012) which we also found to be a property of the novel cold-activated calcium entry (Appendix Fig S6).

In summary, these experiments show the existence of two cold-activated calcium entry mechanisms in both sympathetic and somatosensory neurons: one attributable to depolarization-dependent activation of Ca_V channels, that is blocked by verapamil; and a second whose activation is *enhanced* by verapamil but is suppressed by blockers of ORAI calcium channels. Both mechanisms are activated by cooling into the noxious cold range of temperatures, but the threshold for the Ca_V-dependent calcium entry is significantly less cold than the threshold for the Ca_V-independent calcium entry.

STIM1 and ORAI1 constitute a novel cold transduction mechanism

The above experiments suggest that a member of the ORAI ion channel family may be responsible for the novel cold-activated calcium entry but, as with all pharmacological studies, the results are open to the criticism that off-target block of an unrelated ion channel or channels may be responsible for the observed suppression of the cold-activated calcium influx by ORAI blockers. ORAI proteins form a highly Ca^2-selective, voltage-independent ion channel named I_{CRAC} (calcium release-activated current; Lopez *et al*, 2020). Discharge of intracellular calcium stores is sensed by STIM1 proteins located in the endoplasmic reticulum (ER) membrane, causing clustering of STIM1 into puncta and migration within the ER membrane to areas of close ER-plasma membrane apposition, where STIM1 puncta bind to and activate ORAI1, causing a calcium influx into the cytoplasm and thus enabling the refilling of subcellular calcium stores by ER-resident calcium pumps (Prakriya & Lewis, 2015; Qiu & Lewis, 2019). The membrane current associated with the calcium influx through ORAI channels is typically undetectable because of the high selectivity of the ORAI channel for calcium, and in this respect the ORAI channel matches the properties of the novel cold-activated and Ca_V-independent calcium entry determined above.

We expressed the three homologues of ORAI and two homologues of STIM (Hou *et al*, 2012) in pairs in HEK293 cells with a view to determining which STIM and ORAI homologues may be involved in generating the novel cold-activated calcium entry (see Fig 4A–C). We identified successfully transfected cells by co-transfection of mCherry, after verifying that mCherry expression is an excellent marker for the expression of all ORAI and STIM homologues (Fig EV3). The combination of STIM1 and ORAI1 was found to induce cold responses in transfected cells, but no other combinations of STIM and ORAI homologues produced a significant cold response (Fig 4A and B). The mean cold response threshold in STIM1/ORAI1 co-transfected cells was 18.6°C (Fig 4C), somewhat less cold but not dissimilar to that of the novel cold response in DRG neurons (15.1°C, Fig 1D) and SCG neurons (12.9°C, Fig 1H). Experiments performed with PC12 cells showed that expression of STIM1 and ORAI1 also enhances cold-activated calcium influx in this cell line (Appendix Fig S7). Additional evidence excluding ORAI3 as a contributor to the novel cold response was obtained with SCG neurons from ORAI3 KO mice, in which the amplitudes of the novel cold responses were not significantly different from those in SCG neurons from wildtype littermates (Appendix Fig S8).

We next used an RNA interference (RNAi) gene-silencing approach to knock down STIM1 proteins in SCG neurons (Fig 4D–G). Successful electroporation was indicated by expression of mCherry (# in left-hand panel of Fig 4D). Experiments were carried

Figure 4. STIM1 and ORAI1 underlie cold-induced Ca^{2+} entry.

A Ca^{2+} imaging traces showing the effect of cooling from 36°C to 6°C on HEK293 cells transfected with mCherry in combination with one STIM homologue and one ORAI homologue, or mCherry alone (control). Only the combination of STIM1 and ORAI1 gives a cold-induced calcium increase.

B Bar chart summarizing cold response amplitudes (mean ± SEM) of HEK293 cells successfully transfected with mCherry and STIM/ORAI homologues: STIM1 + ORAI1 ($n = 220$cells), STIM1 + ORAI2 ($n = 172$ cells), STIM1 + ORAI3 ($n = 200$ cells), STIM2 + ORAI1 ($n = 640$ cells), STIM2 + ORAI2 ($n = 251$ cells), STIM2 + ORAI3 ($n = 403$ cells), and control ($n = 303$ cells). Only the combination of STIM1 with ORAI1 induced cold responses that are statistically different from control ($P < 0.0001$, RM one-way ANOVA + Dunnett's test).

C Frequency histogram showing cold response thresholds of 166 HEK293 cells transfected with mCherry, STIM1, and ORAI1. Mean response threshold was 18.6 ± 0.6°C.

D Representative fluorescence images of SCG neurons from neonatal mice (age P5-7) electroporated with plasmids coding for mCherry and for siRNA targeting STIM1. Neurons were cooled from 35°C to 7°C in the presence of 100 μM verapamil. Electroporation was indicated by expression of mCherry (# in left-hand panel); in these neurons a cold response was absent (# in right-hand panel) while in neurons not expressing mCherry (*) cold responses were still observed. Scale bar 20 μm.

E Calcium imaging traces from neurons in D, showing the effect of cooling from 32°C to 6°C on SCG neurons electroporated with mCherry and siRNA targeting STIM1. Black trace from neuron not expressing mCherry (* in D), red trace from a successfully electroporated neuron that expressed mCherry (# in D). Temperature trace below.

F Cold response amplitudes ($F_{340/380}$, mean ± SEM). Successfully electroporated neurons (mCherry positive, red bar, $n = 111$ neurons from four separate cultures) had a significantly lower cold response amplitude than neurons not electroporated (mCherry negative, black bar, $n = 104$ neurons from same cultures, $P = 0.0082$, unpaired *t*-test).

G Percentage of neurons that responded to cold ramp with $F_{340/380}$ increase >0.2. Only 5% of mCherry-positive neurons (successfully electroporated, red bar) responded to cold, compared with 19% of mCherry-negative neurons in same cultures (non-electroporated, black bar, $P < 0.001$, chi-square test).

Source data are available online for this figure.

© 2022 The Authors

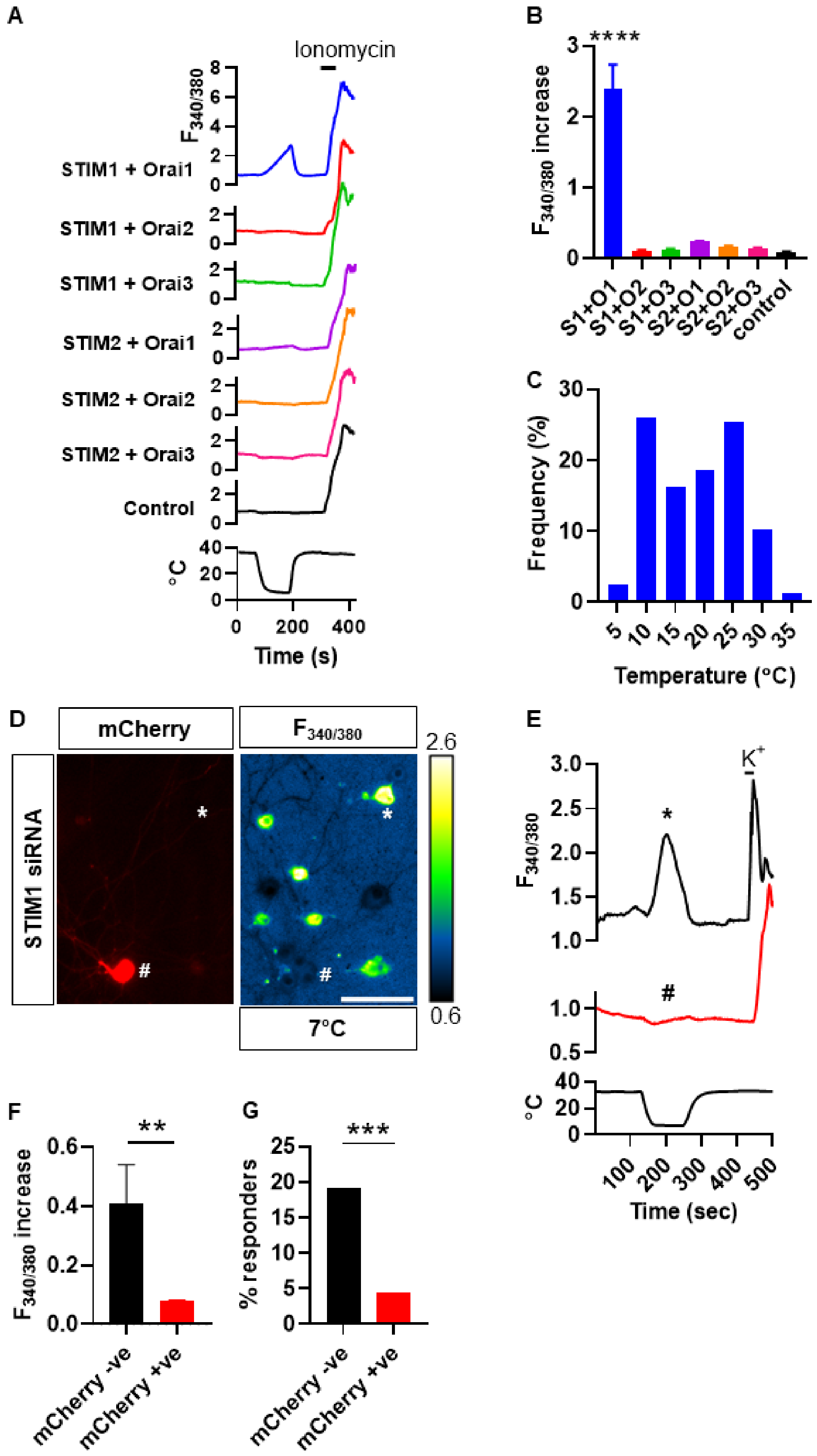
A
Ionomycin
F340/380
STIM1 + Orai1
STIM1 + Orai2
STIM1 + Orai3
STIM2 + Orai1
STIM2 + Orai2
STIM2 + Orai3
Control
°C
Time (s)
B
****
F340/380 increase
S1+O1
S1+O2
S1+O3
S2+O1
S2+O2
S2+O3
control
C
Frequency (%)
Temperature (°C)
D
mCherry
F340/380
STIM1 siRNA
7°C
2.6
0.6
E
K+
F340/380
°C
Time (sec)
F
**
F340/380 increase
mCherry -ve
mCherry +ve
G
***
% responders
mCherry -ve
mCherry +ve

Figure 4.

out in the presence of verapamil to suppress calcium influx due to activation of Ca_V channels, as above. In successfully electroporated neurons, identified by expression of mCherry, the cold response was significantly reduced (# in example shown in right-hand panel of Fig 4D and E), while in neurons in which electroporation had failed, as shown by the absence of expression of mCherry, cold responses were still observed (* in right-hand panel of Fig 4D and E). Successfully electroporated neurons had on average > 5 fold lower cold response amplitude than neurons not electroporated ($P < 0.01$, Fig 4F). Only 5% of successfully electroporated (mCherry positive) SCG neurons gave a detectable response to cold, compared with 19% of neurons not electroporated (mCherry negative, $P < 0.001$, Fig 4G). A control non-targeting siRNA (see Methods) did not significantly reduce the amplitude of the calcium increase in response to cold in successfully electroporated neurons when compared with neurons on the same coverslips that were not electroporated (Appendix Fig S9). These experiments show that expression of STIM1 is critical for novel cold responses in SCG neurons.

STIM1 and ORAI1 form puncta in response to cold

In order to evoke a surface membrane calcium influx in response to emptying of intracellular stores (a store-operated calcium influx, SOCE), STIM1 proteins located in the endoplasmic reticulum aggregate and migrate within the ER membrane to areas of close ER-plasma membrane apposition, where they interact with and open ORAI1 ion channels (reviewed in Prakriya & Lewis, 2015; Qiu & Lewis, 2019). To determine whether a similar process takes place in the case of the novel cold-induced calcium entry, we transfected fluorescently tagged STIM1-YFP and ORAI1-CFP into HEK293 cells and imaged the fluorescent proteins using total internal reflection fluorescence (TIRF) microscopy, which images juxta-membrane fluorescent STIM1 and ORAI1 to an imaging depth that had been set to c. 100 nm (Fig 5A; see Methods). The HEK293 cells were then cooled, with solution changing from 35°C to 13°C and back. We found that the application of a cold stimulus caused HEK293 cells to change shape, making comparison between images difficult, and that this could be minimized by removal of calcium (nominal zero Ca) from the extracellular solution. A second complication was that the fluorescence of STIM1-YFP and ORAI1-CFP was found to be temperature-dependent (STIM1-YFP less so than ORAI1-CFP, see Appendix Fig S10) and the intensities of the images in cold solution shown in Fig 5A have therefore been adjusted for this.

Representative images before, during and after cooling from 35°C to 13°C show that ORAI1 forms puncta in response to cooling, and that these puncta largely disappear on rewarming (Fig 5A, examples are visible most prominently in the centre of the field of view and are marked by *). The numbers of STIM1 and ORAI1 puncta per cell varied considerably, partly because of variations in cell size; the cell shown in Fig 5A has a lower number of both STIM1 and ORAI1 puncta visible than was seen in many other cells, and this cell was chosen because the change in ORAI1 puncta number and colocalization with STIM1 on cooling is best seen when numbers are smaller. A similar reversible increase in Orai1 puncta number on cooling is shown in a cell with a larger number of puncta in Fig EV4. While the magnitudes of the increases in ORAI1 puncta number on cooling were variable in different cells (see data from individual cells in Fig 5B), the averages from all cells showed a highly significant increase ($P < 0.01$, blue line in Fig 5B), followed by reversal on rewarming. The area occupied by ORAI1 puncta was similarly variable (Fig 5C), but the mean increase caused by cooling was also highly significant ($P < 0.0001$).

STIM1 was mostly visible as preformed puncta before cooling (Fig 5A), probably owing to the omission of calcium from the bathing solution and consequent partial store emptying (see above). The videos in Movie EV1 compare STIM1 localization in normal calcium (left-hand video) and in nominal zero calcium (right-hand video). The majority of STIM1 in normal calcium is highly mobile and is visible in long tube-like structures, presumably ER, but some is localized into transient aggregations that resemble puncta. In nominal zero calcium, some STIM1 is visible in tube-like structures, but most STIM1 is in the form of puncta, some of which are fixed, and some mobile or transient. In the example shown in Fig 5A, cooling appears to cause a reduction in the number of STIM1 puncta, but this change was not found to be significant in averaged data from a number of cells ($P > 0.05$, Fig 5D and E). There is, however, a relocation of STIM1 to the cell surface caused by cooling, as seen in the edge-on view of the cell membrane (arrow in Fig 5A, lower panels). The co-localization between STIM1 and ORAI1 was also significantly increased by cooling ($P < 0.0001$, Fig 5F). In each case, the changes seen in Fig 5 were reversed on rewarming (Fig 5D–F). These data are consistent with STIM1, partly preformed into puncta by bathing in low calcium, binding to and capturing ORAI1 ion channels and, by the data shown previously, activating ORAI1 and inducing a calcium influx.

Figure 5. The effect of cold on ORAI1 puncta formation and colocalization of ORAI1 and STIM1 in HEK293 cells.

A Representative TIRF microscopy images (imaging depth c. 100 nm) showing that cooling from 35°C to 13°C increases ORAI1 puncta formation and colocalization of ORAI1 with STIM1 in HEK293 cells transfected with ORAI1-CFP and STIM1-YFP. Cooling to 13°C causes the appearance of new ORAI1 puncta (examples shown by *) that are not present in the 35°C image and are colocalized with STIM1. Cooling also causes STIM1 to move towards the cell surface in edge-on views (arrows). To reduce cell motility, the experiment was conducted in nominal $0[Ca]_e$, which caused partial formation of STIM1 puncta before the images shown. Scale bar = 5 μm. Temperature trace shown below. Mean fluorescence intensity adjusted to compensate for increase in intrinsic fluorescence of CFP and YFP caused by cooling (see Appendix Fig S10).

B–F Collected measurements (see Methods for details) of ORAI1 puncta number; relative area occupied by ORAI1 puncta; and colocalization of STIM1 and ORAI1 in HEK293 cells before, during, and after cooling (mean ± SEM, $**P = 0.0015$ $****P < 0.0001$, RM one-way ANOVA + Dunnett's test, $n = 44$ cells in 8 wells). Individual results shown in light grey in B, C and F. Cooling significantly increases the number of ORAI1 puncta per cell (B, D) and the percentage area of the cell membrane surface covered by ORAI1 puncta (C, E). Cooling causes an apparent small decrease in number of STIM1 puncta per cell (D) and the percentage area of the cell covered by STIM1 puncta (E) though both were non-significant ($P = 0.22$ and $P = 0.10$, respectively). Cooling significantly increased colocalization of STIM1 and ORAI1 (F, $P < 0.0001$, RM one-way ANOVA + Dunnett's test).

Source data are available online for this figure.

© 2022 The Authors

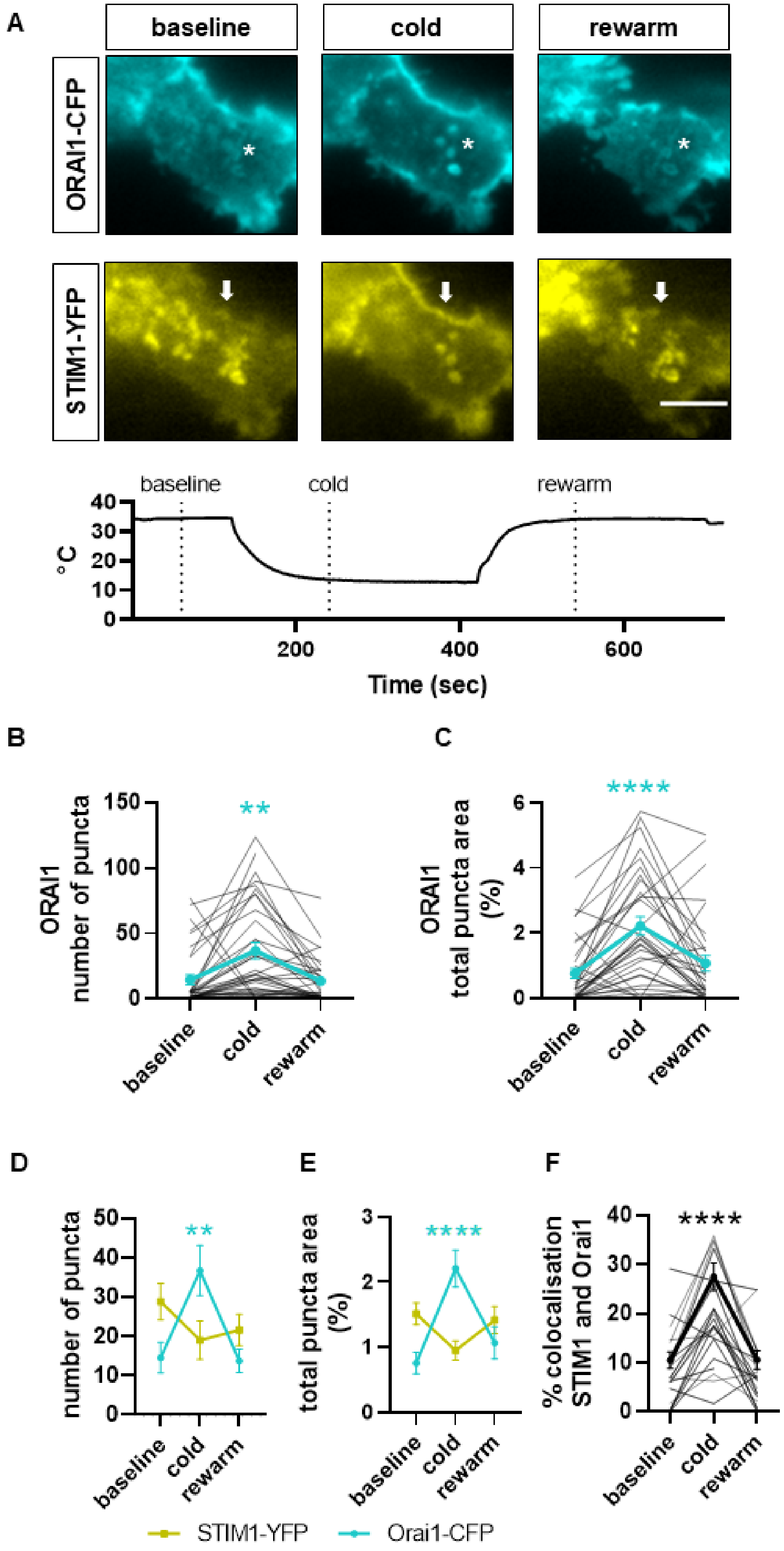
A
baseline
cold
rewarm
ORAI1-CFP
STIM1-YFP
baseline
cold
rewarm
°C
Time (sec)
B
ORAI1
number of puncta
**
C
ORAI1
total puncta area
(%)
****
D
number of puncta
**
E
total puncta area
(%)
****
F
% colocalisation
STIM1 and Orai1
****
STIM1-YFP
Orai1-CFP

Figure 5.

Exposure to cold does not discharge intracellular calcium stores

The effects of cooling on the cellular distribution of ORAI1, as shown in Fig 5, are similar to those observed in other studies when calcium stores are emptied and store-operated calcium entry is evoked at room temperature (Gwozdz *et al*, 2008; McNally *et al*, 2013). One important difference, though, is that cold does not appear to discharge intracellular stores (see Fig 2A), suggesting that cold is able to activate STIM1 and promote calcium entry through ORAI1 channels independently of calcium discharge from intracellular stores.

The experiments shown in Fig EV5 further investigate whether cold discharges calcium stores, by comparing the effect of cold on store-operated calcium entry (SOCE) with that of thapsigargin, which potently evokes SOCE by inhibiting the sarcoplasmic and endoplasmic calcium ATPase (SERCA) responsible for store filling, and therefore empties intracellular calcium stores (Lytton *et al*, 1991). Removal of extracellular calcium in Fig EV5A caused a steady decrease in intracellular calcium, as in Fig 2A. Application of 4°C cold caused no detectable calcium release from intracellular stores, because the only effect of cold on $F_{340/380}$ was a small downward deflection attributable to a direct effect of cold on the fura-2 calcium indicator (Appendix Fig S2). On return to normal extracellular calcium, there was a small overshoot of intracellular calcium, attributable to a small amount of store depletion by the 0Ca exposure and consequent partial activation of SOCE, but an identical small overshoot was also seen after the same period in 0Ca without a cold stimulus (Fig EV5B, and summary data in Fig EV5D), showing that the exposure to cold did not by itself deplete calcium stores. Application of the SERCA blocker thapsigargin also did not cause a detectable increase in intracellular calcium while in 0Ca (Fig EV5A and D), which may be due to the sparse ER in neurons (Gruszczynska-Biegala *et al*, 2016). However, thapsigargin had in fact caused a calcium release from intracellular stores, because following return to normal calcium a significantly increased SOCE was observed (Fig EV5A, and summary data in Fig EV5D). The failure of thapsigargin to cause a visible calcium release from intracellular calcium stores was not due to the length of time in zero external calcium and consequent passive store discharge, because after an even briefer exposure to zero calcium, thapsigargin did not cause a visible calcium release (Fig EV5C and D). This experiment shows that calcium release by cold in SCG neurons is negligible compared with the release caused by the SERCA inhibitor thapsigargin, in support of the proposal that cold directly activates STIM1, and thus causes a calcium influx through ORAI1 channels, without first invoking a discharge of calcium from intracellular stores.

Peripheral sympathetic neurons express nNOS

A possible downstream target for the calcium increase caused by activation of ORAI1 channels by strong cold is suggested by the widespread expression of neuronal nitric oxide synthase (nNOS) in sympathetic neurons (see Fig 6A and B), for instance in the neurons of the superior cervical ganglion (SCG), that innervates targets including the skin of the head and neck, and the sympathetic stellate ganglion (SG) that innervates targets in the chest and skin of the forelimbs. Increases in intracellular calcium are known to activate nNOS (Förstermann & Sessa, 2012), so the cold-induced calcium increase could activate nNOS, releasing nitric oxide and thus causing cold-induced vasodilation (CIVD), an emergency response to cold temperatures that acts to preserve peripheral tissues from frostbite, and for which no mechanism has been found to date.

Fig 6B shows partial co-expression between nNOS and tyrosine hydroxylase (TH), an essential biosynthetic enzyme for noradrenaline (NA), which acts on α-adrenoceptors to cause vasoconstriction. In both the SCG and the SG, most neuronal cell bodies express both nNOS and TH to at least some extent, though expression of nNOS is greater in some neurons and expression of TH is stronger in others. Co-expression of nNOS and TH may seem contradictory because cold-induced firing of efferent action potentials in sympathetic nerves will cause an increase in calcium, directly promoting exocytosis of vesicles containing NA and thus triggering vasoconstriction, while local activation by cold of the ORAI-dependent calcium increase characterized in this article will also increase calcium, activate nNOS and thus potentially cause vasodilation. It is possible that different patterns of calcium elevation—pulsatile in the case of influx through Ca_V ion channels, maintained in the case of influx through Orai1 ion channels—may activate the TH and nNOS pathways differentially to cause vasoconstriction or vasodilation as required (discussed further below).

Discussion

The molecular mechanisms by which noxious heat, non-noxious warmth and non-noxious coolness are detected in peripheral sensory neurons have each been shown to involve activation of different thermally sensitive members of the large TRP ion channel family. The ability to detect more extreme cold is also vital for the survival of organisms, in order to avoid life-threatening thermal loss and tissue damage from frostbite, but the molecular mechanisms by which this essential sensory function is carried out remain mysterious. TRPA1 and TRPC5 have been suggested as extreme cold sensors, but this has not been confirmed by most subsequent work (reviewed in Buijs & McNaughton, 2020; Talavera *et al*, 2020), and

Figure 6. Neuronal Nitric Oxide Synthase (nNOS) is widely expressed in postganglionic sympathetic neurons.

A Representative immunofluorescence images showing adult mouse stellate ganglion (SG) sections stained for nNOS (red), to label nitrergic neurons, βIII Tubulin (green) to label all neurons, and DAPI to label nuclei. nNOS is widely expressed in neurons of the stellate ganglion, but is absent from glial cells (glial nuclei identifiable in merged image, similar results in $n = 5$ animals). Antibody against nNOS from Abcam; similar results obtained with antibodies from ECM and Millipore. All three antibodies show nNOS widely expressed in SG cell bodies and neurites.

B Adult mouse SCG ($n = 2$ animals, upper) and SG ($n = 5$ animals, lower) sections stained for nNOS (red), tyrosine hydroxylase (TH, green) and DAPI (blue) to label nitrergic neurons, noradrenergic neurons and nuclei, respectively. Many noradrenergic cells also express nNOS (yellow cells in merged image). Scale bars 50 µm.

Source data are available online for this figure.

▶

© 2022 The Authors

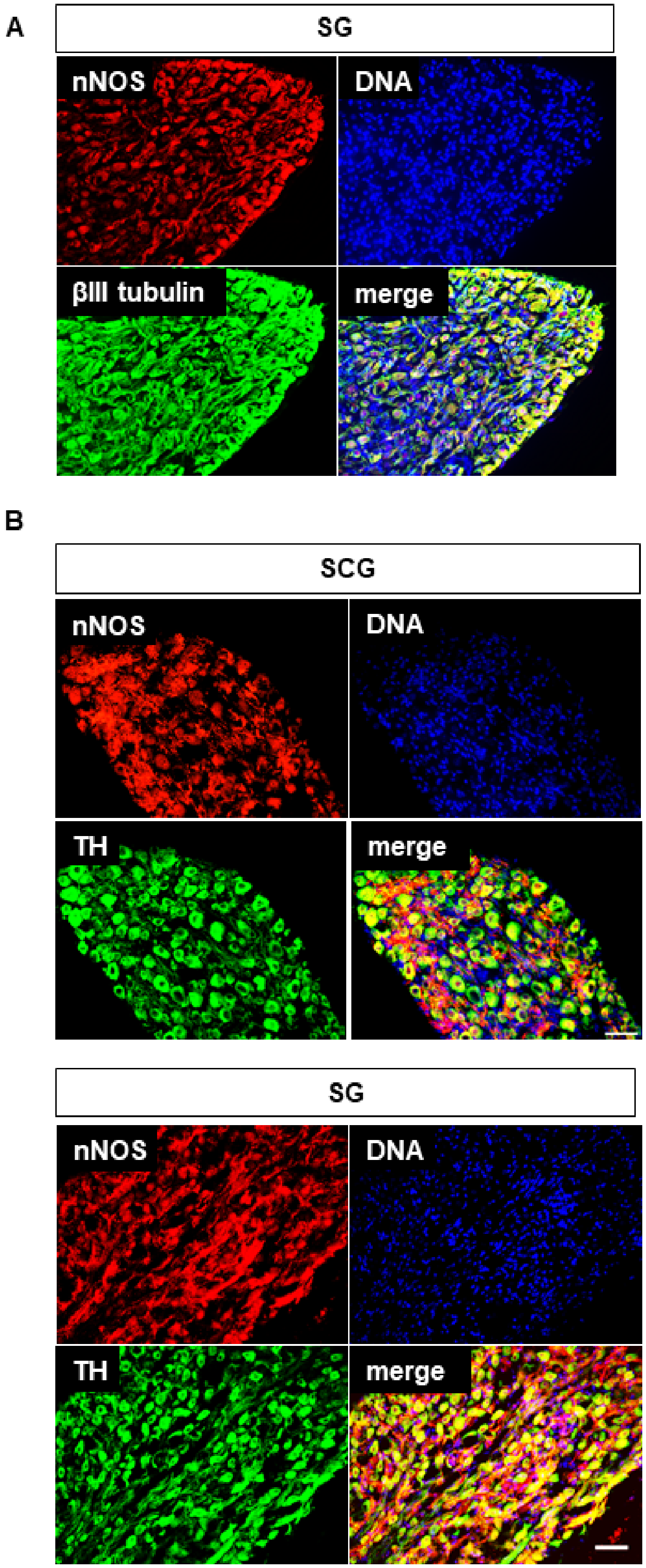
A
SG
nNOS
DNA
βIII tubulin
merge
B
SCG
nNOS
DNA
TH
merge
SG
nNOS
DNA
TH
merge

Figure 6.

in agreement, we show here that expression of TRPA1 and TRPC5 is poorly correlated with cold sensitivity in DRG neurons.

An alternative mechanism for the detection of cold, not involving TRP channels, arises from the finding that the activity of TREK and TRAAK channels, members of the two-pore potassium channel family, is suppressed by cold, and that the consequent membrane depolarization can initiate the firing of action potentials and a calcium influx into a neuron via voltage-dependent calcium (Ca_V) ion channels (Lesage *et al*, 2000; Viana *et al*, 2002; Noël *et al*, 2009; Pereira *et al*, 2014). In the present study, we confirm that this mechanism plays an important role in the cold sensitivity of both somatosensory and sympathetic neurons. We find, however, that an additional mechanism for cold-induced calcium influx, through highly calcium-selective ORAI1 channels, is also important.

We initially confirmed earlier findings that a fraction of DRG neurons (around 14%) respond to a strong cold stimulus with an elevation of intracellular calcium, but that roughly one-third of these do not express any of the potential cold-sensors TRPM8, TRPC5 or TRPA1 (Story *et al*, 2003; Babes *et al*, 2006; Bautista *et al*, 2007; Munns *et al*, 2007; Noël *et al*, 2009). We also found that more than one third of sympathetic neurons, which are usually thought of as motor rather than sensory, sense cold stimuli and respond with an elevation of calcium, and that the large majority of these do not express any of TRPM8, TRPC5 or TRPA1 (see also Babes *et al*, 2004; Smith *et al*, 2004; Munns *et al*, 2007; Ran *et al*, 2016). These observations show that mechanisms other than these three TRP channels underlie cold responses in many DRG and sympathetic neurons. The calcium increase in response to cold was in each case completely abolished by removal of extracellular calcium, and so is due to a calcium influx rather than to a release from internal stores.

In patch clamp and calcium imaging experiments, we confirmed that part of the calcium entry was attributable to a cold-activated neuronal excitability initiated by the suppression of a background outward current, likely mediated by a member of the K2P ion channel family, as reported previously (Lesage *et al*, 2000; Viana *et al*, 2002; Noël *et al*, 2009; Pereira *et al*, 2014). This calcium influx can be suppressed either by voltage-clamp at the resting potential, to prevent activation of Ca_V channels, or by pharmacological block of Ca_V channels. When activation of Ca_V channels was prevented, a second novel cold-activated calcium influx mechanism was observed, that differed from the voltage-dependent calcium influx in several ways: it was unaffected by voltage-clamp at the resting membrane potential; it was not associated with a detectable inward current; it was enhanced, rather than being blocked, by Ca_V ion channel blockers such as verapamil; and it was inhibited by selective blockers of ORAI channels. The two calcium-entry mechanisms were expressed to varying degrees in different neurons: some neurons expressed only the Ca_V-dependent mechanism, some only the novel (ORAI-dependent) cold-activated calcium mechanism, while most neurons expressed both mechanisms to a variable extent. This article focusses mainly on elucidating cold-activation of the novel ORAI1-dependent calcium influx mechanism, which has not previously been described in peripheral neurons.

Members of the SOCE family of proteins (STIM1 and 2; ORAI1–3) are expressed in DRG neurons (Lirk *et al*, 2008; Park & Luo, 2010; Usoskin *et al*, 2015; Hogea *et al*, 2021), mainly in small and medium-sized neurons, the majority of which are nociceptors (Wei *et al*, 2017). To determine which STIM and ORAI homologues participate in the ORAI-dependent calcium influx mechanism, we tested the effects on cold-activated calcium influx of overexpression of all combinations of STIM and ORAI proteins, and we found that only the expression of STIM1 and ORAI1 together were able to recapitulate a cold-activated calcium influx similar to that seen in peripheral neurons. In confirmation, selective knockdown of STIM1 in SCG neurons was found to abolish cold-sensitivity. These experiments point to STIM1 and ORAI1 as the critical homologues mediating the calcium influx in response to extreme cold in sensory and sympathetic neurons.

Finally, TIRF imaging showed that in response to cooling, STIM1 migrates towards the surface membrane, STIM1 and ORAI1 colocalize, and as a result ORAI1 channels in the surface membrane are opened, as also occurs when SOCE is induced following the discharge of intracellular stores. A difference between the well-known store-operated mechanism and the cold-activation of ORAI1, however, is that colocalization of STIM1 and ORAI1 and triggering of a calcium influx through ORAI1 ion channels in response to cold occurs without emptying of intracellular calcium stores. These observations show that STIM1, in addition to its well-known function as a sensor of the calcium levels in intracellular calcium stores, is also able to sense cold and trigger activation of ORAI1 channels without a change in ER calcium levels.

Our results also show that ORAI1 calcium channels are cold-activated by STIM1 only in subsets of somatosensory and sympathetic neurons, a surprising finding because STIM and ORAI proteins are expressed ubiquitously (Uhlen *et al*, 2015). Splice-variants of STIM1 have been shown to be cell-type specific (Nelson & Roe, 2018), but this seems unlikely to explain the presence of cold-sensitivity in specific populations of neurons, because we found that expression of the most common mouse variant of STIM1, in combination with human ORAI1, rendered HEK293 and PC12 cells cold-sensitive. The expression of cold-sensitivity must be regulated in some way, therefore, to confer cold-sensitivity in only a fraction of somatosensory or sympathetic neurons.

Calcium influx through ORAI channels, which are highly calcium-selective, typically does not produce enough inward current to be detectable against other background currents when activated by store-emptying (Derler *et al*, 2013). In agreement, entry of calcium mediated by cold-induced STIM1-ORAI1 activation was also not found in the present study to be associated with any detectable inward current. The cold-induced STIM1-ORAI1 mechanism demonstrated here is therefore unlikely to directly generate cold-activated firing of action potentials, that could then propagate via the axons of sensory neurons to generate a conscious sensation of cold, unlike activation of the non-selective cationic ion channel TRPM8 or inhibition of TREK/TRAAK potassium channels, both of which cause firing of action potentials in cold-sensitive nerve fibres. The STIM1-ORAI1 mechanism is therefore likely to operate at a local level only, and not to initiate a sensation propagating to consciousness.

The diagram in Fig 7 summarizes the three mechanisms that can modulate nerve activity and intracellular calcium in response to a non-noxious cool or a noxious cold stimulus, as observed in the present study.

Somatosensory neurons

(i) Non-noxious coolness (mean threshold around 23°C, see Fig 1D; top left in Fig 7) directly activates TRPM8 ion channels expressed in

 © 2022 The Authors

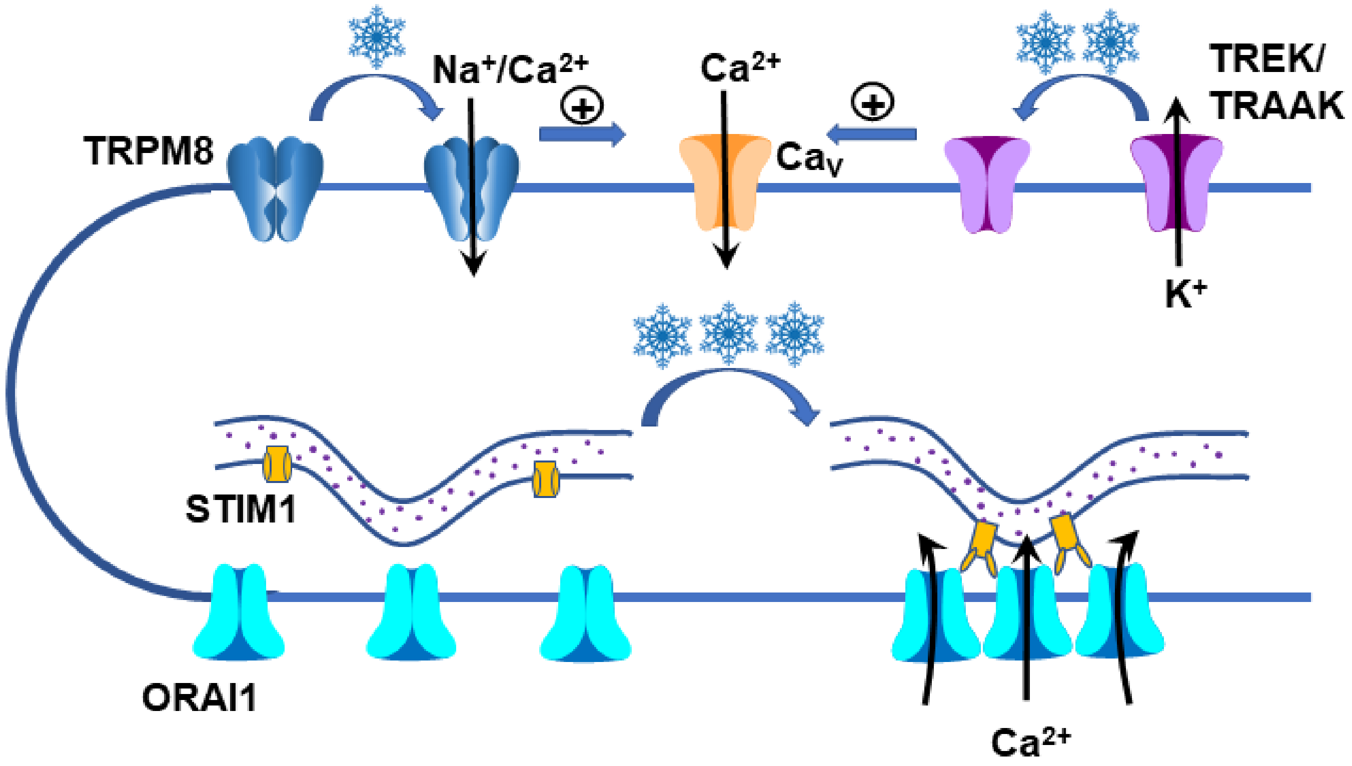

Figure 7. Mechanisms activated by mild and noxious cold in somatosensory and sympathetic neurons.

Top left: Mild non-noxious coolness, represented by a single snowflake (mean threshold 23°C, see Fig 1D) activates TRPM8 ion channels (dark blue) that are permeable to Na^{2+} and Ca^{2+}. The consequent depolarization and calcium influx is amplified by the activation (+) of voltage-dependent calcium channels (Ca_V, orange). The TRPM8 mechanism is present in a small fraction of DRG neurons (5%, Fig EV1A) but is absent from SCG neurons (Fig EV1E). *Top right*: Stronger cold, represented by two snowflakes (mean threshold 13–15°C in DRG and SCG neurons, see Figs 1D and H, and 3H) deactivates potassium channels (lilac) belonging to the TREK/TRAAK two-pore potassium (K2P) channel family, causing depolarization, activation of voltage-dependent calcium channels (orange) and a consequent calcium influx (see Fig 2E–H). This mechanism is present in a subset of both DRG neurons and SCG neurons. *Bottom*: Extreme cold, represented by three snowflakes (mean threshold 9–10°C, see Fig 3D and H) causes movement of the ER-resident membrane protein STIM1 (yellow) to preformed locations adjacent to the surface membrane, triggering aggregation of surface-membrane ORAI1 channels (light blue) into puncta. Consequent opening of the highly calcium-selective ORAI1 channels causes a calcium influx without detectable membrane depolarization. The STIM1-ORAI1 mechanism is triggered by cold without the need for calcium (purple dots) to be emptied from intracellular stores. This mechanism is present in a subset of DRG and SCG neurons, with some but not complete overlap with the TREK/TRAAK mechanism. TRPA1 and TRPC5 ion channels are present in some neurons responsive to cold (Fig EV1) but are also expressed in many neurons that are not cold-responsive (Figs 1 and EV1) so their status as cold-activated sensory ion channels in neurons is doubtful.

a small subset of sensory neurons, causing an influx of Ca^{2+} and Na^{+} ions. The consequent depolarization generates a further calcium influx by activating voltage-dependent Ca_V channels. (ii) Noxious cold (mean threshold around 15°C, see Fig 1D; top right in Fig 7) suppresses potassium channels belonging to the TREK and TRAAK family, again causing depolarization and activating voltage-dependent Ca_V channels. (iii) Finally, a third mechanism (bottom in Fig 7), whose properties were studied in more detail in sympathetic neurons, is activated by extreme noxious cold (mean threshold around 10°C, see Fig 3H); cold activates STIM1, causing aggregation of surface-membrane ORAI1 ion channels into puncta, without the need for emptying of subcellular calcium stores. ORAI1 channels then open to generate a calcium influx. A possible explanation may be that extreme cold causes a change in STIM1 structure that allows it to trigger ORAI1 aggregation in a way that is not possible at normal temperatures.

Sympathetic neurons

The TRPM8 mechanism is not present in sympathetic neurons, but around 40% of SCG neurons express a novel (non-TRP dependent) cold-sensing mechanism, which was a surprise because sympathetic neurons are commonly thought of as purely motor. As in somatosensory neurons, noxious cold (threshold around 13°C, see Fig 1H) triggers closure of K2P channels and thus activates a calcium influx through Ca_V channels (top right in Fig 7). The STIM1-ORAI1 mechanism (botom in Fig 7) is also prominent in sympathetic neurons, with a lower temperature threshold of around 10°C (Fig 3D). The expression of these two mechanisms overlaps in cold-sensitive sympathetic neurons, with some neurons dominated by one or the other, but most expressing a mixture of the two. We discuss below possible reasons for the presence of two mechanisms that can sense noxious cold in sympathetic neurons.

© 2022 The Authors

Apart from the three mechanisms discussed above, other possibilities have been suggested in past work, and while not supported by the present data, are also not completely excluded. (i) TRPA1 and TRPC5 ion channels are present in some somatosensory and sympathetic neurons that are responsive to cold, but are also expressed in many neurons that are not cold-responsive, so their status as cold-activated sensory ion channels in neurons is doubtful. (ii) A recent report identifies the glutamate receptor GLR-3 (mouse homologue GluK2) as a cold sensor in *C. elegans* and mouse (Gong *et al*, 2019). While this possibility was not specifically investigated here, we note that all the cold-activated calcium influx into somatosensory and sympathetic neurons is accounted for by the three mechanisms outlined above.

To the best of our knowledge, a cold-dependent activation of STIM1, leading to opening of surface membrane ORAI1 ion channels and a consequent calcium influx, as reported here, has not been observed before in peripheral neurons, though phenomena that are likely to be related have been observed in other cell types. In STIM1-transfected cell lines a calcium influx is activated following the removal of a heat stimulus (Xiao *et al*, 2011), which may be related to the cold-activated calcium entry described in this article. A similar STIM1-dependent calcium influx in response to removal of a heat stimulus was observed in keratinocytes, and was shown, like the calcium entry reported in this article, not to be dependent on store-emptying (Liu *et al*, 2019a). A study on keratinocytes found a calcium release from intracellular stores, by an undetermined mechanism, in response to a mild cold stimulus (Sadler *et al*, 2020). Both of the most recent studies showed that altering the calcium responses of keratinocytes to thermal stimuli was able to modulate thermal preference *in vivo*, raising the possibility that calcium signals originating in keratinocytes might be transmitted to sensory neurons, for instance by the release of ATP (Sadler *et al*, 2020). In this article, however, the calcium signals that we observe in neurons are not transmitted from other cell types that may be present in the cultures, because they are present in isolated neurons, are inhibited in neurons by ORAI-specific blockers and are prevented by downregulating STIM1 expression in neurons. We also did not observe calcium signals in response to cold stimuli in any non-neuronal cells present in our cultures.

In somatosensory nerves, ORAI channels may be involved in nociception, because they have been found to increase the excitability of C- and A-fibres (Wei *et al*, 2017) and oral administration of the ORAI antagonist YM58483 in mice produces analgesia and prevents the development of chronic pain (Gao *et al*, 2013; Qi *et al*, 2016).

In sympathetic nerves projecting to the skin, which mainly innervate blood vessels, what might be the consequence of the Ca^{2+} entry induced by noxious cold? A dual response of cutaneous blood vessels defends mammals against cold: mild cold causes vasoconstriction, in order to conserve central heat, but when the skin is strongly cooled, blood vessels dilate in an attempt to protect tissue from damage through frostbite, a phenomenon known as cold-induced vasodilation (CIVD; Daanen & Van Der Struijs, 2005). Peripheral vasoconstriction in response to mild cold temperatures is thought to be triggered by TRPM8-expressing somatosensory neurons, that via a central reflex arc initiate the firing of action potentials in sympathetic axons, releasing the sympathetic vasoconstrictor noradrenaline from sympathetic nerve terminals (Tan & Knight, 2018). More extreme cold, on the other hand, requires a vasodilator response that is local rather than reflex, because cold inhibits nerve conduction velocity by 15 m/s per 10°C until full block is achieved at noxious cold temperatures of around 8–10°C (Franz & Iggo, 1968; Vanggaard, 1975). Importantly, CIVD is gradually lost after sympathectomy (Lewis, 1930), showing that it is a local cold response mechanism that does not require intact sympathetic input from the CNS but does require sympathetic nerve endings to be functional.

The expression of nNOS in sympathetic neurons shows that they have the potential ability to release nitric oxide and thereby cause neurogenic vasodilation (see Fig 6 and Taylor *et al*, 1993). Neuronal NOS is a Ca^{2+}/calmodulin-dependent protein (Förstermann & Sessa, 2012) that could potentially be activated by a cold-induced Ca^{2+} influx through ORAI1 channels. Sustained NOS activation in endothelial cells has been shown to require ORAI1 (Kwan *et al*, 2000; Lin *et al*, 2000). We propose, therefore, that activation of STIM1 by noxious cold, leading to the opening of ORAI1 channels, to a calcium influx into sympathetic nerve terminals and consequent release of NO by nNOS, may be the elusive mechanism underlying the cold-induced vasodilation that helps to protect peripheral tissues from damage by extreme cold.

Materials and Methods

Mice

All experimental protocols were conducted in accordance with the Guide for Care and Use of Laboratory Animals (Institute of Laboratory Animal Research, 2011) and the ARRIVE guidelines. C57BL/6J mice were used for all experiments and were maintained on a 12 h light/dark cycle. ORAI3$^{-/-}$ mice were a kind gift from Prof. M. Trebak.

Primary neuron culture

Adult C57BL/6J mice were euthanized by cervical dislocation, DRG or SCG ganglia were excised and incubated in papain (2 mg/ml in Ca^{2+}- and Mg^{2+}-free HBSS) for 30 min at 37 °C, followed by incubation in collagenase (2.5 mg/ml in Ca^{2+}- and Mg^{2+}-free HBSS) for 30 min at 37°C. Ganglia were re-suspended and mechanically dissociated in culture medium containing Neurobasal-A, supplemented with 0.25% (v/v) L-glutamine 200 mM (Invitrogen), 2% (v/v) B-27 supplement (Invitrogen), 1% (v/v) penicillin/streptomycin (Invitrogen) and nerve growth factor (NGF; Sigma-Aldrich) at 50 ng/ml. Dissociated neurons were centrifuged and plated onto coverslips pre-coated with poly-L-lysine (10 µg/ml) and laminin (40 µg/ml). Neurons were kept in an incubator at 37°C and 5% CO_2 for 18–24 h before use.

HEK293 cell culture

Human Embryonic Kidney cells (HEK293, ATCC) were thawed from liquid nitrogen storage in a 37°C water bath, centrifuged and resuspended in Dulbecco's Modified Eagle Medium (DMEM with L-glutamine, 1000 mg/l D-glucose and sodium pyruvate, Invitrogen, supplemented with 10% v/v foetal bovine serum, FBS, Invitrogen, plus 1% penicillin/streptomycin, Thermo Fisher Scientific),

© 2022 The Authors

mechanically dissociated and cultured in cell culture flasks (Fisher Scientific) coated with 40 ng/ml laminin (Fisher Scientific) in 0.01% w/v Poly-L-Lysine solution (Sigma). When grown to 90% confluency, HEK293 cells were detached from the flasks using 0.05% trypsin (Thermo Fisher Scientific), centrifuged, resuspended in DMEM, and mechanically dissociated. Cells were then seeded on pre-coated coverslips and kept in an incubator at 37°C and 5% CO_2 for 18–36 h before use, by which time they had attained 50–80% confluency.

PC12 cell culture

Rat adrenal pheochromocytoma 12 (PC12) cells in 10% DMSO (Greene & Tischler, 1976) were thawed from liquid nitrogen storage in a 37°C water bath, centrifuged, and resuspended in RPMI-1640 medium (Sigma-Aldrich) supplemented with 1% (v/v) penicillin–streptomycin (Invitrogen), 1% (v/v) L-glutamine (200 mM; Invitrogen), and 10% (v/v) horse serum (Invitrogen), mechanically dissociated, and plated on 25cm^2 cell culture flasks (Fisher Scientific) previously coated for 1 h with 1 mg/ml Collagen IV from human placenta (Sigma) dissolved in acetic acid and diluted 10× in 0.01% w/v Poly-L-Lysine solution (Sigma), followed by washing with PBS (Sigma). When grown to 90% confluency, PC12 cells were detached from the flasks using 0.05% trypsin (Thermo Fisher Scientific), centrifuged, resuspended in RPMI, and mechanically dissociated. Cells were then seeded on pre-coated coverslips. Cells were kept in an incubator at 37°C and 5% CO_2 for 18–36 h before transfection, by which time they had attained 50–80% confluency.

Transfection of plasmid DNA using Lipofectamine LTX reagent

Bacterial stabs containing plasmids with ORAI1, ORAI1-CFP, ORAI2, ORAI3, STIM1, STIM1-YFP, STIM2, and mCherry (Table 1) were amplified using a HiSpeed Plasmid Midi Kit (Qiagen) according to the manufacturer's instructions.

Plasmid DNA was transfected into cells using Lipofectamine LTX Reagent (ThermoFisher Scientific) as per the manufacturer's instructions. Briefly, 0.05 μg/ml of total DNA (with STIM, ORAI, and mCherry in 2:1:1 ratio) was dissolved in Opti-MEM Reduced Serum Medium containing 0.5% PLUS Reagent (ThermoFisher Scientific). 0.175% Lipofectamine LTX was added and the solution was vortexed and incubated at room temperature for 25 min to allow formation of DNA-Lipofectamine LTX complexes before adding to the cells. mCherry was used to indicate which individual cells were successfully transfected (Chiu *et al*, 2004). Cells were kept in an incubator at 37°C and 5% CO_2 for 18–24 h post-transfection. Cells with a red pixel intensity value larger than the maximum value of a control coverslip not transfected with mCherry were assumed to be expressing mCherry.

Table 1. Plasmids.

Gene	Supplier	Item#
hORAI1	Addgene	21638
hORAI2	Addgene	16369
hORAI3	Addgene	16370
mSTIM1 myc	Addgene	17732
mSTIM2 myc	Addgene	17734
ORAI1-CFP	Addgene	19757
STIM1-YFP	Addgene	18857
mCherry	Clontech	PT3974-5

Gene silencing

Small interfering RNA (siRNA) against STIM1 (ON-TARGETplus siRNA, Dharmacon) was used to knock down STIM1 expression in SCG neurons collected from new-born mice (P5-7). As a control, ON-TARGETplus Non-targeting Control siRNA was used. The siRNA was resuspended in RNase-free siRNA buffer containing 60 mM KCl, 6 mM HEPES-pH 7.5 and 0.2 mM $MgCl_2$ to produce 20 μM stock and was stored at −20°C. siRNA concentration was verified using a Nanodrop ND-1000 UV spectrophotometer (Sinica).

Superior cervical ganglion (SCG) neurons were enzymatically dissociated as described previously (Eickholt *et al*, 2007) and resuspended in supplemented Nucleofector Solution for primary mammalian neurons (Lonza, Catalogue #: VSPI-1003) containing 300 nM siRNA and 0.4 μg mCherry plasmid at room temperature in aliquots of 20,000–50,000 neurons each and placed in small cell number (SCN) certified cuvettes. Neurons were electroporated using SCN Basic Neuron Programme 6 of a Nucleofector II machine (Amaxa Scientific) as per the manufacturer's instructions. Pre-equilibrated supplemented RPMI-1640 medium (100 μl) was added immediately after electroporation and the cells were then placed in a humidified 37°C, 5%CO_2 incubator for 10 min and then plated onto pre-coated coverslips. Neurons were allowed to adhere for 10–20 min in the incubator before wells were filled with cell culture medium. Cells were kept in an incubator at 37°C and 5% CO_2 post-electroporation. Relative quantification (rqΔΔCt) of the mRNA levels normalized to housekeeping gene GAPDH using qPCR indicated that the largest reduction of mRNA was achieved at 48 and 72 h. Therefore, 72 h was chosen as the time-point for imaging experiments to allow for a lag in protein turnover.

Calcium imaging

Acetoxymethylester fura-2 solution was prepared on the day of the experiment and consisted of 5 μM fura-2 AM (Life Technologies) with 0.02% v/v pluronic acid (Life Technologies) and 0.1% v/v dimethyl sulfoxide (DMSO, Sigma) in Neurobasal-A medium. Cells were loaded with fura-2 for 30 min before imaging. Coverslips were then placed on an RC-25 chamber platform (Warner Instrument Corp.), placed on the stage of a Nikon Eclipse Ti microscope, and perfused at 32°C with modified HBSS solution containing 140 mM NaCl, 4 mM KCl, 1.8 mM $CaCl_2$, 1 mM $MgCl_2$, 10 mM HEPES, and 5 mM glucose; pH adjusted to 7.4 with NaOH and with an osmolarity of 295–310 mOsm. As a negative control, calcium-free extracellular solution was prepared with the formulation above except for the omission of calcium chloride. Since responses were fully blocked in the nominal absence of Ca^{2+}, it was not deemed necessary to include an additional Ca^{2+} buffer. An eight-line manifold gravity-driven system controlled by an automated solution changer with a common outlet was used to apply extracellular solutions. A Peltier device

© 2022 The Authors

(Pedcool) linked to a custom-built temperature controller (CVScientific) was used to apply cold and heat ramps during the experiments. Temperature protocol traces were recorded immediately before the experiment by placing a thermocouple in the centre of the chamber platform. Cells were perfused with HBSS for at least 5 min before the start of the experiment and the perfusion speed (3 ml/min) and baseline temperature were kept constant throughout the day. Cold ramps to ~ 5°C were applied for 2 min. All agonists and antagonists were prepared as a 1,000× stock solution in dimethyl sulfoxide (DMSO) and stored at −20°C. Compounds used were TRPM8 agonist menthol (300 μM), TRPA1 agonist AITC (50 μM), TRPC5 agonist rosiglitazone (100 μM), non-selective Ca^{2+} channel blockers Cd^{2+} (100 μM) and Gd^{3+} (1 μM), non-selective Ca_V blockers bepridil (10 μM), benidipine (10 μM), nickel (100 μM), and mibefradil (10 μM), L-type selective antagonists nifedipine (10 μM) and verapamil (100 μM), L-type selective agonist BayK8644 (30 μM), ORAI channel inhibitors YM58483/BTP2 (3 μM) and MRS1845 (30 μM), and the ionophore ionomycin (5 μM). Stock solutions were diluted in HBSS and pH adjusted to 7.4. A high K^+ solution or ionomycin was used as a positive control for neurons and HEK293 cells, respectively. A high intensity arc lamp and monochromator (Cairn) was used to excite fura2 and an iXon EMCCD camera (Andor) was used to record fluorescence signals. WinFluor software (University of Strathclyde) was used to record fluorescence at a frame rate of 1 Hz, alternating between capturing an image at 340 nm excitation and 380 nm excitation with exposure time of 100 ms per frame to obtain an $F_{340/380}$ ratio image every 2 s. Fura-2 emission is affected by temperature differently at 340 nm and 380 nm excitation, causing a decrease in $F_{340/380}$ during cooling (Oliver *et al*, 2000), but the impact on measured values of $F_{340/380}$ was small (see Appendix Fig S2).

Analysis and statistics: Calcium imaging

Images were exported as TIFF files. ImageJ (NIH) software was used to measure fluorescence intensity per cell over time. Average background values were calculated from five unoccupied regions of the field of view and subtracted from each frame. The ratio of the fluorescence at 340 and 380 nm excitation was calculated from subtracted traces. Cell cultures from peripheral ganglia contain both neuronal and non-neuronal cells, such as glia, which are readily distinguished by morphology, but an increase of $F_{340/380}$ to > 1 caused by application of a high K^+ solution was used to confirm the identity of neurons (see for example Fig 1F). In a similar way, HEK293 cells that did not respond to application of ionomycin with an increase of $F_{340/380} > 1$ were excluded from analysis. Any cell with an abnormally high Ca^{2+} baseline ($F_{340/380} > 2$) was excluded from analysis. These criteria did not result in exclusion of more than 10% of cells. A positive cold response was defined as an increase of $F_{340/380} > 0.2$, a value obtained from the mean of 3.09 SD of baseline variances in a sample of neurons, excluding 99.9% of false positives. Individual thresholds were calculated for each neuron in experiments that measure temperature activation threshold. For experiments that measure the amplitude of responses, statistical significance was determined by a repeated measures one-way ANOVA + Dunnett's multiple comparison test, unless otherwise stated in the text. A χ^2 test was used to determine statistical significance of percentage changes.

Calculation of cold-induced calcium concentration increase

An inward Ca^{2+}-current of 1pA, undetectable against typical noise and background currents of a sensory neuron, would result in an increase in $[Ca^{2+}]_i$ which can be calculated as follows: Ca^{2+} entry rate = I/(zF) so 1pA of Ca^{2+} current equals an increase of c. 5.2×10^{-18} mol/s. Cold-sensitive mouse DRG or SCG neurons are approximately spherical and ~ 20 μm in diameter, and therefore have a cell volume of c. 4.2×10^{-12} l. Because of buffering and intracellular calcium sequestration, only one in every ~ 100 Ca^{2+} ions contribute to a rise in intracellular free $[Ca^{2+}]$ in neurons (Roussel *et al*, 2006). With the above assumptions, free Ca^{2+} intracellular calcium concentration rises c. 1.24×10^{-8} M/s, or 12.4 nM/s. As described above, cold stimuli were applied for 2 min, during which time the cells are cooled below the threshold of the novel cold-activated calcium increase for ~ 80 s (see Fig 1B, for example). Therefore, the total free calcium increase, not allowing for calcium extrusion, is 992 nM, or from ~ 100 nM at rest to ~ 1,092 nM at peak response, which is an 11-fold increase of $[Ca^{2+}]_i$ for a 1pA current.

Total internal reflection fluorescence (TIRF) microscopy

HEK293 cells were plated on Ibidi μ slides and transfected as described above with a combination of ORAI1-CFP and STIM1-YFP plasmids. The field-of-view was selected randomly before imaging on an Olympus IX83 TIRF microscope with an UAPON OTIRF100X/1.49 objective and CellSens software at 100 ms exposure time. The imaging depth of the TIRF beam was adjusted to c. 100 nm. To minimize cell movement, cells were transferred to a nominal zero-calcium modified HBSS solution (composition as above) 1–5 min before imaging. To minimize bleaching, single images were taken once at the 35°C baseline; once after cooling to 13°C for 2 min, and once after rewarming for 5 min (see Fig 5A).

Analysis

Analysis of the number of puncta and percentage of cell area covered by puncta was performed in ImageJ (NIH) according to Gwozdz *et al*, 2008. Images were processed using a convolution filter (1–1–24–1–1), a black and white image was generated with a threshold of 99.7% pixel intensity, and the "Analyse Particles" function was used to include puncta of 3–14 pixels in size and 0.7–1 circularity. For colocalization analysis in ImageJ, images were first median filtered (1 pixel radius), background subtracted (5 pixel rolling ball radius), and made binary using the Otsu thresholding method. From these images, the percentage of overlapping pixels per cell in the STIM1-YFP and ORAI1-CFP images was measured.

Electrophysiology

Borosilicate patch-clamp pipettes (Science Products GmbH) were pulled using a P-97 horizontal micropipette puller (Sutter Instruments). Before use, all pipettes were fire polished with a Narishige MF900 microforge, giving a resistance when filled of 2.5 to 3.5 MΩ. Pipettes were filled with filtered intracellular solution containing (in mmol/l, from Sigma Aldrich): 140 KCl, 1.6 MgCl2, 2.5 MgATP, 0.5 NaGTP, 10 HEPES (pH adjusted to 7.3 and osmolality to 290 mOsm/l), and 100 μM fura-2 pentapotassium salt (Life Technologies). Whole-cell patch-clamp recordings were performed using

 © 2022 The Authors

an Axiopatch 200B patch-clamp amplifier. Pipette offset was corrected before contacting the cell. Once a giga-seal was obtained between the pipette and the cell, capacitive transients were cancelled before achieving the whole-cell configuration. Series resistance was compensated by 40–60%. Cells were held at −60 mV in the voltage-clamp configuration before applying the voltage pulse protocol. When working in current-clamp mode, the I-Clamp fast mode was used. Whole-cell current and voltage recordings were sampled at 20 KHz and low-pass Bessel filtered at 2 KHz. Data were acquired using Axon pCLAMP software version 10.4 and analysed offline with Clampfit 10 (Molecular Devices, LLC).

Immunocytochemistry

Coverslips were fixed with 4% PFA w/v and 15% w/v sucrose solution in PBS for 20 min at room temperature and slides were washed three times in PBS on a rocking plate. Blocking buffer consisting of 3% w/v BSA and 0.1% v/v Triton X in PBS was added for 1 h and slides were washed again. Primary antibodies anti-MYC (Proteintech, 60003-2-Ig, 1:10 dilution factor) and anti-mCherry (GeneTex, GTX128508, 1:100 dilution factor) were added in PBS with 0.1% v/v Triton X overnight at 4°C. The next day, slides were washed three times, and secondary antibodies were added at 1:500 dilution factor in PBS for 1 h at room temperature. Finally, slides were washed and Fluosave and glass coverslips (VWR International) were added. Slides were stored in the dark at 4°C until imaging was performed using an Axioplan2 microscope (Zeiss) and Axiovision software (Zeiss).

Immunohistochemistry

Sections were labelled with fluorescent antibodies using the two-step indirect staining method. Primary antibodies anti-nNOS (Abcam, Ab1376, 1:1000 dilution factor; Millipore, Ab5380, 1:4000 dilution factor; ECM, NP2141, 1:300 dilution factor), anti-βIII Tubulin (Promega, G712A, 1:1000 dilution factor), and anti-TH (Milipore, Ab152, 1:500 dilution factor) were added in PBS with 0.1% v/v Triton X overnight at room temperature. Slides were washed three times in PBS on a rocking platform and incubated with secondary antibodies at dilution factor 1:1,000 in PBS for 2 h at room temperature. In some experiments, DAPI (4′,6-diamidino-2-phenylindole; AnaSpec) was added to the secondary antibody solution to stain nuclei blue. After staining, Fluosave™ reagent (CalBiochem) and glass coverslips (VWR International) were added to the slides. Then, slides were stored at 4°C in the dark until being imaged using an Axioplan2 microscope (Zeiss) and Axiovision software (Zeiss). Images were exported as Tagged Image File Format (TIFF) files.

Data availability

This study includes no data deposited in external repositories.

Expanded View for this article is available online.

Acknowledgements
The work was supported by Wellcome Trust Investigator Award 205006/Z/16/Z and UKRI/BBSRC Award BB/L002787/1 to PMcN, and by UKRI/BBSRC Training Grant BB/L502674/1 to TJB. We thank Mohamed Trebak for ORAI3$^{-/-}$ mice, Carl Hobbs for nNOS antibodies, and Christian Lohmann for his comments on the manuscript.

Author contributions
Peter A McNaughton: Conceptualization; supervision; funding acquisition; validation; investigation; methodology; project administration; writing—review and editing. **Tamara J Buijs:** Conceptualization; data curation; formal analysis; investigation; methodology; writing—original draft. **Bruno Vilar:** Conceptualization; data curation; investigation; methodology; writing—review and editing. **Chun-Hsiang Tan:** Conceptualization; investigation; methodology.

Disclosure and competing interests statement
The authors declare that they have no conflict of interest.

References

Babes A, Zorzon D, Reid G (2004) Two populations of cold-sensitive neurons in rat dorsal root ganglia and their modulation by nerve growth factor. *Eur J Neurosci* 20: 2276 – 2282

Babes A, Zorzon D, Reid G (2006) A novel type of cold-sensitive neuron in rat dorsal root ganglia with rapid adaptation to cooling stimuli. *Eur J Neurosci* 24: 691 – 698

Bautista DM, Siemens J, Glazer JM, Tsuruda PR, Basbaum AI, Stucky CL, Jordt SE, Julius D (2007) The menthol receptor TRPM8 is the principal detector of environmental cold. *Nature* 448: 204 – 208

Buijs TJ, McNaughton PA (2020) The role of cold-sensitive ion channels in peripheral thermosensation. *Front Cell Neurosci* 14: 262

Caterina MJ, Schumacher MA, Tominaga M, Rosen TA, Levine JD, Julius D (1997) The capsaicin receptor: a heat-activated ion channel in the pain pathway. *Nature* 389: 816 – 824

Cesare P, Mcnaughton P (1996) A novel heat-activated current in nociceptive neurons and its sensitization by bradykinin. *Proc Natl Acad Sci USA* 93: 15435 – 15439

Chiu Y, Cao H, Jacque J, Stevenson M, Rana TM (2004) Inhibition of human immunodeficiency virus type 1 replication by RNA interference directed against human transcription elongation factor P-TEFb (CDK9/CyclinT1). *J Virol* 78: 2517 – 2529

Daanen HAM, Van Der Struijs NR (2005) Resistance index of frostbite as a predictor of cold injury in arctic operations. *Aviat Space Environ Med* 76: 1119 – 1122

Derler I, Schindl R, Fritsch R, Heftberger P, Riedl MC, Begg M, House D, Romanin C (2013) The action of selective CRAC channel blockers is affected by the Orai pore geometry. *Cell Calcium* 53: 139 – 151

Dhaka A, Murray AN, Mathur J, Earley TJ, Petrus MJ, Patapoutian A (2007) TRPM8 is required for cold sensation in mice. *Neuron* 54: 371 – 378

Eickholt BJ, Ahmed AI, Davies M, Papakonstanti EA, Pearce W, Starkey ML, Bilancio A, Need AC, Smith AJH, Hall SM *et al* (2007) Control of axonal growth and regeneration of sensory neurons by the p110δ PI 3-kinase. *PLoS One* 2: e869

Förstermann U, Sessa WC (2012) Nitric oxide synthases: Regulation and function. *Eur Heart J* 33: 829 – 837

Franz DN, Iggo A (1968) Conduction failure in myelinated and non-myelinated axon at low temperatures. *J Physiol* 199: 319 – 345

Gao R, Gao X, Xia J, Tian Y, Barrett JE, Dai Y, Hu H (2013) Potent analgesic effects of a store-operated calcium channel inhibitor. *Pain* 154: 2034 – 2044

© 2022 The Authors

Gong J, Liu J, Ronan EA, He F, Cai W, Fatima M, Zhang W, Lee H, Li Z, Kim GH *et al* (2019) A cold-sensing receptor encoded by a glutamate receptor Gene. *Cell* 178: 1375 – 1386.e11

Greene LA, Tischler AS (1976) Establishment of a noradrenergic clonal line of rat adrenal Pheochromocytoma cells which respond to nerve growth-factor. *Proc Natl Acad Sci USA* 73: 2424 – 2428

Gruszczynska-Biegala J, Sladowska M, Kuznicki J (2016) AMPA receptors are involved in store-operated calcium entry and interact with STIM proteins in rat primary cortical neurons. *Front Cell Neurosci* 10: 251

Gwozdz T, Dutko-Gwozdz J, Zarayskiy V, Peter K, Bolotina VM (2008) How strict is the correlation between STIM1 and Orai1 expression, puncta formation, and ICRAC activation? *Am J Physiol Cell Physiol* 295: C1133 – C1140

Hogea A, Shah S, Jones F, Carver CM, Hao H, Liang C, Huang D, Du X, Gamper N (2021) Junctophilin-4 facilitates inflammatory signalling at plasma membrane-endoplasmic reticulum junctions in sensory neurons. *J Physiol* 599: 2103 – 2123

Hou X, Pedi L, Diver M, Long SB (2012) Crystal structure of the calcium release-activated calcium channel Orai. *Science* 338: 1308 – 1313

Knowlton WM, Bifolck-Fisher A, Bautista DM, McKemy DD (2010) TRPM8, but not TRPA1, is required for neural and behavioral responses to acute noxious cold temperatures and cold-mimetics *in vivo*. *Pain* 150: 340 – 350

Kwan H, Huang Y, Yao XCN-C (2000) Store-operated calcium entry in vascular endothelial cells is inhibited by cGMP via a protein kinase G-dependent mechanism. *J Biol Chem* 275: 6758 – 6763

Lesage F, Terrenoire C, Romey G, Lazdunski M (2000) Human TREK2, a 2P domain mechano-sensitive K+ channel with multiple regulations by polyunsaturated fatty acids, lysophospholipids, and Gs, Gi, and Gq protein-coupled receptors. *J Biol Chem* 275: 28398 – 28405

Lewis T (1930) Observations upon the reactions of the vessels of the human skin to cold. *Heart* 15: 177 – 208

Lin S, Fagan KA, Li K, Shaul PW, Cooper DMF, Rodman DM (2000) Sustained endothelial nitric-oxide synthase activation requires Capacitative Ca 2 entry. *Biochemistry* 275: 17979 – 17985

Lirk P, Poroli M, Rigaud M, Fuchs A, Fillip P, Huang CY, Ljubkovic M, Sapunar D, Hogan Q (2008) Modulators of calcium influx regulate membrane excitability in rat dorsal root ganglion neurons. *Anesth Analg* 107: 673 – 685

Liu X, Wang H, Jiang Y, Zheng Q, Petrus M, Zhang M, Zheng S, Schmedt C, Dong X, Xiao B (2019a) STIM1 thermosensitivity defines the optimal preference temperature for warm sensation in mice. *Cell Res* 29: 95 – 109

Liu Z, Wei Y, Zhang L, Yee PP, Johnson M, Zhang X, Gulley M, Atkinson JM, Trebak M, Wang HG *et al* (2019b) Induction of store-operated calcium entry (SOCE) suppresses glioblastoma growth by inhibiting the hippo pathway transcriptional coactivators YAP/TAZ. *Oncogene* 38: 120 – 139

Lopez JJ, Jardin I, Albarrán L, Sanchez-Collado J, Cantonero C, Salido GM, Smani T, Rosado JA (2020) Molecular basis and regulation of store-operated calcium entry. *Adv Exp Med Biol* 1131: 445 – 469

Lytton J, Westlin M, Hanley MR (1991) Thapsigargin inhibits the sarcoplasmic or endoplasmic reticulum Ca-ATPase family of calcium pumps. *J Biol Chem* 266: 17067 – 17071

Maingret F, Lauritzen I, Patel AJ, Heurteaux C, Reyes R, Lesage F, Lazdunski M, Honore E (2000) TREK-1 is a heat-activated background K(+) channel. *EMBO J* 19: 2483 – 2491

McKemy DD, Neuhausser WM, Julius D (2002) Identification of a cold receptor reveals a general role for TRP channels in thermosensation. *Nature* 416: 52 – 58

McNally BA, Somasundaram A, Jairaman A, Yamashita M, Prakriya M (2013) The C- and N-terminal STIM1 binding sites on Orai1 are required for both trapping and gating CRAC channels. *J Physiol* 591: 2833 – 2850

Munns C, AlQatari M, Koltzenburg M (2007) Many cold sensitive peripheral neurons of the mouse do not express TRPM8 or TRPA1. *Cell Calcium* 41: 331 – 342

Nelson HA, Roe MW (2018) Molecular physiology and pathophysiology of stromal interaction molecules. *Exp Biol Med* 243: 451 – 472

Noël J, Zimmermann K, Busserolles J, Deval E, Alloui A, Diochot S, Guy N, Borsotto M, Reeh P, Eschalier A *et al* (2009) The mechano-activated K±channels TRAAK and TREK-1 control both warm and cold perception. *EMBO J* 28: 1308 – 1318

Oliver AE, Baker GA, Fugate RD, Tablin F, Crowe JH (2000) Effects of temperature on calcium-sensitive fluorescent probes. *Biophys J* 78: 2116 – 2126

Park JF, Luo ZD (2010) Calcium channel functions in pain processing. *Channels* 4: 510 – 517

Peier AM, Moqrich A, Hergarden AC, Reeve AJ, Andersson DA, Story GM, Earley TJ, Dragoni I, McIntyre P, Bevan S *et al* (2002) A TRP channel that senses cold stimuli and menthol. *Cell* 108: 705 – 715

Pereira V, Busserolles J, Christin M, Devilliers M, Poupon L, Legha W, Alloui A, Aissouni Y, Bourinet E, Lesage F *et al* (2014) Role of the TREK2 potassium channel in cold and warm thermosensation and in pain perception. *Pain* 155: 2534 – 2544

Prakriya M, Lewis RS (2015) Store-operated calcium channels. *Physiol Rev* 95: 1383 – 1436

Qi Z, Wang Y, Zhou H, Liang N, Yang L, Liu L, Zhang W (2016) The central analgesic mechanism of YM-58483 in attenuating neuropathic pain in rats. *Cell Mol Neurobiol* 36: 1035 – 1043

Qiu R, Lewis RS (2019) Structural features of STIM and Orai underlying store-operated calcium entry. *Curr Opin Cell Biol* 57: 90 – 98

Ramsey IS, Delling M, Clapham DE (2006) An introduction to TRP channels. *Annu Rev Physiol* 68: 619 – 647

Ran C, Hoon MA, Chen X (2016) The coding of cutaneous temperature in the spinal cord. *Nat Neurosci* 19: 1 – 11

Roussel C, Erneux T, Schiffmann SN, Gall D (2006) Modulation of neuronal excitability by intracellular calcium buffering: From spiking to bursting. *Cell Calcium* 39: 455 – 466

Sadler KE, Moehring F, Stucky CL (2020) Keratinocytes contribute to normal cold and heat sensation. *eLife* 9: e58625

Scrimgeour NR, Wilson DP, Rychkov GY (2012) Glu 106 in the Orai1 pore contributes to fast Ca^{2+}–dependent inactivation and pH dependence of Ca^{2+} release-activated Ca^{2+} (CRAC) current. *Biochem J* 441: 743 – 753

Smith MP, Beacham D, Ensor E, Koltzenburg M (2004) Cold-sensitive, menthol-insensitive neurons in the murine sympathetic nervous system. *Neuroreport* 15: 1399 – 1403

Story GM, Peier AM, Reeve AJ, Eid SR, Mosbacher J, Hricik TR, Earley TJ, Hergarden AC, Andersson DA, Hwang SW *et al* (2003) ANKTM1, a TRP-like channel expressed in nociceptive neurons, is activated by cold temperatures. *Cell* 112: 819 – 829

Talavera K, Startek JB, Alvarez-Collazo J, Boonen B, Alpizar YA, Sanchez A, Naert R, Nilius B (2020) Mammalian transient receptor potential TRPA1 channels: From structure to disease. *Physiol Rev* 100: 725 – 803

Tan CL, Knight ZA (2018) Regulation of body temperature by the nervous system. *Neuron* 98: 31 – 48

Tan C-H, McNaughton PA (2016) The TRPM2 ion channel is required for sensitivity to warmth. *Nature* 536: 460 – 463

Taylor WF, Bishop S, Fred W (1993) A role for nitric thermoregulatory oxide in active vasodilation. *Am J Physiol Heart Circ Physiol* 264: 1355 – 1359

Uhlen M, Fagerberg L, Hallstrom BM, Lindskog C, Oksvold P, Mardinoglu A, Sivertsson A, Kampf C, Sjostedt E, Asplund A *et al* (2015) Tissue-based map of the human proteome. *Science* 347: 1260419

© 2022 The Authors

Usoskin D, Furlan A, Islam S, Abdo H, Lönnerberg P, Lou D, Hjerling-Leffler J, Haeggström J, Kharchenko O, Kharchenko PV *et al* (2015) Unbiased classification of sensory neuron types by large-scale single-cell RNA sequencing. *Nat Neurosci* 18: 145 – 153

Vandewauw I, De Clercq K, Mulier M, Held K, Pinto S, Van Ranst N, Segal A, Voet T, Vennekens R, Zimmermann K *et al* (2018) A TRP channel trio mediates acute noxious heat sensing. *Nature* 555: 662 – 666

Vanggaard L (1975) Physiological reactions to wet-cold. *Aviat Space Environ Med* 46: 33 – 36

Viana F, de la Peña E, Belmonte C (2002) Specificity of cold thermotransduction is determined by differential ionic channel expression. *Nat Neurosci* 5: 254 – 260

Viatchenko-Karpinski V, Ling J, Gu JG (2018) Characterization of temperature-sensitive leak K+ currents and expression of TRAAK, TREK-1, and TREK2 channels in dorsal root ganglion neurons of rats. *Mol Brain* 11: 40

Vilar B, Tan CH, McNaughton PA (2020) Heat detection by the TRPM2 ion channel. *Nature* 584: E5 – E12

Vriens J, Owsianik G, Hofmann T, Philipp SE, Stab J, Chen X, Benoit M, Xue F, Janssens A, Kerselaers S *et al* (2011) TRPM3 is a nociceptor channel involved in the detection of noxious heat. *Neuron* 70: 482 – 494

Wei D, Mei Y, Xia J, Hu H (2017) Orai1 and Orai3 mediate store-operated calcium entry contributing to neuronal excitability in dorsal root ganglion neurons. *Front Cell Neurosci* 11: 400

Xiao B, Coste B, Mathur J, Patapoutian A (2011) Temperature-dependent STIM1 activation induces Ca(2)+ influx and modulates gene expression. *Nat Chem Biol* 7: 351 – 358

Zimmermann K, Lennerz JK, Hein A, Link AS, Kaczmarek JS, Delling M, Uysal S, Pfeifer JD, Riccio A, Clapham DE (2011) Transient receptor potential cation channel, subfamily C, member 5 (TRPC5) is a cold-transducer in the peripheral nervous system. *Proc Natl Acad Sci USA* 108: 18114 – 18119

Zitt C, Strauss B, Schwarz EC, Spaeth N, Rast G, Hatzelmann A, Hoth M (2004) Potent inhibition of Ca^{2+} release-activated Ca2+ channels and T-lymphocyte activation by the Pyrazole derivative BTP2. *J Biol Chem* 279: 12427 – 12437

Zurborg S, Yurgionas B, Jira JA, Caspani O, Heppenstall PA (2007) Direct activation of the ion channel TRPA1 by Ca^{2+}. *Nat Neurosci* 10: 277 – 279

License: This is an open access article under the terms of the Creative Commons Attribution License, which permits use, distribution and reproduction in any medium, provided the original work is properly cited.

© 2022 The Authors

Expanded View Figures

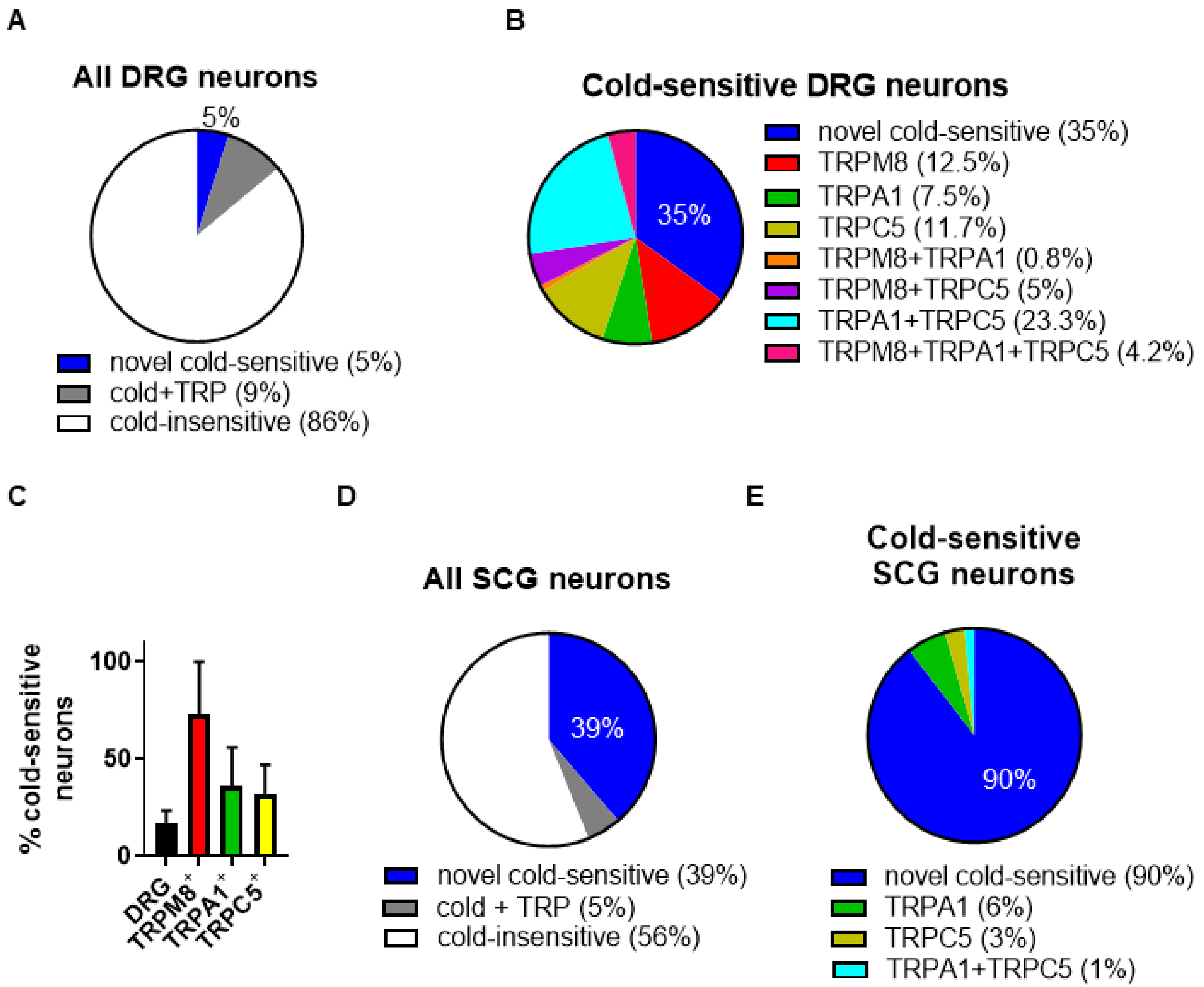

Figure EV1. Expression of TRPM8 is more highly correlated with cold-sensitivity than expression of TRPA1 or TRPC5.

A Percentage of DRG neurons that were both cold-sensitive and expressed one or more of TRPM8, TRPA1, or TRPC5 (grey), and novel cold-sensitive DRG neurons that did not express any of these TRP channels (blue). Protocol as in Fig 1A and E, $n = 859$ neurons imaged on three separate days. 5% of DRG neurons expressed only a novel cold-sensitive mechanism (blue).

B Different populations of cold-sensitive neurons by expression of TRPM8, TRPA1, and TRPC5. $n = 99$ cold-sensitive neurons selected from the experiment in A. 35% of cold-sensitive neurons did not express any of TRPM8, TRPA1 or TRPC5 (blue).

C Average percentage of cold-sensitive DRG neurons as follows: out of all neurons (black); out of the subpopulation of DRG neurons expressing TRPM8 but not TRPA1 or TRPC5 (red); out of the subpopulation of DRG neurons expressing TRPA1, but not TRPM8 or TRPC5 (green); and out of the subpopulation of DRG neurons expressing TRPC5, but not TRPM8 or TRPA1 (yellow, all mean ± SEM).

D Percentage of total SCG neurons that were both cold-sensitive and expressed either TRPA1 or TRPC5 (grey); and novel cold-sensitive SCG neurons that did not express any of the TRP channels (blue). No neuron responded to the TRPM8 agonist menthol ($n = 155$ neurons, 3 days). 39% of SCG neurons expressed a novel cold-sensitive mechanism.

E Different populations of cold-sensitive SCG neurons by expression of TRPA1 and TRPC5. $n = 67$ neurons taken from (D). 90% of cold-sensitive neurons did not express any of TRPM8, TRPA1, or TRPC5 (blue).

Source data are available online for this figure.

 © 2022 The Authors

Figure EV2. Effect of Ca_V channel antagonists on cold-induced Ca^{2+} influx in SCG neurons.

A Cd^{2+} (100 μM) which blocks Ca_V and ORAI channels but activates TRPA1, suppresses cold-induced Ca^{2+} influx in SCG neurons. Temperature trace below.
B Collected results with Cd^{2+} ($P < 0.0001$, RM one-way ANOVA + Dunnett's test, $n = 60$ neurons on 8 coverslips). Cooling SCG neurons from 34°C to 6°C in the presence of Cd^{2+} reversibly decreased cold-response amplitudes by 87%.
C Gd^{3+} (1 μM), which blocks Ca_V and ORAI channels, but activates TRPC5, suppresses cold-induced Ca^{2+} influx in SCG neurons.
D Collected results with Gd^{3+} ($P < 0.0001$, RM one-way ANOVA + Dunnett's test, $n = 78$ neurons, 12 coverslips). Cooling SCG neurons from 35°C to 5°C in the presence of Gd^{3+} reversibly decreased cold-response amplitudes by 82%.
E Effect of L-type Ca_V channel blocker Verapamil (100 μM). Verapamil had opposing effects on different neurons, increasing some responses (blue trace), and decreasing other responses (black trace). There were also many neurons with intermediate levels of either suppression or enhancement.
F Collected results with verapamil ($P < 0.0001$, RM one-way ANOVA + Dunnett's test, $n = 277$ neurons, 4 coverslips). 13% of cold responses were fully blocked by Verapamil, as shown in (E) (black trace).
G Non-selective Ca_V channel blocker Mibefradil (10 μM) enhanced some responses (blue) and suppressed others (black).
H No overall effect on $F_{340/380}$ (114 cold-sensitive neurons).
I Effect of L-type Ca_V channel blocker Nifedipine (10 μM) on cold-induced Ca^{2+} influx in SCG neurons. Nifedipine enhanced cold-induced Ca^{2+} influx in some neurons (blue trace) but suppressed it in others (black).
J Normalized results of 18 cold-sensitive neurons exposed to nifedipine. No overall significant difference in cold response amplitude.
K Effect of non-selective Ca_V channel blocker Bepridil (10 μM).
L Normalized results from 45 cold-sensitive neurons exposed to bepridil. No overall significant difference in cold response amplitude. All error bars mean ± SEM.

Source data are available online for this figure.

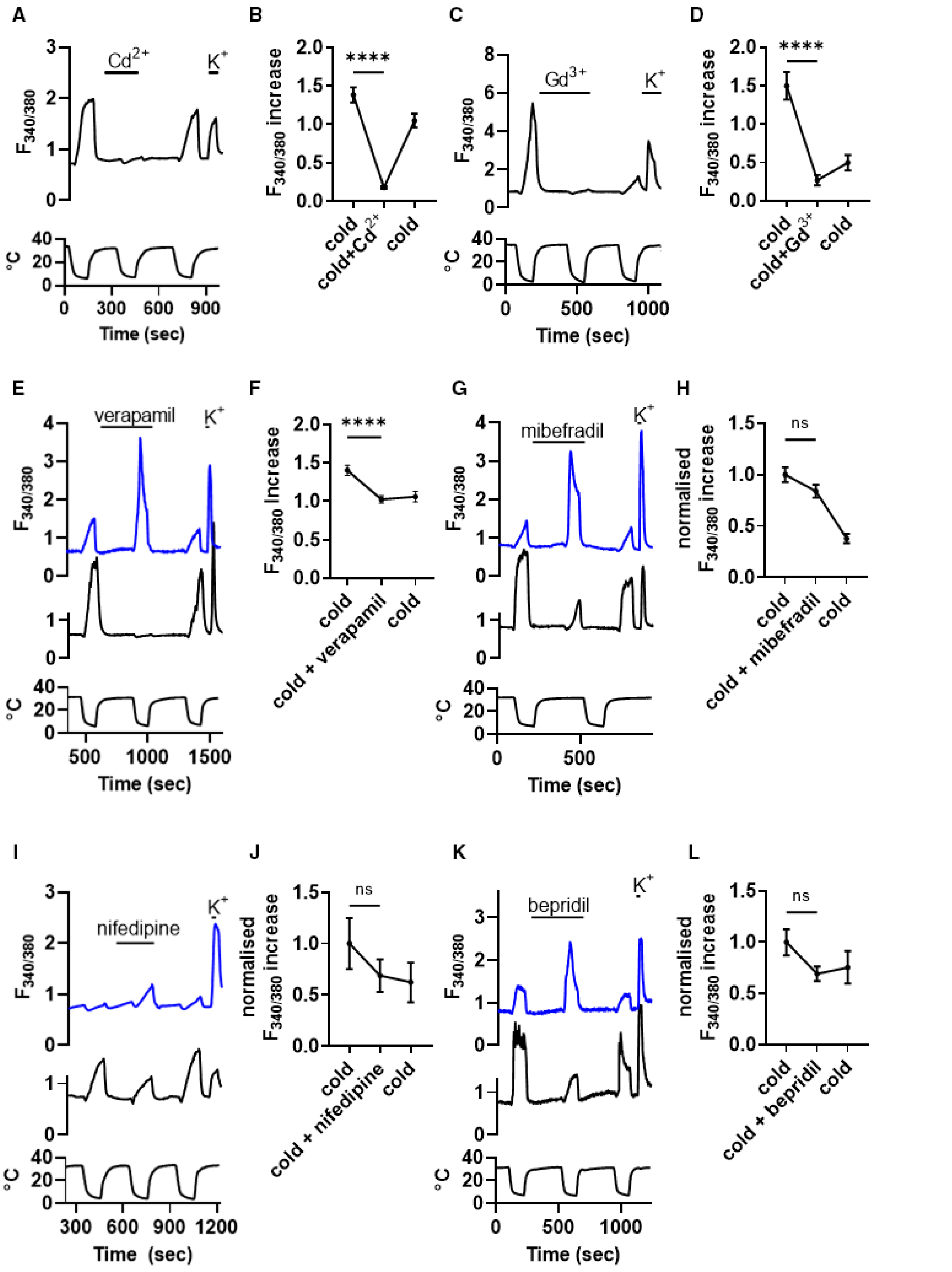

Figure EV2.

 © 2022 The Authors

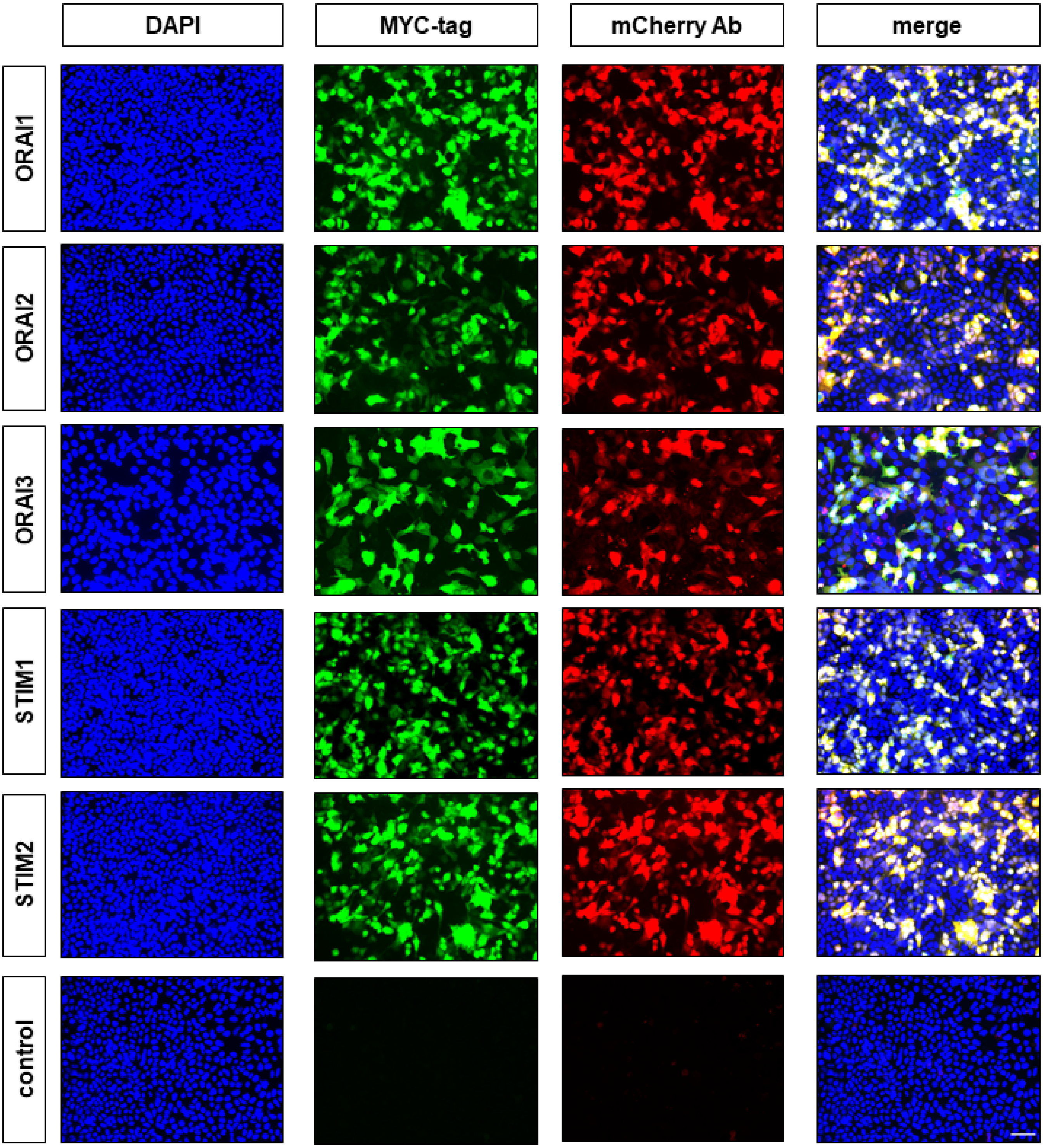

Figure EV3. Presence of mCherry reliably indicates successful overexpression of all STIM and ORAI plasmids.

Immunocytochemistry fluorescence images showing HEK293 cells transfected with a combination of mCherry and one of the MYC-tagged plasmids for STIM or ORAI proteins (indicated at left). Control cells (bottom) underwent same protocol without DNA. Cells were stained with nuclear marker DAPI and antibodies against mCherry and MYC. Not all cells were successfully transfected but all those cells expressing mCherry also express the cotransfected STIM/ORAI proteins, as shown by the merged images (right). $n = 4$ cultures each. Scale bar = 50 μm.

Source data are available online for this figure.

© 2022 The Authors

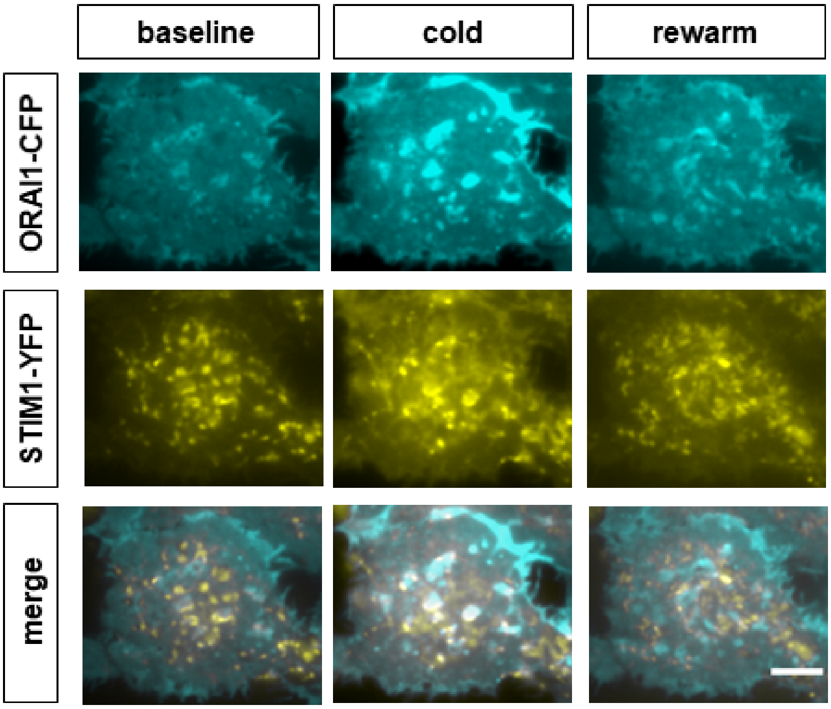

Figure EV4. The effect of cold on ORAI1 puncta formation and colocalization of ORAI1 and STIM1 in a HEK293 cell.

Similar to Fig 5A but with a larger number of visible STIM1 and ORAI1 puncta. Scale bar = 5 µm.

Source data are available online for this figure.

 © 2022 The Authors

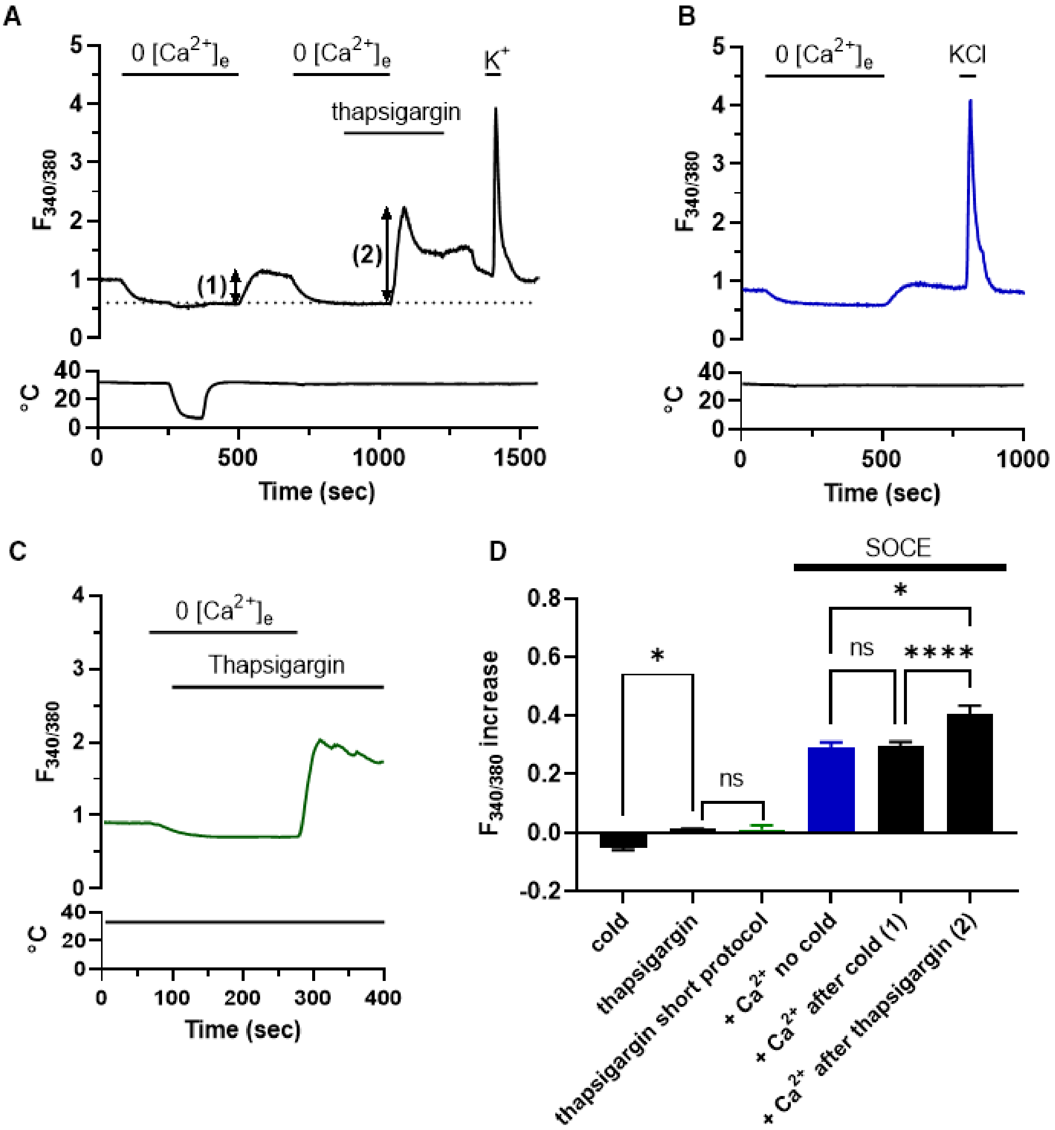

Figure EV5. Cold does not cause discharge of calcium from subcellular stores.

A, B Representative Ca^{2+} imaging trace showing responses of an SCG neuron to cold and to 10 μM thapsigargin, both in the absence of extracellular Ca^{2+}. There was no detectable increase in intracellular calcium caused by discharge of stores in either case (the small negative deflection during application of cold is an artefact caused by the effect of cold on fura-2, see Appendix Fig S2). However on readmission of extracellular calcium the prior discharge of store calcium by thapsigargin is indicated by a significant store-operated calcium entry (SOCE, arrow (2)), while following application of a cold stimulus, SOCE induced by the re-admission of extracellular Ca^{2+} (arrow (1)) is identical to that observed with no cold stimulus - see (B).

C Thapsigargin does not cause a detectable calcium increase even when applied very soon (30 s) after transfer to zero Ca^{2+}.

D Bar chart summarizing response amplitudes (mean ± SEM) with protocol shown in (A) ($n = 169$) or (B) ($n = 26$) or (C) ($n = 34$). No difference in SOCE with or without cold application (ns, $P > 0.05$) while SOCE following thapsigargin is significantly larger than that following cold application ($P < 0.0001$, RM one-way ANOVA + Tukey's test).

Source data are available online for this figure.

© 2022 The Authors
