# Supplementary material for: STIM1 and ORAI1 form a novel cold transduction mechanism in sensory and sympathetic neurons
Source: EMBO J. 2022 Dec 16;42(3):e111348. doi: 10.15252/embj.2022111348 (PMC9890232; doi:10.15252/embj.2022111348)
Supplement: Supplementary file 11 — Source Data for Figure 6 [file EMBJ-42-e111348-s001.zip › Figure_6/EMBOJ-2022-111348R1_SourceDataForFigure6.pdf]

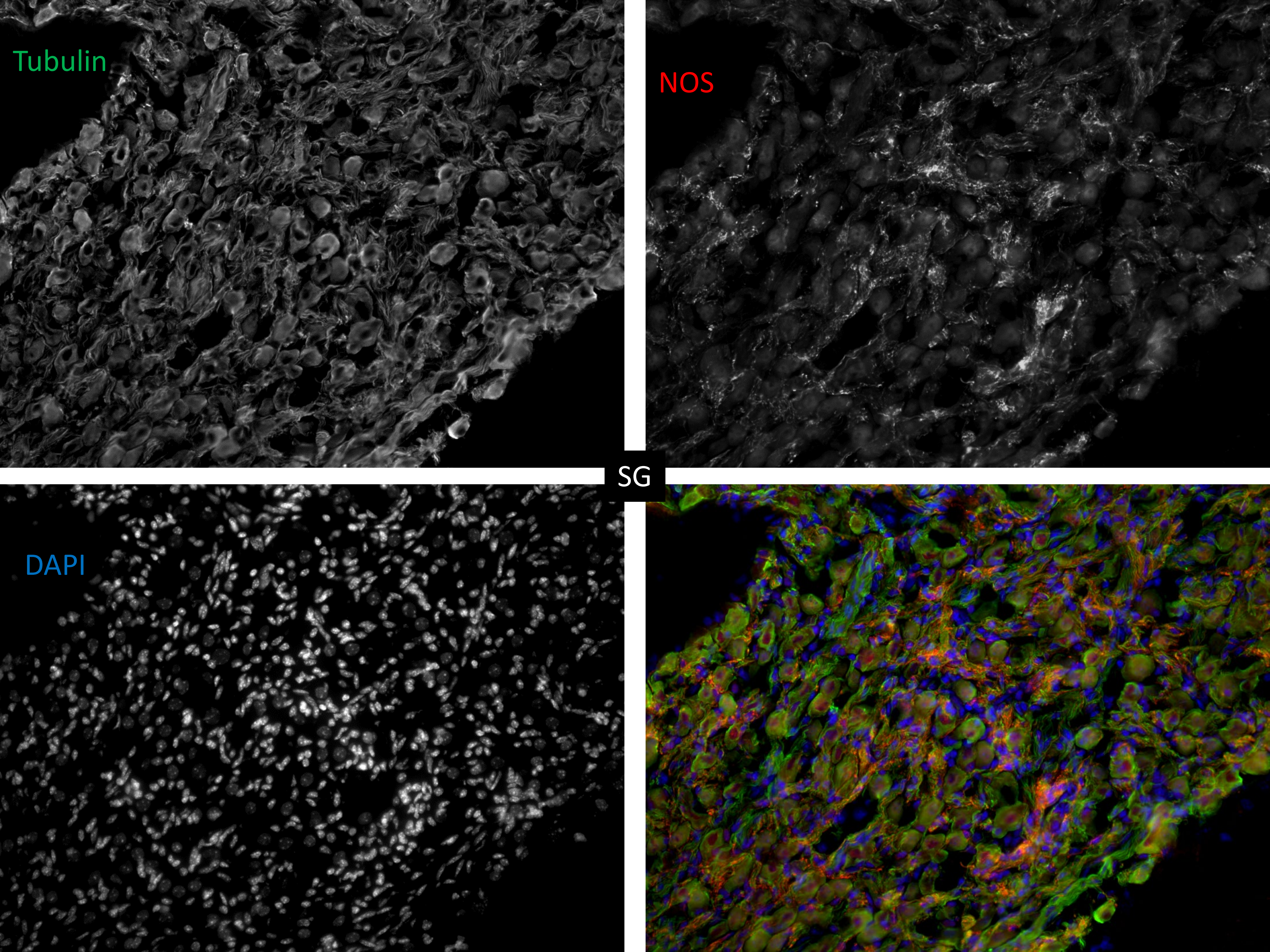
Tubulin
NOS
SG
DAPI

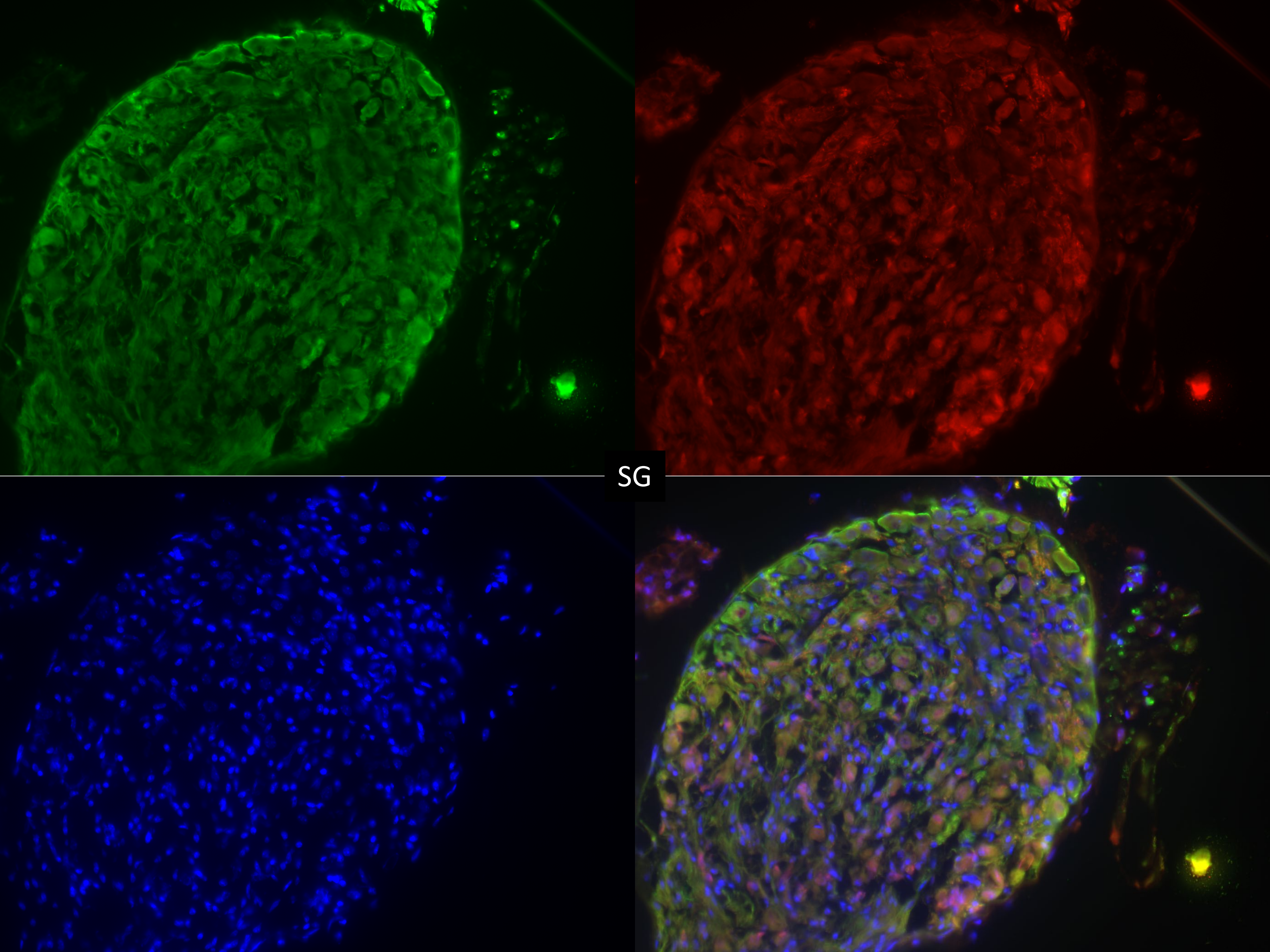
SG

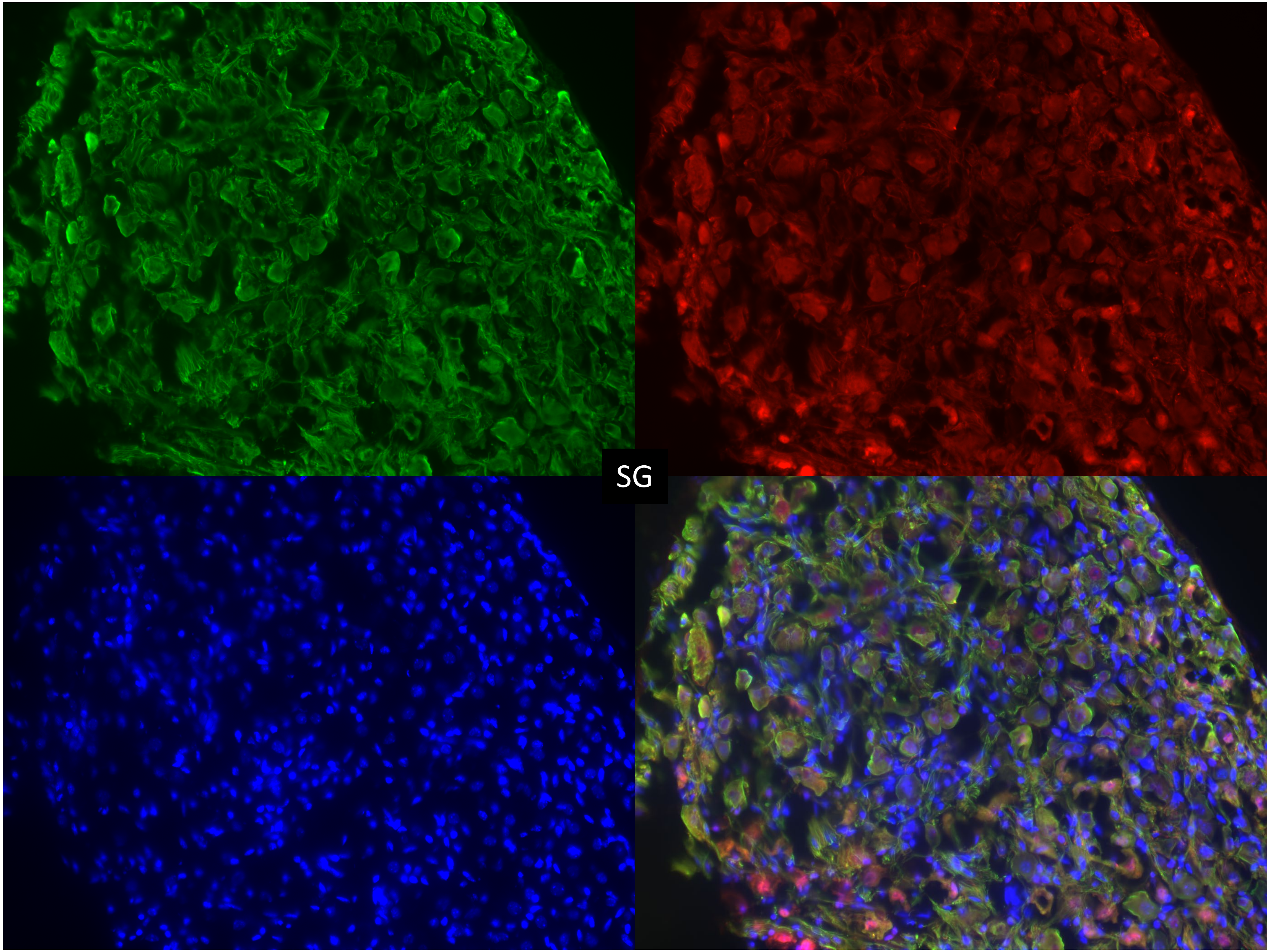
SG

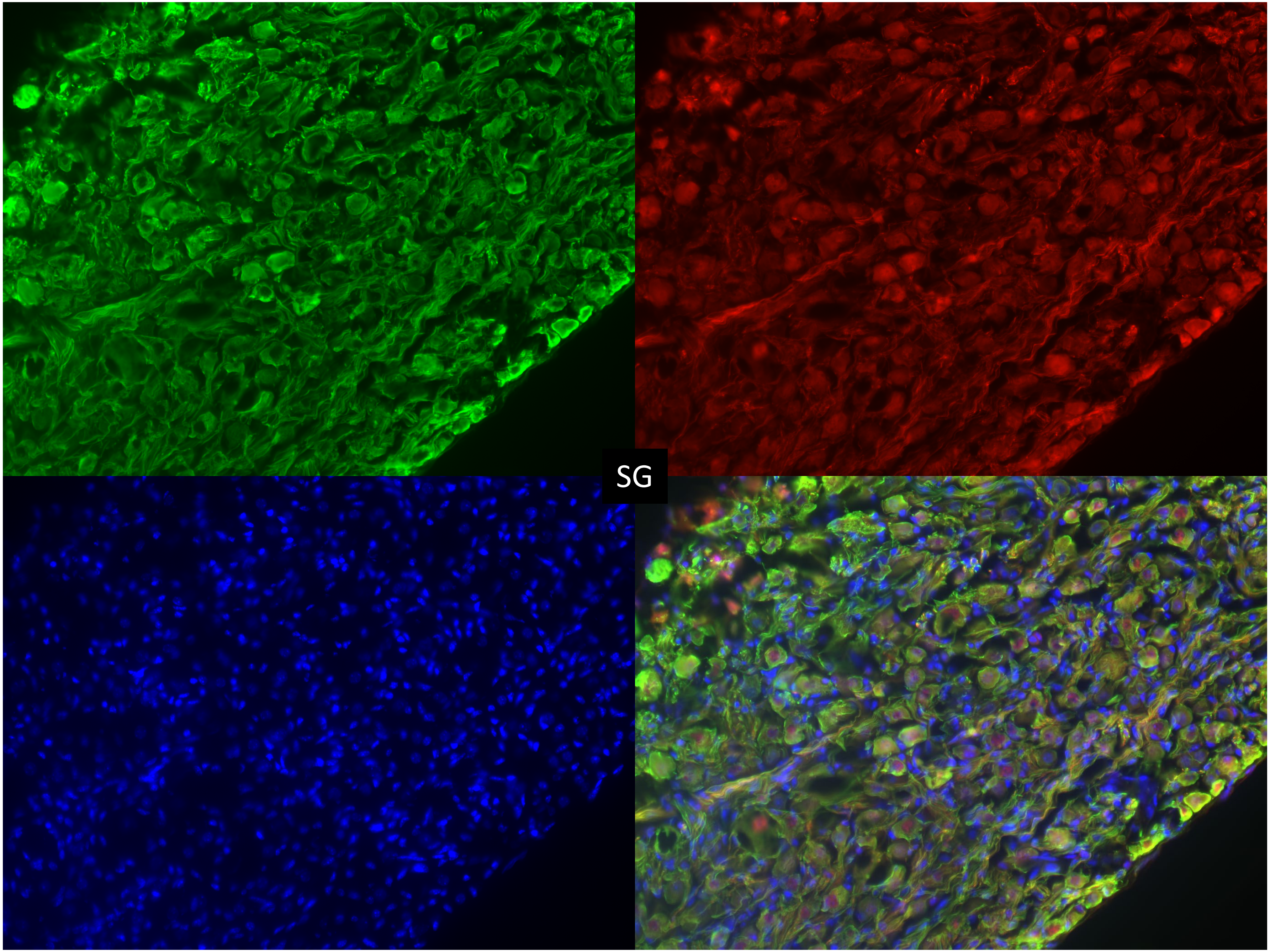
SG

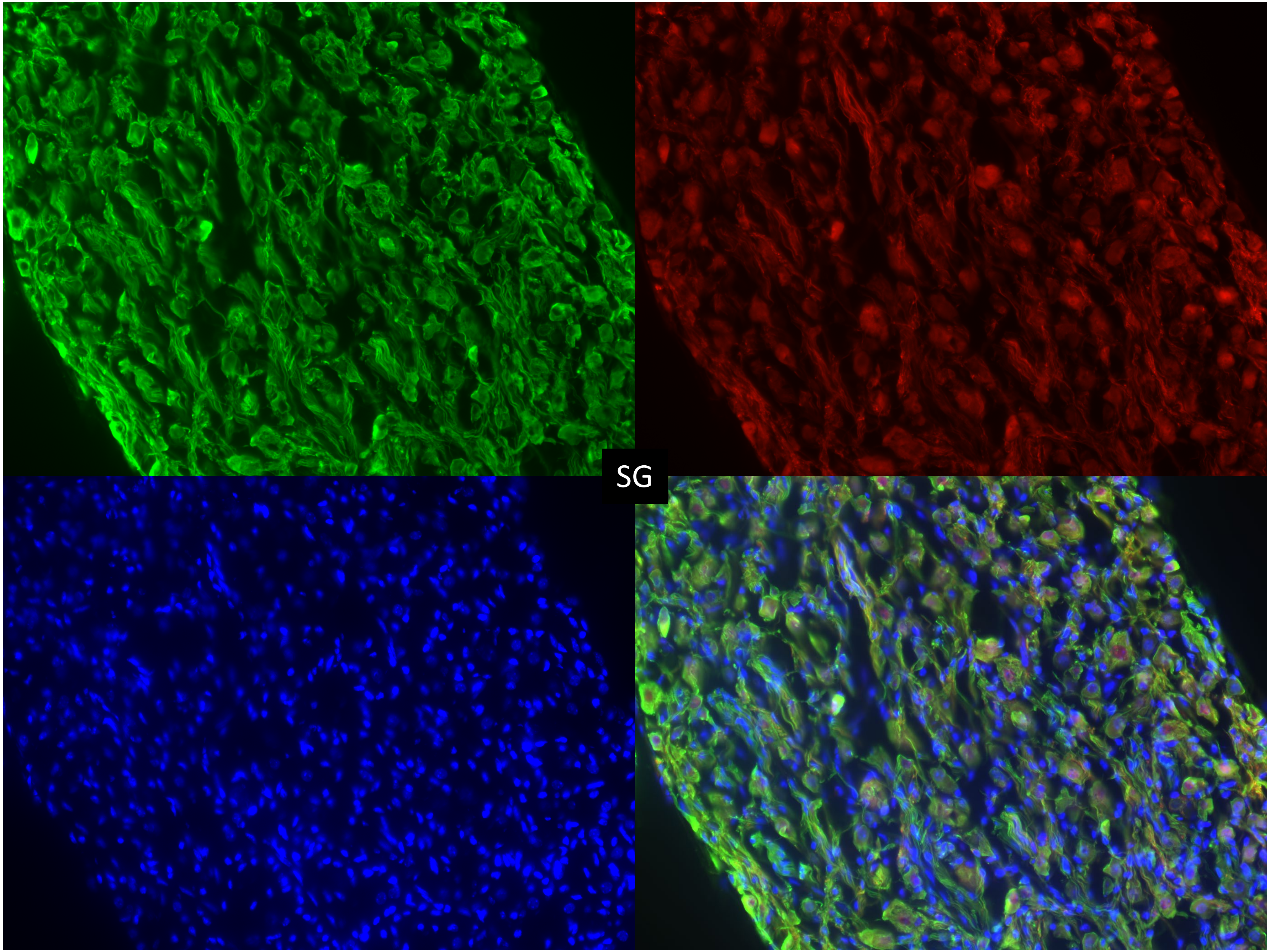
SG

TH

nNOS

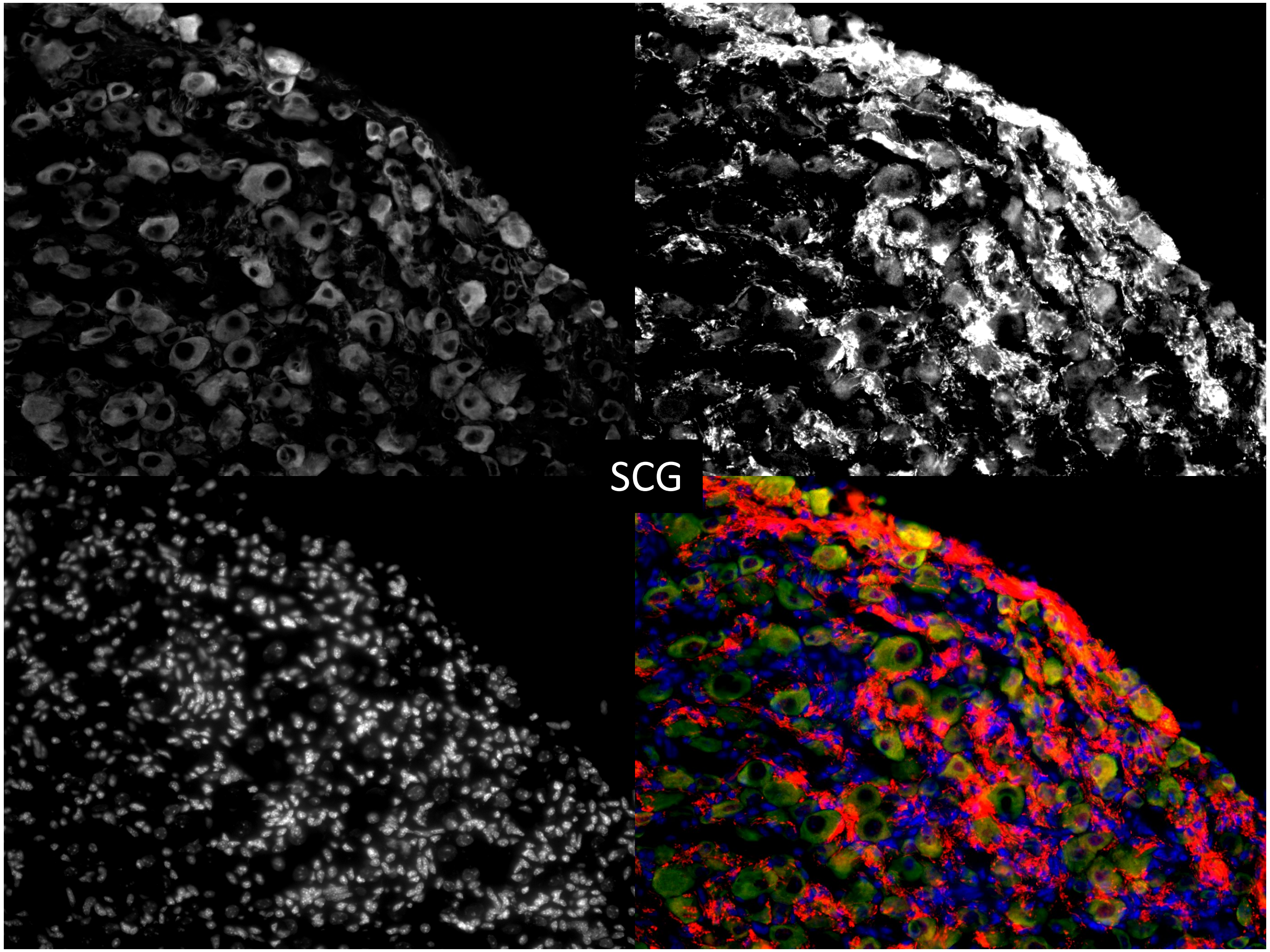

DAPI

nNOS

TH

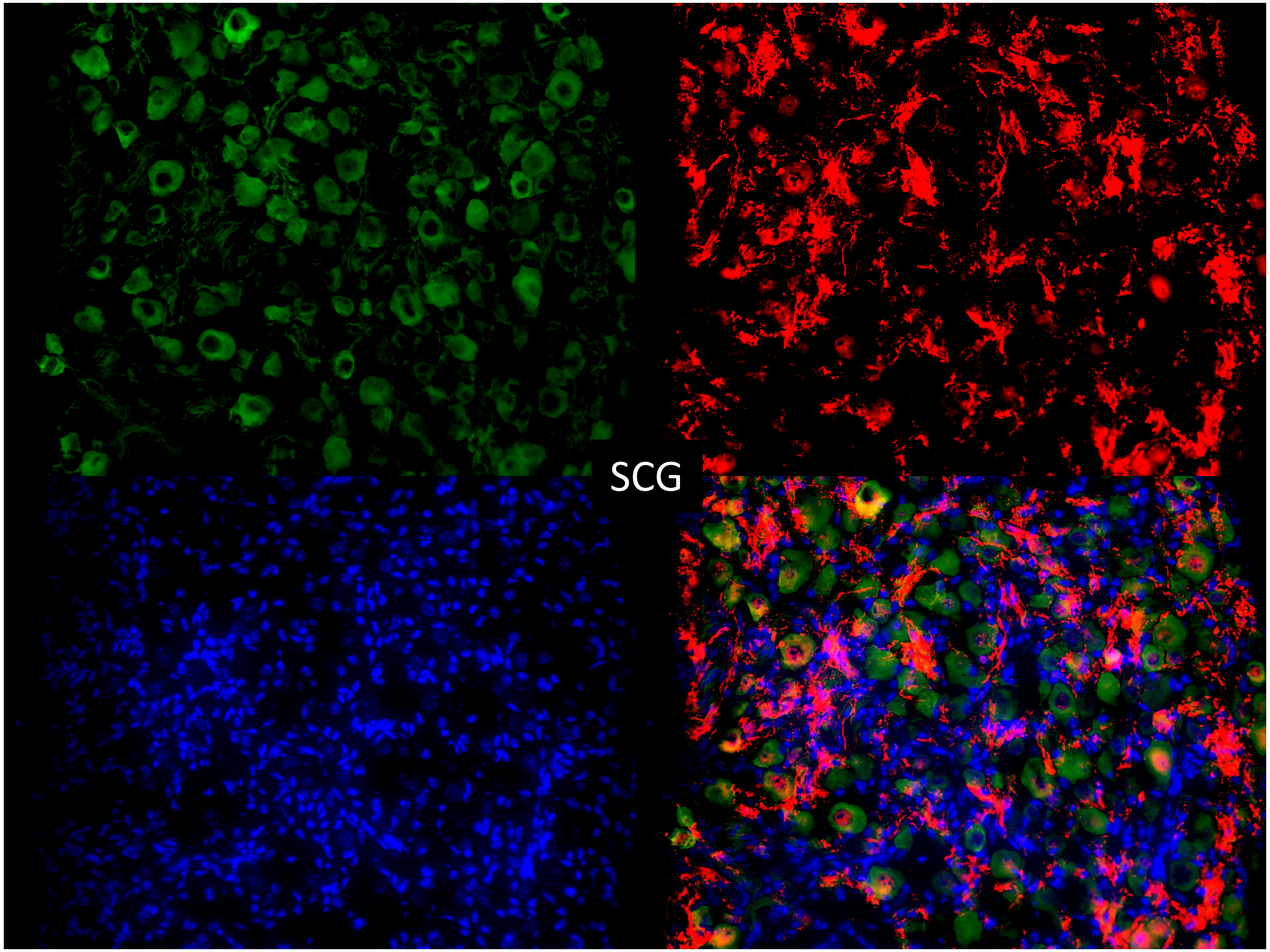

DAPI

TH

nNOS

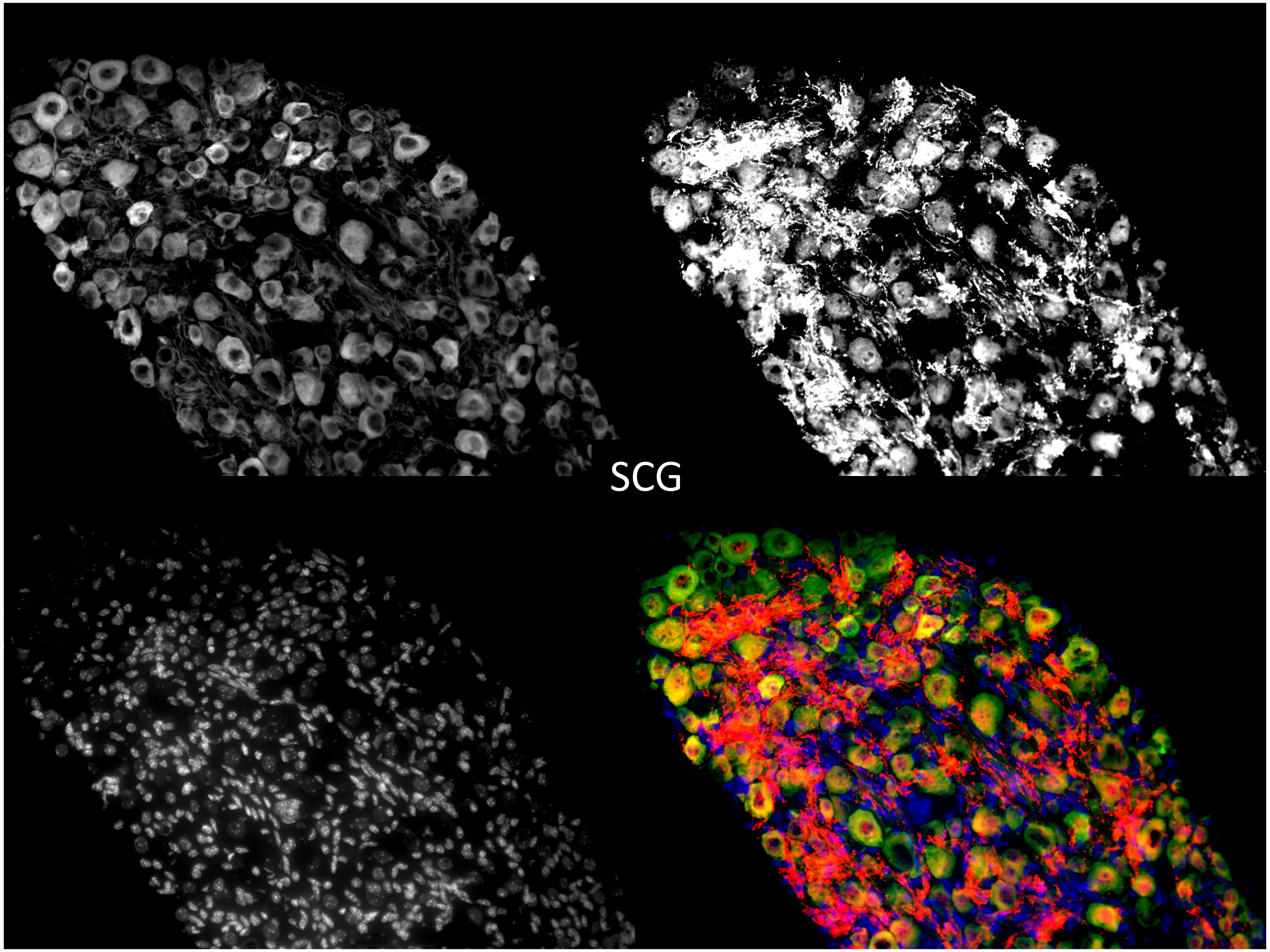

DAPI

TH

nNOS

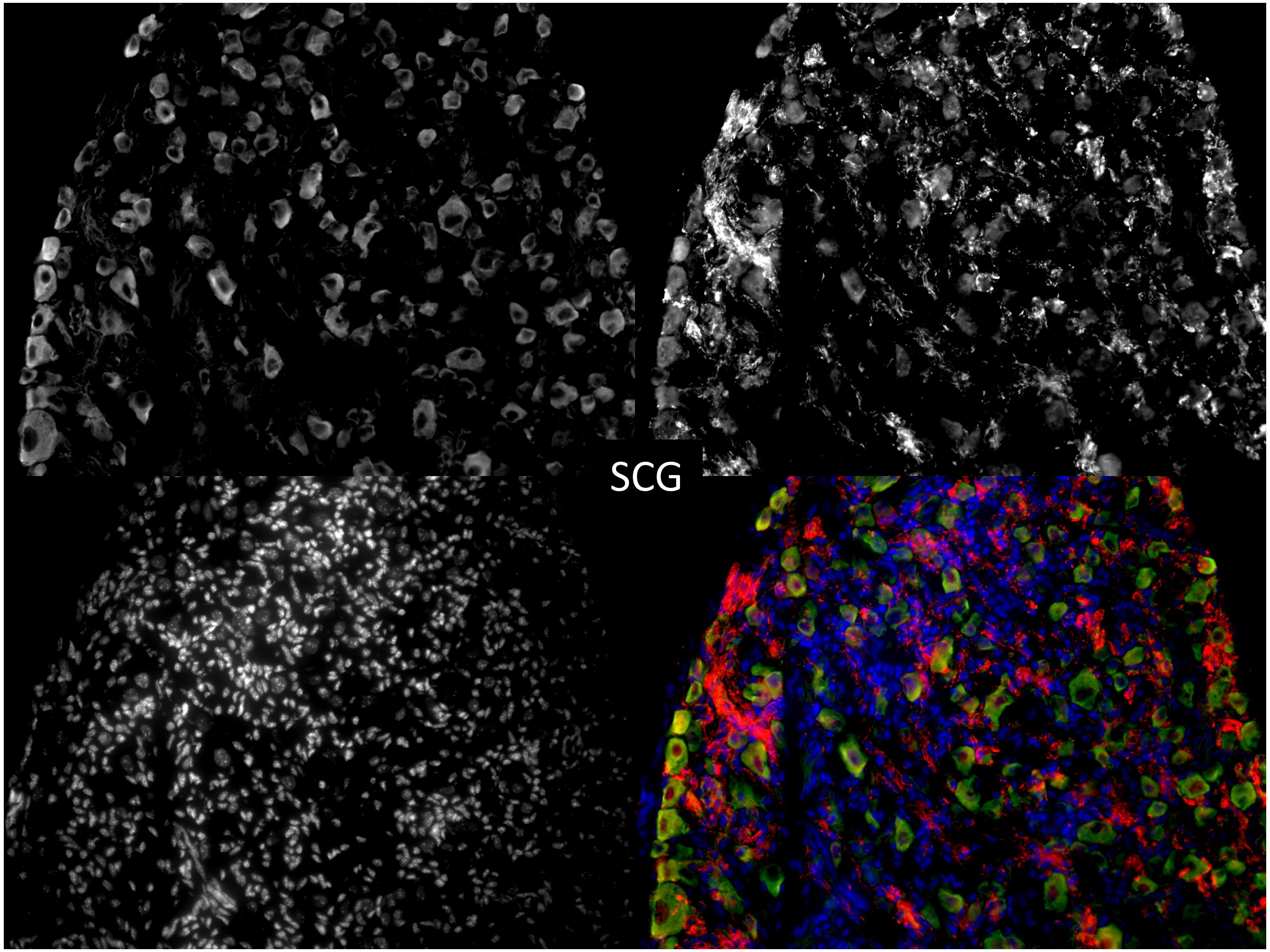

DAPI

TH

nNOS

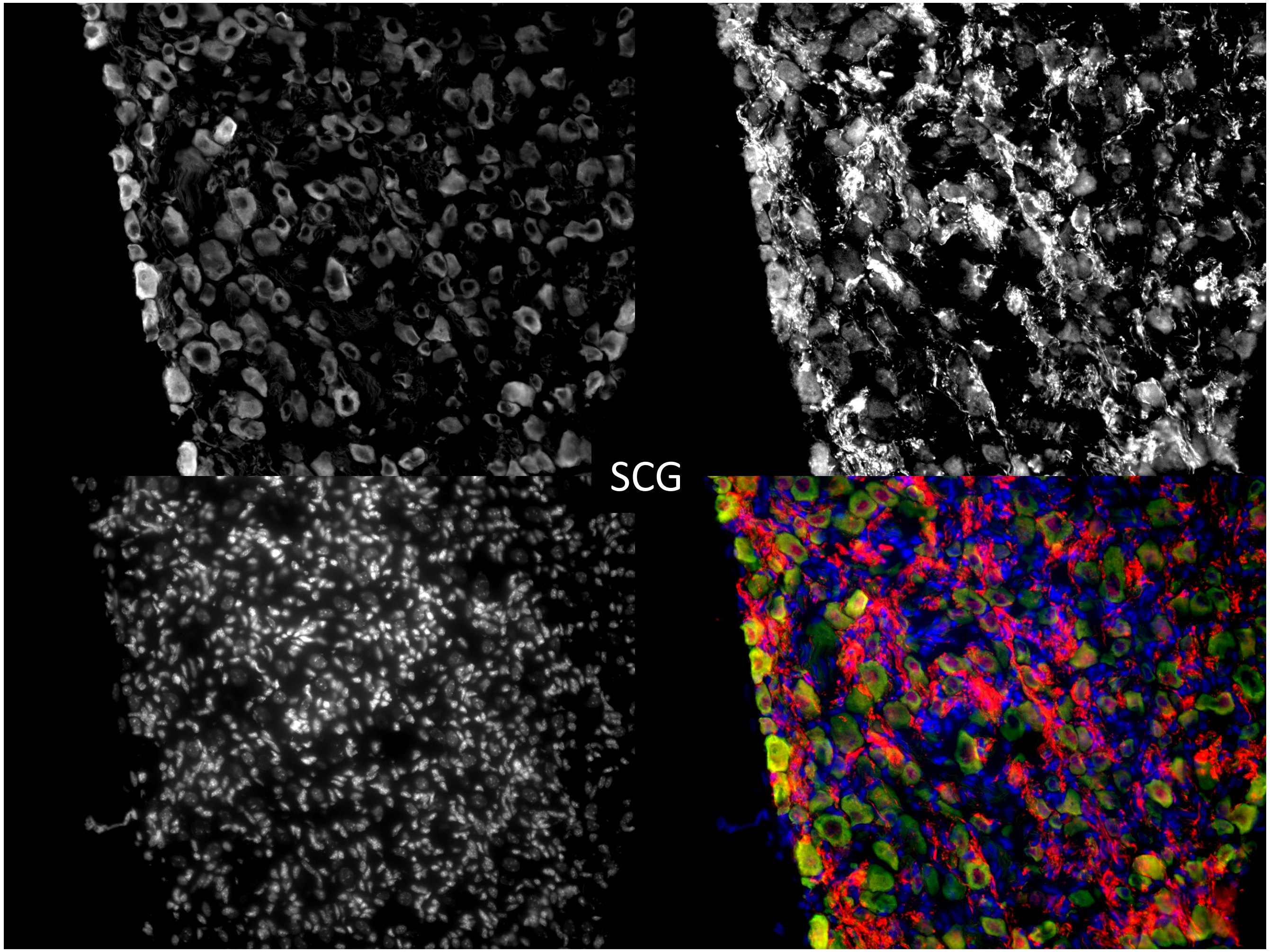

DAPI

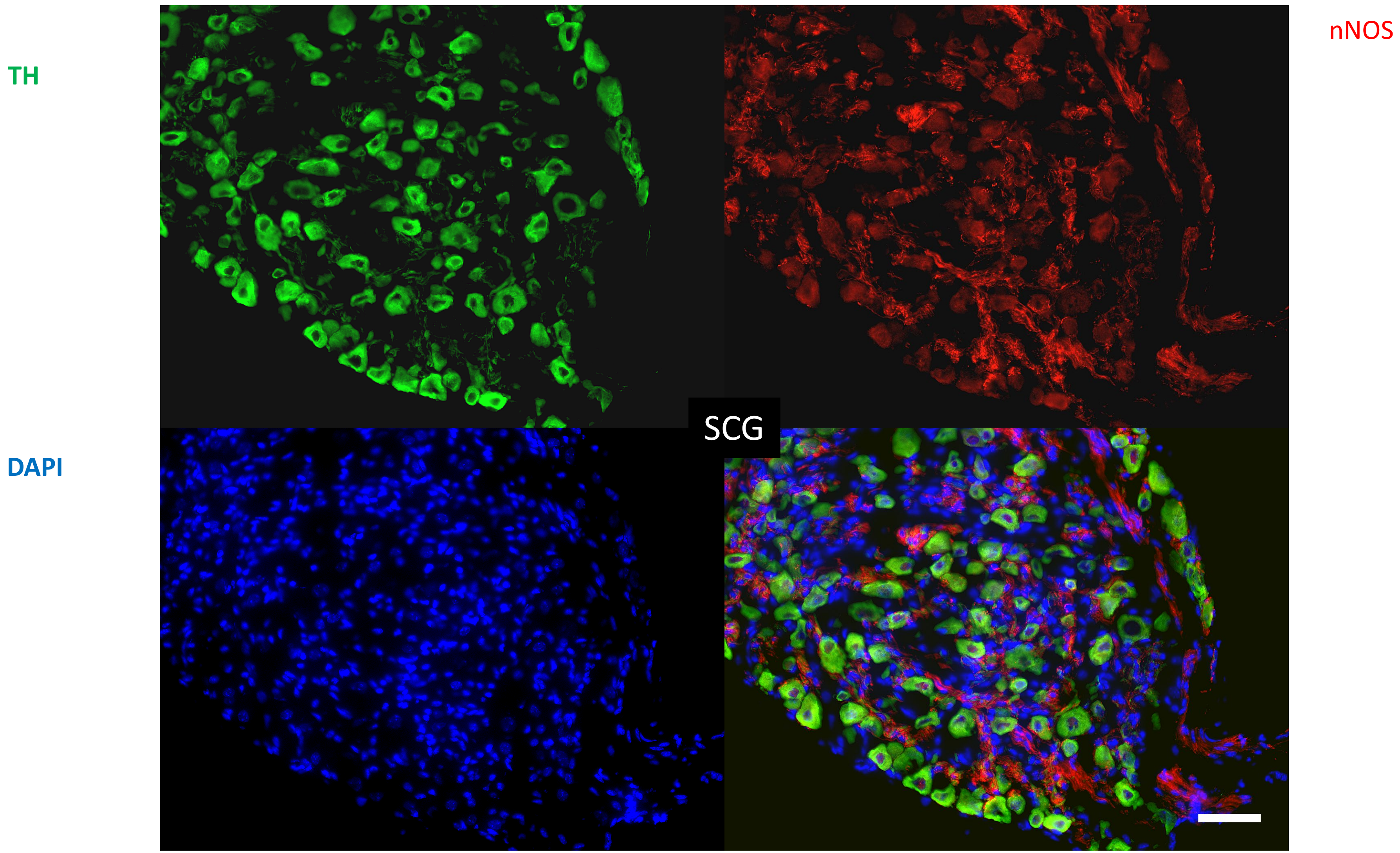
TH
nNOS
DAPI
SCG

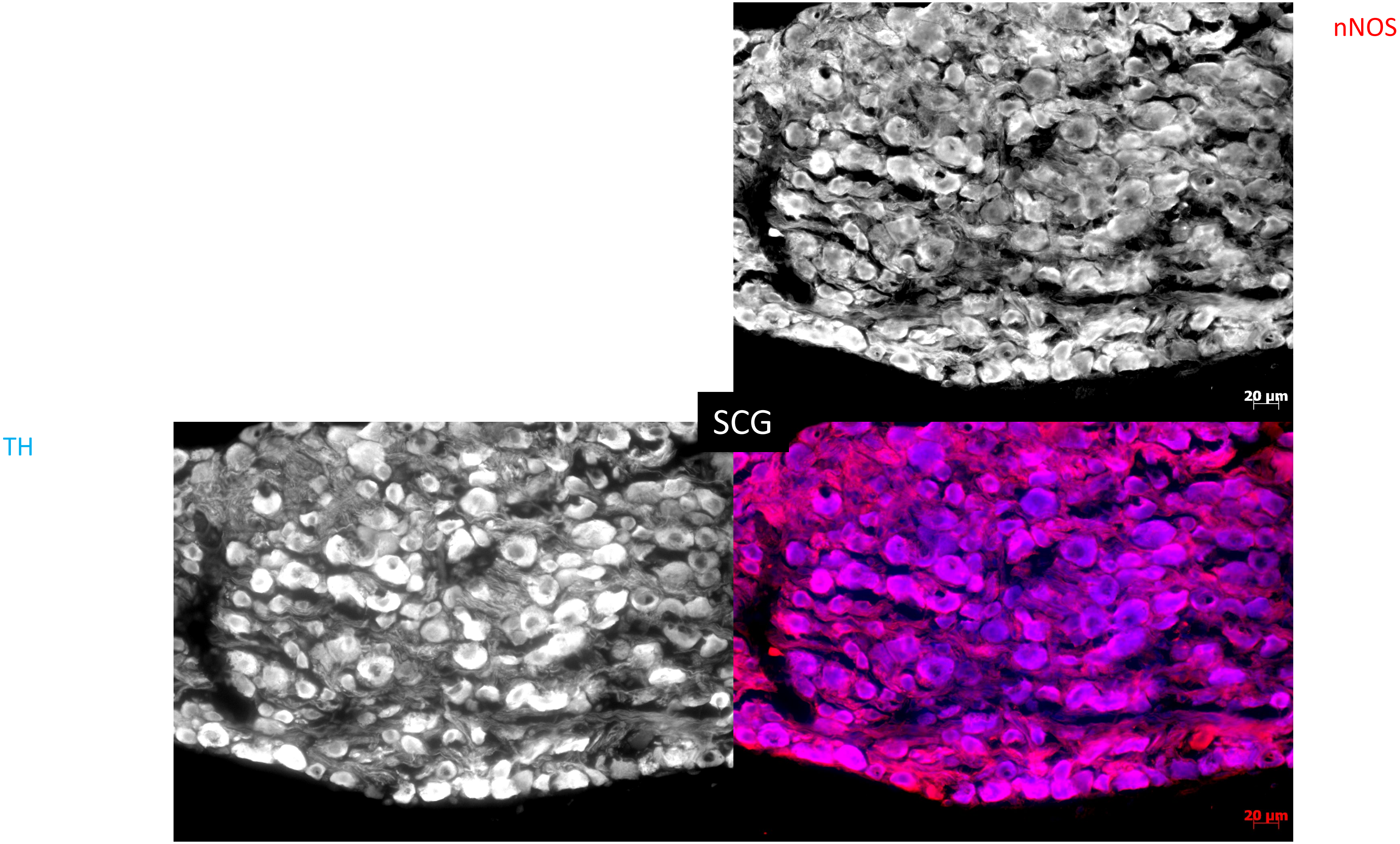
nNOS
TH
SCG
20 µm
20 µm

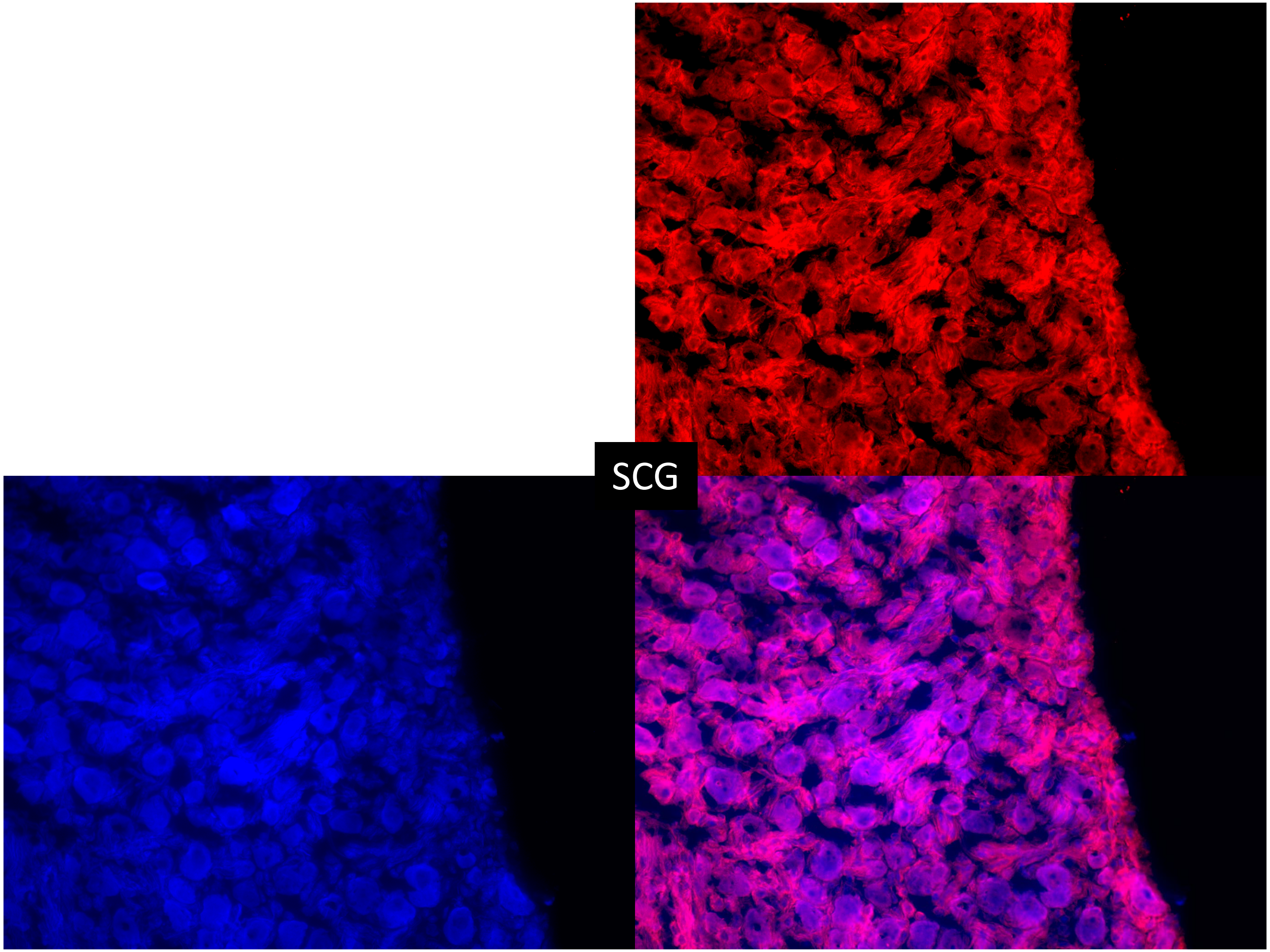
nNOS
SCG
TH

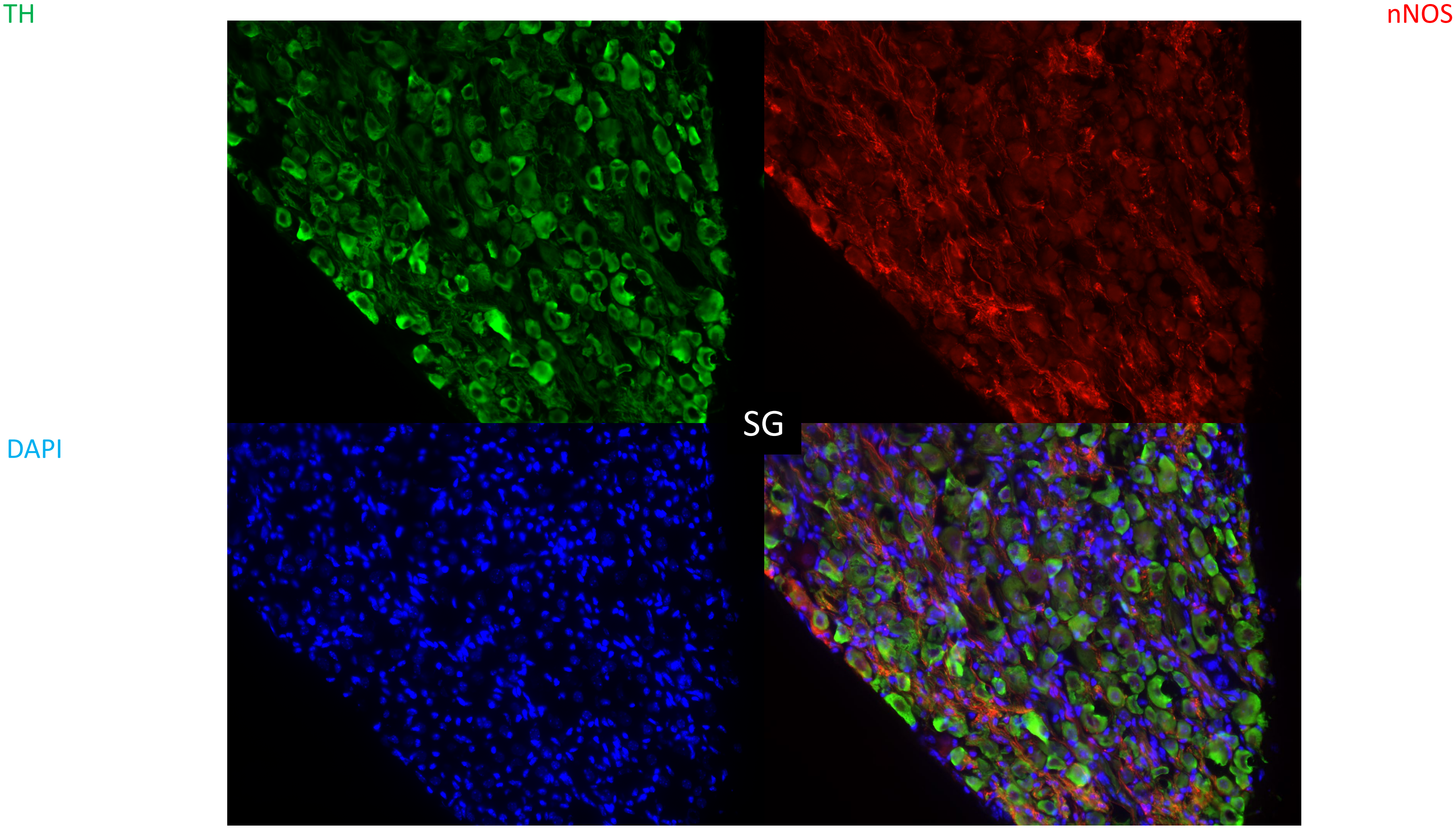
TH
nNOS
DAPI
SG

TH

nNOS

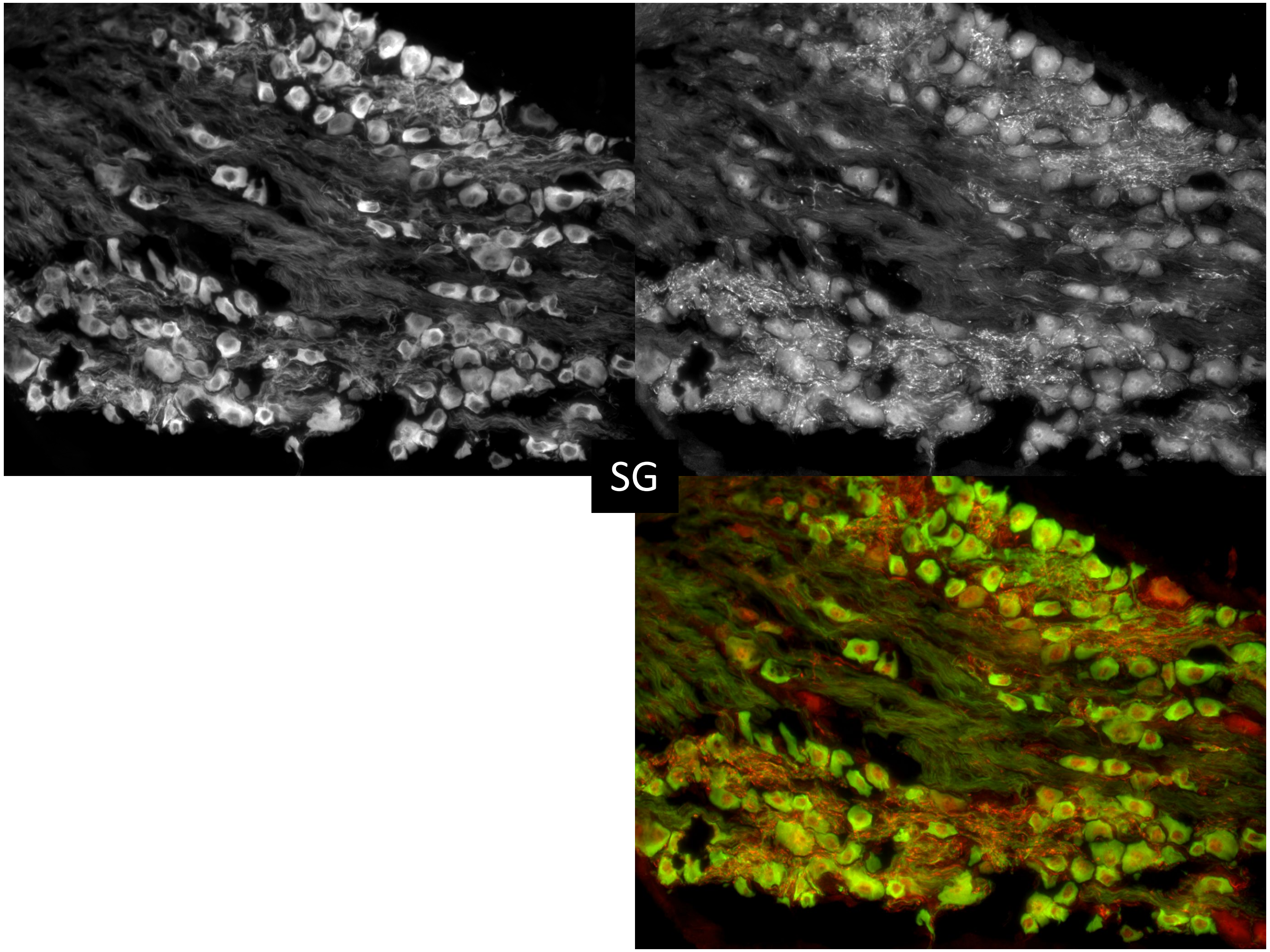

DAPI

TH

nNOS

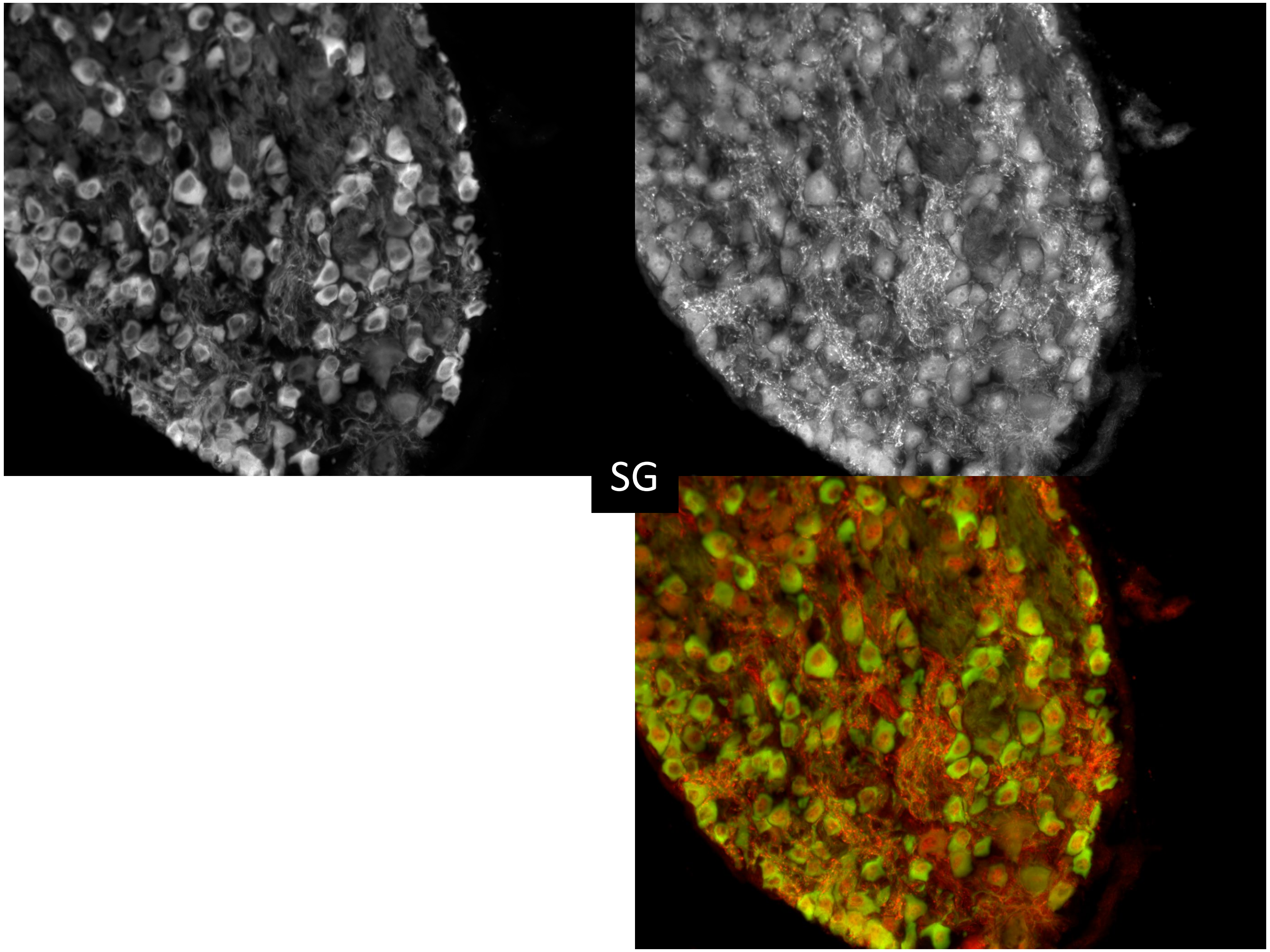

DAPI

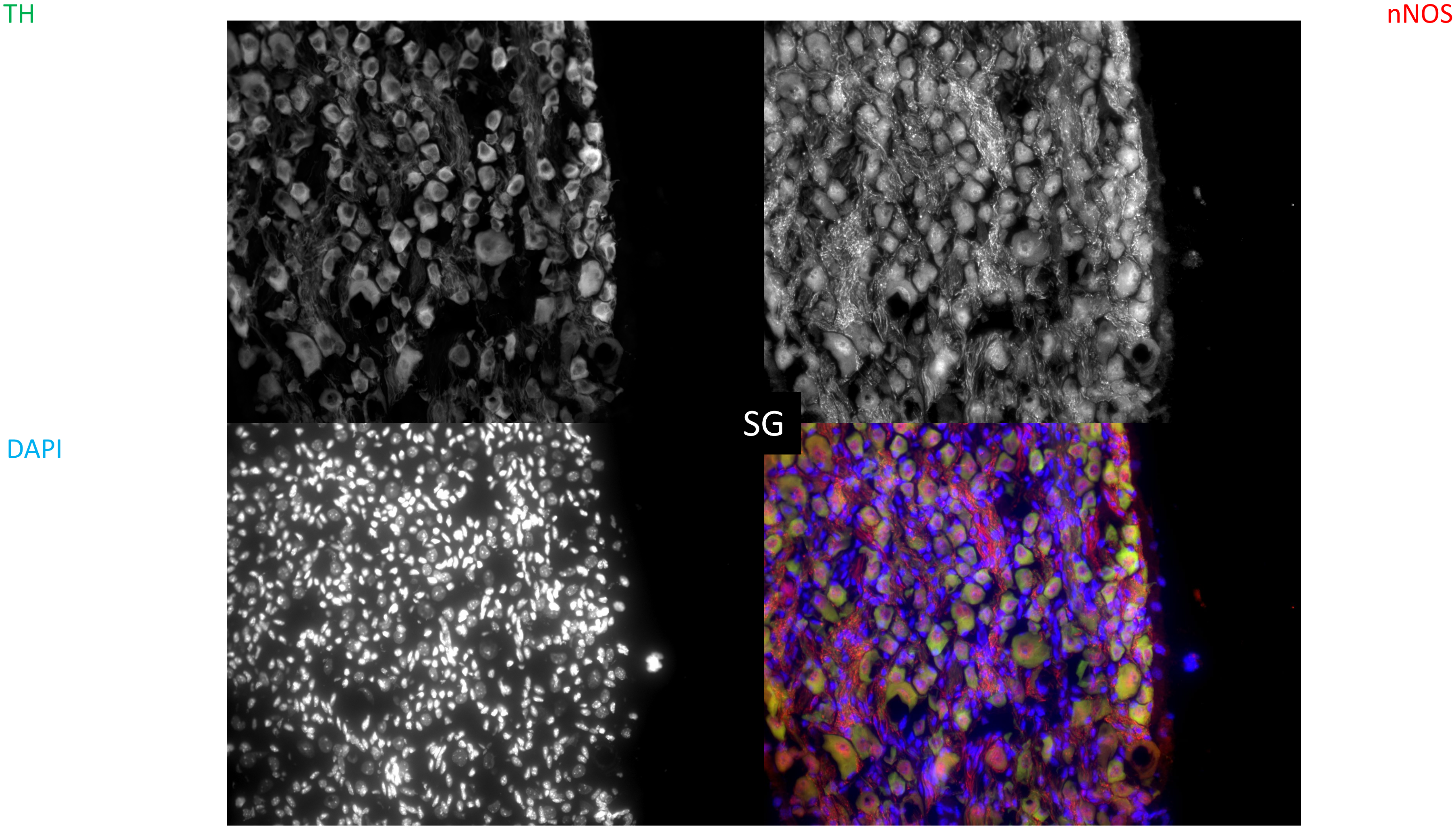
TH
nNOS
DAPI
SG

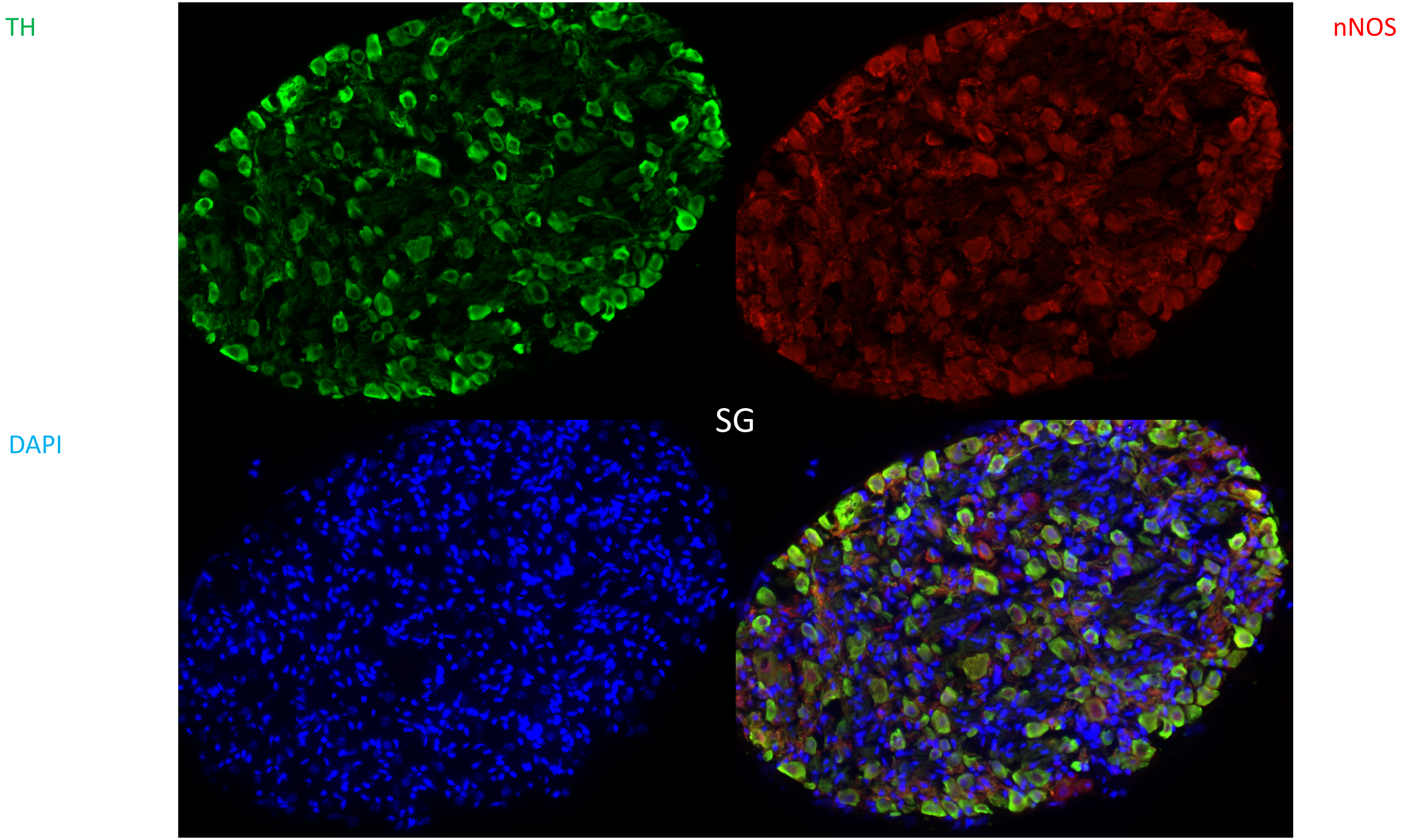
TH
nNOS
SG
DAPI

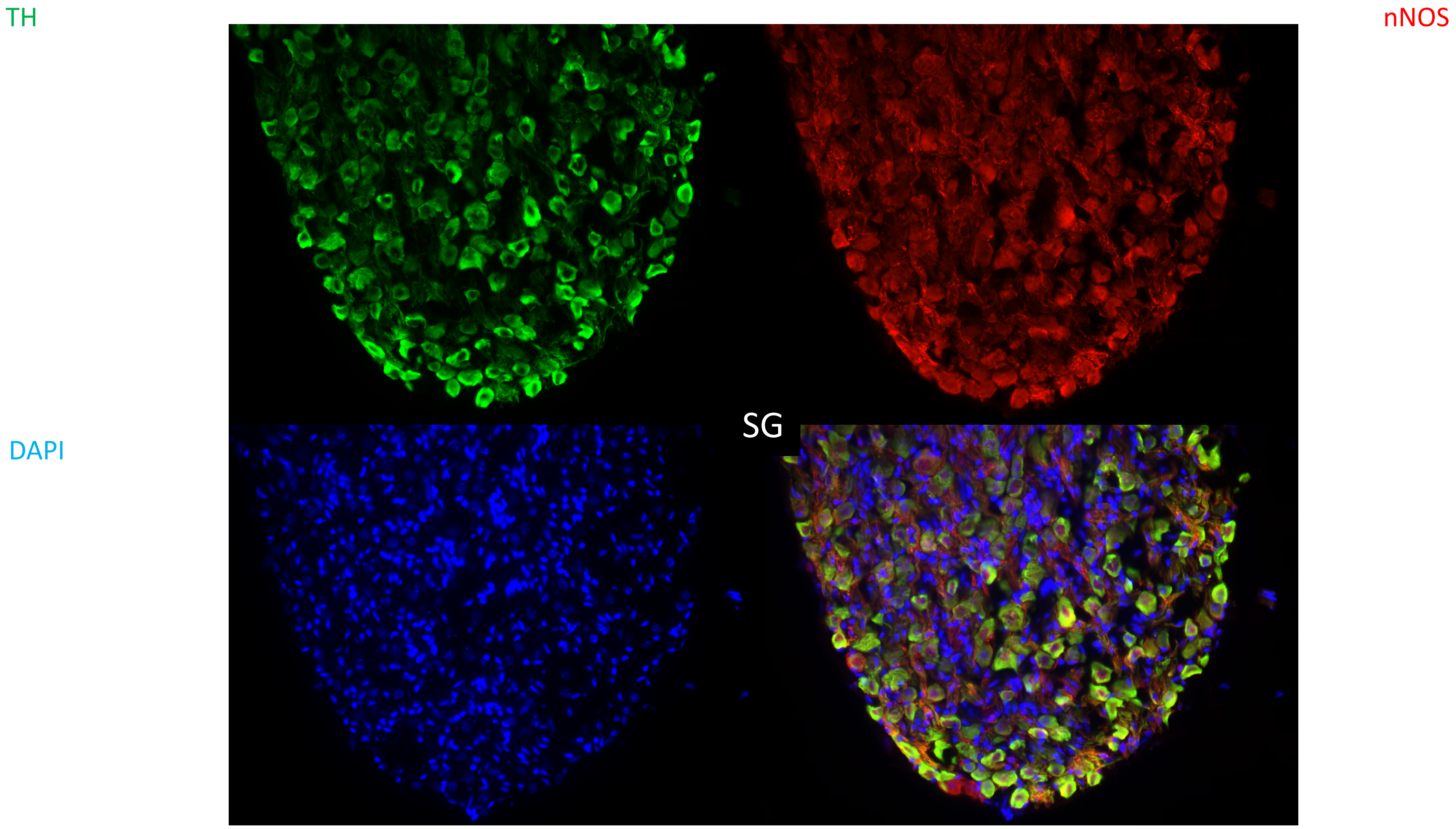
TH
nNOS
DAPI
SG

TH

nNOS

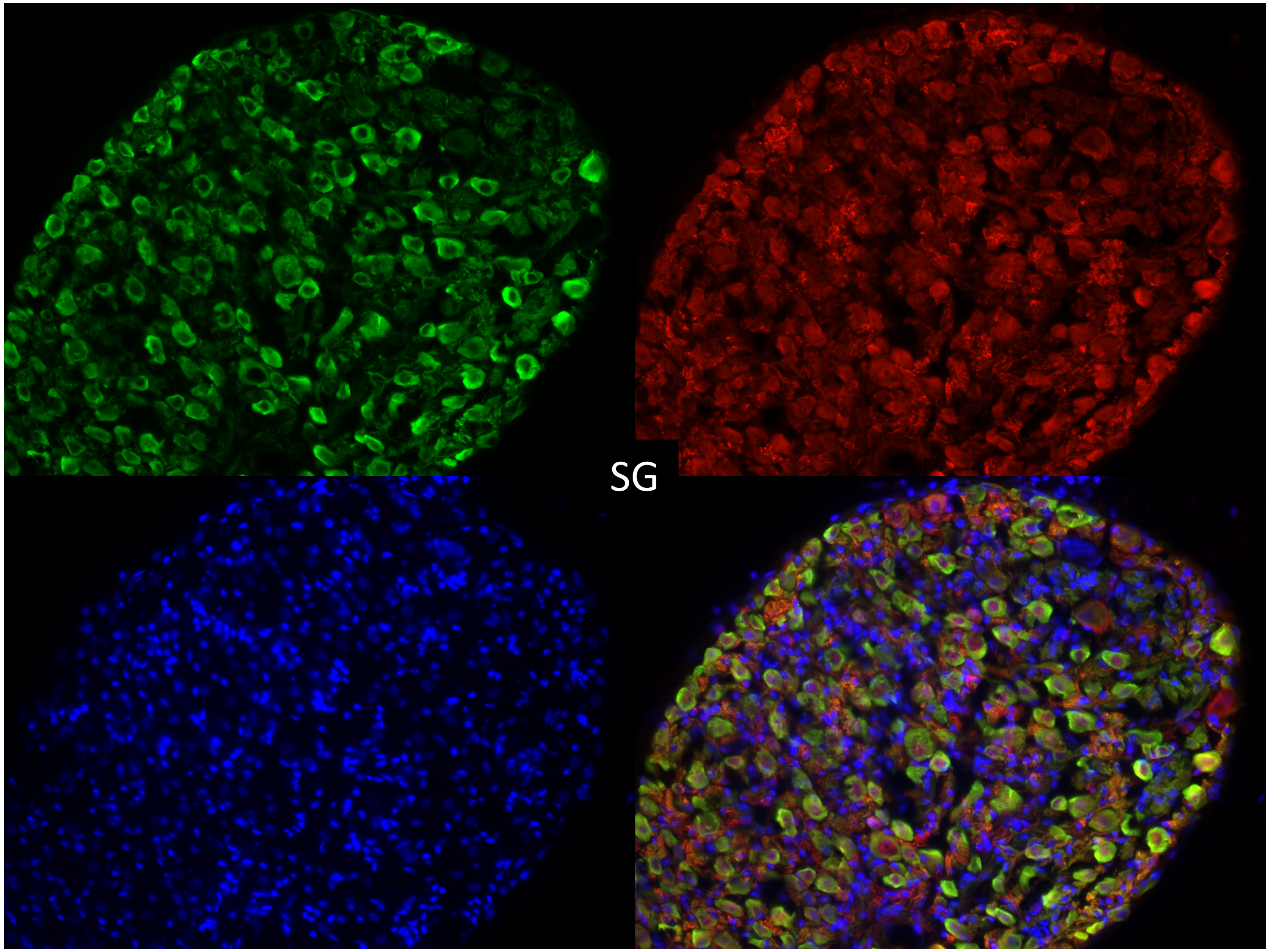

DAPI

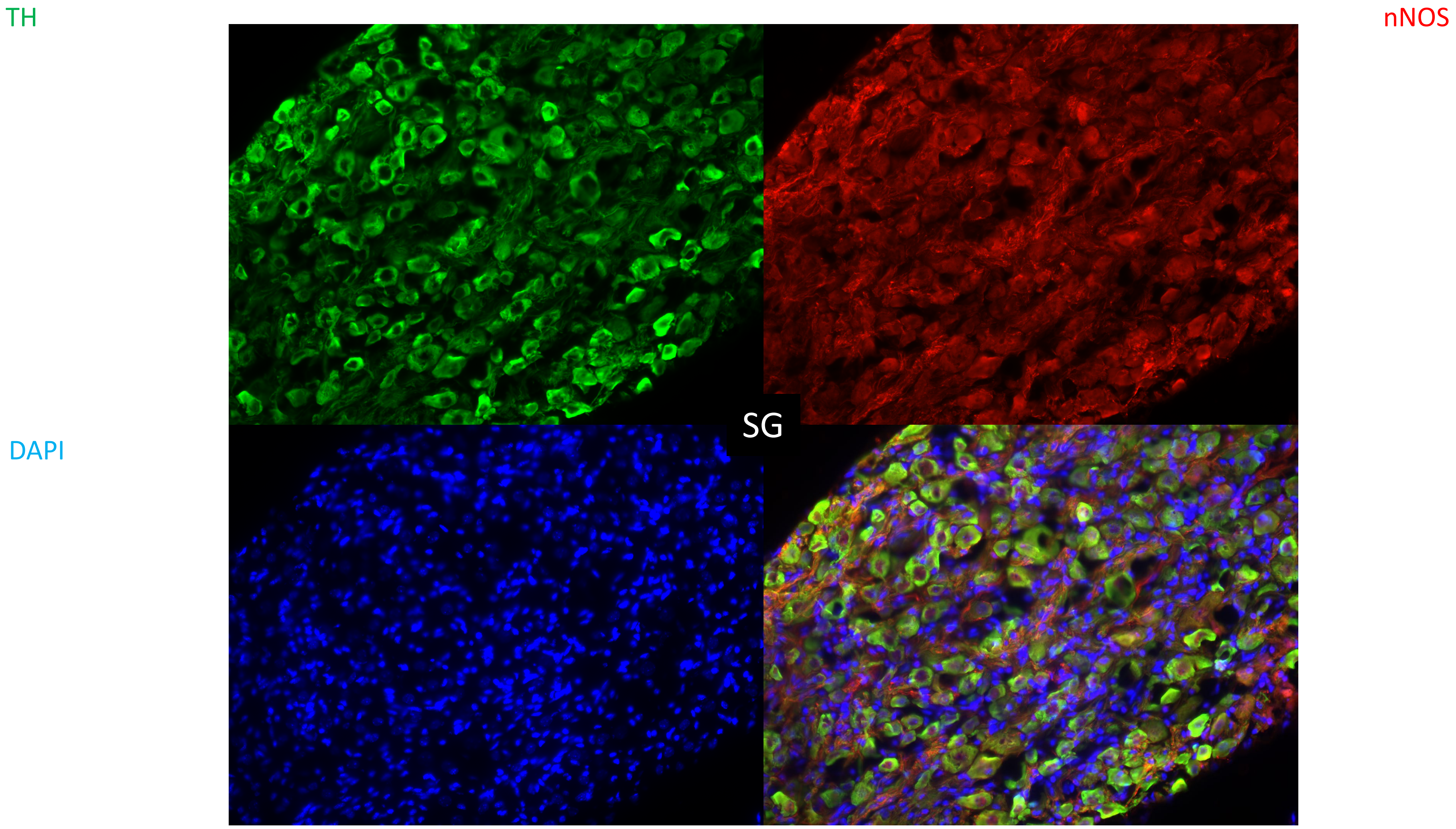
TH
nNOS
DAPI
SG

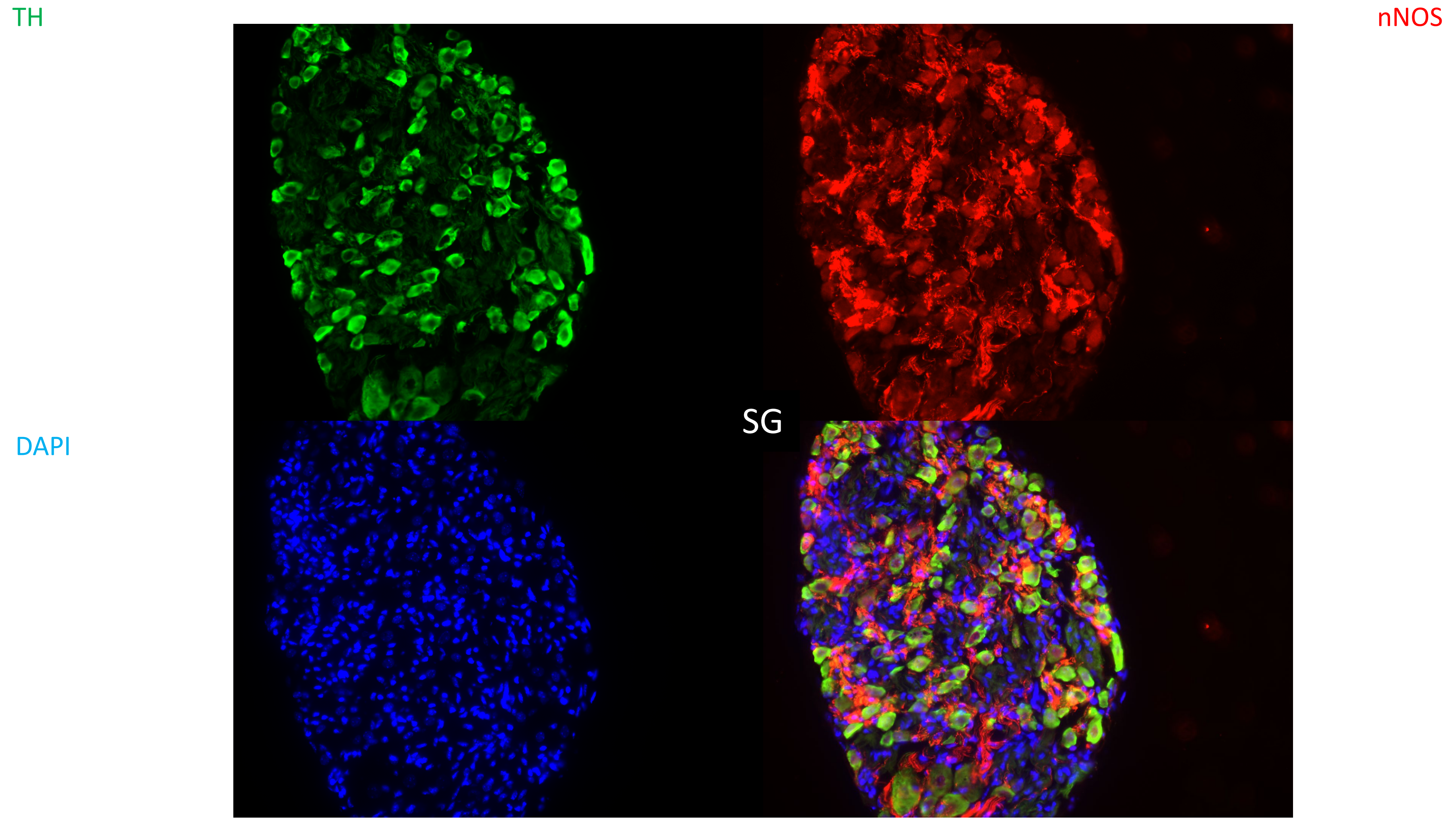
TH
nNOS
DAPI
SG

TH

nNOS

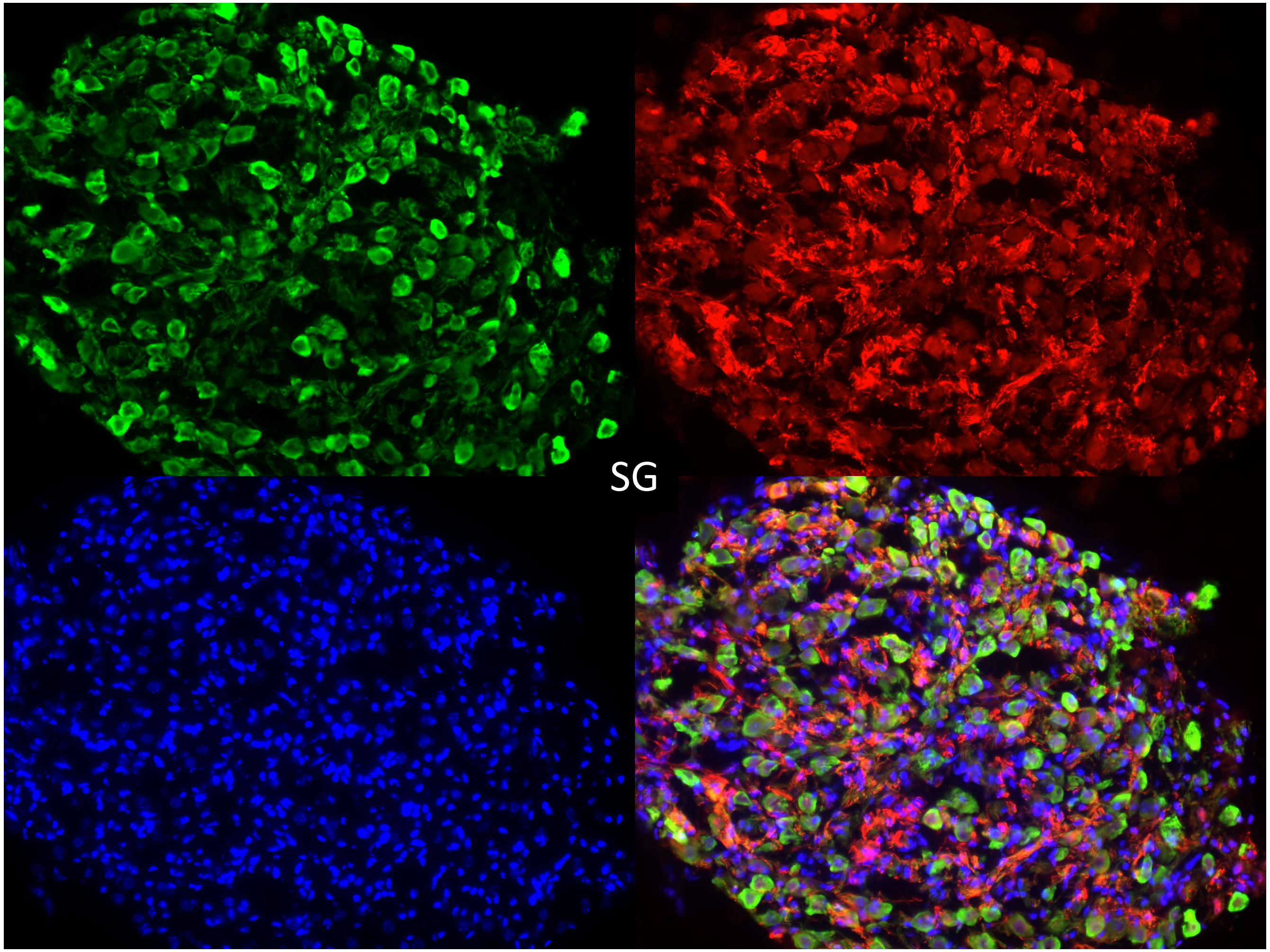

DAPI

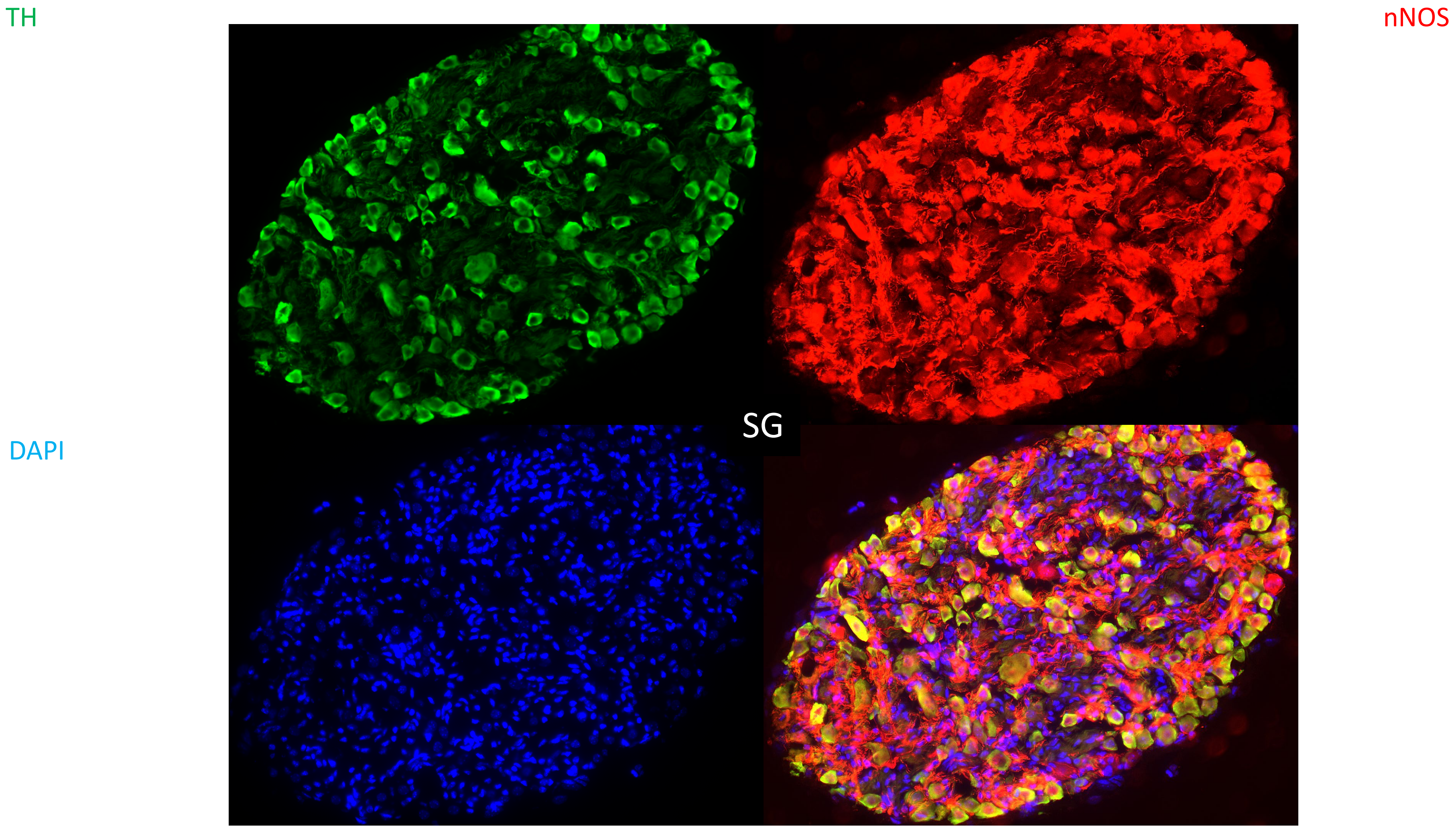
TH
nNOS
SG
DAPI

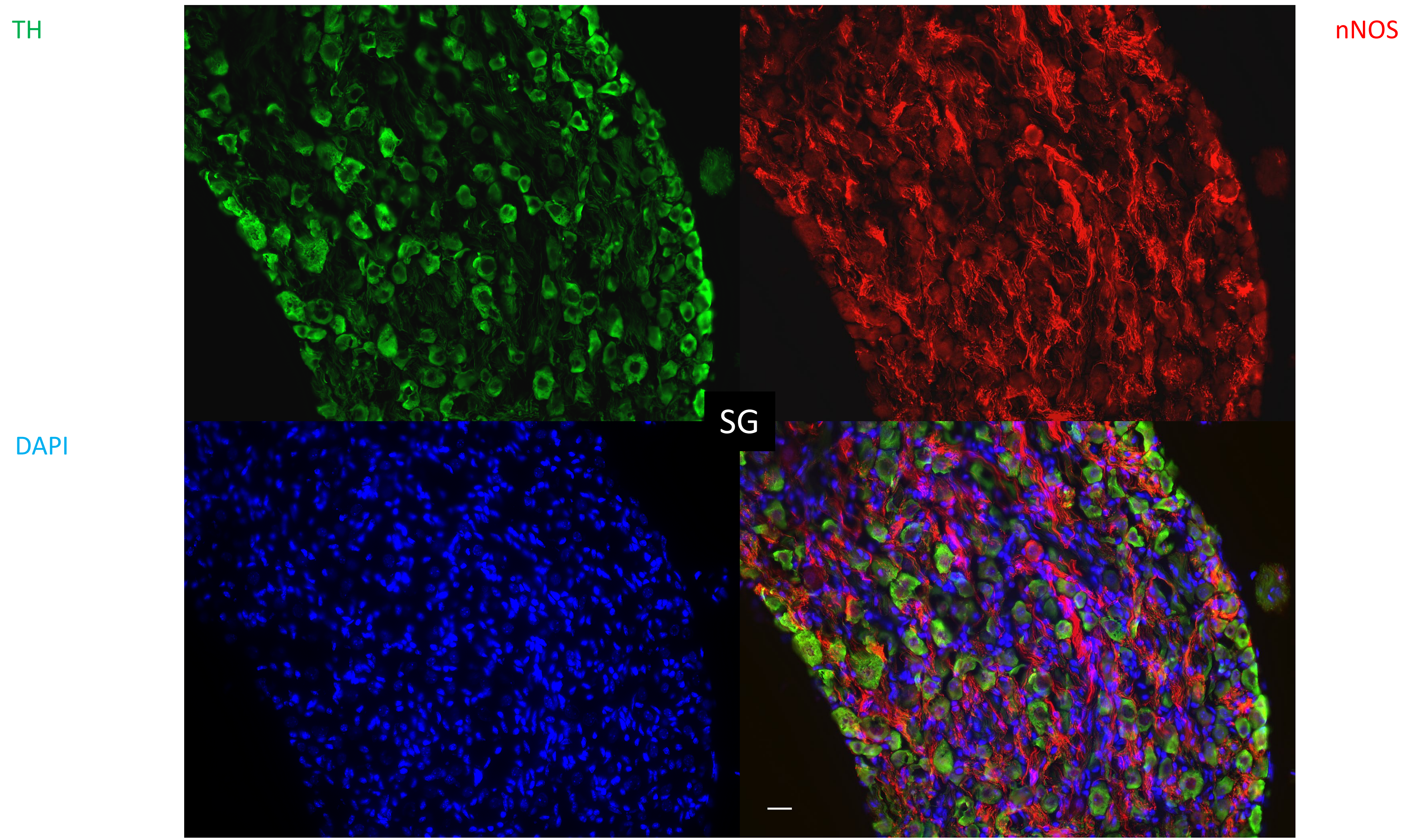
TH
nNOS
DAPI
SG

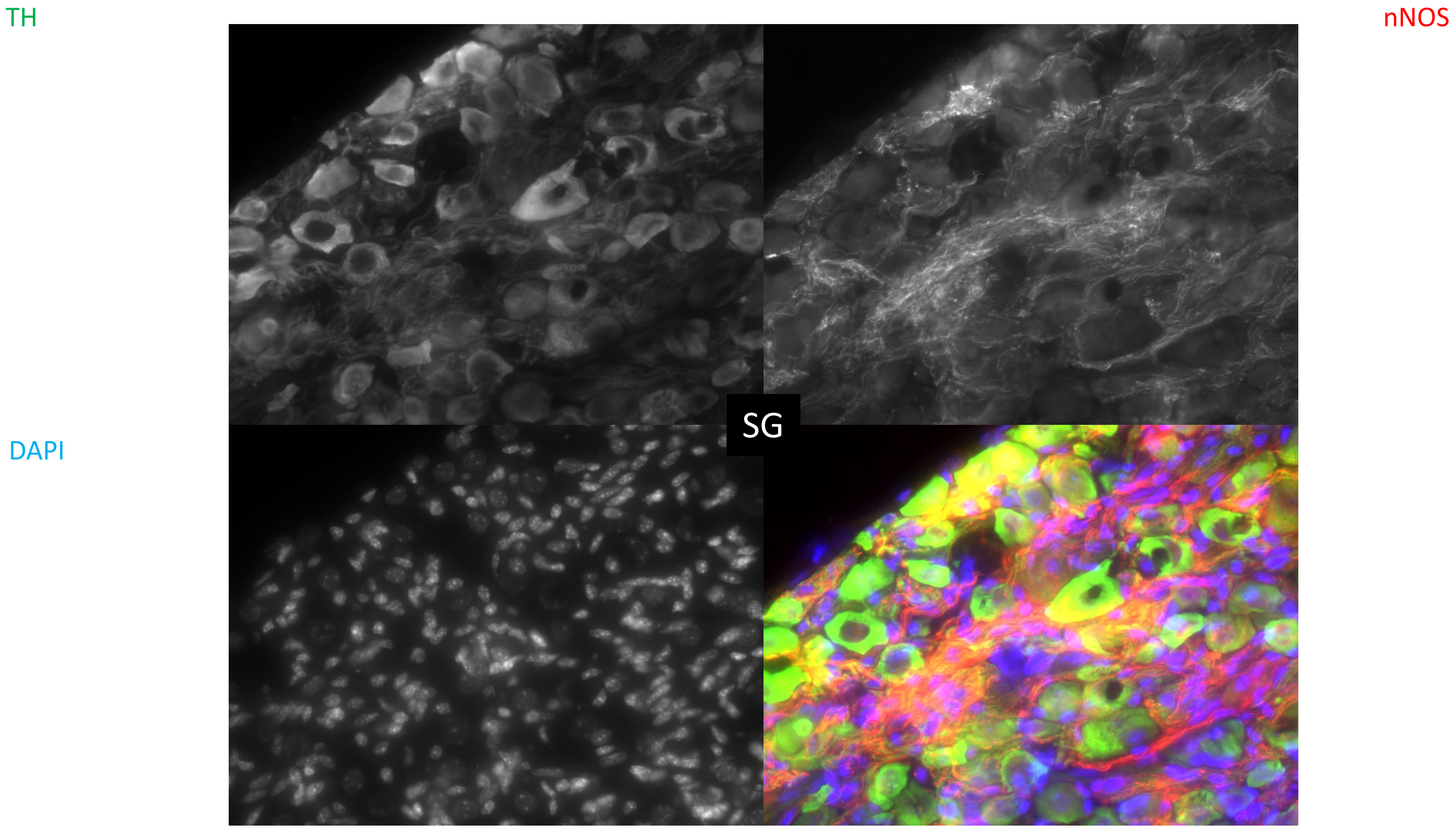
TH
nNOS
DAPI
SG
