# Supplementary material for: STIM1 and ORAI1 form a novel cold transduction mechanism in sensory and sympathetic neurons
Source: EMBO J. 2022 Dec 16;42(3):e111348. doi: 10.15252/embj.2022111348 (PMC9890232; doi:10.15252/embj.2022111348)
Supplement: Supplementary file 12 [file EMBJ-42-e111348-s006.pdf]

THE EMBO JOURNAL

STIM1 and ORAI1 form a novel cold transduction mechanism in sensory and sympathetic neurons

Tamara Buijs, Bruno Vilar, Chun-Hsiang Tan and Peter McNaughton
DOI: 10.15252/embj.2022111348

Corresponding author(s): Peter McNaughton (peter.mcnaughton@kcl.ac.uk)

Review Timeline:

Submission Date:	13th Apr 22
Editorial Decision:	13th May 22
Revision Received:	24th May 22
Editorial Decision:	27th May 22
Revision Received:	1st Sep 22
Editorial Decision:	16th Sep 22
Revision Received:	25th Oct 22
Accepted:	10th Nov 22

Editor: William Teale

Transaction Report:

(Note: With the exception of the correction of typographical or spelling errors that could be a source of ambiguity, letters and reports are not edited. Depending on transfer agreements, referee reports obtained elsewhere may or may not be included in this compilation. Referee reports are anonymous unless the Referee chooses to sign their reports.)

Dear Prof. McNaughton,

Thank you again for sharing your work and the submission of your manuscript entitled "STIM1 and ORAI1 form a novel cold transduction mechanism in sensory and sympathetic neurons (EMBOJ-2021-111348) to The EMBO Journal. Your manuscript was sent to two reviewers for evaluation; we have now received reports from both of them, which I enclose below. As you will see, however, your manuscript failed to garner the level of support from the referees for publication in EMBO Journal.

Your work shows that reducing STIM1 expression in neurons blocks the ability to sense cold. You then develop and test the hypothesis that store-operated calcium entry is a mechanism though which cells sense and respond to noxious cold. However, the referees' concerns, which focused on an underlying lack of experimental support for your key observations, were too substantial for us to ignore.

While we cannot pursue this manuscript further, we encourage you to transfer your study to our not-for-profit open-access sister journal, Life Science Alliance (LSA). We shared your manuscript and the accompanying reviews with LSA Executive Editor, Eric Sawey, who is interested in these findings, and would like to invite further consideration of this manuscript at LSA, revised to address the Reviewer comments, excluding Reviewer 2's point #8. We understand that such a revision might need to be re-reviewed, in which case, Dr. Sawey will walk the Reviewers through our transfer process. We encourage you to use the link below to transfer your manuscript to LSA. You do not need to revise the manuscript before transferring it to LSA. If you transfer, Dr. Sawey will then email you an invitation to revise and resubmit, listing the same revision requests as mentioned above. Please feel free to reach out at e.sawey@life-science-alliance.org if you have any questions about the LSA journal, the transfer process or the revisions requested.

I am sorry we cannot be more positive on this occasion. I nevertheless hope that (whichever direction this work takes towards publication) you will find our referees' comments helpful.

Yours sincerely,

William Teale

William Teale, PhD
Editor
The EMBO Journal
w.teale@embojournal.org

Referee #1:

The manuscript entitled, "STIM1 and ORAI1 form a novel cold transduction mechanism in peripheral sensory and sympathetic neurons" provides evidence that store-operated Ca2+ entry may be a cold sensor. The strongest evidence by far are the observations that STIM1 RNAi eliminates cold sensitivity in SCG neurons and the fact that STIM and Orai colocalize in HEK293 cells overexpressing the proteins in response to cold. However, I also have some major concerns that need to be reconciled. Perhaps the most serious issue is that STIM1 and Orai1 are expressed in every cell except for erythrocytes. As such, it is unclear why novel cold sensitivity observed in a neuronal subset could be accounted for by STIM1 and Orai1 expression. I recognized that this issue was brought up in the discussion, but it should be raised in the introduction and directly addressed within the study. Similarly, the fact that STIM1 activation was previously shown to occur in response to heating and cooling cells should be discussed in the introduction and not at the end, as it is directly relevant to whether or not STIM/Orai are temperature sensitive. The extent to which the current findings reflect a new function of STIM1 should also be addressed.
Major Comments:
1. Although YM58483 does block Orai1, it blocks TRPC channels at similar concentrations (He et al, 2005) and activates TRPM4 at much lower concentrations (Takezawa et al, 2006). As such, these data are insufficient to demonstrate that Orai1 is cold-activated. Although I recognize and acknowledge that MRS1845 was also used. However, I have not seen any recent papers using this pharmacological agent; irrespective I am not aware of ANY truly specific inhibitors of Orai1.
2. The STIM1 siRNA experiments in fig 4 are crucial components of this study. Although somewhat convincing, the use of a scrambled control is necessary to show that this is not a transfection artifact.
3. The authors seem a little confused about where STIM1 is located in cells based on writing in both the introduction and in the description of figure 5. Although there is a small percentage of STIM1 in the plasma membrane, store depletion does not cause it to translocate from the ER to the PM. Rather, it moves within the ER to areas of close ER-PM apposition. There are numerous primary articles and reviews establishing this point.
To that point, it is notable that STIM1 relocalization in response to cold is to areas where Orai1 is also moving to. In truth, these sites are ultimately controlled by STIM1, not Orai1, since STIM1 localization is space limited to these ER-PM junctions while

Orai1 is free to diffuse through the PM. TIRFM can be done at many levels; these images suggest to me that this is a relatively wide TIRF image. They are also highly suggestive that STIM1 is cold sensitive.
4. Given the central claim of this paper, a clear and direct demonstration that cold does not cause emptying of ER Ca2+ stores is needed by comparing ER Ca2+ release using thapsigargin or caffeine.
Minor Comment:
1. Bottom paragraph of page 10, fig 3 is referenced when fig 4 is intended.

Referee #2:

In this manuscript, Buijs and coworkers study the phenomenon of cold-induced calcium (Ca2+) increases in neurons, HEK293 and PC12 cells. Using pharmacological, fura-2 and electrophys approaches, the authors suggest that somatosensory and sympathetic neurons have evolved two distinct mechanisms of cold-induced cytosolic Ca2+ increases, both dependent on extracellular Ca2+: 1) via CaV channels and 2) via SOCE. The authors provide evidence that the SOCE-related mechanisms is dependent on STIM1 and Orai1, but not dependent on ER Ca2+ levels. Finally, the authors suggest that the phenomenon the describe may explain cold-induced vasodilation, but provide no experimental evidence for this.

I have several concerns that preclude publication of this manuscript in the current form:

1) Fluorescence is a temperature-dependent phenomenon. The authors should show how temperature separately affects the 340nm and 380nm Fura-2 signals (i.e. show some raw traces) to demonstrate that the ratios reported are not simply due to temperature-dependent fluorescence changes.

2) It is not clear why cold ramps were performed from 31 deg C to 4 deg C; what happens if the cold ramp starts at a more physiological temperature (i.e. 37 deg)?

3) With cells bathed in HEPES buffer, how can the authors exclude that the apparent temperature-dependent affects are not due to change in bathing medium pH given the deltapKa/deltaT of HEPES?

4) Page 10 - I don't think the appropriate figure is being referred to (should this be Fig 4d-g)? Similarly, further down that paragraph - should this be Figure 4d?

5) Figure 5b - the puncta formation due to cold is not convincing. It appears that those puncta are pre-existing at baseline. Further, the # of puncta per cell (panel b) does not seem to be consistent with the representative images shown in panel A. As a control, the authors should show how puncta form in response to ER Ca2+ depletion.

6) How can the authors reconcile that decreased number of STIM puncta at colder temperature with increased SOCE? Do the authors believe that the activation of Orai1 channels due to cold occurs independent of STIM1? If so, what would be the mechanism?

7) Previous work by Patapoutian group showed that increased temperatures (from 37 to above 40 deg C) induce STIM1 activation, and returning to lower temperatures causes profound activation of Orai1 channels, independent of ER Ca2+ levels. More specific discussion of this previous work and how it may related to the present observations is needed.

8) Previous work showed that nitric oxide suppresses SOCE and CRAC currents; how do the authors reconcile those previous observations with their supposition that the cold-induced SOCE drives CVID via nNOS activity? The authors should consider adding a model diagram with respect CVID signaling in the Discussion, which ties together all the observations from the present study and previous studies. Alternatively, a model diagram that ties together all previous temperature-induced cytosolic Ca2+ stimulation studies with the present observations would be very useful.

9) In general, the referencing in the manuscript appears to be dubious. There are several instances where the authors are referencing STIM1 and/Orai1 observations prior to 2005/2006, when in fact these proteins were not identified until 2005/2006. The authors need to carefully check all references and ensure they are referencing the primary publication.

** As a service to authors, EMBO Press provides authors with the possibility to transfer a manuscript that one journal cannot offer to publish to another EMBO publication or the open access journal Life Science Alliance launched in partnership between EMBO Press, Rockefeller University Press and Cold Spring Harbor Laboratory Press. The full manuscript and if applicable, reviewers' reports, are automatically sent to the receiving journal to allow for fast handling and a prompt decision on your manuscript. For more details of this service, and to transfer your manuscript please click on Link Not Available. **
Please do not share this URL as it will give anyone who clicks it access to your account.

Reply to: EMBOJ-2022-111348 Decision Letter

"STIM1 and ORAI1 form a novel cold transduction mechanism in peripheral sensory and sympathetic neurons"

Referee #1:

The manuscript entitled, "STIM1 and ORAI1 form a novel cold transduction mechanism in peripheral sensory and sympathetic neurons" provides evidence that store-operated Ca2+ entry may be a cold sensor. The strongest evidence by far are the observations that STIM1 RNAi eliminates cold sensitivity in SCG neurons and the fact that STIM and Orai colocalize in HEK293 cells overexpressing the proteins in response to cold. However, I also have some major concerns that need to be reconciled. **Perhaps the most serious issue is that STIM1 and Orai1 are expressed in every cell except for erythrocytes.** As such, it is unclear why novel cold sensitivity observed in a neuronal subset could be accounted for by STIM1 and Orai1 expression. I recognized that this issue was brought up in the discussion, but it should be raised in the introduction and directly addressed within the study. Similarly, the fact that STIM1 activation was previously shown to occur in response to heating and cooling cells should be **discussed in the introduction and not at the end**, as it is directly relevant to whether or not STIM/Orai are temperature sensitive. **The extent to which the current findings reflect a new function of STIM1 should also be addressed**.

Textual changes to address these comments will be made

Major Comments:

1. Although YM58483 does block Orai1, it blocks TRPC channels at similar concentrations (He et al, 2005) and activates TRPM4 at much lower concentrations (Takezawa et al, 2006). As such, these data are insufficient to demonstrate that Orai1 is cold-activated. Although I recognize and acknowledge that MRS1845 was also used. However, I have not seen any recent papers using this pharmacological agent; irrespective I am not aware of ANY truly specific inhibitors of Orai1.

We agree that pharmacological evidence is always open to the criticism that effects could be off-target and for this reason we used two blockers of different structure that have both been shown in previous studies to inhibit Orai1. However, as made clear in the MS, we take the pharmacological evidence only as a guide to future studies in which downregulation of STIM1 in neurons and measures using TIRF microscopy of translocation of STIM1 and formation of ORAI1 puncta provide much stronger evidence for the involvement of STIM1 and ORAI1. We will make textual changes to reinforce this point.

2. The STIM1 siRNA experiments in fig 4 are crucial components of this study. Although somewhat convincing, the use of a scrambled control is necessary to show that this is not a transfection artifact.

We will perform further experiments in which the requested scrambled controls will be carried out.

3. The authors seem a little confused about where STIM1 is located in cells based on writing in both the introduction and in the description of figure 5. Although there is a small percentage of STIM1 in the plasma membrane, store depletion does not cause it to translocate from the ER to the PM.

Rather, it moves within the ER to areas of close ER-PM apposition. There are numerous primary articles and reviews establishing this point.

We are not confused but we accept that the text could be clearer. Our understanding coincides with that of the referee and we will make significant textual changes to clarify.

To that point, it is notable that STIM1 relocalization in response to cold is to areas where Orai1 is also moving to. In truth, these sites are ultimately controlled by STIM1, not Orai1, since STIM1 localization is space limited to these ER-PM junctions while Orai1 is free to diffuse through the PM. TIRFM can be done at many levels; these images suggest to me that this is a relatively wide TIRF image. They are also highly suggestive that STIM1 is cold sensitive.

As in the previous point, we accept that the text could be clearer. Our understanding coincides with that of the referee and we will make significant textual changes to clarify.

4. Given the central claim of this paper, a clear and direct demonstration that cold does not cause emptying of ER Ca2+ stores is needed by comparing ER Ca2+ release using thapsigargin or caffeine.

We will carry out the experiments suggested by the referee and will present the results in a revised MS.

Minor Comment:

1. Bottom paragraph of page 10, fig 3 is referenced when fig 4 is intended.

We will correct.

Referee #2:

In this manuscript, Buijs and coworkers study the phenomenon of cold-induced calcium (Ca2+) increases in neurons, HEK293 and PC12 cells. Using pharmacological, fura-2 and electrophys approaches, the authors suggest that somatosensory and sympathetic neurons have evolved two distinct mechanisms of cold-induced cytosolic Ca2+ increases, both dependent on extracellular Ca2+: 1) via CaV channels and 2) via SOCE. The authors provide evidence that the SOCE-related mechanisms is dependent on STIM1 and Orai1, but not dependent on ER Ca2+ levels. Finally, the authors suggest that the phenomenon the describe may explain cold-induced vasodilation, but provide no experimental evidence for this.

I have several concerns that preclude publication of this manuscript in the current form:

1) Fluorescence is a temperature-dependent phenomenon. The authors should show how temperature separately affects the 340nm and 380nm Fura-2 signals (i.e. show some raw traces) to demonstrate that the ratios reported are not simply due to temperature-dependent fluorescence changes.

Agreed – we will supply the requested traces. Note also that in answer to Referee 1 we will provide stronger evidence that cold does not cause release of calcium from internal stores.

2) It is not clear why cold ramps were performed from 31 deg C to 4 deg C; what happens if the cold ramp starts at a more physiological temperature (i.e. 37 deg)?

The starting temperature of 31° was chosen as it closely reflects the skin temperature. We will carry out the requested experiments at a higher temperature and will supply the data in a revised MS.

3) With cells bathed in HEPES buffer, how can the authors exclude that the apparent temperature-dependent affects are not due to change in bathing medium pH given the deltapKa/deltaT of HEPES?

We will supply the requested measurements of pH as a function of temperature. We will also repeat critical experiments using buffer that has lower ΔKa/ΔT

4) Page 10 - I don't think the appropriate figure is being referred to (should this be Fig 4d-g)? Similarly, further down that paragraph - should this be Figure 4d?

We will check all Fig references in the MS.

5) Figure 5b - the puncta formation due to cold is not convincing. It appears that those puncta are pre-existing at baseline.

Diffuse ORAI1 fluorescence is visible before application of cold in Fig. 5a (left image), as expected, but as shown quantitatively in Fig 5b is mostly below threshold. The effect of cold is to aggregate the ORAI1 fluorescence into distinct bright puncta (Fig. 5a, middle image and Fig. 5b, c). STIM1 fluorescence is also visible, because the TIRF image was intentionally deep and includes STIM1 in the ER close to the surface membrane. We will supply further explanation in the text to reassure the referee.

Further, the # of puncta per cell (panel b) does not seem to be consistent with the representative images shown in panel A.

Fig 5b-d are averages taken from 44 cells in 8 different wells, and cells were selected randomly, not based on whether there are no puncta present at baseline.

As a control, the authors should show how puncta form in response to ER Ca2+ depletion.

We will carry out experiments to address puncta formation after store depletion by examining the effects of ER depletion by thapsigargin on STIM1 translocation and ORAI1 puncta formation.

6) How can the authors reconcile that decreased number of STIM puncta at colder temperature with increased SOCE?

In answer to this point, the most obvious effect of cold, as shown in Fig. 5, is:

1) ***to cause translocation of STIM1 towards the cell membrane;***
2) ***to increase the number of ORAI1 puncta by causing aggregation of existing ORAI1 channels in the surface membrane;***
3) ***To increase co-localization of STIM1 and ORAI1***

As the referee points out, there is also a much smaller (10-fold less) decrease in the number of STIM1 puncta (Fig. S8). This small change is probably due to movement of some STIM1 out of the plane of focus. However this is clearly a minor effect compared to the large increase in STIM1-

Orai1 co-localization (Fig. 5). We will make textual changes and will combine Fig S8 into Fig. 5 to make the results clear for both STIM1 and ORAI1.

Do the authors believe that the activation of Orai1 channels due to cold occurs independent of STIM1? If so, what would be the mechanism?

Our data is consistent with STIM1 translocation to regions of the ER adjacent to the surface membrane, where STIM1 causes ORAI1 puncta formation and consequent activation of ORAI1 channels, and we did not intend to imply the scenario suggested by the referee. We will spell this out more clearly.

7) Previous work by Patapoutian group showed that increased temperatures (from 37 to above 40 deg C) induce STIM1 activation, and returning to lower temperatures causes profound activation of Orai1 channels, independent of ER Ca2+ levels. More specific discussion of this previous work and how it may related to the present observations is needed.

We will expand our discussion of the Patapoutian work (Xiao et al, 2011) and a later paper from one of the original authors (Liu et al, 2019) as outlined in the above reply to Referee 1. However, these authors used temperatures elevated above the normal range and did not look at the effect of cold temperatures. Their work was also carried out in non-neuronal cells. Our work breaks new ground by showing a STIM1-ORAI1 mechanism is important in the responses of neurons to extreme cold temperatures.

8) Previous work showed that nitric oxide suppresses SOCE and CRAC currents; how do the authors reconcile those previous observations with their supposition that the cold-induced SOCE drives CVID via nNOS activity?

Our suggestion is that a calcium increase caused by the opening of ORAI1 channels may activate NOS and thus release the vasodilator NO from neuronal terminals, inducing vasodilation. Physiological levels of NO that are required to cause vasodilation in vivo are low. The work referred to (we presume the paper by Gui et al, J Mol Biol 2018) showing that NO inhibits SOCE used a high concentration in vitro of the NO donor GSNO (250μM). While the referee raises an interesting point, we do not feel that this work demonstrates conclusively that our proposal is invalid.

The authors should consider adding a model diagram with respect CVID signaling in the Discussion, which ties together all the observations from the present study and previous studies. Alternatively, a model diagram that ties together all previous temperature-induced cytosolic Ca2+ stimulation studies with the present observations would be very useful.

We agree that a diagram would increase the accessibility of our work and will provide a suitable one.

9) In general, the referencing in the manuscript appears to be dubious. There are several instances where the authors are referencing STIM1 and/Orai1 observations prior to 2005/2006, when in fact these proteins were not identified until 2005/2006. The authors need to carefully check all references and ensure they are referencing the primary publication.

We have looked through all references and can only find one instance of a reference to I_{CRAC} dating to before the discovery of ORAI channels. We will check and amend all references carefully.

2nd Editorial Decision 27th May 2022

Dear Prof. McNaughton,

Firstly, I would like to thank you for trusting the appeals process at EMBO Journal. I have read your point-by-point response to the authors carefully, and have also taken a fresh look at the referees' reports and the manuscript files.

The initial editorial decision was based on my opinion that you had failed fully to convince the referees that ORAI1 and STIM1 combine to signal noxious cold in neurons. This was possibly due to both a lack of mechanistic detail (as implied by referee #2 - point 6), and a bold hypothesis which set a high bar for your experiments to clear.

Having said this, I fully appreciate that a number of the referees' concerns can be addressed. I am also very impressed by the elegance of the sensory circuit you propose. I would like, therefore, to reverse my decision and invite you to address the referees' comments in a revised version of the manuscript. At this early stage, it is important that I stress that i) your revised manuscript must receive strong support from both referees if it is to be published in EMBO Journal, and ii) it is difficult for me to predict (based on your revision plan) whether this support will be forthcoming. I therefore urge you to add as much mechanistic detail to the manuscript as you can. Should you wish to discuss how best to proceed, I would be happy to meet with you on Zoom one afternoon next week (or the week after). Let me know and I will send a link.

I should add that it is EMBO Journal policy to allow only a single round of revision, and acceptance of your manuscript will therefore depend on the completeness of your responses in this revised version.

When preparing your letter of response to the referees' comments, please bear in mind that this will form part of the Review Process File, and will therefore be available online to the community. For more details on our Transparent Editorial Process, please visit our website: https://www.embopress.org/page/journal/14602075/authorguide#transparentprocess

We generally allow three months as standard revision time, although this is more of a guideline than a deadline. As a matter of policy, competing manuscripts published during this period will not negatively impact on our assessment of the conceptual advance presented by your study. However, we request that you contact the editor as soon as possible upon publication of any related work, to discuss how to proceed. Should you foresee a problem in meeting this three-month deadline, please let us know in advance and we may be able to grant an extension.

Thank you for the opportunity to consider your work for publication. I look forward to your revision.

Yours sincerely,

William

William Teale, PhD
Editor
The EMBO Journal
w.teale@embojournal.org

Instructions for preparing your revised manuscript:

Please make sure you upload a letter of response to the referees' comments together with the revised manuscript.

Please also check that the title and abstract of the manuscript are brief, yet explicit, even to non-specialists.

When assembling figures, please refer to our figure preparation guideline in order to ensure proper formatting and readability in print as well as on screen:
https://bit.ly/EMBOPressFigurePreparationGuideline
See also guidelines for figure legends: https://www.embopress.org/page/journal/14602075/authorguide#figureformat

IMPORTANT: When you send the revision we will require
- a point-by-point response to the referees' comments, with a detailed description of the changes made (as a word file).
- a word file of the manuscript text.
- individual production quality figure files (one file per figure)
- a complete author checklist, which you can download from our author guidelines (https://www.embopress.org/page/journal/14602075/authorguide).
- Expanded View files (replacing Supplementary Information)
Please see out instructions to authors
https://www.embopress.org/page/journal/14602075/authorguide#expandedview

Please remember: Digital image enhancement is acceptable practice, as long as it accurately represents the original data and conforms to community standards. If a figure has been subjected to significant electronic manipulation, this must be noted in the figure legend or in the 'Materials and Methods' section. The editors reserve the right to request original versions of figures and the original images that were used to assemble the figure.

Further information is available in our Guide For Authors: https://www.embopress.org/page/journal/14602075/authorguide

We realize that it is difficult to revise to a specific deadline. In the interest of protecting the conceptual advance provided by the work, we recommend a revision within 3 months (25th Aug 2022). Please discuss the revision progress ahead of this time with the editor if you require more time to complete the revisions. Use the link below to submit your revision:

https://emboj.msubmit.net/cgi-bin/main.plex

When submitting your revised manuscript, please carefully review the instructions below and include the following items:

1) a .docx formatted version of the manuscript text (including legends for main figures, EV figures and tables). Please make sure that the changes are highlighted to be clearly visible.

2) individual production quality figure files as .eps, .tif, .jpg (one file per figure).

3) a .docx formatted letter INCLUDING the reviewers' reports and your detailed point-by-point response to their comments. As part of the EMBO Press transparent editorial process, the point-by-point response is part of the Review Process File (RPF), which will be published alongside your paper.

4) a complete author checklist, which you can download from our author guidelines (https://wol-prod-cdn.literatumonline.com/pb-assets/embo-site/Author Checklist%20-%20EMBO%20J-1561436015657.xlsx). Please insert information in the checklist that is also reflected in the manuscript. The completed author checklist will also be part of the RPF.

5) Please note that all corresponding authors are required to supply an ORCID ID for their name upon submission of a revised manuscript.

6) We require a 'Data Availability' section after the Materials and Methods. Before submitting your revision, primary datasets produced in this study need to be deposited in an appropriate public database, and the accession numbers and database listed under 'Data Availability'. Please remember to provide a reviewer password if the datasets are not yet public (see https://www.embopress.org/page/journal/14602075/authorguide#datadeposition). If no data deposition in external databases is needed for this paper, please then state in this section: This study includes no data deposited in external repositories. Note that the Data Availability Section is restricted to new primary data that are part of this study.

Note - All links should resolve to a page where the data can be accessed.

7) When assembling figures, please refer to our figure preparation guideline in order to ensure proper formatting and readability in print as well as on screen:
http://bit.ly/EMBOPressFigurePreparationGuideline

Please remember: Digital image enhancement is acceptable practice, as long as it accurately represents the original data and conforms to community standards. If a figure has been subjected to significant electronic manipulation, this must be noted in the figure legend or in the 'Materials and Methods' section. The editors reserve the right to request original versions of figures and the original images that were used to assemble the figure.

8) For data quantification: please specify the name of the statistical test used to generate error bars and P values, the number (n) of independent experiments (specify technical or biological replicates) underlying each data point and the test used to calculate p-values in each figure legend. The figure legends should contain a basic description of n, P and the test applied. Graphs must include a description of the bars and the error bars (s.d., s.e.m.).

9) We would also encourage you to include the source data for figure panels that show essential data. Numerical data can be provided as individual .xls or .csv files (including a tab describing the data). For 'blots' or microscopy, uncropped images should be submitted (using a zip archive or a single pdf per main figure if multiple images need to be supplied for one panel). Additional information on source data and instruction on how to label the files are available at .

10) We replaced Supplementary Information with Expanded View (EV) Figures and Tables that are collapsible/expandable online (see examples in https://www.embopress.org/doi/10.15252/embj.201695874). A maximum of 5 EV Figures can be typeset. EV Figures should be cited as 'Figure EV1, Figure EV2" etc. in the text and their respective legends should be included in the main text after the legends of regular figures.

- For the figures that you do NOT wish to display as Expanded View figures, they should be bundled together with their legends in a single PDF file called *Appendix*, which should start with a short Table of Content. Appendix figures should be referred to in the main text as: "Appendix Figure S1, Appendix Figure S2" etc. See detailed instructions regarding expanded view here: .

- Additional Tables/Datasets should be labelled and referred to as Table EV1, Dataset EV1, etc. Legends have to be provided in a separate tab in case of .xls files. Alternatively, the legend can be supplied as a separate text file (README) and zipped together with the Table/Dataset file.

11) Our journal encourages inclusion of *data citations in the reference list* to directly cite datasets that were re-used and obtained from public databases. Data citations in the article text are distinct from normal bibliographical citations and should directly link to the database records from which the data can be accessed. In the main text, data citations are formatted as follows: "Data ref: Smith et al, 2001" or "Data ref: NCBI Sequence Read Archive PRJNA342805, 2017". In the Reference list, data citations must be labeled with "[DATASET]". A data reference must provide the database name, accession number/identifiers and a resolvable link to the landing page from which the data can be accessed at the end of the reference. Further instructions are available at .

Reply to: EMBOJ-2022-111348 Decision Letter

"STIM1 and ORAI1 form a novel cold transduction mechanism in peripheral sensory and sympathetic neurons"

Referee #1:

The manuscript entitled, "STIM1 and ORAI1 form a novel cold transduction mechanism in peripheral sensory and sympathetic neurons" provides evidence that store-operated Ca2+ entry may be a cold sensor. The strongest evidence by far are the observations that STIM1 RNAi eliminates cold sensitivity in SCG neurons and the fact that STIM and Orai colocalize in HEK293 cells overexpressing the proteins in response to cold. However, I also have some major concerns that need to be reconciled. Perhaps the most serious issue is that STIM1 and Orai1 are expressed in every cell except for erythrocytes. As such, it is unclear why novel cold sensitivity observed in a neuronal subset could be accounted for by STIM1 and Orai1 expression. I recognized that this issue was brought up in the discussion, but it should be raised in the introduction and directly addressed within the study.

For the reasons outlined in the existing Discussion, it is unlikely that there is any difference, at the genetic level, between the cold-sensitive STIM expressed in some DRG and SCG neurons and the cold-insensitive STIM1 expressed in others, which leaves post-translational modification (e.g. phosphorylation, RNA editing) as the most likely explanation. We now discuss these possibilities in the revised Discussion. We feel that this particular issue is best kept in the Discussion rather than the Introduction as it deals with a matter arising directly from the results in the paper.

Similarly, the fact that STIM1 activation was previously shown to occur in response to heating and cooling cells should be discussed in the introduction and not at the end, as it is directly relevant to whether or not STIM/Orai are temperature sensitive.

We have retained an abbreviated discussion of the work of the Xiao and Stucky groups in the Discussion, but we now also discuss these papers in the Introduction together with a brief survey of the exciting discoveries about the function of STIM and ORAI that have been made in the last 15 years.

The extent to which the current findings reflect a new function of STIM1 should also be addressed.

We have now inserted a sentence in the Discussion dealing with this: "These observations show that STIM1, in addition to its function as a sensor of calcium levels in intracellular calcium stores, is also able to sense cold and trigger activation of ORAI1 channels without a change in intraluminal calcium levels."

Major Comments:

1. Although YM58483 does block Orai1, it blocks TRPC channels at similar concentrations (He et al, 2005) and activates TRPM4 at much lower concentrations (Takezawa et al, 2006). As such, these data are insufficient to demonstrate that Orai1 is cold-activated. Although I recognize and acknowledge that MRS1845 was also used. However, I have not seen any recent papers using this pharmacological agent; irrespective I am not aware of ANY truly specific inhibitors of Orai1.

We agree that pharmacological evidence is always open to the criticism that effects could be off-target and for this reason we used two blockers of different structure that have both been shown in previous studies to inhibit Orai1. The referee states that MRS1845 has not been used recently, perhaps suggesting that it may have been found not to be an effective ORAI blocker, but the following recent paper used this blocker successfully (Zhu et al., 2021). ***However, as is made clear in the MS, we take the pharmacological evidence only as a guide to studies in which: (1) expression of STIM1 and ORAI1 are shown to recapitulate the cold-activated calcium influx (Fig.4); (2) downregulation of STIM1 in neurons is shown to suppress cold-activated calcium influx (Fig. 4); (3) measures using TIRF microscopy of translocation of STIM1 and formation of ORAI1 puncta support their involvement in cold-induced calcium influx (Fig. 5). These experiments provide much stronger evidence for the involvement of STIM1 and ORAI1. We have made the following textual changes on pg 11 to reinforce this point:***

"The above experiments suggest that a member of the ORAI ion channel family may be responsible for the novel cold-activated calcium entry but, as with all pharmacological studies, the results are open to the criticism that off-target block of an unrelated ion channel or channels may be responsible for the observed suppression of the cold-activated calcium influx by ORAI blockers. We therefore expressed the three isoforms of ORAI and two isoforms of STIM (Hou et al., 2012) ***in HEK293 cells with a view to determining (i) whether expression of an ORAI channel isoform in combination with a member of the STIM family can recapitulate the cold-activated calcium influx; and (ii) which STIM and ORAI isoforms are involved in generating the novel cold-activated calcium entry (see Fig. 4A-C)."***

2. The STIM1 siRNA experiments in fig 4 are crucial components of this study. Although somewhat convincing, the use of a scrambled control is necessary to show that this is not a transfection artifact.

We have performed further experiments in which the requested scrambled control was carried out (new Supplementary Fig. S11). There was no effect of the scrambled siRNA on cold-evoked calcium entry. Now added to text on pg 12:

"A control non-targeting siRNA (see Methods) did not significantly reduce the amplitude of the calcium increase in response to cold in successfully transfected neurons when compared with neurons on the same cover slips that were not transfected (Supplementary Fig. 11). "

3. The authors seem a little confused about where STIM1 is located in cells based on writing in both the introduction and in the description of figure 5. Although there is a small percentage of STIM1 in the plasma membrane, store depletion does not cause it to translocate from the ER to the PM. Rather, it moves within the ER to areas of close ER-PM apposition. There are numerous primary articles and reviews establishing this point.

We are not confused but we accept that our previous text could be clearer. Our understanding coincides with that of the referee and we have made significant textual changes to clarify. Changes to the Introduction have been outlined above, and the text under "STIM1 and ORAI1 form puncta in response to cold" on pg 13 has been modified:

"In order to evoke a surface membrane calcium influx in response to emptying of intracellular stores (a store-operated calcium influx, SOCE), STIM1 proteins located in the endoplasmic reticulum aggregate and migrate within the ER membrane to areas of close ER-plasma membrane apposition, where they interact with and open ORAI1 ion channels (reviewed in Qiu & Lewis, 2019; Prakriya & Lewis, 2015). "

To that point, it is notable that STIM1 relocalization in response to cold is to areas where Orai1 is also moving to. In truth, these sites are ultimately controlled by STIM1, not Orai1, since STIM1 localization is space limited to these ER-PM junctions while Orai1 is free to diffuse through the PM. TIRFM can be done at many levels; these images suggest to me that this is a relatively wide TIRF image. They are also highly suggestive that STIM1 is cold sensitive.

As in the previous point, we accept that the text could be clearer. Our understanding coincides with that of the referee and we have made significant textual changes to clarify. The existence of some pre-existing STIM1 puncta is interesting and may imply some activation of the STIM1-ORAI1 system before application of the cold stimulus. We found that the application of a cold stimulus causes HEK293 cells to change shape and that this could be minimised by removal of calcium (nominal zero Ca) from the extracellular solution, which caused partial formation of STIM1 puncta at baseline.

The TIRF microscope was set up with the laser beam close to the critical angle so as to image a deep section (c. 100nm) of membrane and juxta-membrane features such as STIM1 within ER membrane. We now provide further details in Methods together with changes in the main text.

4. Given the central claim of this paper, a clear and direct demonstration that cold does not cause emptying of ER Ca2+ stores is needed by comparing ER Ca2+ release using thapsigargin or caffeine.

The experiment suggested by the referee has been carried out and is shown in Supplementary Fig 13. During application of a cold stimulus in the absence of external calcium there is no detectable release of intracellular calcium, and on restoration of external calcium the intracellular calcium level returns to its previous level with little or no overshoot. When thapsigargin is applied in the absence of external calcium there is also no detectable discharge of store calcium, which is a little surprising but may be due to the known sparsity of the ER in neurons. However, on restoration of normal extracellular calcium levels there is a significantly greater overshoot due to SOCE, as expected when stores have been discharged by thapsigargin. These experiments confirm that there is no detectable store discharge caused by the application of cold.

Minor Comment:

1. Bottom paragraph of page 10, fig 3 is referenced when fig 4 is intended.

Now corrected.

Referee #2:

In this manuscript, Buijs and coworkers study the phenomenon of cold-induced calcium (Ca2+) increases in neurons, HEK293 and PC12 cells. Using pharmacological, fura-2 and electrophys approaches, the authors suggest that somatosensory and sympathetic neurons have evolved two distinct mechanisms of cold-induced cytosolic Ca2+ increases, both dependent on extracellular Ca2+: 1) via CaV channels and 2) via SOCE. The authors provide evidence that the SOCE-related mechanisms is dependent on STIM1 and Orai1, but not dependent on ER Ca2+ levels. Finally, the authors suggest that the phenomenon the describe may explain cold-induced vasodilation, but provide no experimental evidence for this.

I have several concerns that preclude publication of this manuscript in the current form:

1) Fluorescence is a temperature-dependent phenomenon. The authors should show how temperature separately affects the 340nm and 380nm Fura-2 signals (i.e. show some raw traces) to demonstrate that the ratios reported are not simply due to temperature-dependent fluorescence changes.

We now show the requested traces in Supplementary Fig S3. Although there are significant changes to each individual fluorescence trace, the ratio trace is stable when a cold ramp is imposed in the absence of external calcium, in contrast to significant changes when external calcium is present. We show that any minor temperature-dependent changes in the ration trace are much smaller than any of the calcium influx mechanisms elucidated in the paper.

2) It is not clear why cold ramps were performed from 31 deg C to 4 deg C; what happens if the cold ramp starts at a more physiological temperature (i.e. 37 deg)?

The starting temperature of 31-32° was chosen as it closely reflects the skin temperature and is therefore more "physiological" than 37°. We have now carried out experiments at a starting temperature of 37° and the results are shown in Supplementary Fig. S2. There is no significant difference from the results obtained at a starting temperature of 32°.

3) With cells bathed in HEPES buffer, how can the authors exclude that the apparent temperature-dependent affects are not due to change in bathing medium pH given the deltapKa/deltaT of HEPES?

We have carried out measurements of pH as a function of temperature in buffer as used in the paper. A larger change in pH has no significant effect on the responses of SCG neurons to cold (Supplementary Fig. S4)

4) Page 10 - I don't think the appropriate figure is being referred to (should this be Fig 4d-g)? Similarly, further down that paragraph - should this be Figure 4d?

Thank you for pointing this out. All Fig references have been carefully checked.

5) Figure 5b - the puncta formation due to cold is not convincing. It appears that those puncta are pre-existing at baseline.

We agree that some puncta (particularly STIM1) are pre-existing and we apologise for not making clear how the experiment was done and therefore the likely reason for this. In this experiment we had difficulties with cell movement apparently caused by the application of a cold temperature. The experiment was therefore carried out in nominal zero calcium that was found to immobilise the cells. The low external Ca caused some aggregation of STIM1 and to a lesser extent ORAI1 into puncta. We have added a further description to the appropriate section in Results and also in Methods.

For ORAI1, some weakly fluorescent puncta are observed at 35℃ (see Fig. 5A, B, C). The effect of cold is to cause a significant aggregation of diffuse ORAI1 fluorescence into distinct bright puncta and an approximately 3-fold increase in the number of puncta (see Fig. 5A, middle image and new Fig. 5D,E which includes both STIM1 and ORAI1 data appropriately colour-coded, see below). Thus while there are some ORAI1 puncta visible at baseline, it is not correct to say that that the ORAI puncta are all pre-existing.

For STIM1,the referee's statement is correct - fluorescent puncta are visible at 35℃ and the significant change in STIM1 on cooling is not an increase in number of puncta (Fig. 5D,E) but rather an increased colocalization of STIM1 with ORAI1 (Fig. 5F).

The section in Results dealing with this experiment has been rewritten to make the above clear

Further, the # of puncta per cell (panel b) does not seem to be consistent with the representative images shown in panel A.

Fig. 5A is a single example cell, while Fig 5D,E are averages taken from 44 cells selected randomly from 8 different wells. The area of each cell attached to the bottom of the well and therefore visible in the TIRF image was variable between cells, which explains why the visible puncta in Fig 5A may not appear representative. We now show the data of Fig. 5D and E as individual cells in B and C to show the degree of variability among cells, which we hope clarifies the issue. The changes shown in D and E for ORAI1 are highly significant.

As a control, the authors should show how puncta form in response to ER Ca^{2+} depletion.

This is a reasonable request but we have been unfortunately been unable to perform this experiment in a reasonable time frame because of unavailability of the apparatus. The experiment in Fig. S13 provides a partial answer in that it shows that following ER depletion by thapsigargin SOCE is activated, a process that has in many different cell types been shown to be associated with the formation of ORAI1 puncta.

6) How can the authors reconcile that decreased number of STIM puncta at colder temperature with increased SOCE?

The small apparent decrease in STIM1 puncta in Fig. 5 is non-significant – see revised Fig 5D, E. The probable reason for the failure of STIM1 puncta to increase is explained above and in the revised text – the experiment was done in nominal zero calcium that had caused prior aggregation of STIM1.

The effect of low temperature is to cause translocation of STIM1 to the membrane – seen most clearly in the edge-on view indicated by the arrow in Fig. 5A. This increases the number of ORAI1 puncta by causing aggregation of existing ORAI1 channels in the surface membrane (Fig. 5A - E), and increases co-localization of STIM1 and ORAI1 (Fig. 5F).

We have made textual changes and have combined former Fig S8 into Fig. 5 to make the results clear for both STIM1 and ORAI1.

Do the authors believe that the activation of Orai1 channels due to cold occurs independent of STIM1? If so, what would be the mechanism?

No, our data is consistent with translocation of pre-existing STIM1 puncta (whose formation has been induced by the low calcium in which the imaging was done, see above) to regions of the ER adjacent to the surface membrane, where STIM1 causes ORAI1 puncta formation and consequent activation of ORAI1 channels. We did not intend to imply the scenario suggested by the referee and we have now rewritten the text to make this clear

7) Previous work by Patapoutian group showed that increased temperatures (from 37 to above 40 deg C) induce STIM1 activation, and returning to lower temperatures causes profound activation of Orai1 channels, independent of ER Ca2+ levels. More specific discussion of this previous work and how it may related to the present observations is needed.

See reply to Ref 1 above. We have inserted a discussion of the Patapoutian group work (Xiao et al, 2011) and a later paper from one of the original authors (Liu et al, 2019) into the Introduction. However, we note that these authors used elevated temperatures above the normal range and did not look at the effect of cold temperatures. Their work was also carried out in non-neuronal cells. Our work breaks new ground by showing a STIM1-ORAI1 mechanism is important in the responses of neurons to extreme cold temperatures.

8) Previous work showed that nitric oxide suppresses SOCE and CRAC currents; how do the authors reconcile those previous observations with their supposition that the cold-induced SOCE drives CVID via nNOS activity?

Our paper shows that the opening of ORAI1 channels in SCG and DRG neurons causes an intracellular calcium increase. In the Discussion we suggest that this increase may activate NOS and thus release the vasodilator NO from neuronal terminals, providing a molecular explanation for the phenomenon of cold-induced vasodilation (CIVD). Physiological levels of NO that are required to cause vasodilation in vivo are low. The work referred to (we presume the paper by Gui et al, J Mol Biol 2018), showing that NO inhibits SOCE, used a high concentration in vitro of the NO donor GSNO (250μM). While the referee raises an interesting point, we do not feel that this work demonstrates conclusively that our proposal is invalid.

The authors should consider adding a model diagram with respect CVID signaling in the Discussion, which ties together all the observations from the present study and previous studies. Alternatively, a model diagram that ties together all previous temperature-induced cytosolic Ca2+ stimulation studies with the present observations would be very useful.

We agree that a diagram would increase the accessibility of our work and we now provide a suitable one (Fig. 7) that is referred to in the Discussion.

9) In general, the referencing in the manuscript appears to be dubious. There are several instances where the authors are referencing STIM1 and/Orai1 observations prior to 2005/2006, when in fact these proteins were not identified until 2005/2006. The authors need to carefully check all references and ensure they are referencing the primary publication.

We have looked through all references and can only find one instance of a reference to I_{CRAC} (not STIM/ORAI) dating to before the discovery of ORAI channels. We have checked and amended all

references carefully. In most places we refer to recent authoritative reviews of this field rather than to original papers.

References

Hou, X., Pedi, L., Diver, M., & Long, S. B. (2012). Crystal Structure of the Calcium Release – Activated Calcium Channel Orai. *Science*, *November*, 1–10.

Zhu, X., Ma, K., Zhou, K., Liu, J., Nürnberg, B., & Lang, F. (2021). *Vasopressin-stimulated ORAI1 expression and store-operated Ca 2+ entry in aortic smooth muscle cells*. https://doi.org/10.1007/s00109-020-02016-4/Published

Dear Prof. McNaughton,

We have now received re-review reports on your submitted manuscript from both referees, which are attached below. As you will see, you have addressed most of their comments satisfactorily. However, some concerns remain. The majority of these can be addressed without further experimentation, but I would like you to consider whether the manuscript is weakened by the absence of controls for Figure 5 which were requested by Reviewer 2.

In addition, there are some remaining editorial points which need to be addressed. Would you therefore please:

include a "Data availability" section,
include a "Disclosure and competing interests statement" and remove your declaration from the Acknowledgements section,
complete the contributor role in the CrediT section of our website. CrediT replaces our author contributions section and allows you to provide more detailed descriptions than previously.
Include the data referred to on page 11 ("data not shown") in an Appendix Figure and re-label the Appendix Figures accordingly.
Please also add scale bars to Figure 1A and Figure 1E.

Regarding the figure callouts, Figure 1A and 1B should be called out before Figure 1C, 1D before 1G; Figure 3H should be called out after Figure 3G, and callouts for Figure 1E, 1F and 6A are missing.

We encourage the publication of source data with the aim of making primary data more accessible and transparent to the reader. It would be great if you could provide me with a PDF or Excel file for each figure that contains the original, unprocessed key pieces of data used in the figures. The files should be labeled with the appropriate figure/panel number; further annotation could be useful but is not essential. These files will be published online with the article as supplementary "Source Data" files. We anticipate that their inclusion will make your work more discoverable and useable to scientists in the future.
We include a synopsis of the paper (see http://emboj.embopress.org/). Please provide me with a general summary statement and 3-5 bullet points that capture the key findings of the paper.

We also need a summary figure for the synopsis. The size should be 550 wide by [200-400] high (pixels). You can also use something from the figures if that is easier.
EMBO Press is an editorially independent publishing platform for the development of EMBO scientific publications.

Best wishes,

William

William Teale, PhD
Editor
The EMBO Journal
w.teale@embojournal.org

Instructions for preparing your revised manuscript:

Please check that the title and abstract of the manuscript are brief, yet explicit, even to non-specialists.

When assembling figures, please refer to our figure preparation guideline in order to ensure proper formatting and readability in print as well as on screen:
https://bit.ly/EMBOPressFigurePreparationGuideline
See also figure legend guidelines: https://www.embopress.org/page/journal/14602075/authorguide#figureformat

IMPORTANT: When you send the revision we will require
- a point-by-point response to the referees' comments, with a detailed description of the changes made (as a word file).
- a word file of the manuscript text.
- individual production quality figure files (one file per figure)
- a complete author checklist, which you can download from our author guidelines (https://www.embopress.org/page/journal/14602075/authorguide).
- Expanded View files (replacing Supplementary Information)
Please see out instructions to authors
https://www.embopress.org/page/journal/14602075/authorguide#expandedview

Please remember: Digital image enhancement is acceptable practice, as long as it accurately represents the original data and conforms to community standards. If a figure has been subjected to significant electronic manipulation, this must be noted in the figure legend or in the 'Materials and Methods' section. The editors reserve the right to request original versions of figures and the original images that were used to assemble the figure.

Further information is available in our Guide For Authors: https://www.embopress.org/page/journal/14602075/authorguide

We realize that it is difficult to revise to a specific deadline. In the interest of protecting the conceptual advance provided by the work, we recommend a revision within 3 months (15th Dec 2022). Please discuss the revision progress ahead of this time with the editor if you require more time to complete the revisions. Use the link below to submit your revision:

https://emboj.msubmit.net/cgi-bin/main.plex

Referee #1:

I am essentially satisfied by the author's responses, except for a couple of minor concerns:

1. The idea that STIM1 could translocate from pre-formed puncta to a different pre-formed puncta is difficult to reconcile with my prior understanding of what puncta are. "Puncta" in this context generally refers to ER-PM junctions which STIM1 translocates to. I'm not sure what the aggregates of STIM1 are in DRGs at normal temperatures (Fig 5), but I don't think that they are puncta.

2. The discussion includes some speculations about cell-specific post-translational modifications of STIM1 that could account for DRG-specific cold sensitivity. However, the observation that this could be replicated in HEK293 and PC12 cells is not consistent with that explanation. If one accepts these results as correct, this would have to be an intrinsic property of STIM1.

Referee #2:

Overall, the authors have done a good job addressing most of my concerns. Nevertheless, there remains some inconsistencies in the puncta data that are confusing and should be addressed prior to publication, as I have detailed below.

Reviewer 2 - major comment/response 5 - The new # of puncta counted for individual cells (Fig. 5D) remains inconsistent with the representative images shown in 5A. I can count ~4-5 Orai1 puncta induced after cold exposure, but the numbers in panel 5D suggest ~32-42. Again, a control experiment is needed to show how puncta form (counts/sizes/colocalization with STIM) in response to TG or caffeine treatment in their system. Could the authors consider a collaboration to complete this experiment or perhaps an alternative to TIRF (i.e. confocal?).

Reviewer 2 - major comment/response 6 - If STIM1 are in pre-existing puncta due to the nominally Ca^{2+}-free buffer used, why are they not coupled with Orai1? If the nominally-free Ca^{2+} does not cause the depletion of ER Ca^{2+} stores, what is causing the pre-existing STIM1 puncta, specifically when using the Ca^{2+}-free buffer?

Reviewer 1 - major comment/response 4 - The inability to detect ER Ca^{2+} discharge after TG treatment would suggest to me that the stores are already depleted to some extent. The larger "overshoot" after TG treatment + external Ca^{2+} re-addition could be because TG binding to SERCA is irreversible and the magnitude/extent/persistence of SOCE activation is enhanced with TG. In my opinion, the response to this important comment (related to reviewer 2 comment/response 6) needs more careful thought.

Minor:
Reviewer 1 - major comment/response 1 - These are not isoforms that are expressed - these are homologues/paralogues. This needs to be clarified.

Replies to points raised by Editor

The majority of (the points raised by the referees) can be addressed without further experimentation, but I would like you to consider whether the manuscript is weakened by the absence of controls for Figure 5 which were requested by Reviewer 2.

See reply to Referee 2 below.

In addition, there are some remaining editorial points which need to be addressed. Would you therefore please:

include a "Data availability" section, **- Done**
include a "Disclosure and competing interests statement" and remove your declaration from the Acknowledgements section, **-Done**
complete the contributor role in the CrediT section of our website. CrediT replaces our author contributions section and allows you to provide more detailed descriptions than previously. **- Not able to access website. Editorial staff have assured us that author contributions have already been entered into the EMBO J. website.**
Include the data referred to on page 11 ("data not shown") in an Appendix Figure and re-label the Appendix Figures accordingly. **-Done**
Please also add scale bars to Figure 1A and Figure 1E. **-Done**

Regarding the figure callouts, Figure 1A and 1B should be called out before Figure 1C, 1D before 1G; Figure 3H should be called out after Figure 3G, and callouts for Figure 1E, 1F and 6A are missing.
– All corrected

We encourage the publication of source data with the aim of making primary data more accessible and transparent to the reader. It would be great if you could provide me with a PDF or Excel file for each figure that contains the original, unprocessed key pieces of data used in the figures. The files should be labeled with the appropriate figure/panel number; further annotation could be useful but is not essential. These files will be published online with the article as supplementary "Source Data" files. We anticipate that their inclusion will make your work more discoverable and useable to scientists in the future. **- Done and .zip files attached**

We include a synopsis of the paper (see http://emboj.embopress.org/). Please provide me with a general summary statement and 3-5 bullet points that capture the key findings of the paper. **- Done**

We also need a summary figure for the synopsis. The size should be 550 wide by [200-400] high (pixels). You can also use something from the figures if that is easier. **- Done**

Replies to points raised by Referees

Referee #1:

I am essentially satisfied by the author's responses, except for a couple of minor concerns:

1. The idea that STIM1 could translocate from pre-formed puncta to a different pre-formed puncta is difficult to reconcile with my prior understanding of what puncta are. "Puncta" in this context generally refers to ER-PM junctions which STIM1 translocates to. I'm not sure what the aggregates of STIM1 are in DRGs at normal temperatures (Fig 5), but I don't think that they are puncta.

In the context of our experiments, by "STIM1 puncta" we mean visible aggregations of STIM1, as we do not show that they are associated with ER-PM junctions – this idea comes work from other labs in the context of activation of STIM1 by store emptying. Thus we do not propose, as suggested by the referee in the first sentence, that "STIM1 could translocate from pre-formed puncta to a different pre-formed puncta".

However, STIM1 puncta are in fact to some extent mobile, and we now include videos to illustrate this (Extended View Video Fig. 5). This explains why the STIM1 puncta observed before cooling are often in a different location from the STIM1 puncta observed several minutes later after cooling. Interestingly, there is some evidence that STIM1 forms into aggregations even in normal extracellular calcium (Left-hand video).

2. The discussion includes some speculations about cell-specific post-translational modifications of STIM1 that could account for DRG-specific cold sensitivity. However, the observation that this could be replicated in HEK293 and PC12 cells is not consistent with that explanation. If one accepts these results as correct, this would have to be an intrinsic property of STIM1.

We agree with the comments of the referee and we have shortened the discussion of how the cold-sensitivity of STIM1 might have arisen to the observation that it must be regulated in some way because sensitivity mediated by STIM1/ORAI1 mechanism is observed in only a fraction of somatosensory or sympathetic neurons, while expression of STIM1 and ORAI is seen in all neurons.

Referee #2:

Overall, the authors have done a good job addressing most of my concerns. Nevertheless, there remains some inconsistencies in the puncta data that are confusing and should be addressed prior to publication, as I have detailed below.

Reviewer 2 - major comment/response 5 - The new # of puncta counted for individual cells (Fig. 5D) remains inconsistent with the representative images shown in 5A. I can count ~4-5 Orai1 puncta induced after cold exposure, but the numbers in panel 5D suggest ~32-42.

We agree that the image in Fig. 5A contains a smaller number of ORAI1 puncta than the average data for all cells shown in Fig. 5B (blue line), but it is not atypical (see individual data in Fig. 5B). This image was selected specifically because it contained a small number of puncta and therefore the co-localisation between STIM1 and ORAI1 was easier to see. We now make this clear in the text, and to address the point raised by the referee we shown an additional image (EV Fig. 4) in which a more typical number of puncta is seen.

Again, a control experiment is needed to show how puncta form (counts/sizes/colocalization with STIM) in response to TG or caffeine treatment in their system. Could the authors consider a collaboration to complete this experiment or perhaps an alternative to TIRF (i.e. confocal?).

As we noted in our previous reply, the TIRF microscope that we used for these experiments will be out of action for some time and there is no prospect that we could do these experiments on the same setup (see "in their system" above) within any reasonable time frame. We agree that the proposed experiment, in which the effect of thapsigargin on puncta formation could have been examined , would have been interesting. However, as store-operated calcium entry or puncta formation in response to thapsigargin did not form a central plank of our work, all of which relates to the effect of cold, we do not feel that our conclusions are seriously affected weakened by its absence. Furthermore, there are many other papers that show the effect of thapsigargin in causing STIM1-ORAI1 puncta formation in HEK293 cells.

Reviewer 2 - major comment/response 6 - If STIM1 are in pre-existing puncta due to the nominally Ca2+-free buffer used, why are they not coupled with Orai1? If the nominally-free Ca2+ does not cause the depletion of ER Ca2+ stores, what is causing the pre-existing STIM1 puncta, specifically when using the Ca2+-free buffer?

Our experiments show that: 1) low calcium (not calcium-free as there is around 10μM contaminating calcium) causes formation of STIM1 puncta but these are not co-localised with ORAI1; 2) cold then causes ORAI1 to aggregate with the pre-existing STIM1 puncta, and also causes ORAI1 channels to open, as demonstrated by the ORAI-dependent calcium influx that we observe. What is causing this cold-dependent ORAI1 aggregation and activation? We can speculate that cold has caused a change in STIM1 structure that allows it to trigger ORAI1 aggregation in a way that was not possible at normal temperatures, but there is no direct evidence to support this. We have now spelled this out more clearly in the Discussion.

Reviewer 1 - major comment/response 4 - The inability to detect ER Ca2+ discharge after TG treatment would suggest to me that the stores are already depleted to some extent. The larger "overshoot" after TG treatment + external Ca2+ re-addition could be because TG binding to SERCA is irreversible and the magnitude/extent/persistence of SOCE activation is enhanced with TG. In my opinion, the response to this important comment (related to reviewer 2 comment/response 6) needs more careful thought.

To test the referee's point that stores might have been discharged before thapsigargin was applied in Fig. EV 6A, we have repeated the experiments shown in EV6 by testing application of thapsigargin very soon (30s) after going from normal calcium to 0Ca, to minimise the possibility that, in previous experiments, stores might have slowly discharged before thapsigargin was applied (Fig. EV 6C). There was still no detectable store discharge, consistent with our proposed explanation that the lack of a visible discharge was because stores are poorly developed in neurons, by comparison with model cells such as HEK293 that are often used in experiments on store filling/discharge.

Minor:
Reviewer 1 - major comment/response 1 - These are not isoforms that are expressed - these are homologues/paralogues. This needs to be clarified.

Agree – have substituted "homologue" in place of "isoform"

Dear Prof. McNaughton,

I am pleased to inform you that your manuscript has been accepted for publication in the EMBO Journal.

Congratulations on putting together a really interesting and insightful paper!

Referee #2:

Response regarding Fig. 5:
-The additional EV figure 4 is an improved view of the puncta.

Response regarding control experiment:
-This control experiment was not suggested out of "interest", as the authors suggest, but rather to demonstrate that what the authors are considering puncta in their images/quantification are truly puncta. Nevertheless, given the current technical limitations they face with respect to finding a functional TIRF imaging system, I would say this is not required at this time.

Overall, I believe the manuscript is acceptable for publication in its current form.

Please note that it is EMBO Journal policy for the transcript of the editorial process (containing referee reports and your response letter) to be published as an online supplement to each paper. If you do NOT want this, you will need to inform the Editorial Office via email immediately. More information is available here:
https://www.embopress.org/page/journal/14602075/authorguide#transparentprocess

Your manuscript will be processed for publication in the journal by EMBO Press. Manuscripts in the PDF and electronic editions of The EMBO Journal will be copy edited, and you will be provided with page proofs prior to publication. Please note that supplementary information is not included in the proofs.

You will be contacted by Wiley Author Services to complete licensing and payment information. The required 'Page Charges Authorization Form' is available here: https://www.embopress.org/pb-assets/embo-site/tej_apc.pdf - please download and complete the form and return to embopressproduction@wiley.com

EMBO Press participates in many Publish and Read agreements that allow authors to publish Open Access with reduced/no publication charges. Check your eligibility: https://authorservices.wiley.com/author-resources/Journal-Authors/open-access/affiliation-policies-payments/index.html

Should you be planning a Press Release on your article, please get in contact with embojournal@wiley.com as early as possible, in order to coordinate publication and release dates.

If you have any questions, please do not hesitate to call or email the Editorial Office. Thank you for your contribution to The EMBO Journal.

Best wishes,

William Teale

William Teale, PhD
Editor
The EMBO Journal
w.teale@embojournal.org

** Click here to be directed to your login page: https://emboj.msubmit.net

EMBO Press Author Checklist

Corresponding Author Name: Prof. Peter Anthony McNaughton	
Journal Submitted to: The EMBO Journal	
Manuscript Number: EMBOJ-2022-111348R2	

USEFUL LINKS FOR COMPLETING THIS FORM
The EMBO Journal - Author Guidelines
EMBO Reports - Author Guidelines
Molecular Systems Biology - Author Guidelines
EMBO Molecular Medicine - Author Guidelines

Reporting Checklist for Life Science Articles (updated January 2022)
This checklist is adapted from Materials Design Analysis Reporting (MDAR) Checklist for Authors. MDAR establishes a minimum set of requirements in transparent reporting in the life sciences (see Statement of Task: 10.31222/osf.io/9sm4x). Please follow the journal's guidelines in preparing your manuscript.
Please note that a copy of this checklist will be published alongside your article.

Abridged guidelines for figures
1. Data
The data shown in figures should satisfy the following conditions:
- the data were obtained and processed according to the field's best practice and are presented to reflect the results of the experiments in an accurate and unbiased manner.
- ideally, figure panels should include only measurements that are directly comparable to each other and obtained with the same assay.
- plots include clearly labeled error bars for independent experiments and sample sizes. Unless justified, error bars should not be shown for technical replicates.
- if n<5, the individual data points from each experiment should be plotted. Any statistical test employed should be justified.
- Source Data should be included to report the data underlying figures according to the guidelines set out in the authorship guidelines on Data Presentation.

2. Captions
Each figure caption should contain the following information, for each panel where they are relevant:
- a specification of the experimental system investigated (eg cell line, species name).
- the assay(s) and method(s) used to carry out the reported observations and measurements.
- an explicit mention of the biological and chemical entity(ies) that are being measured.
- an explicit mention of the biological and chemical entity(ies) that are altered/varied/perturbed in a controlled manner.
- the exact sample size (n) for each experimental group/condition, given as a number, not a range;
- a description of the sample collection allowing the reader to understand whether the samples represent technical or biological replicates (including how many animals, litters, cultures, etc.).
- a statement of how many times the experiment shown was independently replicated in the laboratory.
- definitions of statistical methods and measures:
  - common tests, such as t-test (please specify whether paired vs. unpaired), simple χ2 tests, Wilcoxon and Mann-Whitney tests, can be unambiguously identified by name only, but more complex techniques should be described in the methods section;
  - are tests one-sided or two-sided?
  - are there adjustments for multiple comparisons?
  - exact statistical test results, e.g., P values = x but not P values < x;
  - definition of 'center values' as median or average;
  - definition of error bars as s.d. or s.e.m.

Please complete ALL of the questions below.
Select "Not Applicable" only when the requested information is not relevant for your study.

Materials

Newly Created Materials	Information included in the manuscript?	In which section is the information available? (Reagents and Tools Table, Materials and Methods, Figures, Data Availability Section)
New materials and reagents need to be available; do any restrictions apply?	Not Applicable	

Antibodies	Information included in the manuscript?	In which section is the information available? (Reagents and Tools Table, Materials and Methods, Figures, Data Availability Section)
For **antibodies** provide the following information:<br>- Commercial antibodies: RRID (if possible) or supplier name, catalogue number and or/clone number<br>- Non-commercial: RRID or citation	Yes	Methods, Fig legends

DNA and RNA sequences	Information included in the manuscript?	In which section is the information available? (Reagents and Tools Table, Materials and Methods, Figures, Data Availability Section)
**Short novel DNA or RNA including primers, probes**: provide the sequences.	Yes	Methods

Cell materials	Information included in the manuscript?	In which section is the information available? (Reagents and Tools Table, Materials and Methods, Figures, Data Availability Section)
**Cell lines:** Provide species information, strain. Provide accession number in repository **OR** supplier name, catalog number, clone number, and/**OR** RRID.	Yes	Methods
**Primary cultures:** Provide species, strain, sex of origin, genetic modification status.	Yes	Methods
Report if the cell lines were recently **authenticated** (e.g., by STR profiling) and tested for mycoplasma contamination.	Not Applicable	

Experimental animals	Information included in the manuscript?	In which section is the information available? (Reagents and Tools Table, Materials and Methods, Figures, Data Availability Section)
**Laboratory animals or Model organisms:** Provide species, strain, sex, age, genetic modification status. Provide accession number in repository **OR** supplier name, catalog number, clone number, **OR** RRID.	Yes	Methods
**Animal observed in or captured from the field:** Provide species, sex, and age where possible.	Not Applicable	
Please detail **housing and husbandry conditions**.	Yes	Methods

Plants and microbes	Information included in the manuscript?	In which section is the information available? (Reagents and Tools Table, Materials and Methods, Figures, Data Availability Section)
**Plants:** provide species and strain, ecotype and cultivar where relevant, unique accession number if available, and source (including location for collected wild specimens).	Not Applicable	
**Microbes:** provide species and strain, unique accession number if available, and source.	Not Applicable	

Human research participants	Information included in the manuscript?	In which section is the information available? (Reagents and Tools Table, Materials and Methods, Figures, Data Availability Section)
If collected and within the bounds of privacy constraints report on age, sex and gender or ethnicity for all study participants.	Not Applicable	

Core facilities	Information included in the manuscript?	In which section is the information available? (Reagents and Tools Table, Materials and Methods, Figures, Data Availability Section)
If your work benefited from core facilities, was their service mentioned in the acknowledgments section?	Not Applicable	

Design

Study protocol	Information included in the manuscript?	In which section is the information available? (Reagents and Tools Table, Materials and Methods, Figures, Data Availability Section)
If study protocol has been **pre-registered, provide DOI in the manuscript**. For clinical trials, provide the trial registration number **OR** cite DOI.	Not Applicable	
Report the **clinical trial registration number** (at ClinicalTrials.gov or equivalent), where applicable.	Not Applicable	

Laboratory protocol	Information included in the manuscript?	In which section is the information available? (Reagents and Tools Table, Materials and Methods, Figures, Data Availability Section)
Provide DOI OR other citation details if **external detailed step-by-step protocols** are available.	Not Applicable	

Experimental study design and statistics	Information included in the manuscript?	In which section is the information available? (Reagents and Tools Table, Materials and Methods, Figures, Data Availability Section)
Include a statement about **sample size** estimate even if no statistical methods were used.	Not Applicable	
Were any steps taken to minimize the effects of subjective bias when allocating animals/samples to treatment (e.g. **randomization procedure**)? If yes, have they been described?	Not Applicable	
Include a statement about **blinding** even if no blinding was done.	Not Applicable	
Describe **inclusion/exclusion criteria** if samples or animals were excluded from the analysis. Were the criteria pre-established?<br><br>If sample or data points were omitted from analysis, report if this was due to attrition or intentional exclusion and provide justification.	Not Applicable	
For every figure, are **statistical tests** justified as appropriate? Do the data meet the assumptions of the tests (e.g., normal distribution)? Describe any methods used to assess it. Is there an estimate of variation within each group of data? Is the variance similar between the groups that are being statistically compared?	Yes	

Sample definition and in-laboratory replication	Information included in the manuscript?	In which section is the information available? (Reagents and Tools Table, Materials and Methods, Figures, Data Availability Section)
In the figure legends: state number of times the experiment was **replicated** in laboratory.	Yes	Fig legends
In the figure legends: define whether data describe **technical or biological replicates**.	Yes	All data contain biological repeats only

Ethics

Ethics	Information included in the manuscript?	In which section is the information available? (Reagents and Tools Table, Materials and Methods, Figures, Data Availability Section)
Studies involving **human participants**: State details of **authority granting ethics approval** (IRB or equivalent committee(s), provide reference number for approval.	Not Applicable	
Studies involving **human participants**: Include a statement confirming that **informed consent** was obtained from all subjects and that the experiments conformed to the principles set out in the WMA Declaration of Helsinki and the Department of Health and Human Services Belmont Report.	Not Applicable	
Studies involving **human participants:** For publication of **patient photos**, include a statement confirming that consent to publish was obtained.	Not Applicable	
Studies involving experimental **animals**: State details of **authority granting ethics approva**l (IRB or equivalent committee(s), provide reference number for approval. Include a statement of compliance with ethical regulations.	Not Applicable	
Studies involving **specimen and field samples:** State if relevant **permits** obtained, provide details of authority approving study; if none were required, explain why.	Not Applicable	

Dual Use Research of Concern (DURC)	Information included in the manuscript?	In which section is the information available? (Reagents and Tools Table, Materials and Methods, Figures, Data Availability Section)
Could your study fall under dual use research restrictions? Please check biosecurity documents and list of **select agents and toxins** (CDC): https://www.selectagents.gov/sat/list.htm	Not Applicable	
If you used a select agent, is the security level of the lab appropriate and reported in the manuscript?	Not Applicable	
If a study is subject to dual use research of concern regulations, is the name of the **authority granting approval and reference number** for the regulatory approval provided in the manuscript?	Not Applicable	

Reporting

The MDAR framework recommends adoption of discipline-specific guidelines, established and endorsed through community initiatives. Journals have their own policy about requiring specific guidelines and recommendations to complement MDAR.

Adherence to community standards	Information included in the manuscript?	In which section is the information available? (Reagents and Tools Table, Materials and Methods, Figures, Data Availability Section)
State if relevant guidelines or checklists (e.g., **ICMJE, MIBBI, ARRIVE, PRISMA**) have been followed or provided.	Yes	Methods
For **tumor marker prognostic studies,** we recommend that you follow the **REMARK** reporting guidelines (see link list at top right). See author guidelines, under 'Reporting Guidelines'. Please confirm you have followed these guidelines.	Not Applicable	
For **phase II and III randomized controlled trials**, please refer to the **CONSORT** flow diagram (see link list at top right) and submit the CONSORT checklist (see link list at top right) with your submission. See author guidelines, under 'Reporting Guidelines'. Please confirm you have submitted this list.	Not Applicable	

Data Availability

Data availability	Information included in the manuscript?	In which section is the information available? (Reagents and Tools Table, Materials and Methods, Figures, Data Availability Section)
Have **primary datasets** been deposited according to the journal's guidelines (see 'Data Deposition' section) and the respective accession numbers provided in the Data Availability Section?	Not Applicable	
Were **human clinical and genomic datasets** deposited in a public access-controlled repository in accordance to ethical obligations to the patients and to the applicable consent agreement?	Not Applicable	
Are **computational models** that are central and integral to a study available without restrictions in a machine-readable form? Were the relevant accession numbers or links provided?	Not Applicable	
If publicly available data were reused, provide the respective **data citations in the reference list**.	Not Applicable	
